# The native cistrome and sequence motif families of the maize ear

**DOI:** 10.1371/journal.pgen.1009689

**Published:** 2021-08-12

**Authors:** Savannah D. Savadel, Thomas Hartwig, Zachary M. Turpin, Daniel L. Vera, Pei-Yau Lung, Xin Sui, Max Blank, Wolf B. Frommer, Jonathan H. Dennis, Jinfeng Zhang, Hank W. Bass

**Affiliations:** 1 Department of Biological Science, Florida State University, Tallahassee, Florida, United States of America; 2 Institute for Molecular Physiologie, Heinrich-Heine-Universität, Düsseldorf, Germany; 3 Independent research groups, Max Planck Institute for Plant Breeding Research, Cologne, Germany; 4 Department of Statistics, Florida State University, Tallahassee, Florida, United States of America; University of Minnesota, UNITED STATES

## Abstract

Elucidating the transcriptional regulatory networks that underlie growth and development requires robust ways to define the complete set of transcription factor (TF) binding sites. Although TF-binding sites are known to be generally located within accessible chromatin regions (ACRs), pinpointing these DNA regulatory elements globally remains challenging. Current approaches primarily identify binding sites for a single TF (e.g. ChIP-seq), or globally detect ACRs but lack the resolution to consistently define TF-binding sites (e.g. DNAse-seq, ATAC-seq). To address this challenge, we developed MNase-defined cistrome-Occupancy Analysis (MOA-seq), a high-resolution (< 30 bp), high-throughput, and genome-wide strategy to globally identify putative TF-binding sites within ACRs. We used MOA-seq on developing maize ears as a proof of concept, able to define a cistrome of 145,000 MOA footprints (MFs). While a substantial majority (76%) of the known ATAC-seq ACRs intersected with the MFs, only a minority of MFs overlapped with the ATAC peaks, indicating that the majority of MFs were novel and not detected by ATAC-seq. MFs were associated with promoters and significantly enriched for TF-binding and long-range chromatin interaction sites, including for the well-characterized FASCIATED EAR4, KNOTTED1, and TEOSINTE BRANCHED1. Importantly, the MOA-seq strategy improved the spatial resolution of TF-binding prediction and allowed us to identify 215 motif families collectively distributed over more than 100,000 non-overlapping, putatively-occupied binding sites across the genome. Our study presents a simple, efficient, and high-resolution approach to identify putative TF footprints and binding motifs genome-wide, to ultimately define a native cistrome atlas.

## Author summary

Understanding gene regulation remains a central goal of modern biology. Delineating the full set of regulatory DNA elements that orchestrate this regulation requires information at two scales; the broad landscape of accessible chromatin, and the site-specific binding of transcription factors (TFs) at discrete *cis*-regulatory DNA elements. Here we describe a single assay that uses micrococcal nuclease (MNase) as a structural probe to simultaneously reveal regions of accessible chromatin in addition to high-resolution footprints with signatures of TF-occupied *cis*-elements. We have used maize developing ear tissue as proof of concept, showing the method detects known TF-binding sites. This genome-wide assay not only defines chromatin landscapes, but crucially enables global discovery and mapping of sequence motifs underlying small footprints of ~30 bp to produce an atlas of candidate TF occupancy.

## Introduction

One of the fundamental drivers of phenotypic variation is the activation or repression of gene transcription. Transcriptional gene regulation generally depends on transcription factors (TFs), which bind either directly, or indirectly as parts of complexes, to specific DNA binding sites in gene promoters or distal enhancer sites. Determining where TFs bind genome-wide not only provides insights into transcriptional programs that are active across organs and environmental conditions, it also allows for the identifcation of *cis*-elements and underlying sequence motifs [1,2]. Efforts to identify TF motifs *in vivo* rely mostly on chromatin immunoprecipitation followed by sequencing (ChIP-Seq) [3]. However, ChIP requires a potent and epitope- or TF-specific antibody, and only a few antibodies generally qualify as such [4]. While ChIP-seq does reveal TF-binding sites genome-wide and in the native chromatin context, it does so for a single TF, which dramatically limits its applicability and scalability to characterize entire cistromes. A comprehensive understanding of all TF-binding sites for even a single organ would therefore not only require the prior knowledge of which TFs are present and active, but also thousands of ChIP-seq experiments performed under identical conditions.

Alternative approaches that have proven valuable toward identifying TF-binding sites include *in vitro* based methods such as DNA affinity purification (DAP-seq) [5]. DAP-seq utilizes heterologously-expressed or *in vitro*-translated affinity-tagged TFs incubated with fragmented genomic DNA, allowing TF-DNA footprints to be identified through sequencing and mapping of TF affinity-purified DNA libraries. The DAP-seq technique has major advantages of scalability for defining potential TF-binding sites in purified DNA, but necessarily lacks the native chromatin context, including nucleosomes or transcriptional repressors. DAP-seq is also more suited for TFs that act as homodimers rather than heteromultimeric complexes. Consequently, DAP-seq generally is supplemented with additional empirical evidence from chromatin profiling assays, to support identification of putative TF-bound sites.

Chromatin structure profiling methods, including DNAse-seq, DNS-seq and ATAC-seq, make use of a relatively light endonuclease digests to define accessible chromatin regions (ACRs) [6–12]. These ACRs are associated with transcriptional regulation and share several features including reduced nucleosome occupancies, DNA hypo-methylation, and enrichment of TF-binding sites [13,14]. Unlike ChIP-seq, most chromatin profiling methods do not require antibodies, are more scalable, and identify ACRs within nuclear chromatin. Among these, ATAC-seq further allows direct *in vitro* transposition of sequencing adaptors into chromatin, simplifying library construction and making it widely used to characterize the DNA regulatory landscapes [15]. Despite these advances, ACRs are usually less defined in replicates and average several hundred base pairs [16]. For instance, a recent summary of DNAse hypersensitive sites from the ENCODE project identifies DHSs as typically ranging from 151–240 bp [17]. In contrast, TF-bound sequences and *cis*-regulatory sites are generally much smaller, on the order of 10–20 bp. The relatively large, less defined size of ACRs makes it challenging to identify individual TF-bound sites with certainty and to use them for *de novo* motif discovery [8,18]. One potential strategy to reduce the size of cross-linked DNA-bound fragments is to utilize the exonuclease activities of MNase or other nucleases during sample treatment prior to sequencing [12,19–25]. Cut-&-Run, for example, exploits the property of MNase activity cleaving protein-free but not protein-bound DNA, resulting in high-resolution ChIP-seq footprints of DNA binding sites [22].

Here we define a cistrome of the developing maize ear, including hundreds of thousands of putative protein-occupied loci along with hundreds of underlying TF motif families. To achieve this, we used an approach termed MNase-defined cistrome-Occupancy Analysis (MOA-seq). We characterized the ability of the MOA-seq assay to combine the strength of chromatin profiling methods for ACR identification with the benefits of MNase’s exonuclease and also specific bioinformatic approaches to further refine putative TF-binding sites globally in a given tissue. We used the developing maize ear as a source tissue and proof-of-concept to illustrate the method’s ability to pinpoint known and candidate DNA regulatory sites.

## Results

### A high-throughput approach to identify high-resolution TF footprints genome-wide

**Fig 1:**
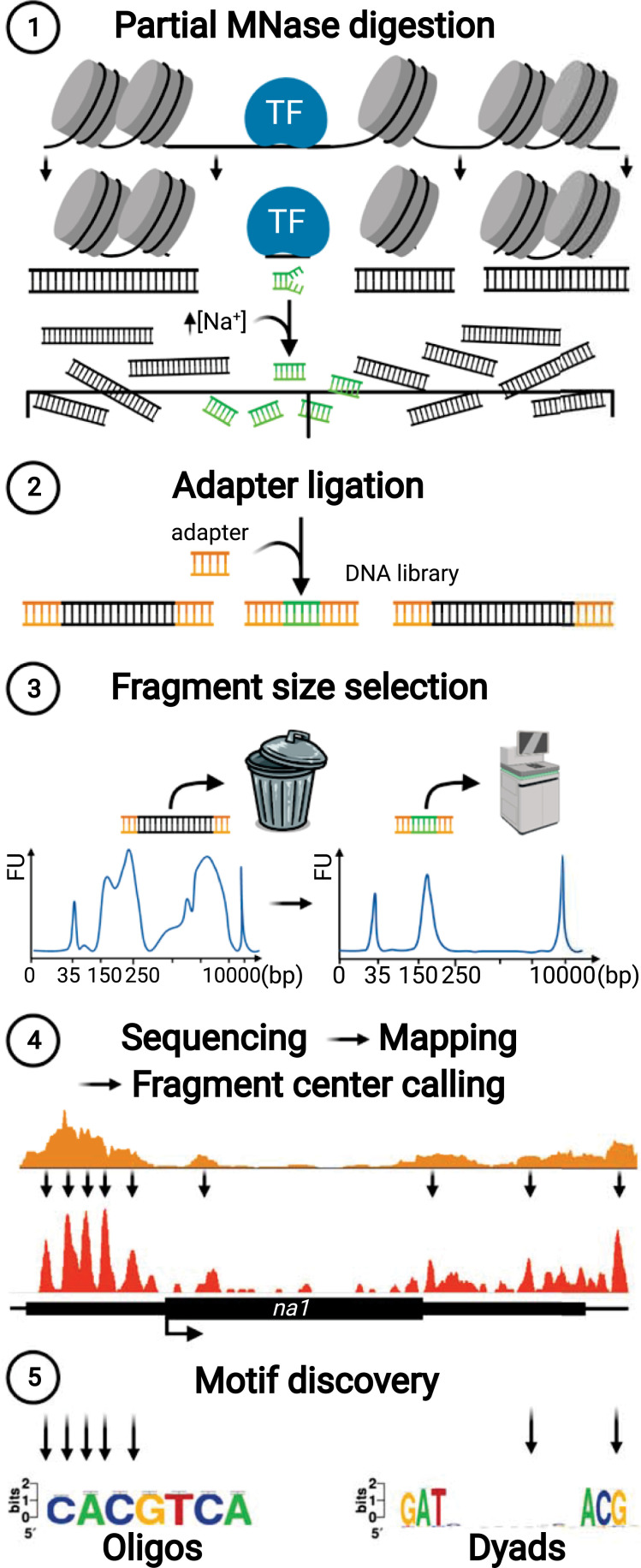
Overview of MOA-seq method. Flowchart summarizes the five key steps that allow for genome-wide, high-throughput and -resolution transcription factor footprinting and candidate motif discovery. Crosslinked, purified nuclei are subjected to light digestion with MNase (Step 1), producing fragments that range from large nucleosome-sized (grey dsDNA) to small, protein-bound (green dsDNA) DNA-footprints. High salt is included during DNA purification and adapter ligation (Step 2) is performed prior to size selection (Step 3). These steps serve to increase the recovery of desired small footprint fragments while efficiently eliminating unwanted nucleosome-sized fragments, reducing the required sequencing depth. After MOA-seq fragments are aligned to the genome (Step 4) as illustrated around the *na1* gene [63]. The resolution of the putative protein/DNA interaction sides can be further enhanced to approximately 30 bp wide footprints by defining fragment centers (MOA-footprints, orange). Sequences underlying MOA-footprints were used as input for *de novo* motif discovery (Step 5), helping to define the maize earshoot cistrome.

To define specific candidate TF-binding sites within accessible chromatin regions, we developed MOA-seq to capture the putative footprints of native DNA-protein interactions. The assay was streamlined to be scalable for high-throughput application and includes a computational pipeline to improve the discovery of putative TF-binding sites as summarized in Fig 1. The protocol (S1 File) starts with the preservation of DNA-protein interactions by formaldehyde-crosslinking prior to tissue homogenization and nuclei extraction. To recover these small interaction regions, we took advantage of the endo- and the exo-nuclease activities of MNase, both of which are inhibited at sites of protein-bound DNA. After MNase treatment, the small DNA fragments can be recovered by reverse crosslinking, protein digestion, and size selection (S1 Fig). To increase the recovery of small (~30–80 bp) TF-bound fragments, while efficiently removing larger nucleosome-size fragments, we added higher salt concentration during the decrosslinking and performed the size selection after adapter ligation during library construction (Fig 1, Steps 1–3). Following sequencing and read mapping to a reference genome (summarized in S1 Table), we plotted the density of aligned fragment midpoints to determine MOA footprints (MFs, average 29.5 bp) and used these to improve the spatial resolution of putative TF-binding event prediction (Fig 1, Step 4). We then performed *de-novo* motif discovery (Fig 1, Step 5) to annotate potential *cis*-elements and compare them to previously defined TF motifs in plants. In summary, this MOA-seq protocol was designed to repurpose MNase from mapping nucleosomes to mapping smaller particles within ACRs, resulting in a simple, scaleable, and antibody-free approach to globaly identify putative TF-bound *cis*-elements at relatively high spatial resolution.

**Fig 2:**
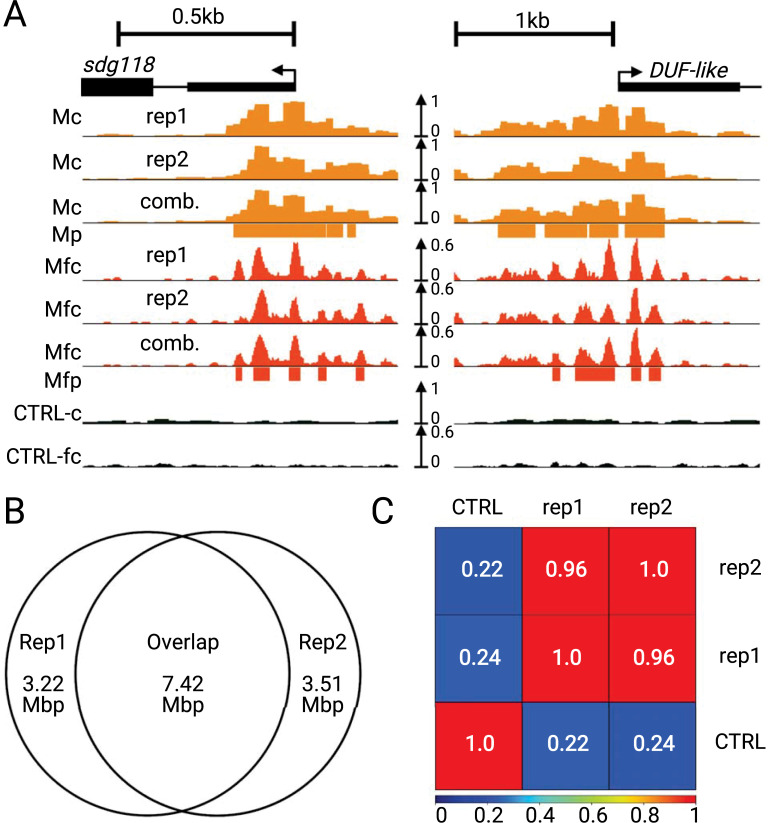
Reproducibility of MOA-seq coverage and footprints from bio-replicate analysis. Comparison of the two bio-replicates, rep1 and rep2, each from ABCD combined (Methods). (A) MOA-seq read coverage (Mc, light orange), MOA footprint coverage (Mfc, dark orange), MNase control coverage (CTRL-c), and MNAse control footprint coverage (CTRL-fc) are shown for two representative areas around the *sdg118* gene (left, Zm00001eb363830) and a DUF-like gene (right, Zm00001eb364010). For each example, the peaks for the combined datasets (Mp and Mfp) are shown. (B) Venn diagram of rep1 and rep2 shows the 1-to-1 quantification of overlapping bases within overlapping peaks. The total shared base pairs (7.42 Mbp) represent 69.7% of Rep1 and 67.8% of Rep2 bases in their respective peaks. (C) Biological replicate correlation analysis for replicate 1 (rep1), replicate 2 (rep2) and the MNase control (CTRL).

To assess the reproducibility of the method we analyzed the bioreplicates, summarized in Fig 2. At the genic level, MOA coverage and MFs showed good agreement between replicates (Fig 2A). This agreement was quantitatively supported genome-wide by multiple different measures of reproducibility and data quality. First, we found that ~70% of bases were shared within significantly enriched peaks that account for only ~0.6% of the genome for each replicate (Fig 2B). Second, the Fraction of Reads in Peaks (FRiP) score was 8.6% for MOA coverage, considerably higher than the 1% threshold recommended by the ENCODE consortium [26].Third, genome-wide coverage correlations were determined because they provide a statistical measure of replicate and control correspondences, independent of peak calling (Fig 2C). We found that the Pearson’s *r* between bio-replicates (*r* = 0.96) far exceeded the ENCODE recommendations for correlation values exceeding *r* = 0.8 [26,27]. In contrast the correlations between the MOA replicates and control were quite low (*r* < 0.25). Based on all of these measures, we concluded that the MOA-seq methodology as performed and described here was decidedly reproducible.

Next we assessed the degree to which MNase sequence-specific bias contributes potential false positive MFs by including a partially MNase-digested chromatin-free DNA input control (Fig 2A and 2C, CTRL; S2 Fig), which did not explain nor match the MOA-seq coverage profiles. Taken together, these findings suggest a good reproducibility of MOA-seq bioreplicates and consistency with previous reports that the known AT-rich bias of MNase does not substantially bias MNase-based nucleosome footprint assays [28].

### MOA-seq identifies shared and unique footprints

**Fig 3:**
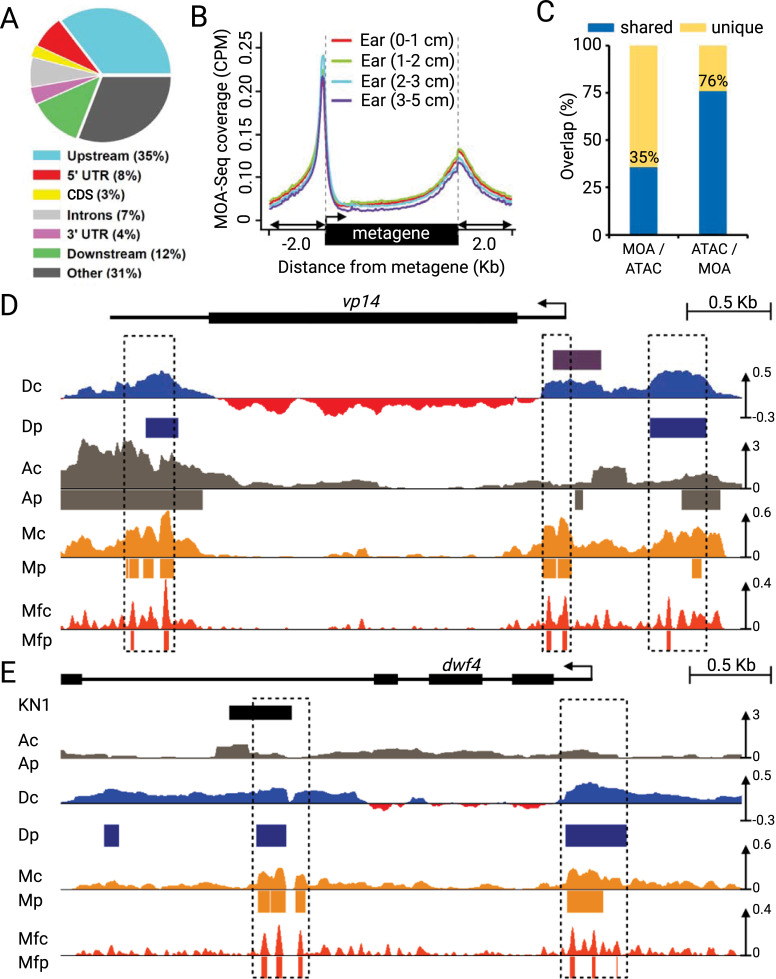
MOA-seq identifies shared and unique regions in open chromatin. Comparison of MOA-seq to open chromatin profiling techniques DNS-Seq and ATAC-Seq. (A) Genome-wide distribution of MOA-seq footprints relative to genomic annotations. (B) MOA-seq read coverage (CPM) is non-randomly distributed around genes. Genes were scaled (metagene) to 2kb and average coverage is shown within the metagene body and adjacent genomic regions +/- 1.5kb from the nearest metagene boundary (grey dash). (C) Comparative analysis, shown as bar graphs, of MOA-seq peaks overlapping (shared, blue, %) or non-overlapping (unique, yellow) with ATAC-seq (MOA/ATAC), and the reciprocal intersections (ATAC/MOA). (D) and (E) Distribution of previously published DNS-seq (Positive light-heavy digest = blue, negative = red), ATAC-Seq (grey) coverage, MOA-seq (light orange) coverage (Mc) and peaks (Mp), and MF (dark orange) coverage (Mfc) and peaks (Mfp), surrounding example genes, *vp14* (D) and *dwf4/brs1* (E), with direction of transcription and the TSS indicated (arrow).

Transcription factors preferentially bind directly upstream and downstream of the transcription start sites (TSSs) and transcription termination sites (TTSs), respectively, while being depleted in protein-coding regions. To test whether MFs showed a similar pattern, we analyzed their genome-wide distribution relative to genes, as shown in Fig 3. In our analysis, almost half (47%) of all MFs flanked genes. The second-largest fraction of MFs (31%) were located in intergenic regions, while the smallest fraction (3%) overlapped with protein-coding regions (Fig 3A). The highest enrichment of MFs was observed directly upstream and downstream the TSS and TTS, respectively (Fig 3B). Overall this pattern reflects typical TF-binding sites and open chromatin patterns around genes and intergenic enhancer regions. We previously showed that MNase-based differential nuclease sensitivity profiling could be used to map functional regions of the maize genome [29]. Because both the DNS-seq and MOA-seq make use of light-digest MNase fragments, albeit in different ways, to define accessible chromatin [30], we expected that the two would mark similar genomic regions. Indeed, by inspection and peak overlap analysis, the two methods were congruent (S3 Fig), and confirmed genome-wide with shared peaks accounting for 59% of all peaks from MOA-seq and 67% of all peaks from DNS-seq.

Since MOA-seq and ATAC-seq peaks also share a similar distribution pattern, but deploy different nucleases, we analyzed the number of shared sites between them. For this, we compared our data with recently published ATAC-seq data from similar-stage earshoots [31,32], the best available dataset for comparison. While the majority (76%) of ATAC-seq peaks overlapped with one or more MOA-seq peaks, only a minority (35%) of MOA-seq peaks overlapped with one or more ATAC-seq peaks (Fig 3C). Although ATAC and MOA peaks collectively capture a similar fraction of the genome (ATAC, 0.52%; MOA-seq, 0.55%), MOA-seq did identify more, smaller, and unique peaks compared to ATAC-seq (S2 Table). However, given the differences in peak sizes and numbers, some of these differences could be due to differences in the statistical cutoffs between the segmentation algorithms. Reanalyzing the ATAC-seq data with iSeg, we found similar overlaps. The higher number of peaks called for MOA-seq does not necessarily indicate, therefore, a higher performance.

The differences in the distribution of MOA and ATAC-seq peaks were observed as distributed over large regions covering hundreds of thousands of bases as well as at the genic level (S4 Fig). The latter is illustrated, e.g., for the well-characterized abscisic acid biosynthetic pathway gene *viviparous14* (Fig 3D, *vp14*) and the brassinosteroid biosynthesis gene *dwarf4* (Fig 3E, dwf4). Many MOA-seq and ATAC peaks were shared in the vicinity of these two genes. However, MOA-seq identified additional peaks, not detected by ATAC, which overlapped with accessible chromatin regions also detected by DNS-seq in earshoot [7] as well as TF-binding sites such as *teosinte branched1* (*tb1*) and *knotted1* (*kn1*) (Fig 3D and 3E). These findings illustrate that MOA-seq and ATAC-seq map ACRs globally with considerable agreement, especially around promoter regions, whereas MOA-seq often further resolves subregions or identifies additional regions (S5 Fig), similar to that described for Arabidopsis [12].

### MOA-seq maps to functional elements

**Fig 4:**
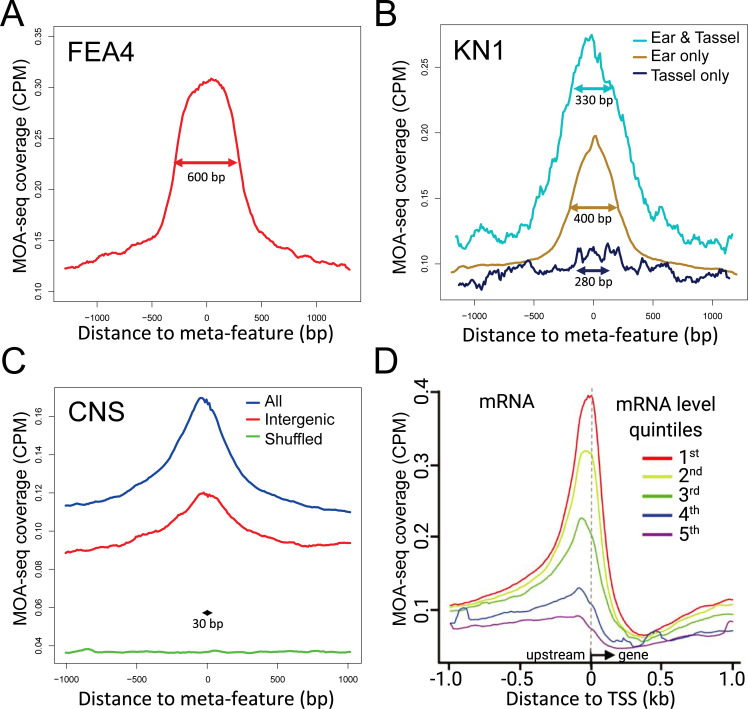
MOA-seq overlaps with TF-binding sites and correlates at promoters with mRNA levels. Average earshoot MOA-seq coverage was plotted around the midpoints for FEA4 tassel ChIP-seq peaks (A), Knotted1 ChIP-seq peaks subdivided into Ear only, Ear & Tassel, or Tassel only (B), and conserved non-coding sequences (CNS) showing all, position-shuffled control (Shuffled), or an intergenic subset classified as being more than 1 kb from genes (C). For each plot, the average peak size was used to define a single metapeak size (metapeak, indicated in each plot) for fitting the data within the peak region; metapeak flanking regions are in real bp relative to the metapeak region. (D) MOA-seq coverage is positively correlated with gene regulation. Mean MOA-seq coverage for genes divided into quintiles (1^st^ highest mRNA to 5^th^ lowest mRNA level) according to mRNA levels in earshot tissue.

Given the expectation that MOA-seq identifies regulatory regions enriched for *cis*-elements, we examined MFs at known and predicted TF-binding sites, as shown in Fig 4. We first analyzed the MOA-seq average coverage at ChIP-seq sites previously published for the TF encoded by *fasciated ear4* (*fea4*) [33] which is active in both ear and tassel. Although tassel FEA4 ChIP-seq data was used, MOA-seq coverage peaked at FEA4 binding sites, compared to the surrounding area (Fig 4A). In the case of the KNOTTED1 (KN1) [34] transcription factor, both tassel and ear ChIP-seq data was available. Similar to FEA4, MOA-seq coverage peaked at KN1 binding sites present in both tassel and ear as well as sites unique to the ear. The tissue-specific binding of KN1 was mirrored by the MOA-seq coverage (Fig 4B). In addition to TF-binding sites, we examined evolutionarily-defined conserved noncoding sequences (CNS) which are enriched for *cis*-regulatory elements [35]. We found that MFs aligned with CNSs, even at intergenic sites far removed from known promoters (Fig 4C).

Previous studies demonstrated that gene expression levels and chromatin accessibility show positive correlation at proximal promoters [29,30]. To examine this relationship for MOA-seq in a genome-wide manner, we sorted the 36,441 maize genes into quintiles based on their steady-state mRNA levels from matched earshoot tissues, and inspected MOA-seq profiles around the TSSs. Across expression quintiles, we detected a positive correlation between average gene expression levels and MOA-seq read coverage, most clearly evident within ~300 bp upstream of the TSSs (Fig 4D). Together, these analyses establish compelling evidence for our starting hypothesis that MOA-seq footprints can define regulatory loci likely to be occupied by DNA-binding proteins.

### MOA-seq footprints identify hundreds of putative TF motifs

Global chromatin structure assays such as ATAC-seq, DNAse-seq, and DNS-seq identify accessible chromatin regions, but their larger average footprint profiles may reduce the accuracy of footprint analysis [36]. In contrast, given the small average size of 29.5 bp for MFs (S2 Table), and their presence at known functional *cis*-elements, the MOA-seq-defined footprints should be ideal for *de novo* motif and putative TF-binding site discovery, all of which aims to elucidate genome-wide native cistromes.

**Fig 5:**
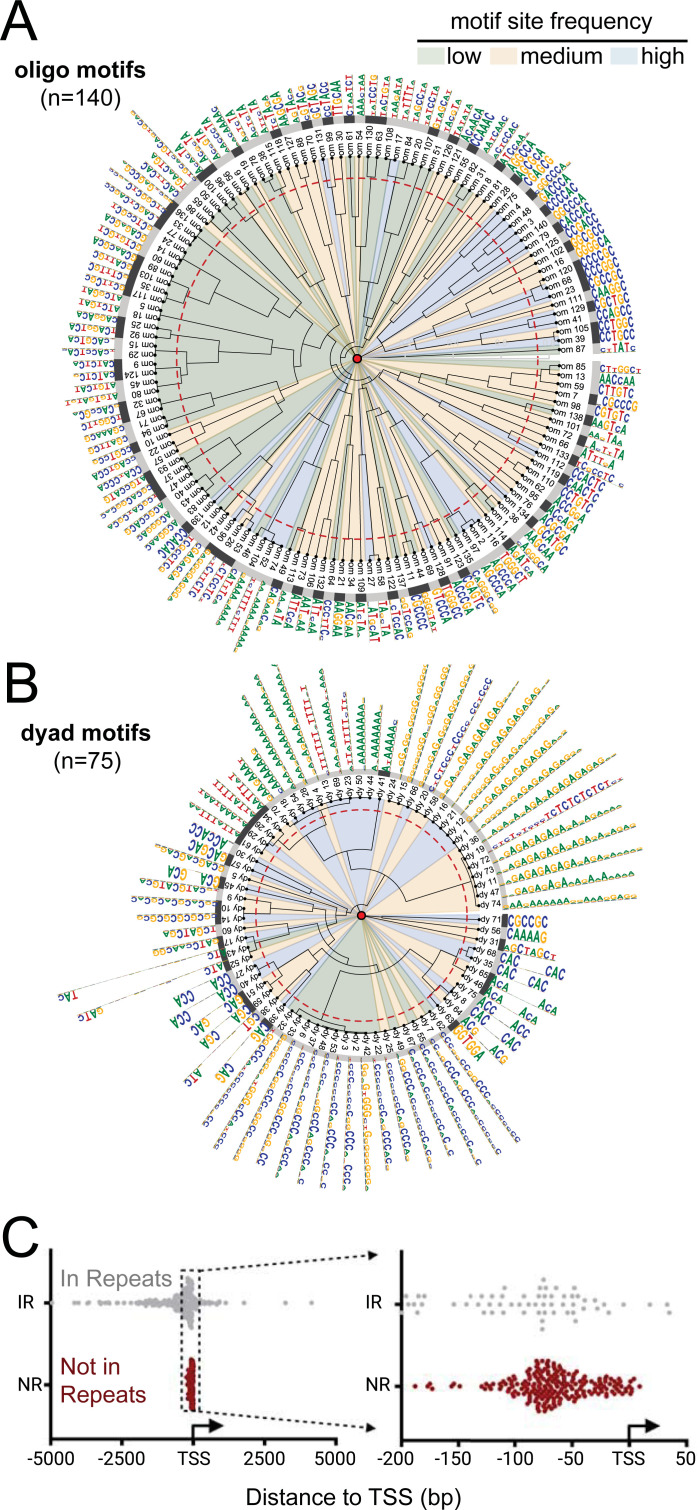
Global summary of maize earshot motifs discovered by MOA-seq. (A-B) Radial similarity-cluster tree of the 140 oligo (A) and 75 dyad (B) consensus motifs identified within MOA-seq footprints. Circular sectors underlying tree branches are colored to highlight the frequency at which each motif occurred (high > 2000, medium 2000–1000, low < 1000). The inner (red dash) colored ring indicates similarity threshold for motif clustering at 90% similarity, delineating different motif clusters (alternating light and dark gray ring) flanked by motif names and consensus sequence logos at the inner and outer circles, respectively. (C) Median distance to the nearest transcription start site (TSS) plotted for each motif found in regions annotated as within repeats (IR, grey) or in non-repetitive regions (NR, red).

We used RSAT (http://rsat.sb-roscoff.fr/) for *de novo* discovery of enriched motifs within the 143,009 MFs (S2 Table, iSeg-BC7 for B73v3) as input genomic sequences. We identified a total 215 significantly enriched motifs (S3 Table), summarized in the matrix similarity tree shown in Fig 5 and separated for the two types of motif discovery methods used: the 6–7 bp long "oligos" (Fig 5A, n = 140) or the "dyads" (Fig 5B, n = 75). The number of individual sites for individual MOA-identified motifs ranged from less than 200 to more than 6,000. We compared the 215 MF motifs with those previously identified both *in-vivo* and *in-vitro* by ChIP-Seq and DAP-Seq, respectively, using multiple plant motif databases. Among the full collection of motifs, 85% (119/140) of the oligo motifs and 75% (56/75) the dyad motifs showed similarities to motifs listed in at least two motif databases (S3 Table, including footprintdb [37], and softberrydb [38]). To characterize their distribution relative to genes, we split them into groups of those found within repetitive DNA, or not. The frequency of repeat-overlapping motifs ranged from 3% for *dym05* to 88% for *dym43*, with a median value 15% for the 215 motifs. Plotting their positional tendencies relative to genes, we found that the median distance to TSS for motifs not within repeats showed a remarkable clustering within 100 bp proximal to the promoters (Fig 5C). Higher resolution TSS annotations [39] may better refine the positional locations of these motifs. For each of the 215 motifs, we produced summary catalog files for the dyad motifs (S6 File) and the oligos motifs (S7 File). These files provide reference documents, one page per motif family, listing the total number of sites, percentage found in annotated repeats, the sequence LOGOs, positional frequency distributions around TSSs, local base frequencies, and average local MOA coverage.

### MOA-seq footprints improve the spatial resolution of TF binding event prediction

**Fig 6:**
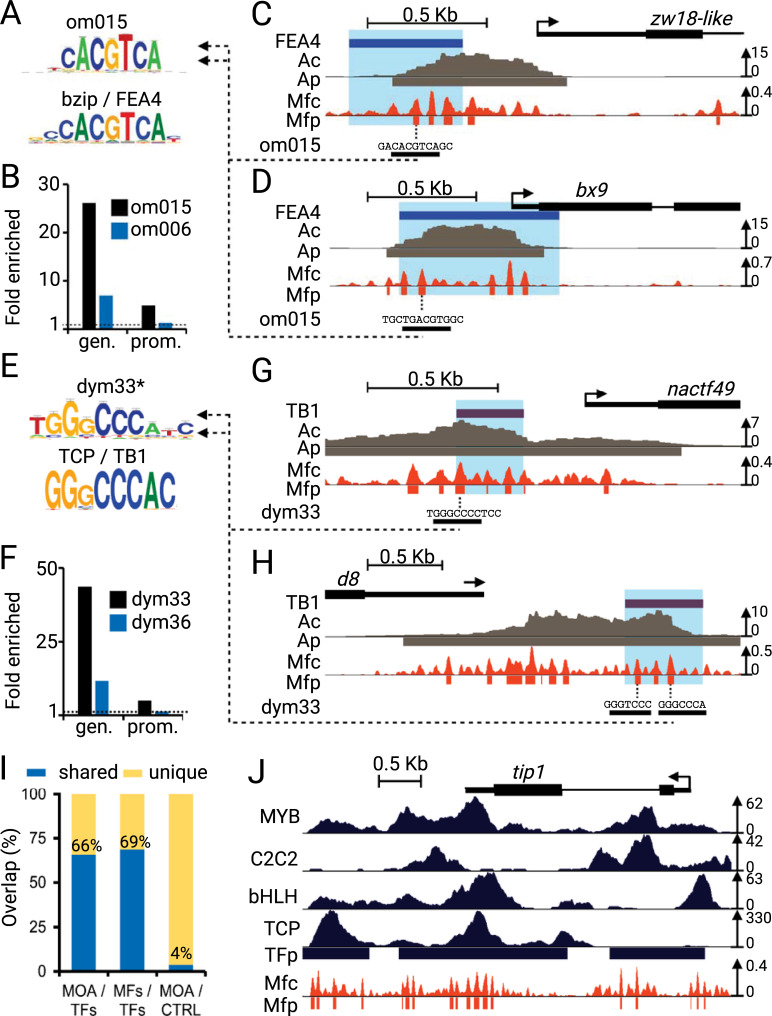
MOA-seq footprints improve the spatial resolution of TF binding event prediction. (A-F) ChIP-seq binding peaks of (A-B) FEA4 or (E-F) TB1, ATAC-Seq coverage (Ac) and peaks (Ap), MOA-seq footprint coverage (Mfc) and peaks (Mfp), and MOA-seq motif om015 in the promoter region of example genes (A) *zw18-like* (GRMZM2G039505), (B) *bx9* (GRMZM2G161335), (E) *nactf49* (GRMZM2G347043), and (F) *d8* (GRMZM2G144744). (C, G) MOA-seq motif (C) om015 (G) dym33, aligned to their best database hit (footprintdb) of bzip and TCP family, respectively. The asterisk for (G) dym33 indicates that only a portion of the larger dym33 logo is shown. (D, H) Enrichment of motifs (D) om015 and a control motif om006 within FEA4 ChIP-seq peaks, or (H) dym33 and control motif dym36 with TB1 ChIP-seq peaks, relative to the mappable B73 genome (gen.), and the 3 kb, non-ORF overlapping, region upstream of B73 gene models (prom.). (I) Genome-wide overlap analysis of base pairs shared between MOA (MOA, MFs) and ChIP-seq of 104 TFs [27] or the MNase control (CTRL). (J) Representative example of the complex landscape of TF and MFs profiles surrounding *tip1* (Zm00001eb00373). TF tracks shown: MYB, TF55 rep1 GSM4095564; C2C2 TF235 rep2 GSM4095532,; bHLH, TF6 rep2 GSM4095589; TCP, TF237 rep1 GSM4095534.

Depending on the size of the sheared DNA, ChIP-seq data usually identifies regions substantially larger than the underlying TF-binding site(s) even for TFs in multimeric complexes. Given the relatively small size of MFs, we tested whether the motifs identified at MFs were enriched at known TF-binding sites which also have the canonical motif. We found that 93% (3882/4195) and 82% (2770/3396) of the earshoot-expressed *FEA4* and *TB1* TF ChIP-seq peaks, respectively, overlapped with at least one MF. We then tested whether those overlapping MFs were enriched for MOA-defined motifs that closely resemble the canonical motif for the respective TF (summarized in Fig 6). The oligo motif *om015* (Fig 6A) closely resembles the database motif for the bzip TF family as well as the published FEA4 consensus sequence, NCGTCA [10]. Genome-wide, *om015* and a similarly abundant control motif *om006* were enriched 6.9- and 1.2-fold, respectively, at FEA4 binding sites within 3 Kb gene promoters (Fig 6B, prom.). Similar enrichment trends were also observed in the entire mappable genome (Fig 6B, gen.). Examples of this overlap can be seen within FEA4 ChIP-seq binding sites upstream of putative target genes *zw18-like* (Fig 6C) and *bx9* (Fig 6D). Similarly, a database search of the dyad motif *dym33* revealed that part of its consensus sequence closely resembled the consensus motifs of the TCP TF family as well as that of one of its members, TB1, GGNCCC [40] (Fig 6E). Genome-wide, *dym033* was enriched 11.7-fold at TB1 ChIP-binding sites in 3kB gene promoters, compared to only a 1.3-fold enrichment of a more abundant *dym36* MOA-motif (Fig 6F). Examples of this overlap can be seen within TB1 ChIP-seq binding sites upstream of putative target genes *nactf49* (Fig 6G) and downstream of *d8* (Fig 6H). For the two examples (FEA4-like *om015*, TB1-like *dym33*), we found that the matching MOA motif families showed the expected enrichment at the corresponding TF ChIP peaks (S6 Fig). Notably, of the 215 motif families, *om015* was ranked 2nd for enrichment at FEA4 ChIP peaks, whereas *dym33* was ranked 3rd for enrichment at TB1 ChIP summits. Similarly, among the top 5 motifs enriched at KN1, the motif family *om050* includes two matches to the core TGCA motif associated with KN1 binding sites [34]. More broadly comparing MOA peaks to the binding sites of 104 TFs identified by ChIP-seq we found that 66% and 69% of the base pairs in MOA coverage and footprint peaks, respectively, were shared with those under the TF ChIP-seq peaks, compared to only 4% for peaks from the MNase control (Fig 6I). In addition to this global agreement, we observed genic level overlap illustrated by the TF vs. MF profiles around the *tip1* gene (Fig 6J). Given that members of TF families often share similar motifs, and that multiple members of a TF family can be co-expressed, there may exist some discrepancies between the consensus sequence of motif families defined from our global assay with MOA-footprints compared to those from individual TF ChIP assays.

### MOA-seq footprints occur at chromatin interaction and distal enhancer regions

**Fig 7:**
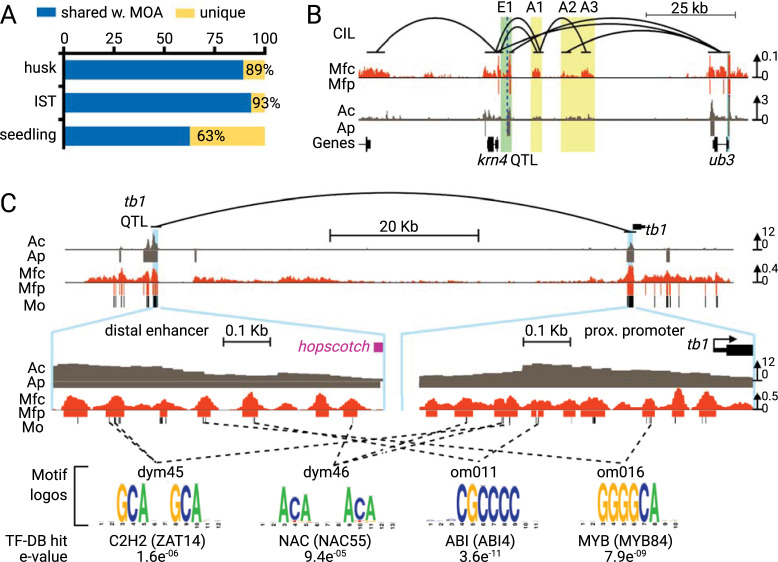
MOA-seq footprints highlight known long-distance chromatin interaction. Genome-wide overlap analysis of base pairs shared between MOA and previously defined enhancers (husk, IST, from [13]) and long-range interaction sites (seedling, from [41]). (B-C) Examples of MOA-seq coverage and peaks (Mfc, Mfp, dark orange) at known long-range interaction sites compared to ATAC-seq coverage and peaks (Ac, Ap, gray) at the (B) UB-KRN4 region and the (C) TB1 region. (B) Panel shows chromatin interaction (CI, black lines) loops inferred from HiC, UB2 ChIP-seq, and H3K27ac- and H3K4me3-mediated ChIA-PET-seq data for the *KRN4* and *UB3* region, three high-confidence remote sites (A1, A2 and A3; yellow columns), and the locations of *KRN43*.*1* (light green column), and *UB3* promoter (light blue column) [45]. (C) Interaction region of the -65 Kb distal enhancer region (left zoom-in panel) and promoter region (right zoom-in panel) of *TB1*. Motif names, positions (Mo) and consensus logos, identified within MOA-seq footprints, are indicated. Dashed lines connect consensus motifs identified in both interacting regions.The best database hit (footprintdb) with the similarity p-value are indicated.

In addition to gene-proximal promoters, distal enhancers and long range chromatin contacts play important but not fully understood roles in gene regulation. Proximity ligation methods for detecting 3D chromatin contacts, such as HiC and ChIA-PET have been instrumental in the identification of long distance chromatin interactions. We analyzed MOA-seq coverage at previously characterized candidate enhancer and chromatin interaction sites, summarized in Fig 7. We found that the majority of maize candidate enhancers in husk and inner shoot tissue [13] and long-range chromatin interaction sites in seedlings [41] overlapped with MOA peaks (Fig 7A). To explore these at a regional level, we examined two well-characterized long-distance regulatory elements in maize around the genes *tb1* and *ub3*, both of which are related to important agronomic traits [42,43]. We analyzed the MF profile at these long-distance chromatin interaction regions, and compared it to that of ATAC-seq. *KERNEL ROW NUMBER4* (*KRN4*) is an intergenic quantitative trait locus that presumably contributes *cis*-regulatory elements to the control of the inflorescence gene *UNBRANCHED3* (*UB3*). *KRN4* is bound by the UB2 (Fig 7B, E1) transcription complexes and interacts with the *UB3* promoter (Fig 7B, light blue highlight) by at least three duplex interactions (Fig 7B, A1, A2 and A3, yellow highlight) that affect *UB3* expression [44,41,45]. We observed clear MF footprints at all previously annotated KRN4/UB2/UB3 interactions [44,45] in this region, even in those where ATAC-seq coverage was relatively low (Fig 7B).

The 65 Kb upstream distal enhancer region of the *TB1* gene includes large-effect quantitative trait loci associated with plant morphology traits [46]. The distal control region includes a hopscotch transposon insertion site which enhances the expression of TB1 in domesticated maize compared to its wild ancestor, teosinte, affecting apical dominance and tiller bud dormancy [42]. Inspecting the MOA-seq profiles, we detected strong MFs in the proximal *tb1* promoter and distal upstream region (Fig 7C, light blue highlights). Given their long-distance interaction and the known regulatory role of the hopscotch insertion site in *tb1* expression, we speculated that both regions may share regulatory elements. To test this hypothesis, we inspected motifs identified within MFs for the 600 bp regions directly upstream of the hopscotch insertion (Fig 7C, distal enhancer zoomed area) and *tb1* TS site (Fig 7C, proximal promoter zoomed area). Among the motifs found at these two relatively small regions, we found four different motifs, two dyad and two oligo motifs, that were indeed shared between the distal and proximal regions. Comparing these motif sequences to the footprintdb [37] database revealed significant overlaps with consensus motifs of *dym45* with the C2H2-type zinc finger, *dym46* with a NAC, *om011* with an ABI, and *om016* with the MYB TF family. Taken together, all of the findings reported here demonstrate the potential for MOA-seq to pinpoint candidate promoter TF-binding sites at promoters and intergenic loci.

## Discussion

A comprehensive understanding of gene regulation requires at least the knowledge of all *cis*-acting targets of regulatory factors genome-wide. Over the last decade regulatory regions have been mapped using nuclease sensitivity assays, such as DNAse-seq, ATAC-seq or various MNase-based approaches, to identify ACRs [6,18]. These ACRs are known to be enriched for TF-binding sites and contain similar amounts of functional variation compared to gene coding regions [8]. However, given the relatively large size of ACRs [17], pinpointing the best candidates for *cis*-elements remains challenging and often limited to individual TFs showing overlap with ChIP-seq data.

The MOA-seq approach reported here leverages the desirable properties of MNase as a probe of accessible chromatin regions and shares initial steps with other MNase-based protocols [21,30,10,12] but is distinct in steps that optimize recovery of small fragments potentially originating from TF/DNA interactions and the refinement of those footprints. MOA-seq includes optimizations during library construction aimed to enhance scalability and reduce loss of small fragments, resulting in fewer PCR cycles being required for sufficient library concentrations. Together, these key steps make MOA-seq applicable to high-throughput experiments. In order to refine our ability to define potential *cis*-targets of TF-occupied sites at higher resolution, we defined and analyzed fragment centers to achieve < 30 bp MOA-seq footprints (Fig 1A, see supplementary methods). Defining the midpoints of genome-aligned fragments of MNase digested chromatin has been proven useful, originally to define the center of nucleosome positions [47], and later for sub-nucleosomal particles [10,22]. Accordingly, we have incorporated fragment midpoint analysis as a key step (Fig 1, Step 4) defining the footprints expected to be centered on DNA sequence elements bound by their cognate DNA binding protein.

To date, relatively few studies have used MNase to examine small fragments from subnucleosomal particles in plants [21,10,12]. According to a recent study in Arabidopsis, Tn5 digest recovers only a subset of the accessible chromatin as compared to MNase [12]. Similarly, we observed that in maize, MOA-seq identified nearly two times more regions than ATAC, while sharing almost 80% of ATAC discovered regions (Fig 3). This difference has been primarily attributed to the larger molecular weight of the various nucleases used (Tn5, 53 kDa; DNaseI, 32 kDa vs. MNase, 17 kDa). In the case of ATAC-seq the relatively large Tn5 “motif” [15,18], and/or the complex cleavage mechanism requiring a Tn5 dimer may contribute to some of the observed differences. Other reasons, such as the use of crosslinking, preferred for MOA-seq but not ATAC-seq, or plant growth and harvest conditions, could also contribute to differences between MOA-seq and other chromatin accessibility profiling methods.

TFs mostly bind to short, specific DNA sequences allowing the determination of motifs for recognized *cis*-targets [48,49]. Previous studies have used open chromatin assays such as DNAse-seq and ATAC-seq to identify potential TF footprints within ACRs and their related motifs. Recent progress in footprint calling approaches have offered multiple strategies of increasingly high-resolution for motif discovery using open chromatin assays such as ATAC-seq [16,50]. We found that despite their small size, the MFs were significantly enriched for sequences similar to those previously identified as potential motifs for TFs using *in silico*, *in vitro*, or *in vivo* methods [37]. It will be important to validate these predicted motifs by functional assays to establish what proportion of them are genuinely bound by TFs.

Comparing our MOA-seq vs. ChIP-seq for more than 100 TFs showed strong, but not complete overlap (Fig 6I), suggesting that these methods identify similar genomic sites. However the lack of a complete overlap could result from one of a few possibilities, including the differences in tissue (earshoot vs. leaf mesophyll), the methodology (homogenized tissue vs. protoplast), or analytical methods. In addition, we can not exclude the possibility that the multiple different cell types in our earshoot could result in failure to detect footprints, especially those from minority cell types. Lastly, the large TF study sampled most of the TF families, but not all their members, which could also contribute to the lack of complete overlap. Despite this overall agreement between MOA-seq and ChIP-seq data (~67% overlap), we observed an exception for the ChIP-seq peaks of the inflorescence TF RAMOSA1 [51]. For the 735 peak regions shared between the two RA1 ChIP seq datasets, we observed relatively low overlap with peaks from either MOA-seq (22%) or ATAC-seq (20.5%). Despite these small values, the RA1-intersecting peaks of MOA and ATAC showed good agreement (78.4% in common) with each other. Possible explanations for this exception could be the repetitive nature of the top RA1 candidate binding site (GA_n_), which may reduce unique mappability, especially given the short reads of both MOA and RA1 ChIP-seq sequencing. Another possibility is the RA1 may be less abundant in the developmentally later earshoots analysed by MOA-seq or ATAC-seq compared to those used for the RA1 ChIP-seq.

In addition to local TF overlaps, we also found considerable coincidence of MFs with previously identified intergenic enhancers and long-distance chromatin interaction sites. This overlap was particularly strong for enhancers defined via multiple epigenomic marks and chromatin accessibility [13]. However, we can not exclude the possibility that some of these intergenic sites may be non-annotated genes. Consistent with this idea, we observed that some of these candidate enhancers displayed gene-like features such as RNA coverage or were annotated as genes in previous B73 assemblies (S7 Fig).

Merging all overlapping motif sites into contiguous intervals, we detected 107,745 MF merged regions (averaging 16 bp) that all together represent <0.1% of the maize genome. Whereas we expect that the motifs found will include some false positive sites, almost 80% of them overlapped with putative TF DNA-binding sites in at least two plant databases. Additional analyses and integration with other epigenomic information will be key to advance functional tests needed to ascertain the predictive power and myriad hypotheses generated from knowledge of these motifs. This approach and the resulting cistrome atlas represents the most comprehensive map of putative TF-binding sites produced for a crop species. This relatively simple and scalable genome-wide native chromatin structure assay is expected to be applicable to attempts to broadly define and analyze gene regulatory networks. Knowledge of chromatin landscapes should help focus genome editing and accelerate larger applied research efforts such as those guiding precision agriculture and medicine.

## Methods

### Plant materials

Earshoots from B73 wild-type maize were harvested from field-grown plants during mid-morning. The tissue harvesting for materials used in this paper is the same as that used for nuclease sensitivity profiling, DNS-seq, as previously described [7]. For the materials used in this study, field-grown (Mission Road Research Facility, Dept. Biological Science, Tallahassee, FL, USA) earshoots were harvested between 9-11am on sunny days of June 8th, 9th, and 13th of 2015. Earshoot samples were rapidly harvested, measured, and immediately frozen in liquid nitrogen, pooled by date of harvest and size class and stored at -80°C. Multiple earshoots were ground frozen in liquid nitrogen, followed by subsequent aliquoting of the frozen powder for multiple preparation replicates. Size A = pool of 15–20 earshoots of length 0.5–1 cm, size B = pool of 15–20 earshoots of length 1–2 cm, size C = pool of 8 earshoots of length 2–3 cm, and size D = pool of 8 earshoots of length 3–5 cm.

### MOA-seq and MNase control library sequencing

The MOA-seq bench protocol is provided (S1 File). It includes tissue fixation, nuclei isolation, MNAse digestion, library preparation, and library size selection. The size-selected indexed libraries were subjected to an equimolar pool of 10 libraries (summarized in S1 Table). The 10 libraries correspond to 2 replicates of each size class, A, B, C, and D, and technical replicates of the two B samples. The technical replicates are from the production of two different libraries made from the light digest pools for B biorep1 and B biorep2. All 10 libraries were separately size-selected, quantified, and subjected to equi-molar pooling for NSG using Illumina HiSEq 2500 paired-end 50-cycle sequencing (FSU College of Medicine, Translational Science Laboratory, https://med.fsu.edu/translationalLab/home). For the MNase control library, total maize DNA was purified using Qiagen plant (greenhouse-grown, v5 leaf blade, B73) DNA purification kit and used for partial digest titrations similar to that for MOA-seq. Specifically, we carried out a partial digest, but with reducing the MNase concentration by 1/64th to achieve the same level of partial digest used for MOA. DNA purification and library construction and size selection was done exactly as described above for MOA-seq and subjected to 100 bp PE sequencing on the MGI-2000(DNBSEQ-G400) platform. Raw sequences from the MOA-seq libraries and MNase control were deposited to the NCBI Sequence Reads Archive (https://www.ncbi.nlm.nih.gov/sra) under the BioProject ID PRJNA477338. The bio-replicate analysis showed high similarity between all (Pearson’s *r* > 0.9) and earshoot library bio-replicates were pooled into "rep1" or "rep2" for the two bio-replicates, or as "combined" for an earshoot pool of all 10 libraries used for peak calling.

### MOA-seq data processing, genome alignment and analysis

The demultiplexed, “raw” sequence data (fastq format) was processed to trim the 3’ adaptor sequences with CutAdapt without filtering for low-quality bases at the 3’ or 5’ end (-q = 0). The minimum overlap length required between read and adapter was set to 1 (-O = 1). All other parameters were set to their defaults in CutAdapt version 1.16 [52]. Trimmed reads were aligned to the B73v3 and B73v5 reference genome assemblies with bowtie 2 using the following options: end-to-end,—no-mixed,—no-discordant, minimum fragment length = 0 (-I = 0), maximum fragment length = 1000 (-X = 1000). All other parameters were set to their defaults in Bowtie 2 version 2.3.1 [53]. Aligned reads were processed using various programs from the *BEDTools* suite [54,55], as described below. The bam files were combined with "samtools merge" across samples into pools for all biological and technical replicates for all the "B" sized earshoots (indicated as B_all or Bc_all) or for the pools of rep1, rep2, or all (combined). Mate-pairs of reads were specified using “samtools fixmate” and suspected PCR duplicates were marked with “samtools markdup.” Combined, deduplicated bam files were then converted to bed format with “bedtools bamtobed”. Further analyses were done on both resulting aligned fragments of all sizes, and ≤80 bp fragments. Fragment densities were calculated genomewide using “bedtools genomecov.” Coverage files in 10 bp windows were calculated utilizing “bedtools makewindows” and “bedtools intersect.” Resulting bedgraph files were then converted to bigwig format with “bedGraphToBigWig” (S1 Zip File, pooled coverage file used for peak segmentation).

To further increase the resolution of MNase-sensitive footprints, we calculated the fragment centers (also called "frenters" or "midpoints") by extracting the geometric centers of each aligned fragment with “awk,” and intersected these midpoints with 21 bp genomic windows with a step size of 5 bp. Resulting bedgraph files were then converted to bigwig format for UCSC genome browser display using “bedGraphToBigWig”.

### iSeg peak calling and analysis

We applied the iSeg peak calling algorithm [56] to detect peaks in each MOA-seq read coverage file using a range of "BC" stringencies as previously applied to DNS-seq data [7]. The parameters and their values for segmenting MOA-seq data are listed below: (1) iSeg Biological Cutoff (BC) value: A BC value of 1.0 means that the height of a significant segment has to be greater than 1.0 x standard deviation of the data mean. We set the BC values to: 1.0, 2.0, 3.0, 4.0, 5.0, 7.0 and 9.0 for segmenting MOA-seq. (2) Minimum and maximum window length: These values are set allowing iSeg to scan a large number of segments within these window lengths. The settings used are minimum window length of 20 bp and maximum window length of 120 bp. (3) Standard Deviation (SD) and median absolute deviation (MAD): While not necessary in general, these values are pre-computed in our application by calculating sample SD and sample MAD for each MOA-seq file when calling its peaks. The global non-zero regions are determined by the regions in the combined COVERAGE pool that had at least one read in any sample. When computing the sample statistics, we removed the global zero regions in each MOA-seq to reduce the degree of distortion caused by sparsity. The INPUT files were bedGraph format with four tab-separated entries: chrom, chromStart, chromEnd, dataValue. The iSeg OUTPUT files are text files, tab-delimited tables with six columns (chrom, SegmentStart, SegmentEnd, meanHeight, t-statistics, p-value). If needed, adjacent book-ended peaks or those separated by 1 bp were merged to produce the final peaks BED files. We used the first three columns to generate BED format files (S1 Zip File for MOA-seq coverage peaks; S2 Zip File for MFs) and converted it into bigBed format for browser display purposes. Peak overlap analyses were done using the "Table Browser" tools from the UCSC Genome Browser [57] using the "Intersection" function with the "any overlap" setting. To optimize the comparisons across different datasets and genome assemblies (B73v3 and B73v5) we used a genomic fraction equivalency approach to select peaks that captured 0.5–0.7% of the genome for MOA coverage or control profiles and 0.1–0.2% of the genome for MOA fragment centers (MFs) or control fragment center profiles (S2 Table).

### RNA purification, sequencing, and analysis

RNA was prepared from ~0.1g/samples of frozen ground powder from the same aliquots described above for MOA-seq, with replicates matching the MOA seq sample design. The mRNA was purified using RNeasy Plant MiniKit (Qiagen 74904) and submitted for library preparation and sequencing (Molecular Cloning Facility, Florida State University, Tallahassee, FL, USA). The 10 RNA-seq libraries were prepared and sequenced and available from NCBI Sequence Reads Archive BioProject PRJNA477338. For analysis of RNA-seq data, trimmed and QC filtered Fastq sequence reads were mapped to the B73v3 reference genome (AGPv3.22) using STAR v. 2.7.1a [58] in two-pass mode with additional parameters:—outSAMstrandField intronMotif,—outFilterType BySJout,—outFilterIntronMotifs RemoveNoncanonical,—quantMode TranscriptomeSAM GeneCounts. Unique reads were filtered by mapping quality (q13) and PCR duplicates removed using Samtools (v. 1.3.1). Transcript accumulation was analyzed in R (v. 3.6) using the DEseq2 software (v. 1.24.0) [59]. The combined pooled sample was used to examine the transcript abundance versus MOA coverage (Fig 4D).

### Comparative analysis of MOA-seq to other genomic annotations

Several published or shared datasets were analyzed. We obtained a recently published dataset of ATAC-seq peaks from nuclei isolated from 1 cm field-grown earshoots [31,32]. For *knotted1* [34] and *fasciated ear4* [33] we obtained published ChIP-seq peaks and used their genomic coordinates as central features to plot the average local MOA-seq coverage. For conserved noncoding sequences, CNS, we obtained a public but unpublished dataset (courtesy of Liang, Zhikai; Schnable, James (2019): Conserved Noncoding Sequences. figshare. Dataset: https://doi.org/10.6084/m9.figshare.7804268.v1).

This CNS dataset, called CNS_ZL_v319c in this study, was produced as part of a comparison with the STAG-CNS methods previously described by Lai et al. [60].

### Motif discovery, nomenclature, and analysis

Motif discovery was done using the RSAT ChIP motif discovery tool and integrated [61] [62]. The MOA-seq footprints (MFs) from iSeg peaks called at bc7 (S4 File, peaks BED file) were used to specify genomic sequences as input for RSAT. For the RSAT pipeline, peak regions were used to extract genomic DNA sequences using bedtools (vers. 2.27.1). Some of the peaks were below the minimum size limit for RSAT input (24 bp). For these, we expanded the peaks to 24 bp to retain them in the input data. The data was analyzed using default settings except: the oligo length for all analyzes was set to 6 and 7, and the markov order was set to M = 1. For the motif discovery the following TF motif databases were included: ArabidopsisPBM (11/2015), Athamap (11/2015), cisBP (Ath, 6/2015 v1.02), Cistrome by DAP-seq (6/2016), footprintDB-plants (6/2018) and JASPAR core nonredundant plant motifs (2018). NsitePL with the PlantProm database [38], which includes 3,032 previously identified plant TF-binding sites found in 576 experimentally tested promoter sequences, was further used to identify putative TFs and motifs underlying MOA-seq peaks. The full results RSAT reports for the two methods are in the supporting information zip files (S3 Zip File for dyads; S4 Zip File for oligos) and combined into a browser-compatible all motifs BED file (S4 File).

## Supporting information

S1 Fig MOA-seq library preparation. (A) Analytical agarose gels of purified, decrosslinked DNA for each sample (see Methods for A-D earshoot sizes) following digestion with MNase titrations. Lanes 1 (Mr) contain DNA size markers; lanes 2–8 correspond to digest levels (U/mL MNase for M1 = 80, M2 = 40, M3 = 20, M4 = 10, M5 = 5.0, M6 = 2.5, M7 = 0). For each of the eight light-digest libraries, the 2–3 digestions chosen for pooling are indicated (yellow boxes). (B) Agilent Bioanalyzer electropherograms (red line trace plots) for the 10 libraries after BluePippin-based size selection. Inset box shows upper (purple line) and lower (green line) internal size standards marked in the densitometry plots for all ten final libraries with sample and library ID table. Ear shoot size class key: Ac = A1+A2, Bc = B1+B1T1+B2+B2T1, Cc = C1+C2, Dc = D1+D2.
(EPS)

S2 Fig Close-up comparison of MOA coverage, MFs, and MNase control. Comparison of MOA coverage, MFs, and MNAse control footprint coverage surrounding *ramosa3*. (A-B) Normalized MOA-seq read coverage (MOA Cov, orange), MNase control coverage (MNase CTL COV), and MOA minus MNAse control coverage (MOA COV CTL subtracted) as well as MFs (MOA FRENTERs), MNAse control FRENTERs (MNase CTL FRENTERs), and MF minus MNase CTL FRENTERs are shown at (A) the same scale and (B) with each track’s y-axis autoscaled. The profile substructures of MNase CTL and MOA/MF, even when zoomed by autoscaling, do not appear to exhibit matching patterns.
(EPS)

S3 Fig MOA-seq read coverage and genome-wide distribution compared to DNS-seq. (A) Browser screenshot from a 100 kb region around the maize *tb1* gene showing congruence of MOA-seq profiles with those of open chromatin from DNS-seq [7]. Browser tracks show "Clean Repeats excluding dust," from plants.ensembl.org (repeats), conserved non-coding sequences, CNS_ZL_v319c (CNS), gene models from Ensembl/Gramene (genes), earshoot iSeg peaks called at stringency bc2.0 (ES DNS iSeg bc2.0), earshoot differential nuclease sensitivity (ES DNS) profiles with positive/blue MNase-sensitive regions and negative/red MNase-resistant regions, earshoot MOA-seq coverage for 1-2mm earshoots (ES MOA (B) cov, S5 File) and associated iSeg peaks at four stringences (ES MOA (B) iSeg bc1,3,5, & 7), earshoot MOA-seq coverage for 0.5-5cm earshoots (ES MOA (A-D) cov) and associated iSeg peaks at four stringences (ES MOA (A-D) iSeg bc1,3,5, & 7), and DNS profiles for coleoptile node (CN), root tip (RT), and 15 day endosperm (EN). The combined MOA-seq coverage data and peaks called at iSeg bc5 used for subsequent analysis are highlighted (boxes & arrows). (B) 7 kbp zoom of the region ~ 60 kb upstream of *tb1* showing substructure (arrows) of MOA-seq relative to open chromatin mapped by DNS-seq. (C) Bar chart showing % overlap of combined ES-MOA peaks at iSeg-bc5 with DNS-seq positive peaks at iSeg-bc2 for tissues indicated below the graph. (D) Bar chart showing % overlap of ES DNS-seq positive peaks with the three other DNS-seq tissues using iSeg-bc2 for all comparisons.
(EPS)

S4 Fig MOA-seq compared to two other earshoot open chromatin profiling methods, DNS-seq and ATAC-seq. Genome browser views of regions of the genome showing MOA-seq peak segments from this study along with previously published comparable earshoot peaks of open chromatin profiling assays from MNase-based DNS-seq (Turpin et al., 2018 [7] and ATAC-seq [31]. The peak segment tracks are from MOA-seq (MOA ES Cov iSEG bc5.0, orange), ATAC-seq (ATAC peaks EAR, grey), or DNS-seq (ES DNS+ iSeg Peaks bc2.0, dark blue). Other tracks are displayed as described in S3 Fig. (A) A 1 Mb view of the maize b73v3 genome (Chr5:204,507,240–205,507,239) showing similarity of regions with peaks from MOA-seq and ATAC-seq. (B) A 10 kb view of the maize genome (Chr1:265,738,394–265,748,393) located ~ 70 kbp upstream of *tb1* gene highlighting regions where all three assays have called peaks (green-dashed boxes). (C) A 7 kb view of the maize genome (Chr5:65,137,565–65,144,564) covering the *na2* gene highlighting promoter and 5’ gene regions where the MOA-seq and DNS-seq but not ATAC-seq assays have called peaks (blue-dashed boxes). (D) A 12 kb view of the maize genome (Chr3:176,842,911–176,854,910) covering the *lg2* gene highlighting a genic region where the MOA-seq and ATAC-seq but not DNS-seq assays have called peaks (grey-dashed box). Dark orange arrows indicate examples of regions identified by MOA-seq but not ATAC-seq in panels A, C, and D.
(EPS)

S5 Fig Chromatin accessibility profiles averages around MOA peaks at ATAC-shared or MOA-only sites. MOA coverage peaks were split into two groups, those that do (Shared sites, blue) or do not (MOA-only sites, red) overlap ATAC peaks [31]. The read depth-normalized ATAC and MOA coverage for the shared peaks group was set to 100% for comparison. The ATAC coverage at MOA-only sites showed a large reduction (>5 fold) compared to shared sites, indicating the MOA-only sites are considerably less accessible to ATAC.
(EPS)

S6 Fig Motif family enrichment at TF ChIP peaks for FEA4, TB1, and KN1. ChIP-seq peaks for three TFs [34,40,33] were used to intersect MF motifs. The motif families were ranked according to the percent per family that intersected peaks for each TF. The top five MOA-seq Motif families are and the families shown (yellow highlight) and sequences associated with the binding sites (red) are indicated.
(EPS)

S7 Fig MOA Coverage and MFs around potentially non-annotated genes. MOA coverage around annotated previously characterized intergenic enhancers [13], distal regulatory regions [44], or TF-binding sites [27] are shown for two examples (A, B) from genome browser windows as described in S3 and S4 Figs. Peak segments (top bars) are shown along with MOA (orange) and MF (dark orange) coverage (cov.), fragment centers (frenters) and peaks, MNase control coverage and fragment centers, and TF ChIP-seq coverage (TF ChIP cov.) These regions, initially classified as intergenic, show some gene characteristics (not depicted) such as overlapping mRNA coverage, uninterrupted open-reading frame and TF-binding sites up and downstream.
(EPS)

S1 File PDF MOA-seq bench protocol. MOA-seq bench protocol.
(PDF)

S2 File Bigwig file of read-normalized MOA coverage for B73v3. Read-normalized coverage from combined libraries aligned to B73v3 and used as input for peak segmentation with the iSeg algorithm. The bigwig file is published and available via FigShare, https://doi.org/10.6084/m9.figshare.13012529.v1.
(DOC)

S3 File Bigwig file of MFs coverage for B73v3. MFs (fragment centers, frenters) file produced using a sliding window average to enhance detection of groups of reads with shared centrally-located regions, combined from for all libraries (ABCDc) aligned to B73v3 and used as input for peak segmentation with the iSeg algorithm. The bigwig file is published and available via FigShare, https://doi.org/10.6084/m9.figshare.13012553.v1.
(DOC)

S4 File MOA-seq motifs BED file, full list from B73 earshoot RSAT result using peak sequences from MFs. All motifs, dyads and oligos, from RSAT of earshoot MFs peaks as iSeg cutoff bc7 (footprints herein designated MFs), aligned to B73v3. The BED file name combines the motif family name, its consensus sequence, and the matching local sequence: "name"_"rsatMotifCons"_"ExactLocalSeq". Motif orientation is indicated as strand (+ or -) and motifs can self-overlap as either palindromes or partially offset matches. The entire set has 215 motifs are indexed to 344,201 total sites which can be merged into 107,745 non-overlapping contigs.
(BED)

S5 File Bigwig file of MOA-seq normalized coverage for 1-2cm "B" sized earshoots. Read-normalized coverage from combined biological and technical replicates of libraries corresponding to "B" (1-2cm) sized earshoots, aligned to B73v3 and used for MOA coverage trend plots. The bigwig file is published and available via FigShare, https://doi.org/10.6084/m9.figshare.13014143.v1.
(DOC)

S6 File PDF summary catalog files for the "DYAD" motifs found with RSAT under MOA-seq footprints (MFs, frenter peaks). This PDF file provides a reference document, one page per motif, listing the assigned RSAT-dyad motif name (e.g. *dym01*), total number of sites in B73v3, percentage found in annotated repeats, the consensus and sequence LOGOs from RSAT reports, the median TSS-relative position and motif frequency histograms around the TSSs of the filtered gene set (FGS), the local base count composition flanking the motif midpoints, and average local MOA-seq coverage centered on the motifs for all (All) motifs, or those motifs split into either not overlapping/in repeats (NR) or overlapping/in repeats (IR).
(PDF)

S7 File PDF summary catalog files for the "OLIGO" motifs found with RSAT under MOA-seq footprints (MFs, frenter peaks). This PDF file provides a reference document, one page per motif, listing the assigned RSAT-oligo motif name (e.g. *om001*), total number of sites in B73v3, percentage found in annotated repeats, the consensus and sequence LOGOs from RSAT reports, the median TSS-relative position and motif frequency histograms around the TSSs of the filtered gene set (FGS), the local base count composition flanking the motif midpoints, and average local MOA-seq coverage centered on the motifs for all (All) motifs, or those motifs split into either not overlapping/in repeats (NR) or overlapping/in repeats (IR).
(PDF)

S8 File MOA earshoot COVERAGE rep1 bigwig file for B73v5. Read-normalized coverage from combined bio-replicate 1 libraries of earshoot MOA-seq aligned to B73v5 in 20 bp window bins. The bigwig file is published and available via FigShare, https://doi.org/10.6084/m9.figshare.14411084.v1.
(DOC)

S9 File MOA earshoot COVERAGE rep2 bigwig file for B73v5. Read-normalized coverage from combined bio-replicate 2 libraries of earshoot MOA-seq aligned to B73v5 in 20 bp window bins. The bigwig file is published and available via FigShare, https://doi.org/10.6084/m9.figshare.14411099.v1.
(DOC)

S10 File MOA earshoot COVERAGE combined bigwig file for B73v5. Read-normalized coverage from combined libraries of earshoot MOA-seq aligned to B73v5 in 20 bp window bins and used as input for peak segmentation. The bigwig file is published and available via FigShare, https://doi.org/10.6084/m9.figshare.14411075.v1.
(DOC)

S11 File MOA earshoot MFs rep1 bigwig file for B73v5. Read-normalized FRENTERs profiles from combined bio-replicate 1 libraries of earshoot MOA-seq aligned to B73v5 in 5 bp window bins. The bigwig file is published and available via FigShare, https://doi.org/10.6084/m9.figshare.14411849.v1.
(DOC)

S12 File MOA earshoot MFs rep2 bigwig file for B73v5. Read-normalized FRENTERs profiles from combined bio-replicate 2 libraries of earshoot MOA-seq aligned to B73v5 in 5 bp window bins. The bigwig file is published and available via FigShare, https://doi.org/10.6084/m9.figshare.14412110.v1.
(DOC)

S13 File MOA earshoot MFs combined bigwig file for B73v5. Read-normalized MF (FRENTER) profiles from combined libraries of earshoot MOA-seq aligned to B73v5 in 5 bp window bins and used as input for peak segmentation. The bigwig file is published and available via FigShare, https://doi.org/10.6084/m9.figshare.14411117.v1.
(DOC)

S14 File B73 MNase Control COVERAGE bigwig file for B73v5. Read-normalized coverage profile from MNase partial digest DNA control aligned to B73v5 in 20 bp window bins and used as input for peak segmentation. The bigwig file is published and available via FigShare, https://doi.org/10.6084/m9.figshare.14412551.v1.
(DOC)

S15 File B73 MNase Control fragment centers bigwig file for B73v5. Read-normalized FRENTER (fragment center) profile from MNase partial digest DNA control aligned to B73v5 in 5 bp window bins and used as input for peak segmentation. The bigwig file is published and available via FigShare, https://doi.org/10.6084/m9.figshare.14412851.v1.
(DOC)

S1 Table Library, sequence processing, and alignment metrics. Summary of MOA-seq library and alignment statistics.
(XLSX)

S2 Table Summary of Peaks Data. Summary statistics for peak segments called for MOA-seq coverage and MF profiles. Individual bed file available in S1, S2, and S5 Zip Files.
(XLSX)

S3 Table Summary information for MOA-seq RSAT motifs of B73v3 earshoot cistrome. The 215 motifs (75 dyad plus 140 oligo) are sorted by family name and include information about abundance, consensus sequences, and average genic location.
(XLSX)

S1 Zip File Zipped BED files of iSeg peak calls for earshoot MOA-seq read coverage on maize B73v3. Bed files of peak segments called by iSeg at a series of cutoffs; bc 1, 3, 5, 7, and 9. MOA-seq read- and quantile-normalized fragment coverage scores were used as the input. Included are the 5 BED files for B73v3, a readme (txt) file, and a summary statistics table (xlsx) captured from Table Browser for files on UCSC genome browser, genomaize, for each iSeg bigbed source file.
(ZIP)

S2 Zip File Zipped BED files of iSeg peak calls for earshoot MFs (frenters) on maize B73v3. Bed files of peak segments called by iSeg at a series of cutoffs; bc 1, 3, 5, 7, and 9. MFs (sliding window capturing fragment midpoint clusters) scores were used as the input. Included are the 5 BED files for B73v3, a readme (txt) file, and a summary statistics table (xlsx) captured from Table Browser for files on UCSC genome browser, genomaize, for each iSeg bigbed source file.
(ZIP)

S3 Zip File RSAT report for 75 DYAD motifs found at MFs (frenters) iSegBC7 peaks. RSAT report for motif discovery of DYADs type search pattern, using input genomic sequences under MOA-seq maize earshoot MFs with settings: extension to 24bp if <24, n500, min6, max7, pat250, no database, max1000bp. The unzipped file produces folders & files summarizing the motif families. The Zip file is published and available via FigShare, https://doi.org/10.6084/m9.figshare.13012670.v1.
(DOC)

S4 Zip File RSAT report for 140 OLIGO motifs found at MFs (frenters) iSegBC7 peaks. RSAT report for motif discovery of OLIGOs type search pattern, using input genomic sequences under MOA-seq maize earshoot MFs peaks with settings: extension to 24bp if <24, n500, min6, max7, pat250, all databases, max1000bp. The unzipped file produces folders & files summarizing the motif families. The Zip file is published and available via FigShare, https://doi.org/10.6084/m9.figshare.13012673.v1.
(DOC)

S5 Zip File Zipped BED files of iSeg peaks, bigbed files for rep1, rep2, and combined earshoot MOA-seq read COVERAGE on maize B73v5. Bed files of peak segments called by iSeg at a series of cutoffs (bc 1, 2, 3, 4, 5, 7, and 9 for each replicate; bc 1, 3, 4, 5, 7, and 9 for combined). MOA-seq read- and quantile-normalized fragment COVERAGE scores from B73v5 alignments for replicate 1, replicate 2, or combined were used as the input. Included are the multi-series output iSeg BED files for each input, and a readme (txt) file.
(ZIP)

S6 Zip File Zipped BED files of iSeg peaks, bigbed files for rep1, rep2, and combined earshoot MFs (frenters) on maize B73v5. Bed files of peak segments called by iSeg at a series of cutoffs (bc 1, 2, 3, 4, 5, 7, and 9 for each replicate; bc 1, 3, 4, 5, 7, and 9 for combined). MOA-seq read- and quantile-normalized MF (frenters) coverage scores from B73v5 alignments for replicate 1, replicate 2, or combined were used as the input. Included are the multi-series output iSeg BED files for each input, and a readme (txt) file.
(ZIP)

S7 Zip File Zipped BED files of iSeg peaks, bigbed files for MNase CONTROL coverage (COV) and MFs (FRENTERs) on maize B73v5. Bed files of peak segments called by iSeg at a series of cutoffs (bc 1, 2, 3, 4, 5, 7, and 9) for MNase DNA-only control digests coverage (COV) or MF-like (FRENTERs) profiles as the input. Included are the 7-series output iSeg BED files for each input, and a readme (txt) file.
(DOC)

S8 Zip File MOA-seq Motifs BED files for B73v5 mapped via BLAST-over from B73v3. Zip file contains a readme text file and 2 BED files: a Single Base BED file (Moa Motif Mid Pt) for UCSC browser, chromosomes only, for B73v5, BLASTED from B73v3 source FASTAs with 100 bp flanks each side, retained if 100% match and reduced down to central base (Andorf, Bass, 2021); and a motif BED file (Moa Motif Full Motif) reduced back down to the motif and retaining the motif name and the B73v3 motif family consensus and exact genomic match sequences.
(ZIP)
