# Supplementary material for: The native cistrome and sequence motif families of the maize ear
Source: PLoS Genet. 2021 Aug 12;17(8):e1009689. doi: 10.1371/journal.pgen.1009689 (PMC8360572; doi:10.1371/journal.pgen.1009689)
Supplement: S1 File — MOA-seq bench protocol. (PDF) [file pgen.1009689.s008.pdf]

MOA-seq Bench Protocol

This protocol is a modification of the DNS-seq chromatin sensitivity profiling methods that we have developed and previously described (Turpin et al. 2018). DNS-seq uses both light and heavy MNase digests, whereas MOA-seq uses only light digest with additional steps to ensure sufficient yield and recovery of small DNA fragments. This protocol describes the MOA-seq protocol used for this study (SD Savadel, DL Vera, T Hartwig, HW Bass et al). Optional tested revisions found useful are noted.

(**2018**) Turpin ZM, Vera DL, Savadel SD, Lung, P-Y, Wear EE, Mickelson-Young L, Thompson, WF, Hanley-Bowdoin L, Dennis, JH, Zhang J, and Bass HW. "Chromatin Structure Profile Data from DNS-seq: Differential Nuclease Sensitivity Mapping of Four Reference Tissues of B73 Maize (*Zea mays* L)". ***Data in Brief*** 20:358-363 (DOI: 10.1016/j.dib.2018.08.015).

Materials

0.5 M EGTA: 0.5 M EGTA in ddH_2O pH 8.0 (adjust with NaOH), sterilize with 0.2 µm filter.

1:1 Phenol:chloroform pH 8.0

10% formaldehyde: 10% formaldehyde from high-quality paraformaldehyde in ddH_2O. Prepare fresh and use in fume hood.

10% SDS:10% SDS in ddH_2O, sterilize with 0.2 µm filter.

10% Triton X-100: dissolve 10% Triton X-100 in ddH_2O overnight with constant mixing, sterilize with 0.2 µm filter.

1000x Dithiothreitol (DTT): 1.0 M DTT, 0.01 M Sodium acetate in ddH_2O, sterilize with 0.2 µm filter. Aliquot and store at -80ºC.

1000x Polyamines: 0.15 M Spermine tetra HCl, 0.5 M Spermidine in ddH_2O, sterilize with 0.2 µm filter. Aliquot and store at -80ºC.

10X BFA salts: 150 mM PIPES, 800 mM KCl, 200 mM NaCl, 20 mM EDTA, and 5 mM EGTA in ddH_2O pH 6.8 (adjust with NaOH), sterilize with 0.2 µm filter. Aliquot and store at -80ºC.

150 µm Partec filters (Sysmex Partec)

2.5 M Glycine: 2.5 M Glycine in ddH_2O, sterilize with 0.2 µm filter.

20,000 U/mL MNase: Dissolve 45,000 Units of lyophilized MNase in 2.25 mL MNase storage buffer.

200 mM Phenanthroline: 200 mM Phenanthroline in Isopropanol

200 mM Phenylmethylsulfonyl fluoride (PMSF): 200 mM PMSF in Isopropanol.

3M Sodium acetate: 3 M Sodium acetate in ddH$_2$O pH 5.2 (adjust with acetic acid), sterilize with 0.2 µm filter.

5X Sorbitol: 1.6 M Sorbitol in ddH$_2$O, sterilize with 0.2 µm filter. Aliquot and store at -80ºC.

DAPI: 10 µg/mL DAPI in ddH$_2$O

Fixation Buffer: 6 mL ddH$_2$O, 1 mL 10X Buffer A Salts, 2 mL 5X sorbitol, 10 µL 1000X DTT, 10 µL 1000X polyamines , 10 µL 200 mM PMSF, 10 µL 200 mM phenanthroline, 1 mL 10% formaldehyde. Prepare immediately before use in a fume hood and directly dispense after mixing.

Miracloth (EMD Millipore)

MNase Digestion Buffer (MDB): 50 mM HEPES pH 7.6, 12.5% Glycerol, 25 mM KCl, 4 mM MgCl$_2$, 1 mM CaCl$_2$, sterilize with 0.2 µm filter.

MNase Digestion Buffer + 1% Triton X-100 (MDBT): MDB made to include 1% Triton X-100.

MNase Storage Buffer: 10 mM Tris-HCl, 50 mM NaCl, 1mM EDTA, 50% Glycerol, pH 7.5, sterilize with 0.2 µm filter.

NEBNext Ultra II DNA Library Prep kit (New England Biolabs)

Proteinase K: 20 mg/mL Proteinase K in Tris-HCl pH 8.0, 50% Glycerol

TE: 10 mM Tris-HCl pH 7.6, 1 mM EDTA-NaOH pH 8.0, autoclave and pass through 0.2 µm filter.

TER: 10 mM Tris-HCl pH 7.6, 1 mM EDTA-NaOH pH 8.0, 40 µg/mL RNase A, autoclave then 0.2 µm filter prior to addition of RNAse A.

TR: 10 mM Tris-HCl pH 7.6, 40 µg/mL RNase A, autoclave, then 0.2 µm filter prior to addition of RNase A.

TT-20 Buffer; 10 mM Tris-HCl pH 8, 0.2% Tween-20, autoclave, then 0.2 µm filter prior to the addition of Tween-20.

Specialist Equipment:

Agencourt AMPure XP beads (Beckman Coulter)

DynaMag-2 Magnetic 1.5 mL Tube rack (Thermo Fisher Scientific)

"Qubit" Dye-based DNA concentration fluorometer (Thermo Fisher Scientific)

Agilent 2100 Bioanalyzer for analysis of DNA fragment size and concentration

High sensitivity DNA kit (Agilent)

Polytron PT 10-35 tissue disruptor (Kinematica)

==

MOA-seq Protocol for 1 Gram of Maize Earshoot Tissue

(or for ~1 mL Ground Tissue Powder)

==

1 Isolation of Formaldehyde-Fixed Nuclei from Frozen Tissue.

1. Harvest tissue as rapidly as practical[1] by freezing in liquid Nitrogen[2].
2. Grind tissue to complete fine powder under liquid nitrogen without thawing.[3]
3. Set aside at least 10% frozen, unfixed tissue for optional future analyses, such a RNA-seq or proteomics.
4. Distribute ~1.0 g frozen tissue powder per 50 mL conical[4].

---

[1]Tissues should be rapidly harvested under closely matched conditions, including time of day. Tissues should be flash-frozen in liquid nitrogen within 10-60 seconds of harvest. These flash-frozen tissues can be stored at -80ºC indefinitely.

[2] Liquid Nitrogen is a potentially hazardous cryogen. Protective hand and eye wear must be worn at all times when handling liquid nitrogen. Non-reinforced containers (such as polypropylene tubes) of liquid nitrogen cannot be sealed before the nitrogen evaporates, or it might build pressure and explode the sealed container.

[3]Materials and equipment necessary for this step should be labeled, arranged, and pre-chilled prior to removing frozen tissues from -80ºC storage. Vent (1) 50 mL polypropylene conical tube / frozen sample by puncturing three (3) holes in the lid of each tube with a 28 gauge syringe to prevent explosive pressure from building above liquid nitrogen. Prechill all conical tubes on dry ice. Pre-chill mortar and pestle by filling mortar with liquid nitrogen with pestle inside. Transfer tissue directly into a liquid nitrogen-filled mortar and grind to a fine powder under nitrogen, slowly adding liquid nitrogen as needed to prevent thawing.

[4] Tissue must be kept on dry ice until all nitrogen has *completely* evaporated. Vented lids can then be replaced with new, intact lids. Tissue aliquots may be stored at -80ºC long-term or held on dry ice for immediate processing.

5. Fix the tissue by addition of 10 mL of fresh-made Fixation Buffer[5] containing 1% formaldehyde. Allow fixation to proceed for 10 minutes at RT with constant gentle mixing[6].
6. Stop fixation reaction by addition of 0.1 vol (~1 mL) of 2.5 M glycine to achieve a final concentration of 250 mM glycine, with constant, gentle mixing at RT for 5 minutes.
7. Remove ~50 µL of fixed cells for Microscopic Check 1 "MC1" and hold at 4ºC.
8. Dilute fixed tissue into MDB[7] to a final volume of 45 mL.
9. Concentrate fixed tissue by centrifugation at 1500 rcf for 5 minutes at RT.
10. Resuspend fixed tissue in 4 mL MDB with 1% Triton X-100 (MDBT).
11. Remove ~50 µL of fixed cells for Microscopic Check 2 "MC2" and hold at 4ºC or DAPI stain and check at this point to confirm tissue breakage and nuclei release[8].
12. Further mechanically disrupt tissues with a Polytron (3 x 10 seconds at ~ 1/5 maximum speed) to liberate nuclei[9].
13. Filter lysate through a sterile funnel lined with 1 layer of miracloth and collect into a new 15 mL polypropylene conical tube.
14. Remove ~50 µL or Microscopic Check 3 "MC3" and hold at 4ºC to confirm the presence of nuclei or nuclei-containing cell fragments.
15. Dilute nuclei into a final volume of 15 mL MDB.[10,11]

---

[5] 10 mL Fixation Buffer per ~1g tissue aliquot must be prepared fresh at room temperature just before use from constituent solutions (see materials).

[6] Complete fixation buffer should be mixed with a 10 mL pipette and directly dispensed to a ~1g aliquot of frozen tissue powder. Aggregates of frozen tissue must be dispersed prior to incubation at room temperature to ensure complete fixation of powdered tissue. Failure to disperse tissue aggregates during resuspension will increase the frequency of nuclei with abnormal morphology such as ruptured nuclei or degraded chromatin. Once the tissue is resuspended, place on rotary shaker, nutator, or rotating mixer.

[7] Dilution to ~50 mL decreases the density of the sorbitol-containing fixation buffer to allow fixed cells to pellet during centrifugation for maximum recovery of fixed cells prior to lysis.

[8] Qualitative examination of DAPI-stained nuclei at 40x magnification at this step is sufficient to confirm successful lysis of fixed cells. If very large aggregates of cellular debris with few intact nuclei are present, it may be necessary to subject the sample to further mechanical disruption (see optional step 1.12). Quantitative examination of DAPI-stained, fixed nuclei can be performed at this stage using a hemacytometer or similar equipment. In our experience, observation of three or more intact nuclei per field of view at 40-60x magnification is indicative of a sufficient yield, even if cellular debris is evident.

[9] Meristematic tissues do not usually require further mechanical disruption after thorough resuspension in MDBT.

[10] For a 4g input, dilute the nuclei suspension to a final volume of 50 mL with MDB.

16. Pellet nuclei by centrifugation at 2000 rcf for 15 minutes at 4ºC.
17. Discard the supernatant and resuspend nuclei in 15 mL ice cold MDB.[12]
18. Pellet nuclei by centrifugation at 2000 rcf for 15 minutes at 4ºC.
19. Resuspend nuclei in 2.2 mL ice-cold MDBT.[13]
20. Remove ~50 μL or Microscopic Check 4 "MC4" and hold at 4ºC to confirm recovery of intact nuclei and removal of cellular debris.[14]
21. Aliquot 500 μL nuclei into each of four 1.5 mL screwcap tubes.[15]
22. Flash freeze all four aliquots of isolated nuclei in liquid nitrogen for future use or long term storage at -80ºC.[16]
23. During or following these steps, examine MC1 - MC4 to confirm the quality, approximate quantity, and purity of the nuclei, preparing slides as follows.
    a. For each 50 μL aliquot, add 5 uL 10 μg/mL DAPI (~ 1μg/mL DAPI final).
    b. To each slide, add 5 uL of DAPI-stained nuclei followed by 10 uL mounting medium. Mix by stirring with the end of pipette, and seal under a glass coverslip.
    c. Examine nuclei by epifluorescence microscopy with a 40X objective lense[17].

---

[11] Dilution of nuclei-containing aspirate is necessary to decrease the density of the solution to allow for centrifugal sedimentation of nuclei.
[12] For a 4g input, dilute nuclei to 50 mL.
[13] For a 4g input, resuspend nuclei in 4 mL ice-cold MDB containing 1% Triton X-100.
[14] After this point, the protocols for a 4g and 1g preps are identical.
[15] Label tubes "1 of 4" through "4 of 4". The first tube will be used for the analytical titration, the second for the preparative digests, and the last two as extras in case needed.
[16] Flash freeze all aliquots to prevent variation between samples due to one freeze-thaw cycle.
[17] For Observation with 40X-60X lens can be used to estimate "good yields" suitable for proceeding. Here, good yields refer to the ability to readily detect nuclei when scanning around and looking through the eyepiece. For each MC, look for the following: MC1 (5μL of 11 mL)- observe quenched, fixed cells. Large tissue aggregates (of >20 cells) indicate incomplete tissue disruption and could explain low final nuclei yield or "smear" following MNase titration; MC2 (5 uL of 4 mL lysate)- observe lysis of fixed cells. Should observe cellular debris, wall fragments, etc in addition to intact, fixed nuclei, and can include nuclei in broken cell fragments; MC3 (5 uL of 4 mL homogenized lysate) - observe lysis of fixed cells. Should observe cellular debris, wall fragments, etc in addition to intact, fixed nuclei, and can still include nuclei in broken cell fragments; MC4 (5 uL of 2.2 mL purified nuclei) - observe isolated, fixed nuclei, and some residual cellular debris.

2 Analytical MNase Titration; wide-range digestions

1. Thaw 1 (tube #1 of 4) of the 500 µL nuclei aliquots at RT, gently resuspend.
2. Aliquot 60 uL into each of seven 1.5 mL screw-cap centrifuge tubes.
3. Prewarm the eight tubes of nuclei in 37ºC heat block while preparing MNase series.
4. Prepare enzyme dilutions by 2-fold serial dilution starting with 800 U/mL MNase (from -20 stock at 20,000 U/mL) in MDB and ending with 25 U/mL. Serial dilutions were made by combining 20 uL enzyme with 20 uL diluent (MDB) followed by brief vortex and pop-spin. Repeat to produce six "enzyme dilution tubes" plus one last tube for 0 U/mL (MDB alone).
5. Digest nuclei at 37ºC for 15 minutes by rapid addition and quick, thorough mixing of 6.7 µL from "enzyme dilution tubes" or MDB-only to the seven RT tubes each containing 60 µL nuclei aliquots.[18]
6. Stop reactions by rapid addition of 5µL 0.5M EGTA-NaOH (8.0).
7. Add decrosslinking ingredients: 360 µL water, 50 µL 10% SDS, 50 uL 1.5M NaCl[19], and 5 µL 20 mg/mL proteinase K. Briefly vortex to mix.
8. Incubate the 537 uL mixture at 65ºC overnight (12-16h) to reverse the formaldehyde crosslinks.

3 Purification of DNA from Preparative Digests[20]

1. Remove tubes from 65ºC incubator. Allow samples to cool to RT.
2. Phase extract the DNA with phenol (pH 8).
    a. Add ~1.1 Volume (600 µL) buffered Phenol to each tube.
    b. Vortex for 1 minute.

---

[18] MNase titrations must be performed in such a way as to minimize the amount of time between addition of reagents to multiple samples, as small variations in reaction duration can impact degree of digestion of fixed chromatin.

[19] Extra NaCl (to 140 mM extra Na) has been added at this step specifically for MOA-seq; to improve recovery (avoid denaturation) of small DNA fragments following decrosslinking.

[20] If samples are not already in screw cap tubes with air-tight gasket seals, transfer samples to these tubes. Organic solvents used in the extraction of hydrophobic proteins and nuclear debris have significant vapor pressure at room temperature and atmospheric pressure. Use of improper containers at this step may result in sample loss due to tube-lid failure. Halogenated and non-halogenated organic solvents must be disposed of according to all institutional and governmental regulations.

c. Centrifuge at top speed (~20,000 rcf) for 10 minutes at RT with slow deceleration.
d. Transfer top (aqueous) phase containing DNA to a new 1.5 mL screwcap tube. Discard the lower, organic phase into the appropriate hazardous waste.

3. Phase extract again with 1:1 phenol:chloroform (pH 8.0) as above.
4. Phase extract again with chloroform only, as above.
5. Precipitate the DNA by the addition of 0.05 Volume of 3 M sodium acetate (pH 5.2), 1 µL 25 mg/mL LPA[21], and then 2 Volumes of 100% -20ºC ethanol and briefly vortex to mix.
6. Incubate at -20ºC for at least 30 minutes.
7. Centrifuge at top speed (~ 20,000 rcf) 20,817 rcf for 15 minutes at 4ºC.
8. Discard supernatant and wash the pellet with 1 mL 70% -20ºC ethanol.
9. Centrifuge at top speed (~ 20,000 rcf) 20,817 rcf for 10 minutes at 4ºC.
10. Discard supernatant and air dry the DNA pellet.
11. Resuspend DNA in 100 µL 37ºC TER, and incubate for 1h at RT to remove RNA.
12. Repeat the Ethanol precipitation described above (steps 5-10), but omitting LPA.
13. Resuspend final dried DNA pellet in 50 µL 37ºC TE.
14. Quantify DNA by spectrophotometry.
15. Analyze 50-100 ng DNA on a 1.4% agarose/TBE gel to verify recovery and visualize quality (nucleosomal ladders) of the digests. Store purified DNA at -80ºC until further use.
16. Select digests to be pooled for library preparations. Criteria should be uniform across all samples. For this study, we pooled either two or three digests by inspecting the digest patterns from the gel, and selecting digests (see Fig. 1 and associated results text) in which fragment sizes spanned the entire size range, from mononucleosome size to the largest (undigested) size.

[21] Co-precipitation carriers such as Linear Polyacrylamide are used as an inert carrier for DNA precipitations to increase efficient recovery from low concentration samples and to increase the visibility of the resulting DNA pellet.

4 Library Preparation and Size Selection for NGS.

1. Construct NGS libraries according to manufacturer's directions. [22]
2. Assess DNA yield with Qubit fluorometric quantitation.
3. Assess fragment size distribution with an Agilent Bioanalyzer HS DNA chip.
4. Libraries were size-selected using Blue Pippin using lower (135 bp) and upper size (250 bp) cutoff values to recover maize genomic insert sizes ranging 15-130 bp.
5. Libraries are ready for final qPCR quantitation, pooling, verification of pooled quantity by qPCR and bioanalyzer, and Illumina NGS.
6. Pool indexed libraries for paired-end or single-end sequencing.

[22] For DNS-seq libraries, we use 200-500 ng DNA per library, using NEBNext® Ultra™ II DNA Library Prep (Cat. E7103S).
