# Supplementary material for: The native cistrome and sequence motif families of the maize ear
Source: PLoS Genet. 2021 Aug 12;17(8):e1009689. doi: 10.1371/journal.pgen.1009689 (PMC8360572; doi:10.1371/journal.pgen.1009689)
Supplement: S2 File — Read-normalized coverage from combined libraries aligned to B73v3 and used as input for peak segmentation with the iSeg algorithm. The bigwig file is published and available via FigShare, https://doi.org/10.6084/m9.figshare.13012529.v1. (DOC) [file pgen.1009689.s009.doc]

**Bigwig file of read-normalized MOA coverage for B73v3.** Read-normalized coverage from combined libraries aligned to B73v3 and used as input for peak segmentation with the iSeg algorithm. The bigwig file is published and available via FigShare, <https://doi.org/10.6084/m9.figshare.13012529.v1>.

DataCite:

Bass, Hank (2021): S2 File. Bigwig file of read-normalized MOA coverage for B73v3. figshare. Online resource. https://doi.org/10.6084/m9.figshare.13012529.v1
