# Supplementary material for: The native cistrome and sequence motif families of the maize ear
Source: PLoS Genet. 2021 Aug 12;17(8):e1009689. doi: 10.1371/journal.pgen.1009689 (PMC8360572; doi:10.1371/journal.pgen.1009689)
Supplement: S3 File — MFs (fragment centers, frenters) file produced using a sliding window average to enhance detection of groups of reads with shared centrally-located regions, combined from for all libraries (ABCDc) aligned to B73v3 and used as input for peak segmentation with the iSeg algorithm. The bigwig file is published and available via FigShare, https://doi.org/10.6084/m9.figshare.13012553.v1. (DOC) [file pgen.1009689.s010.doc]

**Bigwig file of MFs coverage for B73v3.** MFs (fragment centers, frenters) file produced using a sliding window average to enhance detection of groups of reads with shared centrally-located regions, combined from for all libraries (ABCDc) aligned to B73v3 and used as input for peak segmentation with the iSeg algorithm. The bigwig file is published and available via FigShare, <https://doi.org/10.6084/m9.figshare.13012553.v1>.

DateCite:

Bass, Hank (2021): S3 File. Bigwig file of MFs (frenters) coverage for B73v3. figshare. Online resource. https://doi.org/10.6084/m9.figshare.13012553.v1
