# Supplementary material for: The native cistrome and sequence motif families of the maize ear
Source: PLoS Genet. 2021 Aug 12;17(8):e1009689. doi: 10.1371/journal.pgen.1009689 (PMC8360572; doi:10.1371/journal.pgen.1009689)
Supplement: S5 File — Read-normalized coverage from combined biological and technical replicates of libraries corresponding to "B" (1-2cm) sized earshoots, aligned to B73v3 and used for MOA coverage trend plots. The bigwig file is published and available via FigShare, https://doi.org/10.6084/m9.figshare.13014143.v1. (DOC) [file pgen.1009689.s012.doc]

**Bigwig file of MOA-seq normalized coverage for 1-2cm "B" sized earshoots.** Read-normalized coverage from combined biological and technical replicates of libraries corresponding to "B" (1-2cm) sized earshoots, aligned to B73v3 and used for MOA coverage trend plots. The bigwig file is published and available via FigShare, <https://doi.org/10.6084/m9.figshare.13014143.v1>.

DataCite:

Bass, Hank (2021): S5 File. Bigwig file of MOA-seq normalized coverage for 1-2cm "B" sized earshoots. figshare. Dataset. https://doi.org/10.6084/m9.figshare.13014143.v1
