# Supplementary material for: The native cistrome and sequence motif families of the maize ear
Source: PLoS Genet. 2021 Aug 12;17(8):e1009689. doi: 10.1371/journal.pgen.1009689 (PMC8360572; doi:10.1371/journal.pgen.1009689)
Supplement: S6 File — This PDF file provides a reference document, one page per motif, listing the assigned RSAT-dyad motif name (e.g. dym01), total number of sites in B73v3, percentage found in annotated repeats, the consensus and sequence LOGOs from RSAT reports, the median TSS-relative position and motif frequency histograms around the TSSs of the filtered gene set (FGS), the local base count composition flanking the motif midpoints, and average local MOA-seq coverage centered on the motifs for all (All) motifs, or those motifs split into either not overlapping/in repeats (NR) or overlapping/in repeats (IR). (PDF) [file pgen.1009689.s013.pdf]

Motif Name	Total Number	In Repeats
dym01	2,561	9%
Consensus	rraGGGrGGAGAGAGAGAGGrrr	
LOGO	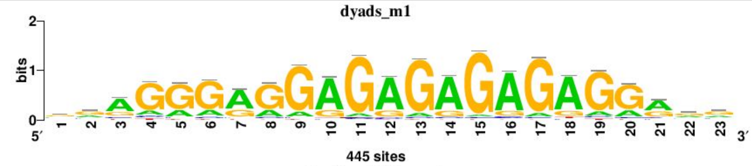	
LOGO RC	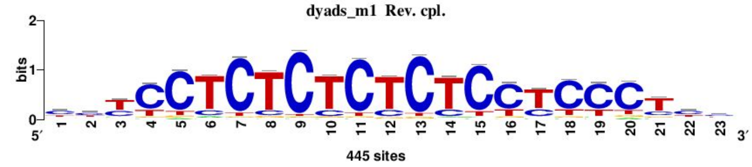	

	Frequency Distr. at TSS (FGS, B73v3)	Average Local Base Frequency (FGS, B73v3)
Median Position Relative to TSS =	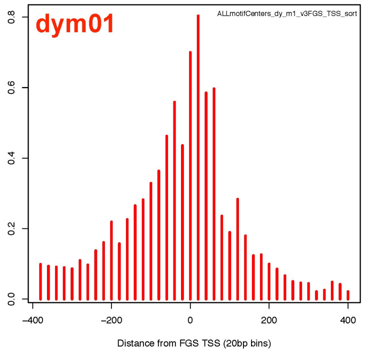	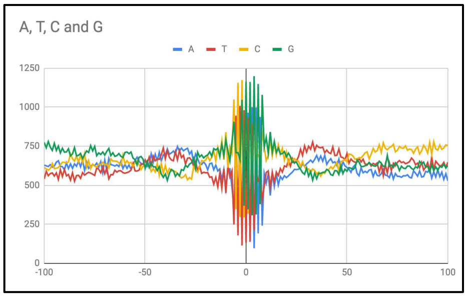
-11		

Average MOA Coverage Around Motif (RPM)

All	Not in Repeats (NR)	In Repeats (IR)
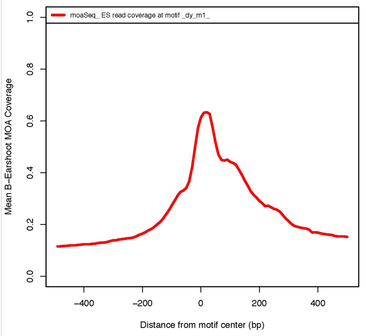	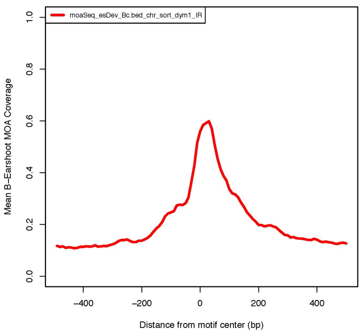	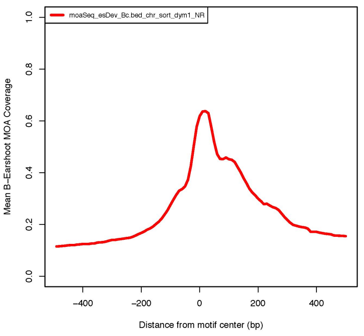

Motif Name	Total Number	In Repeats
dym02	880	17%
Consensus	cctCCCccCCCGGCCCAbCCCacc	
LOGO	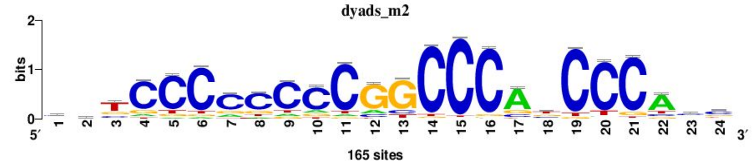	
LOGO RC	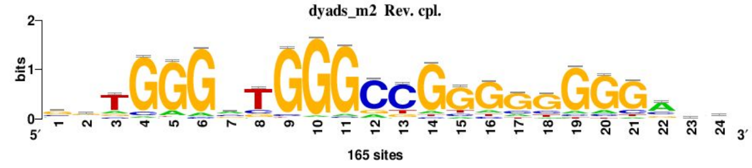	

	Frequency Distr. at TSS (FGS, B73v3)	Average Local Base Frequency (FGS, B73v3)
Median Position Relative to TSS =	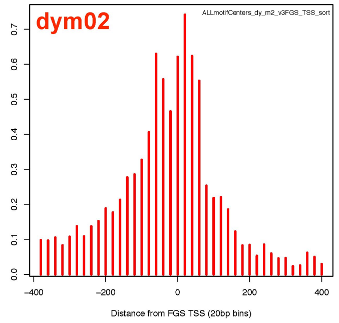	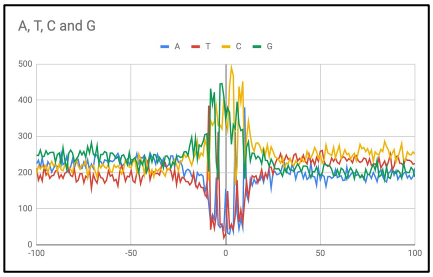
-53		

Average MOA Coverage Around Motif (RPM)		
All	Not in Repeats (NR)	In Repeats (IR)
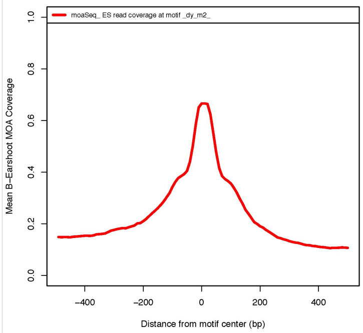	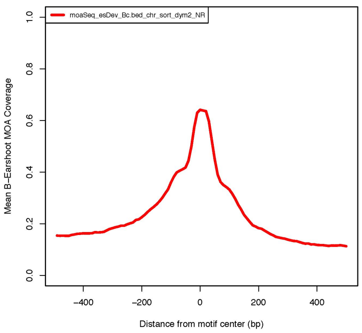	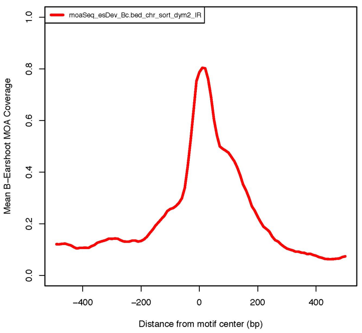

Motif Name	Total Number	In Repeats
dym03	725	15%
Consensus	cmtCCCccCCCAGCCCAaCCgCmc	

LOGO

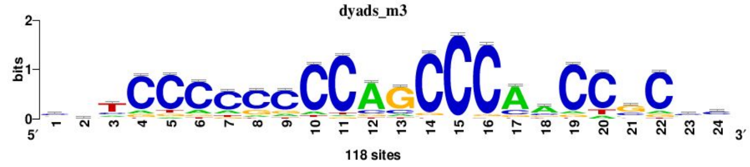

LOGO RC

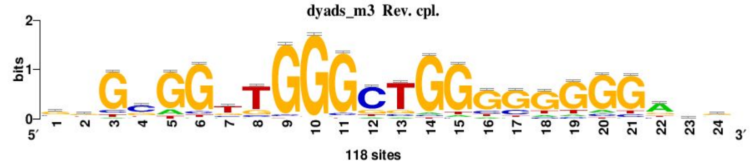

	Frequency Distr. at TSS (FGS, B73v3)	Average Local Base Frequency (FGS, B73v3)
Median Position Relative to TSS = -40		

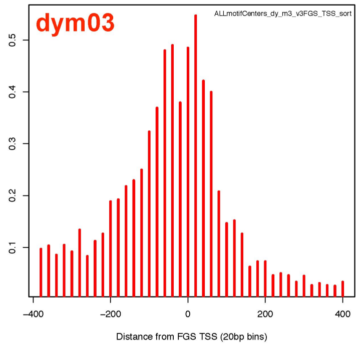

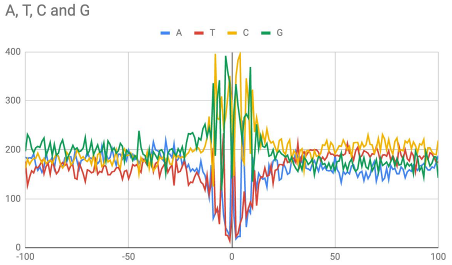

Average MOA Coverage Around Motif (RPM)

All	Not in Repeats (NR)	In Repeats (IR)

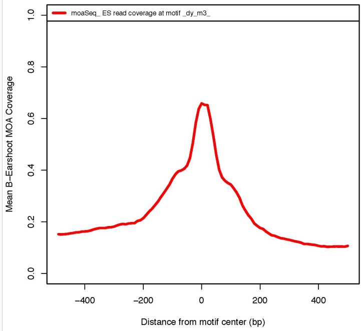

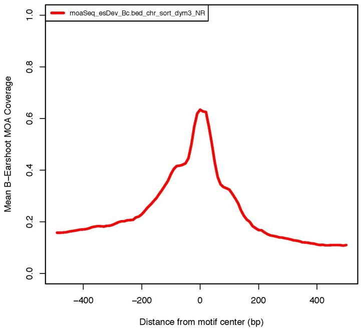

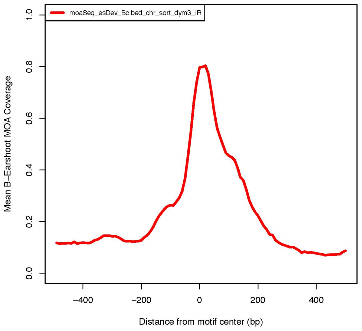

Motif Name	Total Number	In Repeats
dym04	1,937	23%
Consensus	ragAGAGArArAAAAAAAwAAmwa	
LOGO	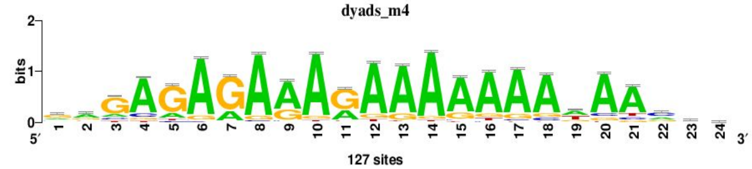	
LOGO RC	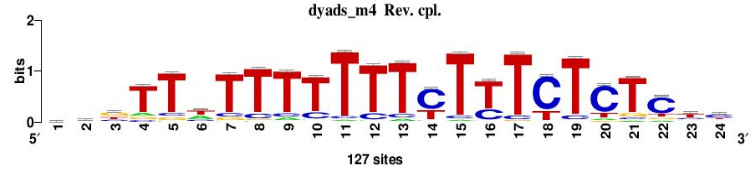	

	Frequency Distr. at TSS (FGS, B73v3)	Average Local Base Frequency (FGS, B73v3)
Median Position Relative to TSS = -59	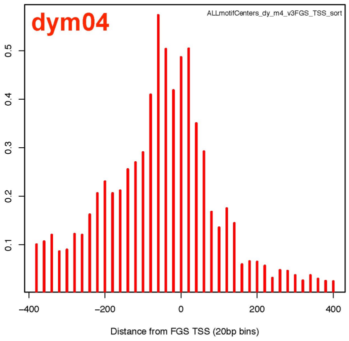	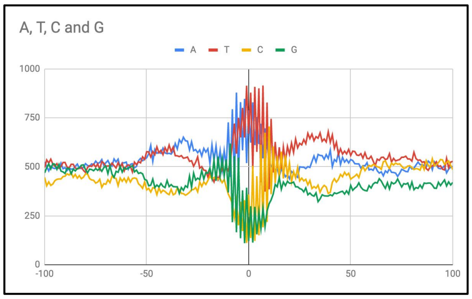

Average MOA Coverage Around Motif (RPM)

All	Not in Repeats (NR)	In Repeats (IR)
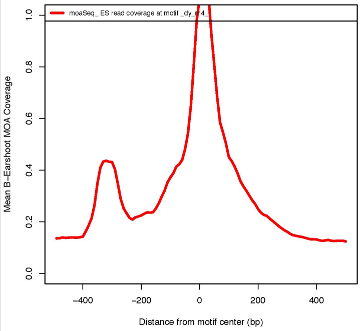	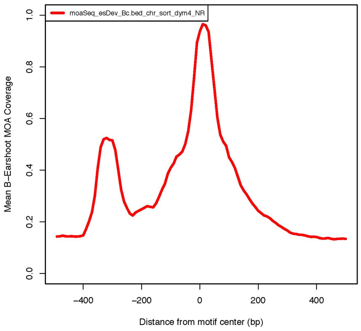	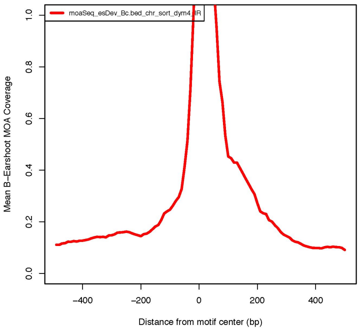

Motif Name	Total Number	In Repeats
**dym05**	**1,558**	**3%**
**Consensus**	**srGCAGCAGCAGgms**	
**LOGO**	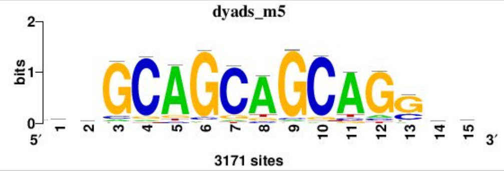	
**LOGO RC**	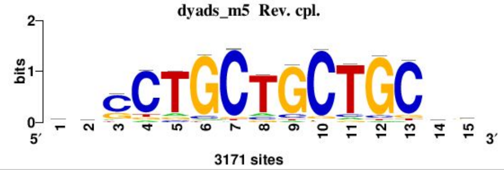	

	Frequency Distr. at TSS (FGS, B73v3)	Average Local Base Frequency (FGS, B73v3)
**Median Position Relative to TSS =**	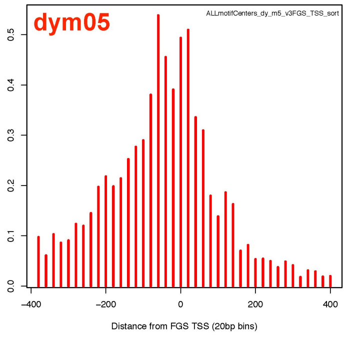	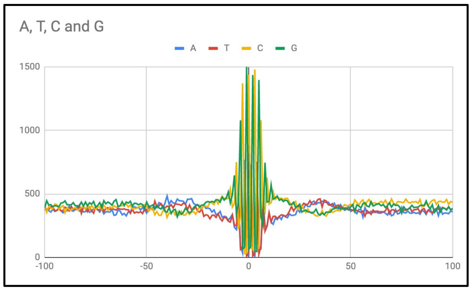
**-63**		

Average MOA Coverage Around Motif (RPM)

All	Not in Repeats (NR)	In Repeats (IR)
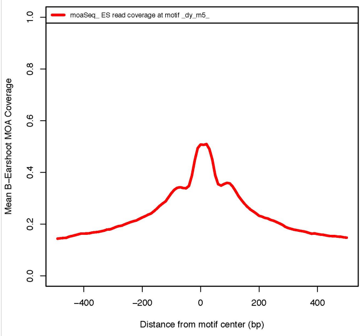	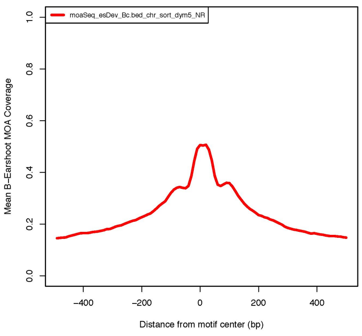	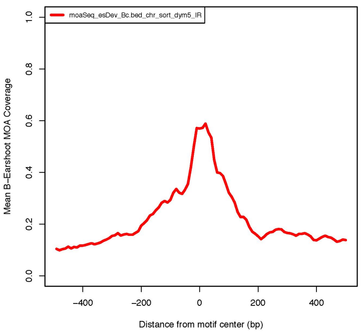

Motif Name	Total Number	In Repeats
dym06	589	8%
Consensus	ssCCCCCCGGCCCsGGCCggGGss	
LOGO	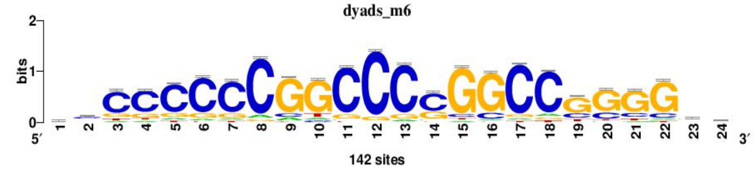	
LOGO RC	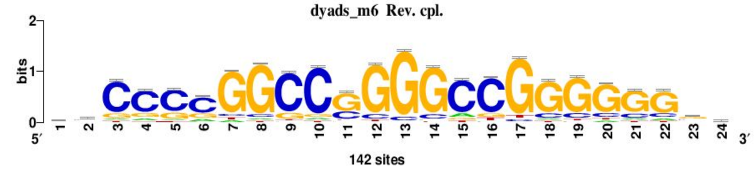	

	Frequency Distr. at TSS (FGS, B73v3)	Average Local Base Frequency (FGS, B73v3)
Median Position Relative to TSS =	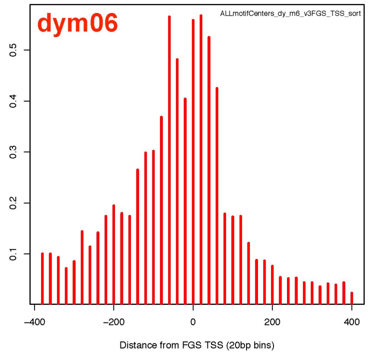	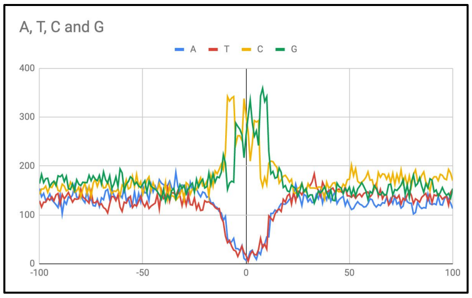
-119		

Average MOA Coverage Around Motif (RPM)

All	Not in Repeats (NR)	In Repeats (IR)
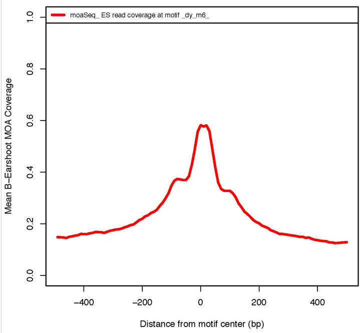	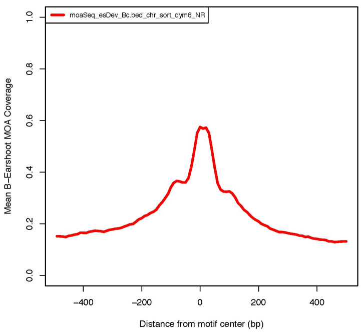	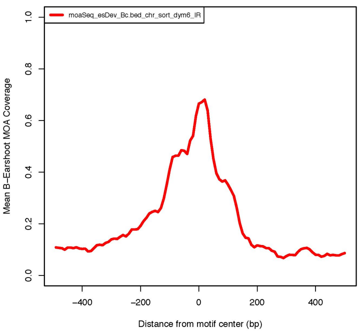

Motif Name	Total Number	In Repeats
dym07	1,014	16%
Consensus	ssCCgGCCCAGCCCAcCCCccCcc	

LOGO

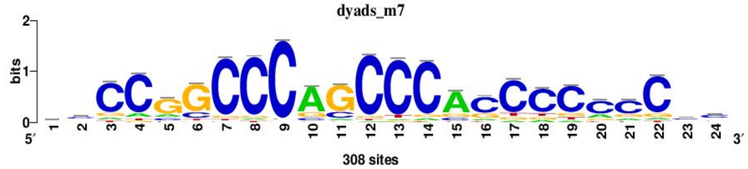

LOGO RC

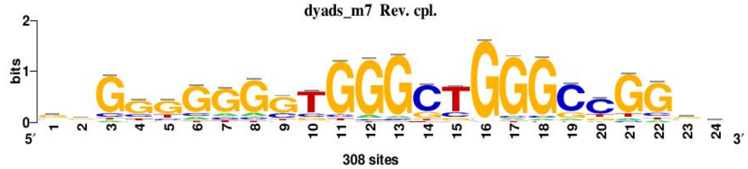

	Frequency Distr. at TSS (FGS, B73v3)	Average Local Base Frequency (FGS, B73v3)
Median Position Relative to TSS = -53	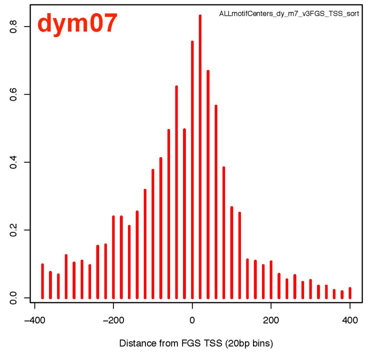	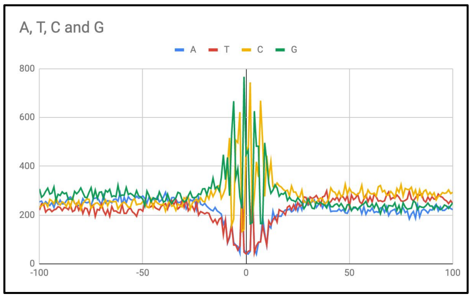

Average MOA Coverage Around Motif (RPM)

All	Not in Repeats (NR)	In Repeats (IR)
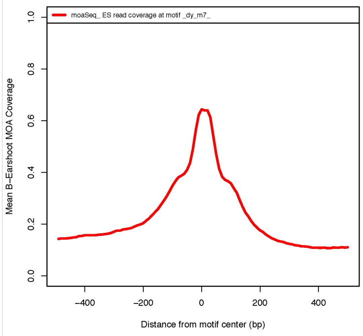	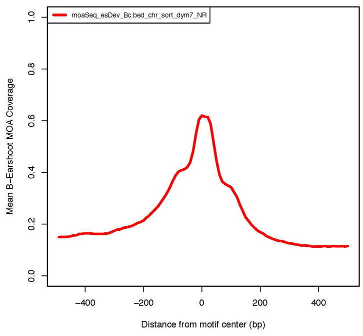	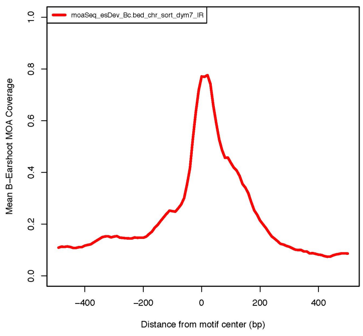

Motif Name	Total Number	In Repeats
dym08	3,615	11%
Consensus	csCACvssCACvs	
LOGO	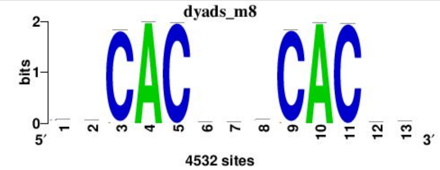	
LOGO RC	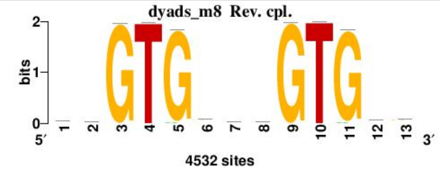	

	Frequency Distr. at TSS (FGS, B73v3)	Average Local Base Frequency (FGS, B73v3)
Median Position Relative to TSS =	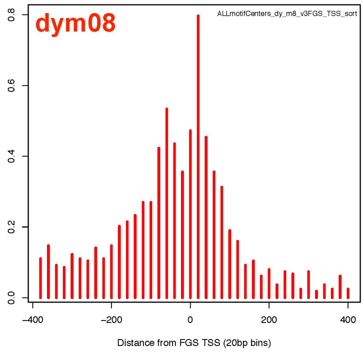	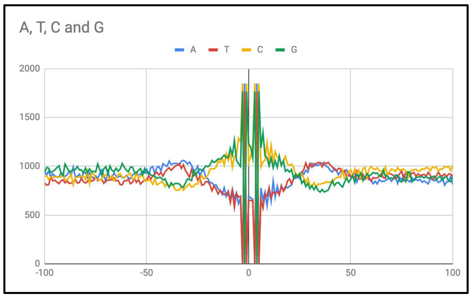
-50		

Average MOA Coverage Around Motif (RPM)

All	Not in Repeats (NR)	In Repeats (IR)
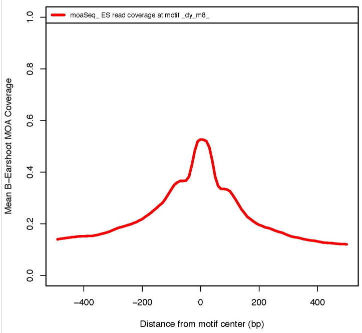	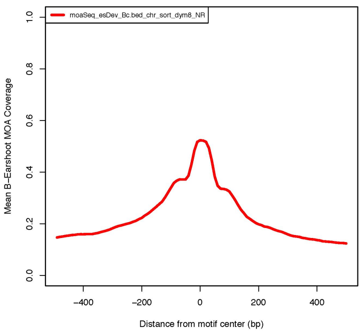	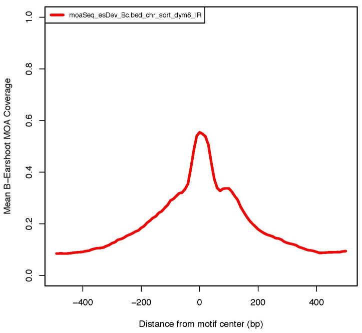

Motif Name	Total Number	In Repeats
dym09	1,780	5%
Consensus	srtgCATGCATGCAys	
LOGO	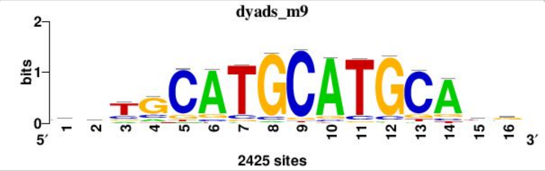	
LOGO RC	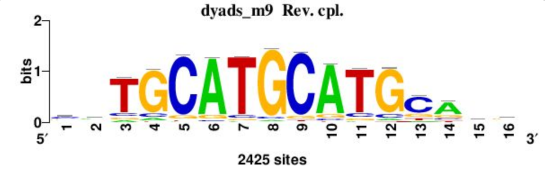	

	Frequency Distr. at TSS (FGS, B73v3)	Average Local Base Frequency (FGS, B73v3)
Meian Position Relative to TSS =	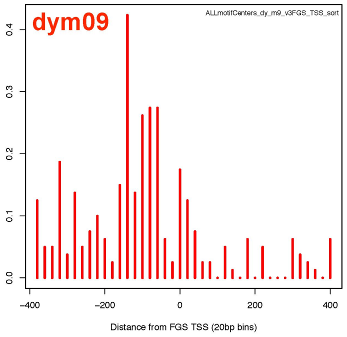	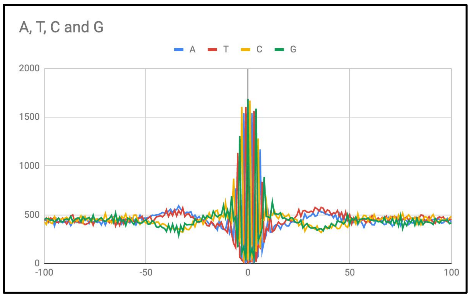
-122		

Average MOA Coverage Around Motif (RPM)

All	Not in Repeats (NR)	In Repeats (IR)
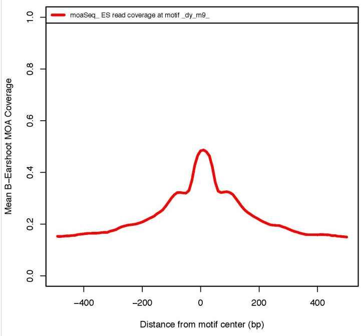	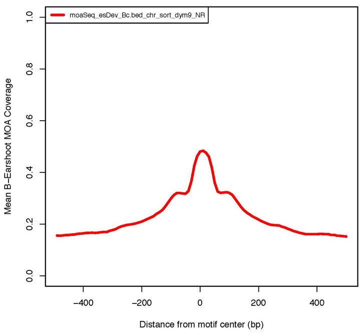	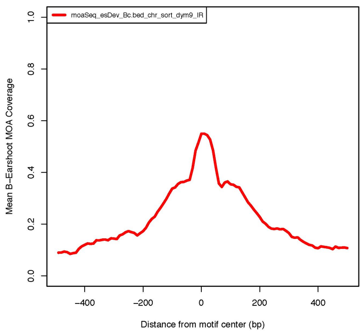

Motif Name	Total Number	In Repeats
dym10	1,866	13%
Consensus	cvCGCACGCACGCACgc	

LOGO

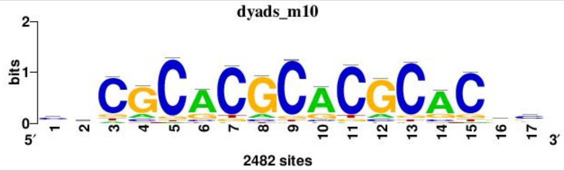

LOGO RC

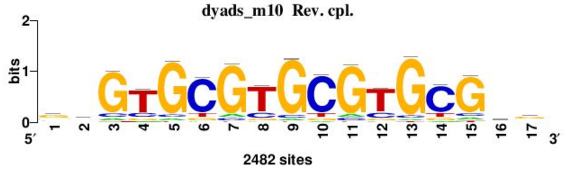

	Frequency Distr. at TSS (FGS, B73v3)	Average Local Base Frequency (FGS, B73v3)
Median Position Relative to TSS = -73	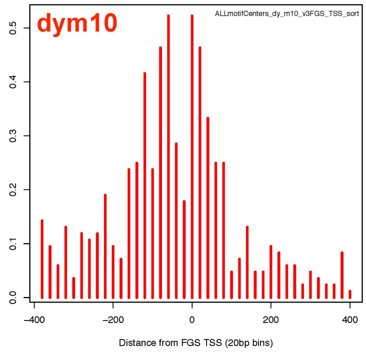	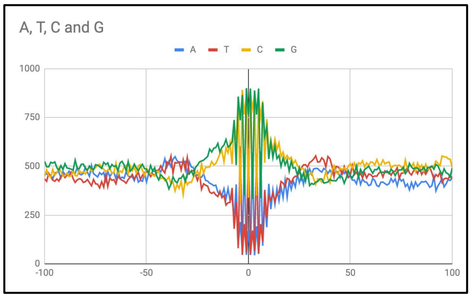

Average MOA Coverage Around Motif (RPM)

All	Not in Repeats (NR)	In Repeats (IR)
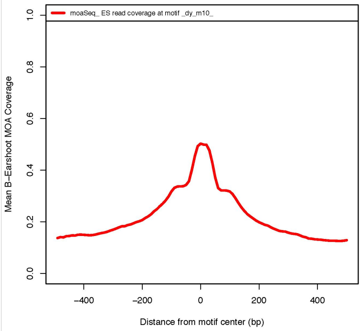	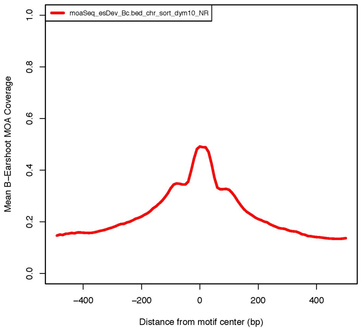	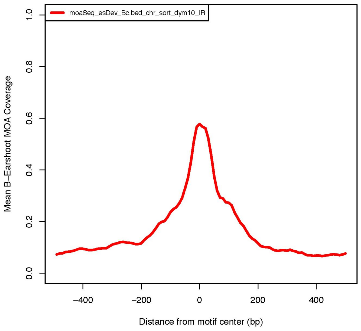

Motif Name	Total Number	In Repeats
dym11	**1,944**	**12%**
**Consensus**	**gAGAGAGAGAGArAGArArarrrr**	
**LOGO**	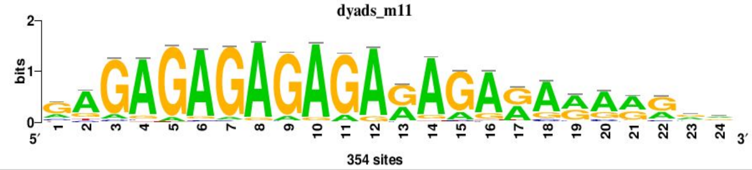	
**LOGO RC**	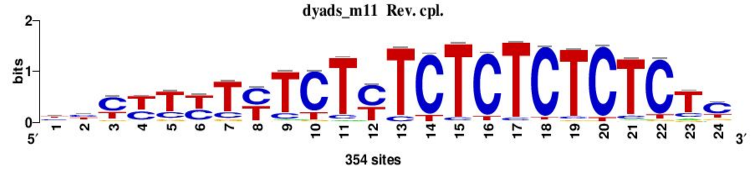	

	Frequency Distr. at TSS (FGS, B73v3)	Average Local Base Frequency (FGS, B73v3)
**Median Position Relative to TSS =** **-26**	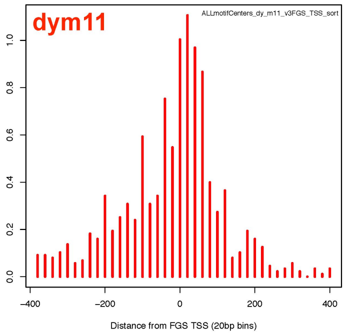	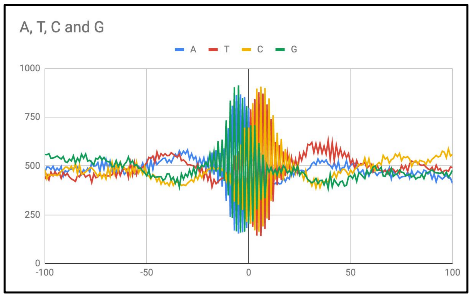

Average MOA Coverage Around Motif (RPM)

All	Not in Repeats (NR)	In Repeats (IR)
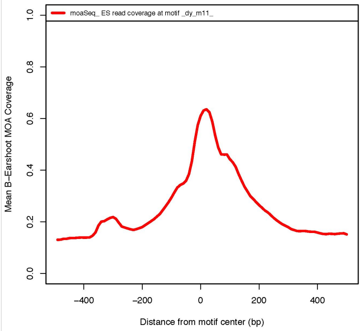	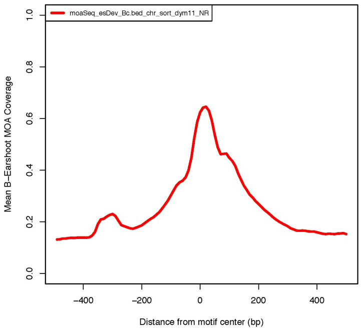	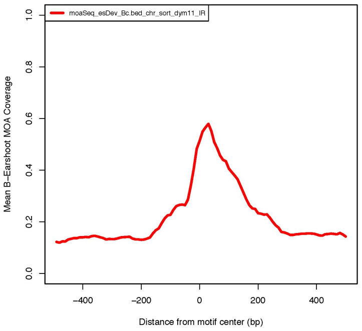

Motif Name	Total Number	In Repeats
dym12	2,153	8%
Consensus	grGrGrGAGAGrGAGAGAGArrrr	

LOGO

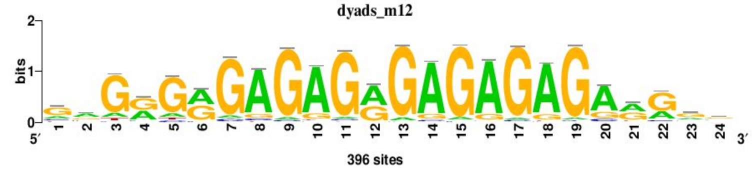

LOGO RC

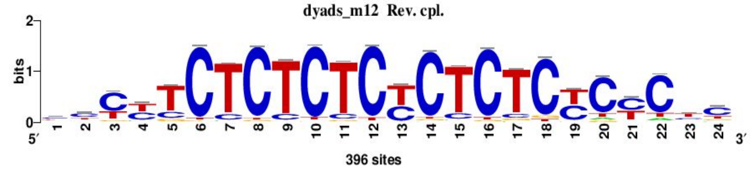

	Frequency Distr. at TSS (FGS, B73v3)	Average Local Base Frequency (FGS, B73v3)
Median Position Relative to TSS = -13	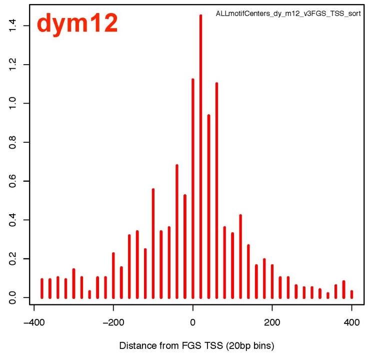	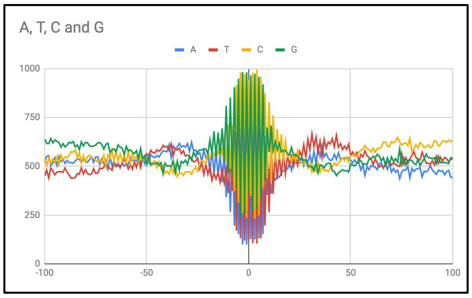

Average MOA Coverage Around Motif (RPM)

All	Not in Repeats (NR)	In Repeats (IR)
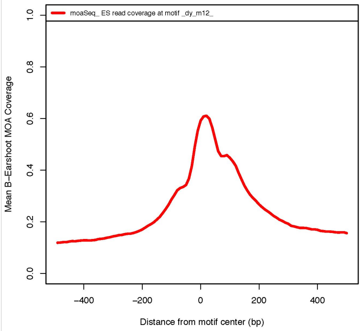	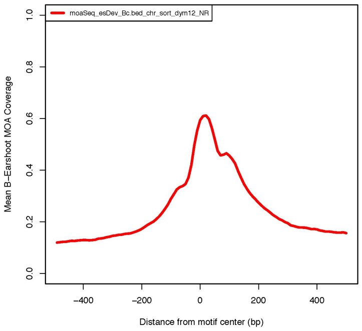	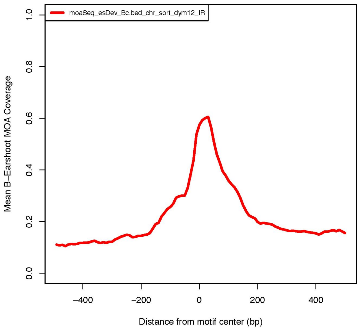

Motif Name	Total Number	In Repeats
**dym13**	**4,800**	**28%**
**Consensus**	ttcTTTTTTwTTTTwTtt	
**LOGO**	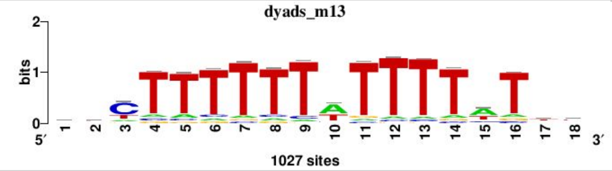	
**LOGO RC**	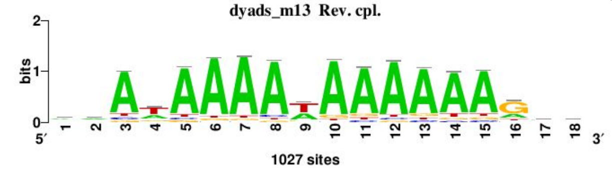	

	Frequency Distr. at TSS (FGS, B73v3)	Average Local Base Frequency (FGS, B73v3)
Median Position Relative to TSS = **-87**	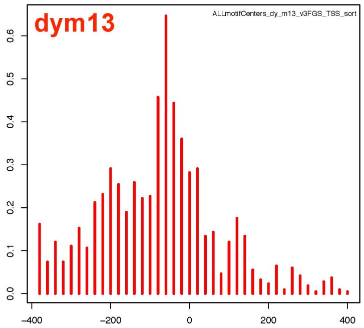	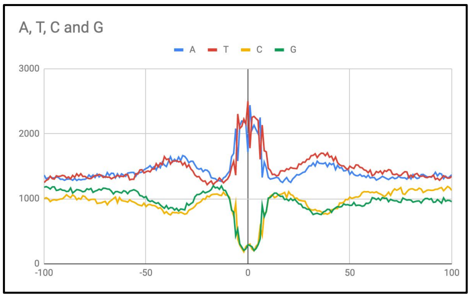

Average MOA Coverage Around Motif (RPM)

All	Not in Repeats (NR)	In Repeats (IR)
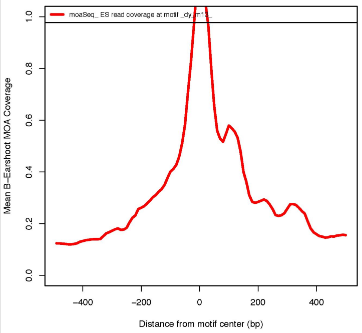	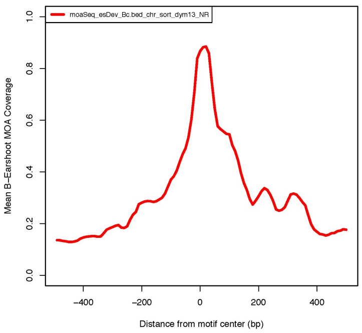	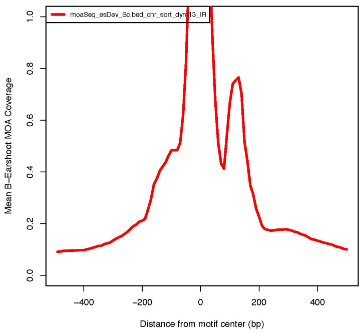

Motif Name	Total Number	In Repeats
**dym14**	**2,436**	**18%**
**Consensus**	ssCGCGCGCGCGss	
**LOGO**	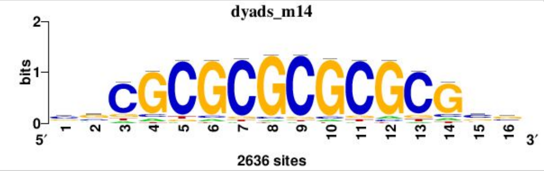	
**LOGO RC**	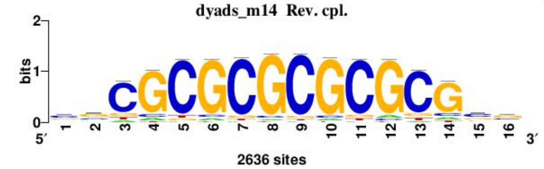	

	Frequency Distr. at TSS (FGS, B73v3)	Average Local Base Frequency (FGS, B73v3)
**Median Position Relative to TSS =**	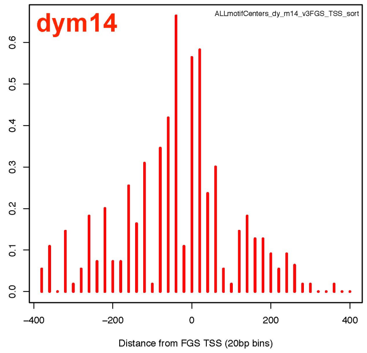	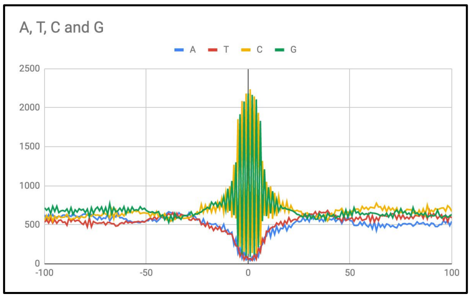
**-93**		

Average MOA Coverage Around Motif (RPM)		
**All**	**Not in Repeats (NR)**	**In Repeats (IR)**
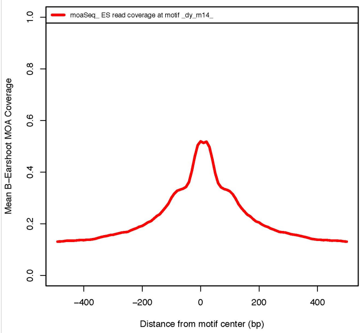	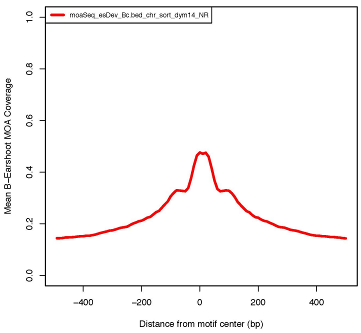	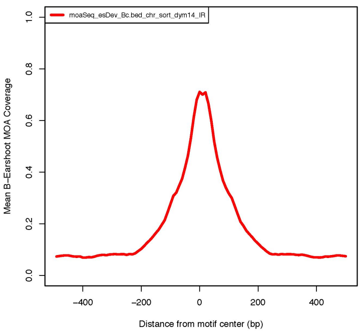

Motif Name	Total Number	In Repeats
dym15	1,629	6%
Consensus	grGGGGGGAsGGGaGGaGGaGrr	

LOGO

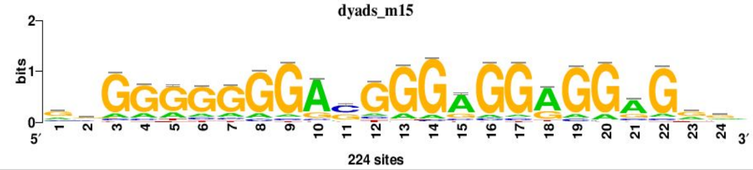

LOGO RC

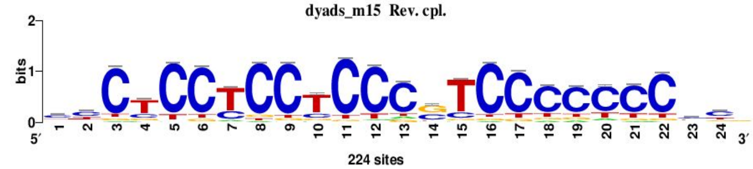

	Frequency Distr. at TSS (FGS, B73v3)	Average Local Base Frequency (FGS, B73v3)
Median Position Relative to TSS =	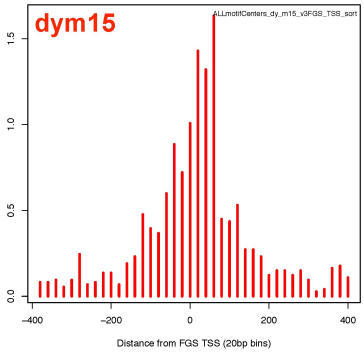	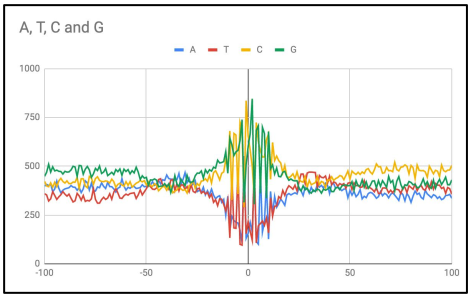
+2		

Average MOA Coverage Around Motif (RPM)

All	Not in Repeats (NR)	In Repeats (IR)
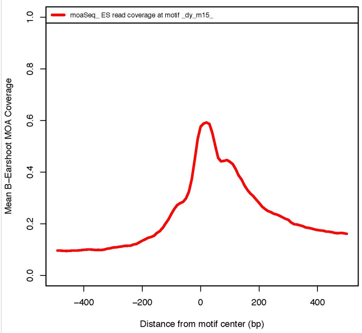	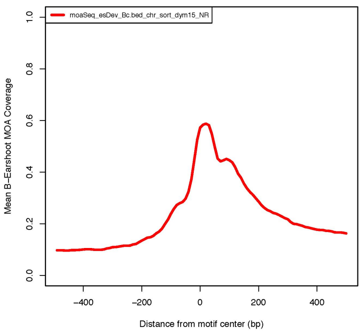	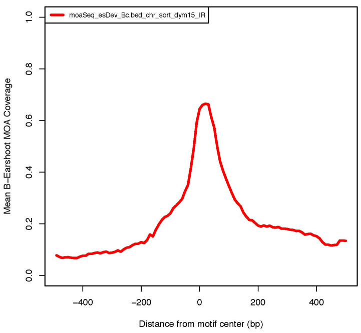

Motif Name	Total Number	In Repeats
dym16	2,198	8%
Consensus	rgrGrGrGAGrGAGAGAGAGAGrg	
LOGO	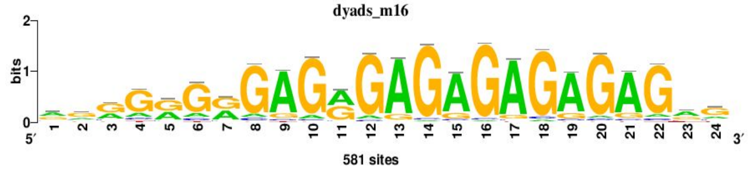	
LOGO RC	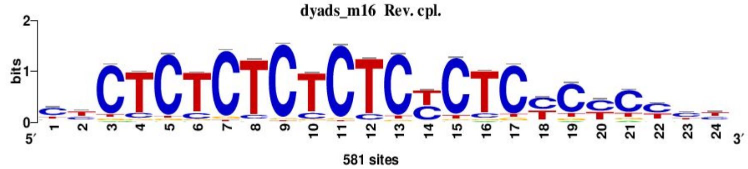	

	Frequency Distr. at TSS (FGS, B73v3)	Average Local Base Frequency (FGS, B73v3)
Median Position Relative to TSS = -15	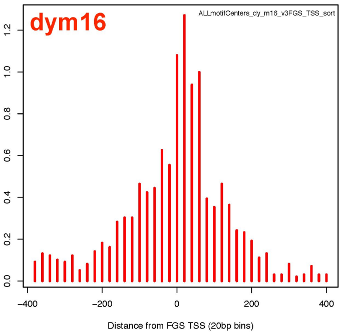	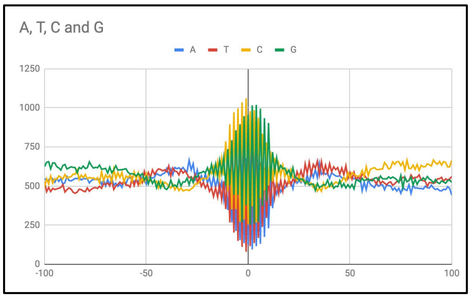

Average MOA Coverage Around Motif (RPM)

All	Not in Repeats (NR)	In Repeats (IR)
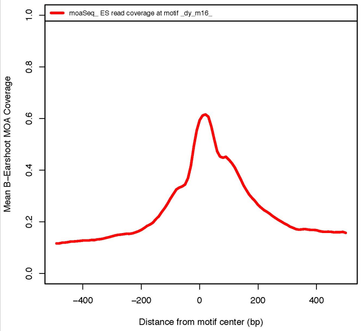	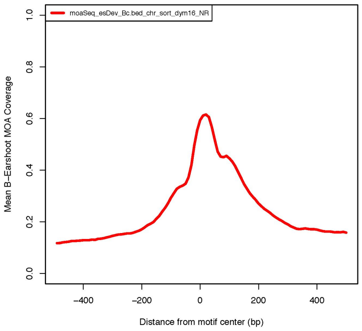	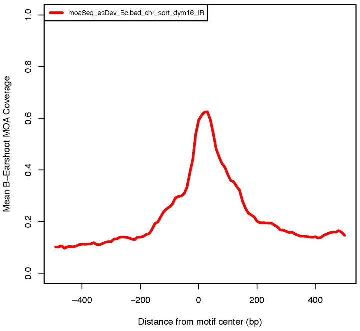

Motif Name	Total Number	In Repeats
dym17	2,058	63%
Consensus	ysGATCCAACGGyy	

LOGO

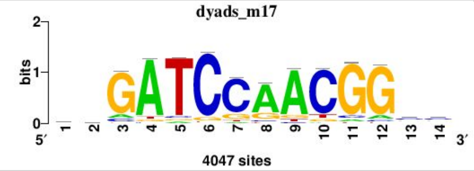

LOGO RC

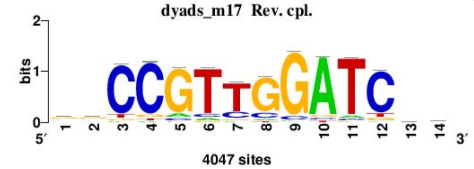

	Frequency Distr. at TSS (FGS, B73v3)	Average Local Base Frequency (FGS, B73v3)
Median Position Relative to TSS = -76		

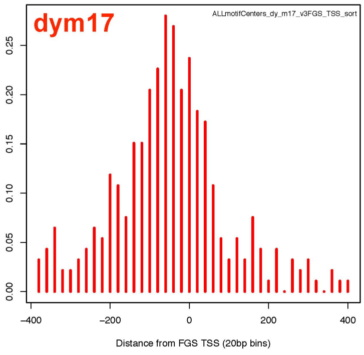

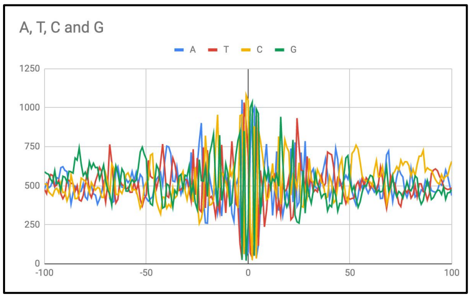

Average MOA Coverage Around Motif (RPM)

All	Not in Repeats (NR)	In Repeats (IR)

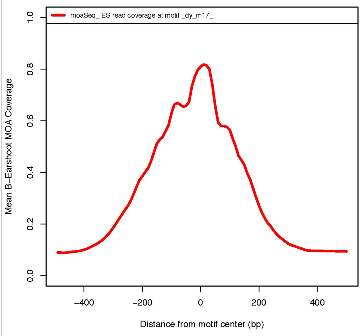

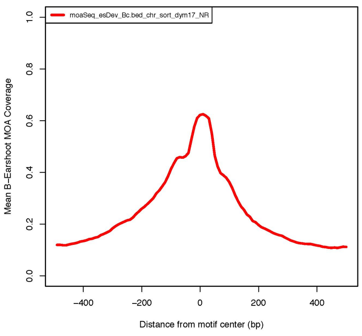

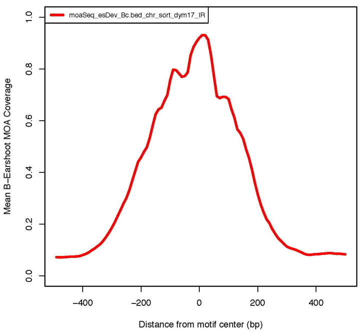

Motif Name	Total Number	In Repeats
**dym18**	**3,276**	**17%**
**Consensus**	aaCAAAAAAGra	
**LOGO**	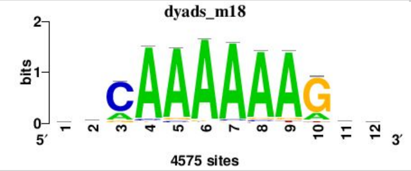	
**LOGO RC**	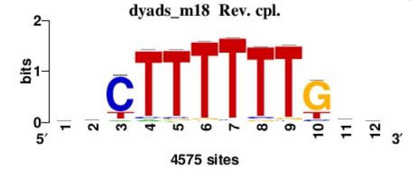	

	Frequency Distr. at TSS (FGS, B73v3)	Average Local Base Frequency (FGS, B73v3)
**Median Position Relative to TSS =** **-89**	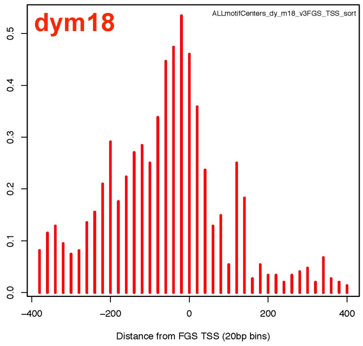	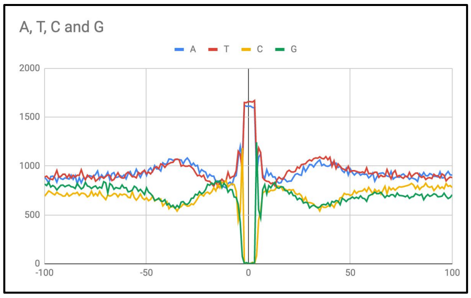

Average MOA Coverage Around Motif (RPM)

All	Not in Repeats (NR)	In Repeats (IR)
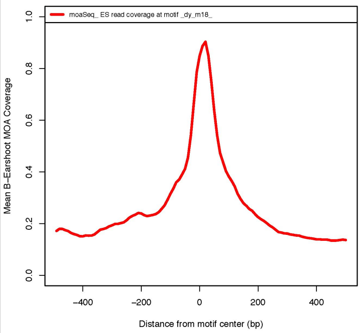	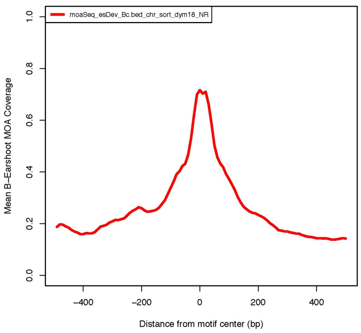	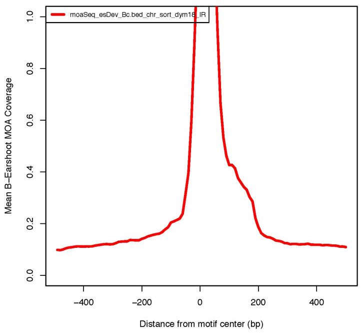

Motif Name	Total Number	In Repeats
dym19	1,977	11%
Consensus	yyCTCTyTyyyTCTCTCTCTCTct	
LOGO	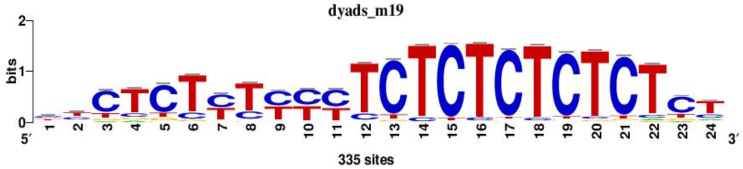	
LOGO RC	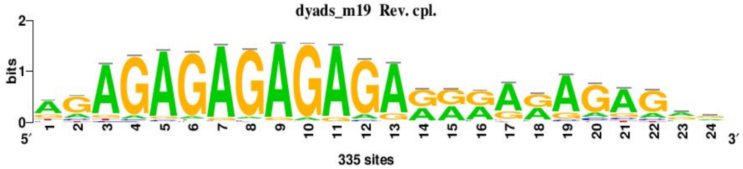	

	Frequency Distr. at TSS (FGS, B73v3)	Average Local Base Frequency (FGS, B73v3)
Median Position Relative to TSS =	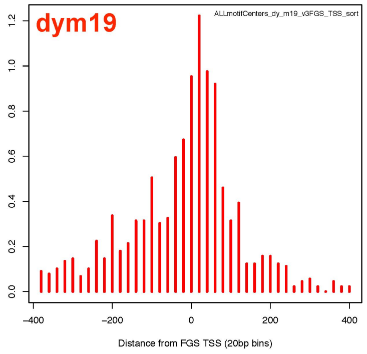	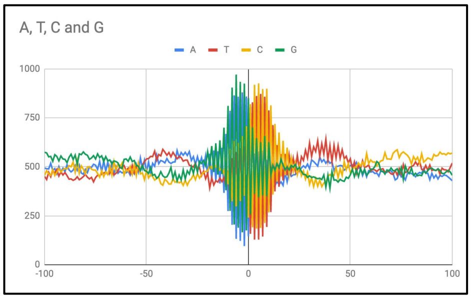
-24		

Average MOA Coverage Around Motif (RPM)

All	Not in Repeats (NR)	In Repeats (IR)
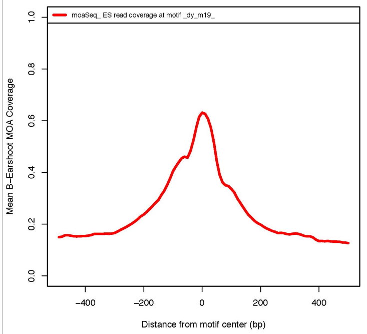	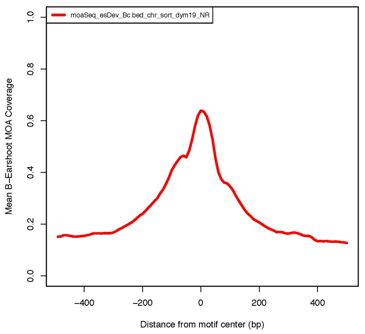	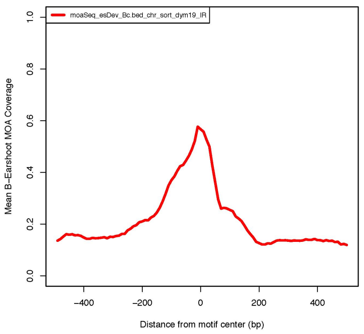

Motif Name	Total Number	In Repeats
**dym20**	**1,475**	**9%**
**Consensus**	cyCtCTCCCTCCCCmCCTCCCmcy	
**LOGO**	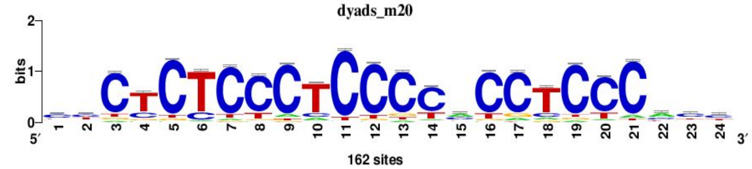	
**LOGO RC**	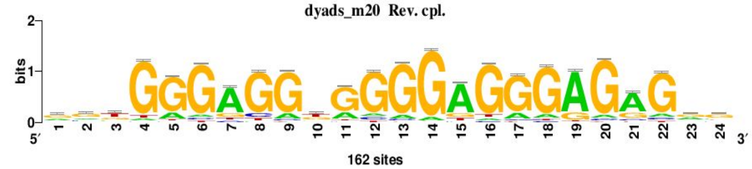	

	Frequency Distr. at TSS (FGS, B73v3)	Average Local Base Frequency (FGS, B73v3)
**Median Position Relative to TSS =**	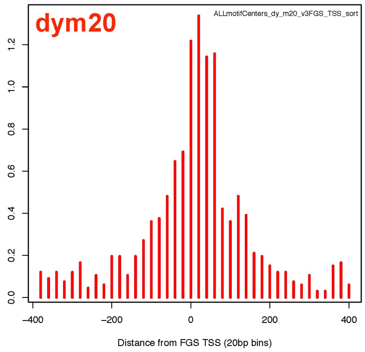	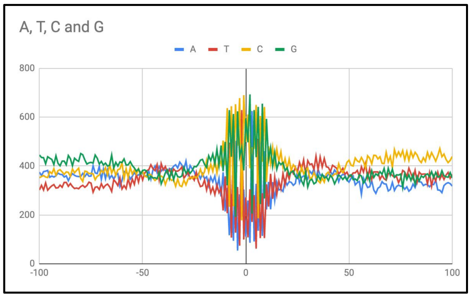
**-3**		

Average MOA Coverage Around Motif (RPM)

All	Not in Repeats (NR)	In Repeats (IR)
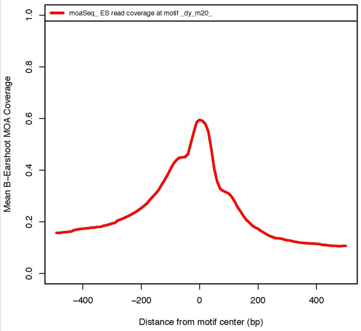	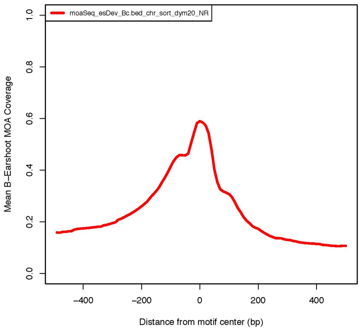	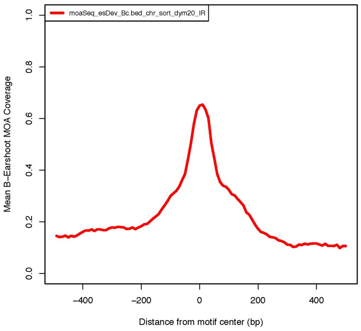

Motif Name	Total Number	In Repeats
dym21	2,417	8%
Consensus	rraGGGGAGGGAGaGrGGGAGagr	
LOGO	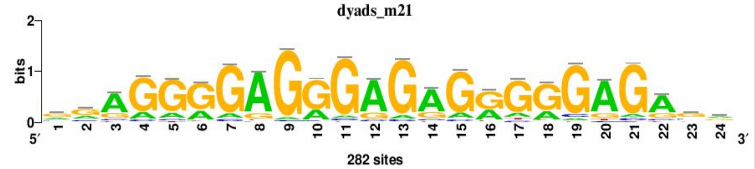	
LOGO RC	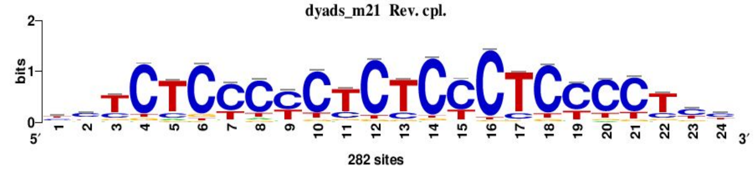	

	Frequency Distr. at TSS (FGS, B73v3)	Average Local Base Frequency (FGS, B73v3)
Median Position Relative to TSS =	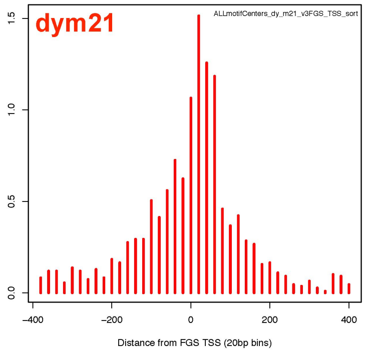	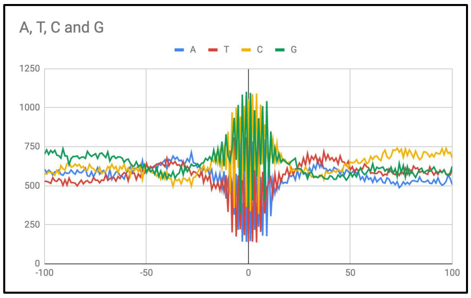
-8		

Average MOA Coverage Around Motif (RPM)

All	Not in Repeats (NR)	In Repeats (IR)
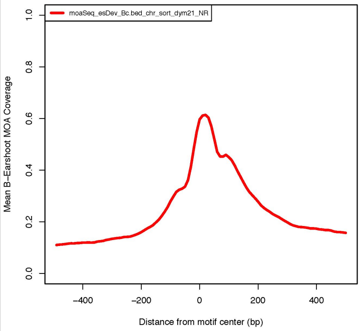	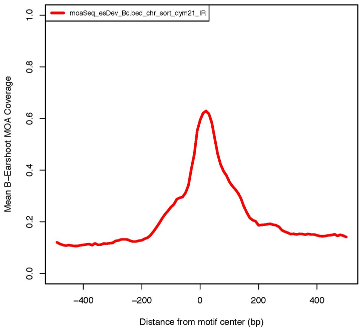	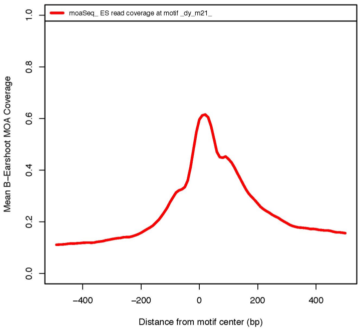

Motif Name	Total Number	In Repeats
dym22	947	12%
Consensus	cctCCCcCCCCAGCCCACgcc	
LOGO	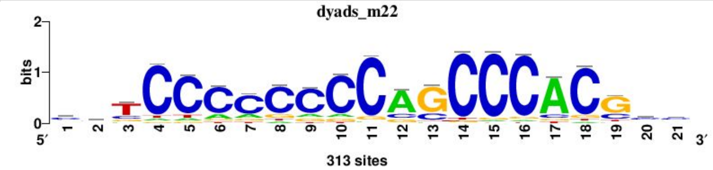	
LOGO RC	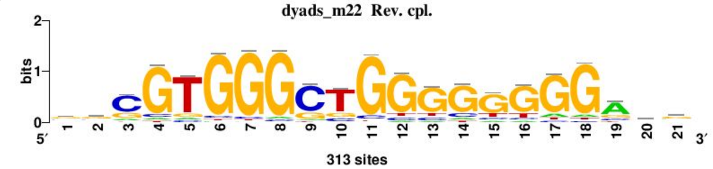	

	Frequency Distr. at TSS (FGS, B73v3)	Average Local Base Frequency (FGS, B73v3)
Median Position Relative to TSS =	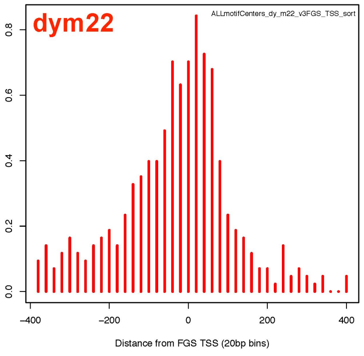	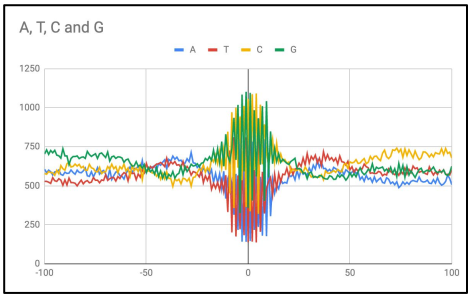
-43		

Average MOA Coverage Around Motif (RPM)

All	Not in Repeats (NR)	In Repeats (IR)
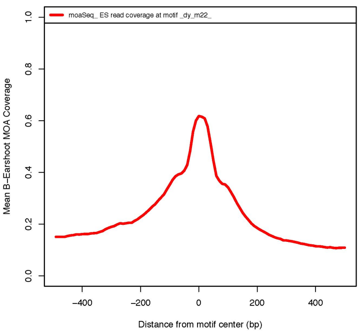	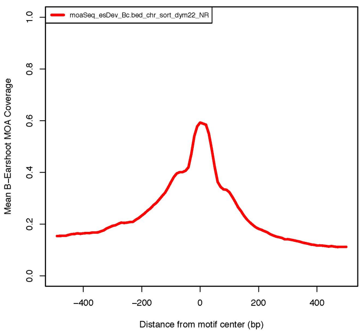	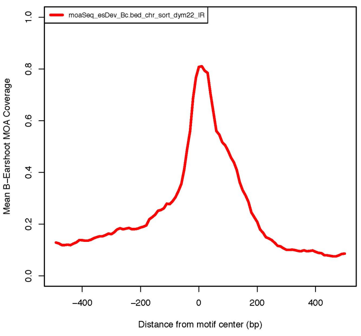

Motif Name	Total Number	In Repeats
dym23	**2,104**	**29%**
**Consensus**	**wtATTATTTTcTTcyt**	
**LOGO**	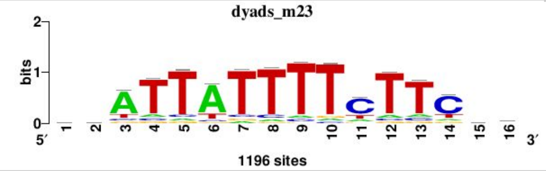	
**LOGO RC**	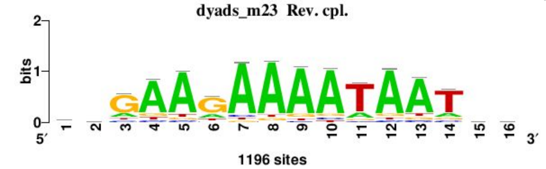	

	Frequency Distr. at TSS (FGS, B73v3)	Average Local Base Frequency (FGS, B73v3)
**Median Position Relative to TSS =**	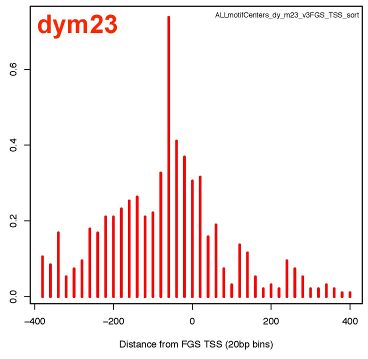	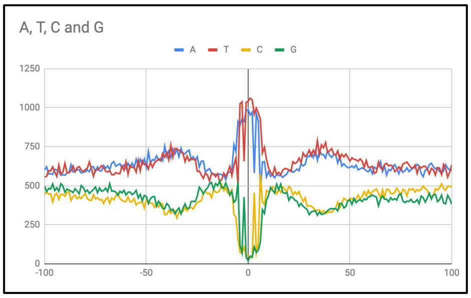
**-78**		

Average MOA Coverage Around Motif (RPM)

All	Not in Repeats (NR)	In Repeats (IR)
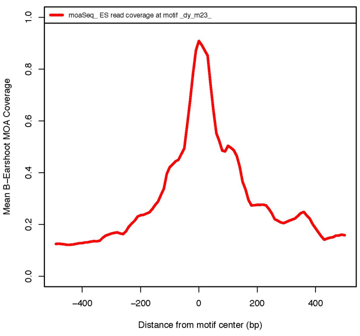	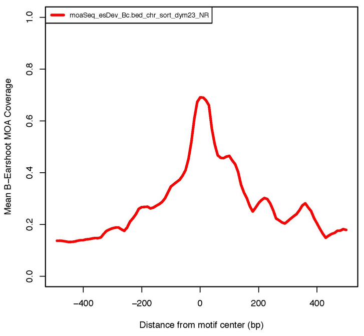	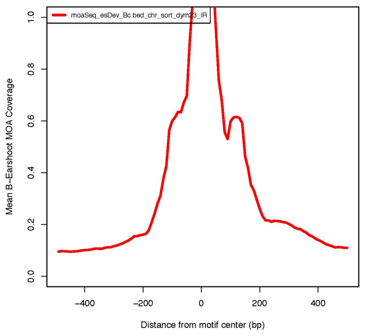

Motif Name	Total Number	In Repeats
**dym24**	**1,912**	**6%**
**Consensus**	ggrGGaGGaGGGGAGGGGGGaGgr	

LOGO

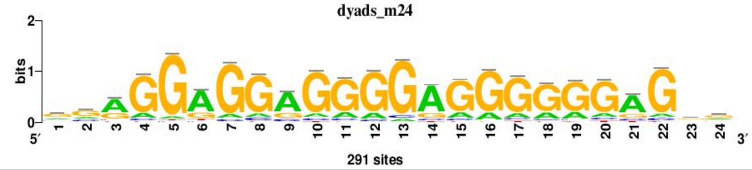

LOGO RC

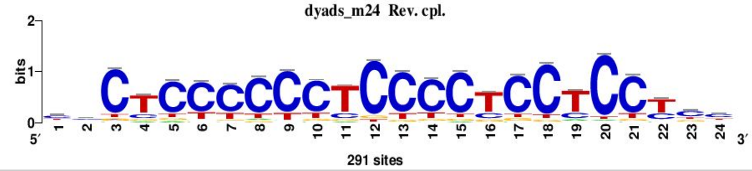

	Frequency Distr. at TSS (FGS, B73v3)	Average Local Base Frequency (FGS, B73v3)
Median Position Relative to TSS = **+2**	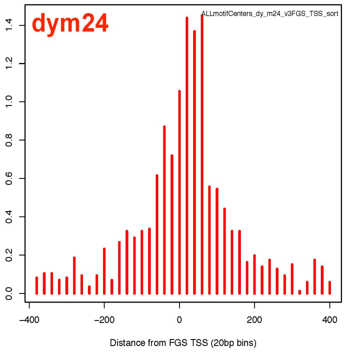	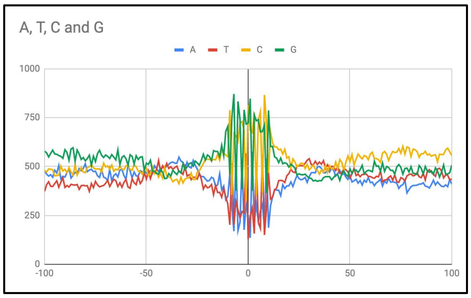

Average MOA Coverage Around Motif (RPM)

All	Not in Repeats (NR)	In Repeats (IR)
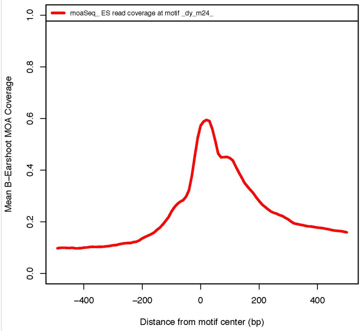	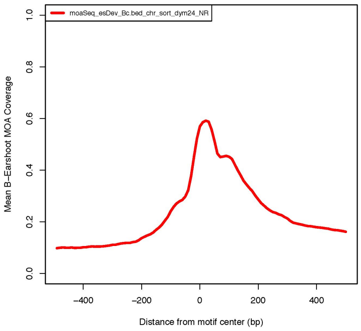	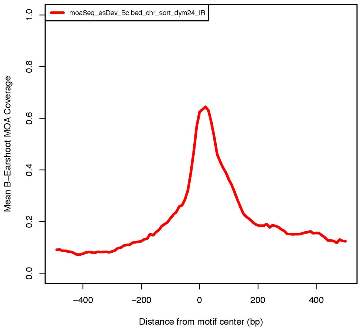

Motif Name	Total Number	In Repeats
**dym25**	**1,081**	**18%**
**Consensus**	cctCCCccCCCAGCCCAgcc	
**LOGO**	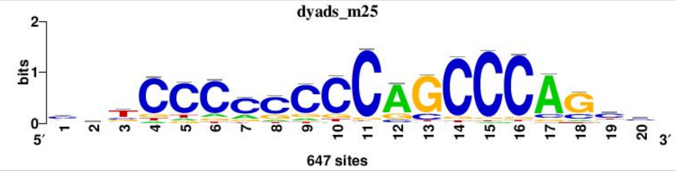	
**LOGO RC**	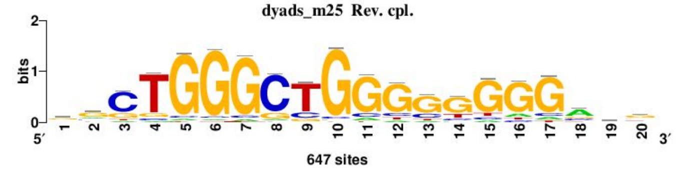	

	Frequency Distr. at TSS (FGS, B73v3)	Average Local Base Frequency (FGS, B73v3)
**Median Position Relative to TSS =** **-60**	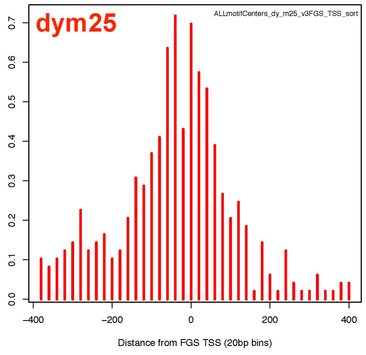	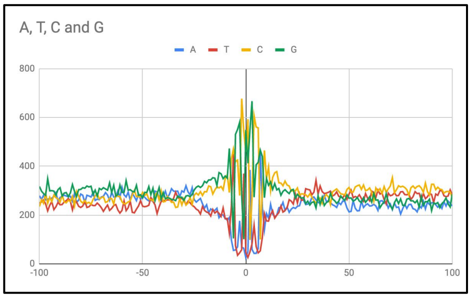

Average MOA Coverage Around Motif (RPM)

All	Not in Repeats (NR)	In Repeats (IR)
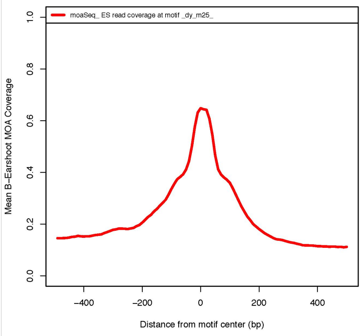	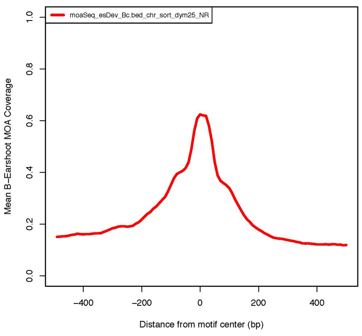	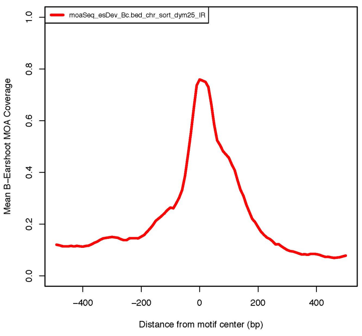

Motif Name	Total Number	In Repeats
dym26	1,920	39%
Consensus	wwATTATTTTgww	
LOGO	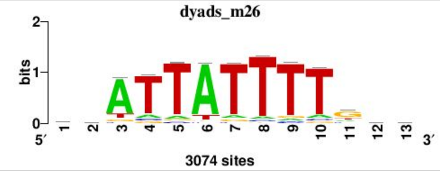	
LOGO RC	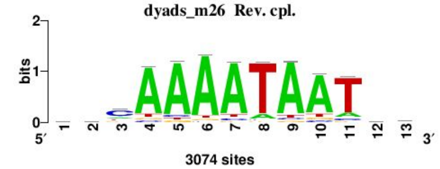	

	Frequency Distr. at TSS (FGS, B73v3)	Average Local Base Frequency (FGS, B73v3)
Median Position Relative to TSS =	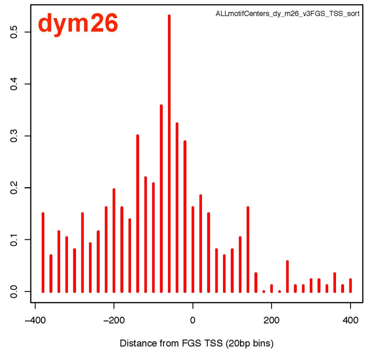	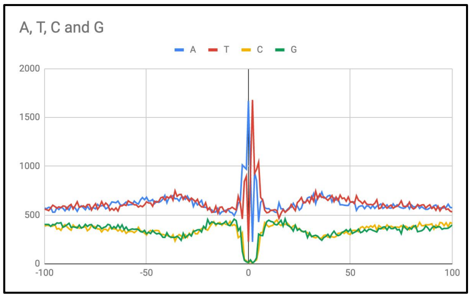
-73		

Average MOA Coverage Around Motif (RPM)

All	Not in Repeats (NR)	In Repeats (IR)
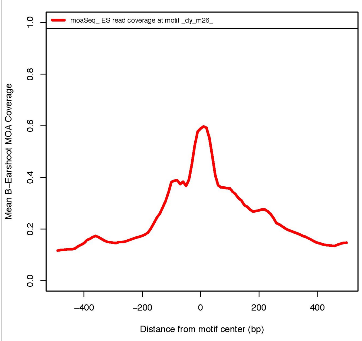	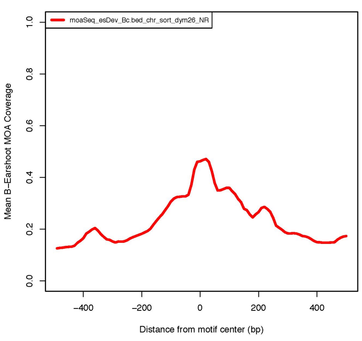	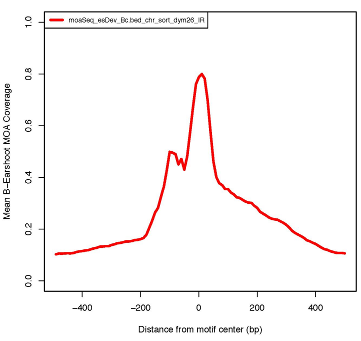

Motif Name	Total Number	In Repeats
dym27	3,266	11%
Consensus	scCCAsscCCAss	
LOGO	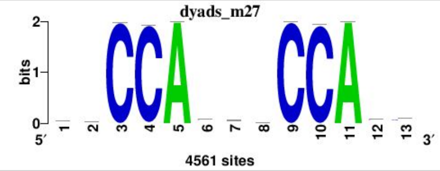	
LOGO RC	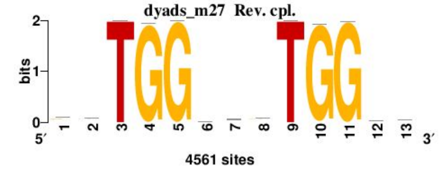	

	Frequency Distr. at TSS (FGS, B73v3)	Average Local Base Frequency (FGS, B73v3)
Median Position Relative to TSS =	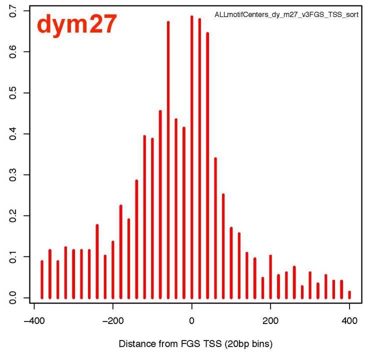	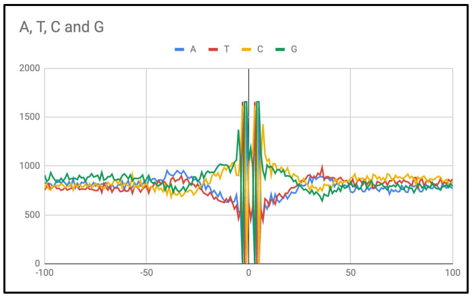
-63		

Average MOA Coverage Around Motif (RPM)		
All	Not in Repeats (NR)	In Repeats (IR)
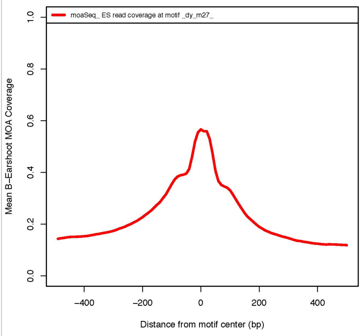	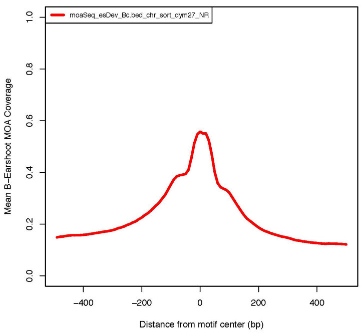	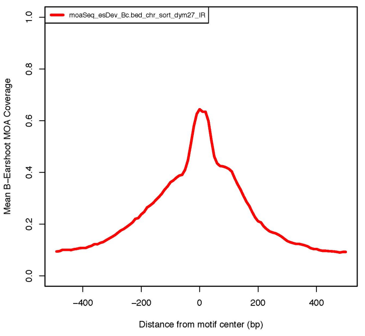

Motif Name	Total Number	In Repeats
**dym28**	**2,759**	**24%**
**Consensus**	rrrAArAAAAAArAAAArAAwra	
**LOGO**	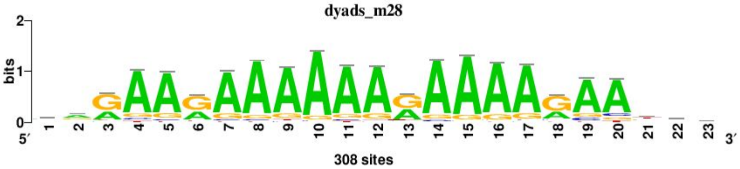	
**LOGO RC**	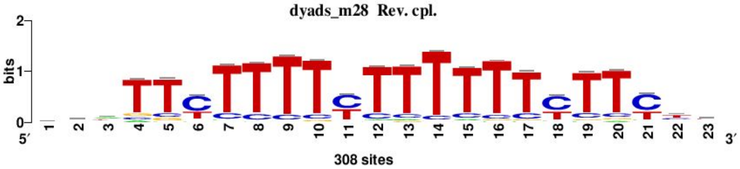	

	Frequency Distr. at TSS (FGS, B73v3)	Average Local Base Frequency (FGS, B73v3)
**Median Position Relative to TSS =** **-67**	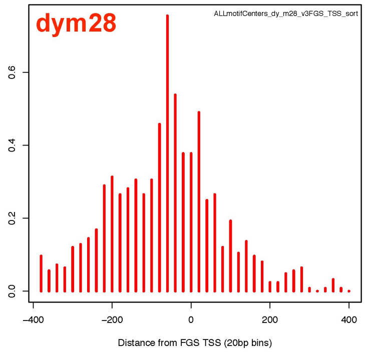	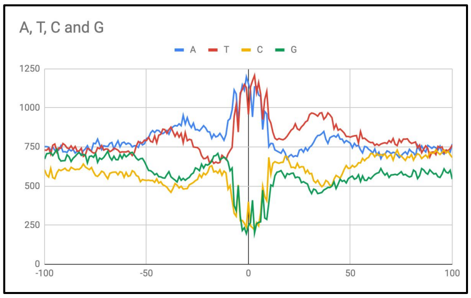

Average MOA Coverage Around Motif (RPM)

All	Not in Repeats (NR)	In Repeats (IR)
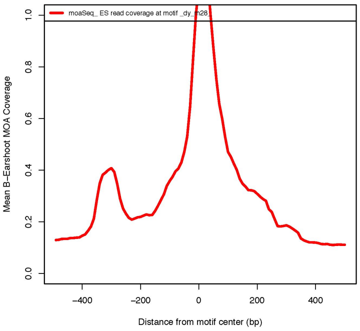	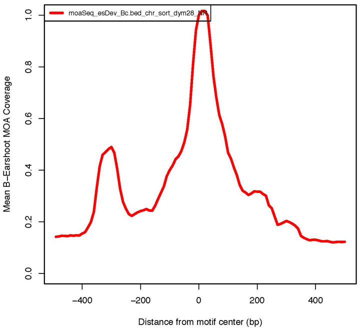	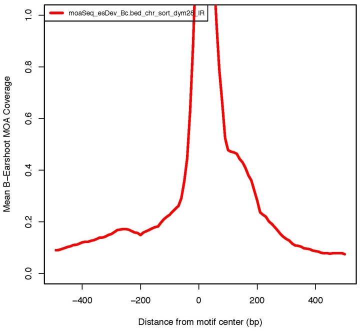

Motif Name	Total Number	In Repeats
dym29	2,049	37%
Consensus	wwAtAAATAATww	
LOGO	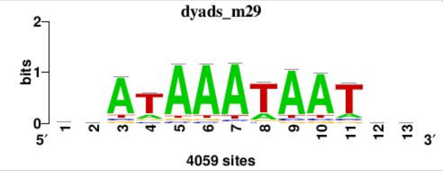	
LOGO RC	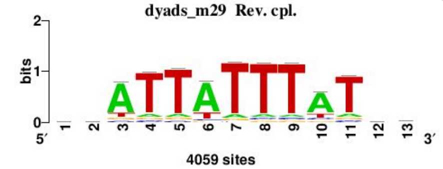	

	Frequency Distr. at TSS (FGS, B73v3)	Average Local Base Frequency (FGS, B73v3)
Median Position Relative to TSS = -74	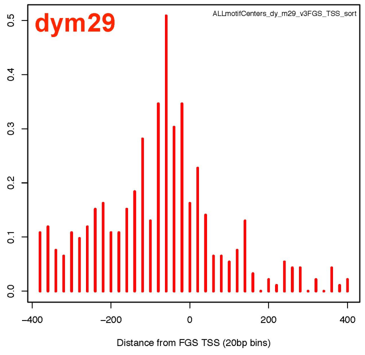	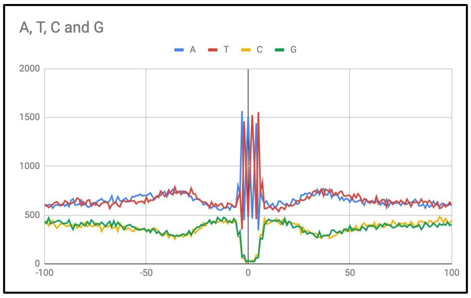

Average MOA Coverage Around Motif (RPM)

All	Not in Repeats (NR)	In Repeats (IR)
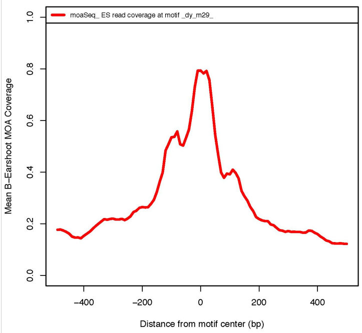	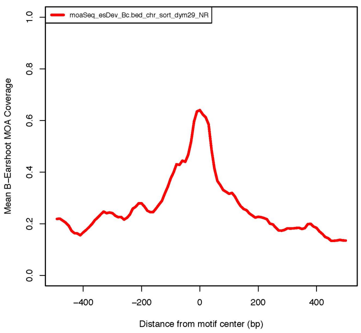	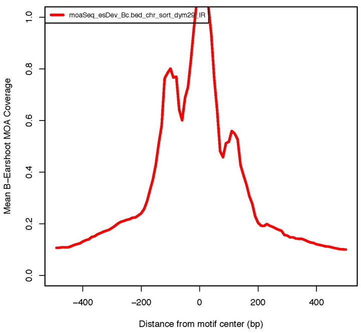

Motif Name	Total Number	In Repeats
dym30	2,369	11%
Consensus	ccACCACCas	
LOGO	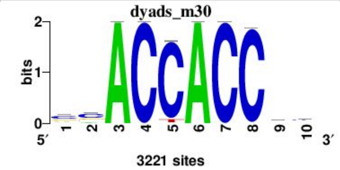	
LOGO RC	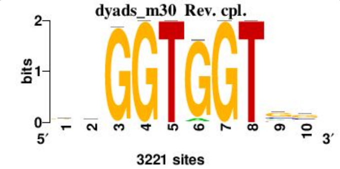	

	Frequency Distr. at TSS (FGS, B73v3)	Average Local Base Frequency (FGS, B73v3)
Median Position Relative to TSS = -51	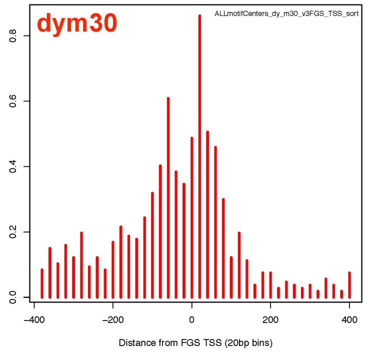	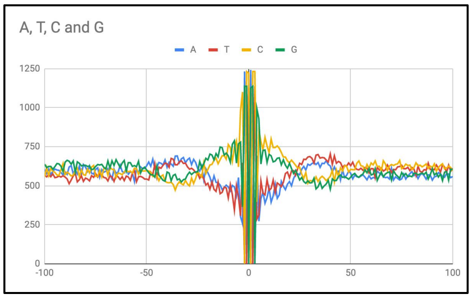

Average MOA Coverage Around Motif (RPM)

All	Not in Repeats (NR)	In Repeats (IR)
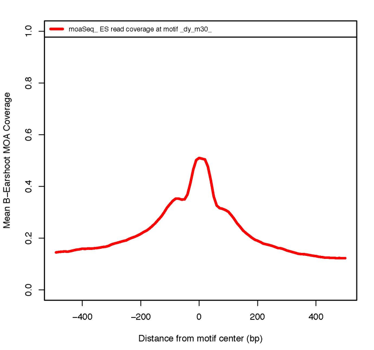	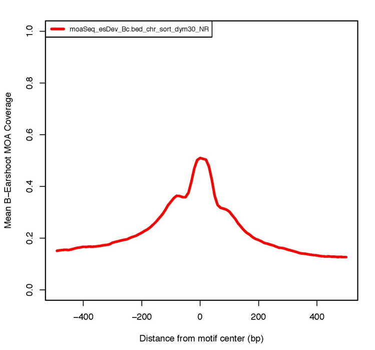	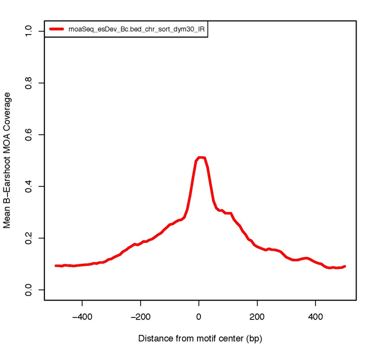

Motif Name	Total Number	In Repeats
dym31	1,788	6%
Consensus	syAGCTAGCTrs	
LOGO	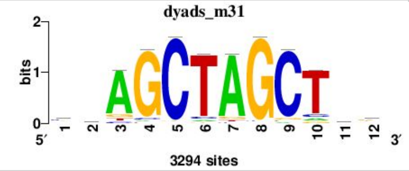	
LOGO RC	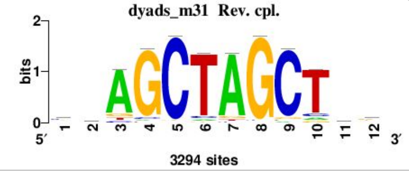	

	Frequency Distr. at TSS (FGS, B73v3)	Average Local Base Frequency (FGS, B73v3)
Median Position Relative to TSS = -188	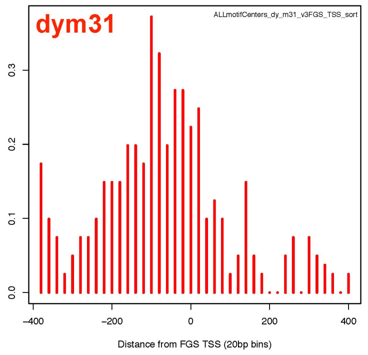	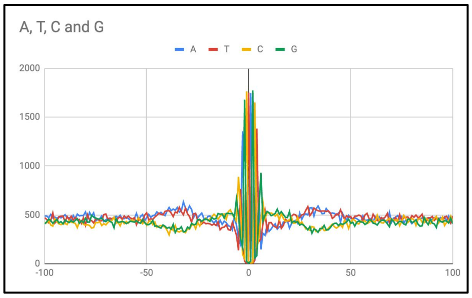

Average MOA Coverage Around Motif (RPM)

All	Not in Repeats (NR)	In Repeats (IR)
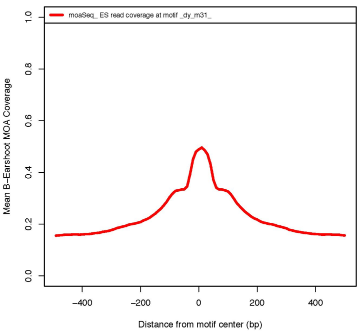	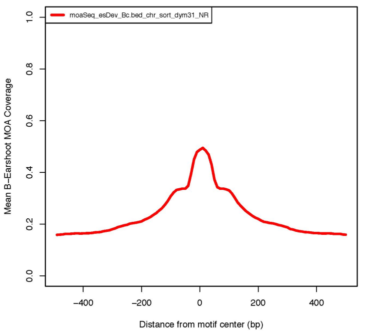	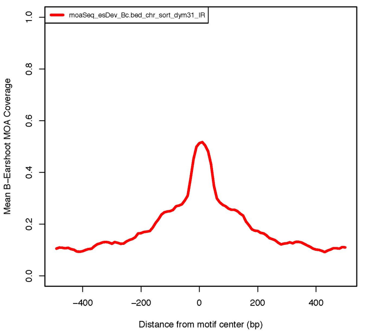

Motif Name	Total Number	In Repeats
dym32	573	17%
Consensus	sstGGCCCAtCCCCCCcgCCass	
LOGO	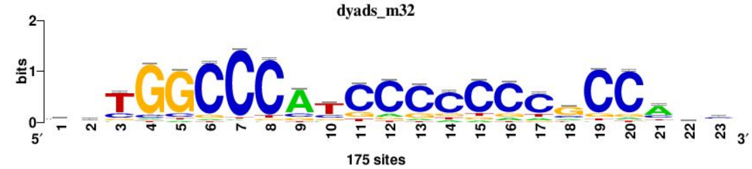	
LOGO RC	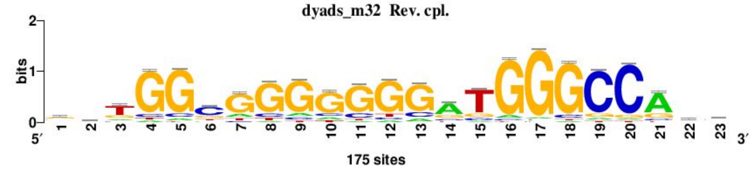	

	Frequency Distr. at TSS (FGS, B73v3)	Average Local Base Frequency (FGS, B73v3)
Median Position Relative to TSS = -80	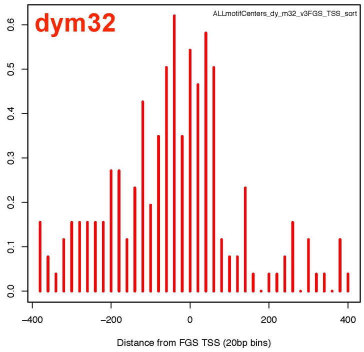	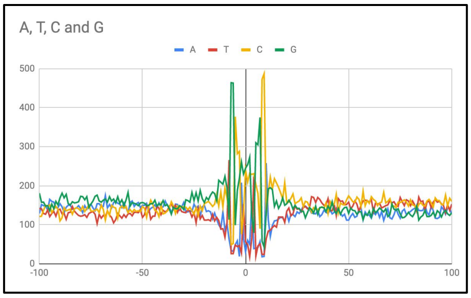

Average MOA Coverage Around Motif (RPM)

All	Not in Repeats (NR)	In Repeats (IR)
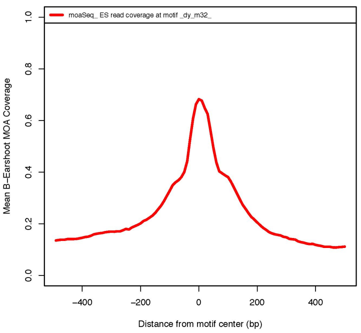	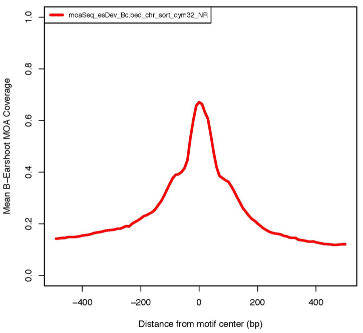	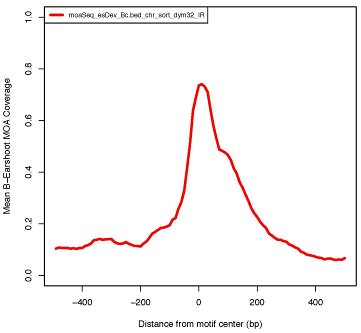

Motif Name	Total Number	In Repeats
dym33	318	20%
Consensus	ghGGGGGGaTGGGCCCAkCCCms	
LOGO	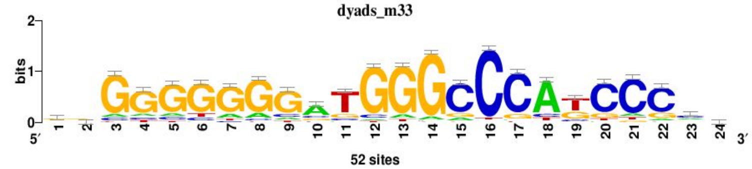	
LOGO RC	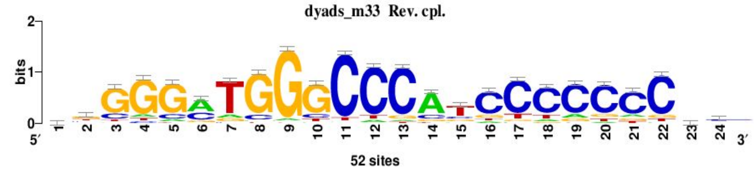	

	Frequency Distr. at TSS (FGS, B73v3)	Average Local Base Frequency (FGS, B73v3)
Median Position Relative to TSS = -113	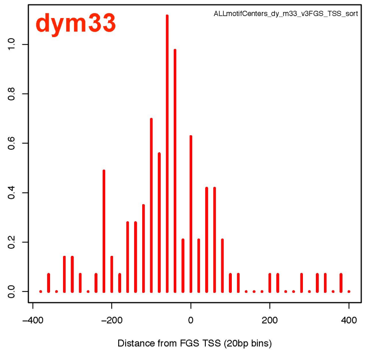	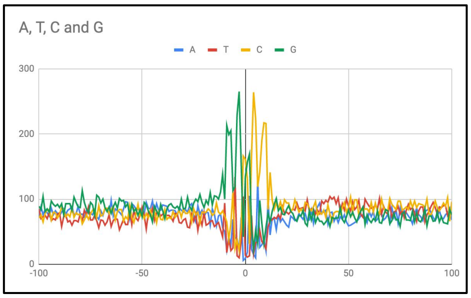

Average MOA Coverage Around Motif (RPM)

All	Not in Repeats (NR)	In Repeats (IR)
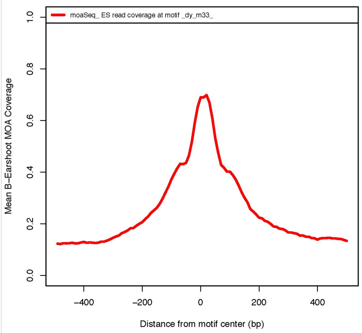	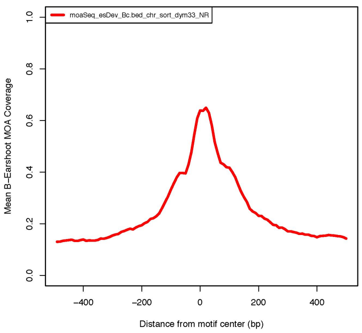	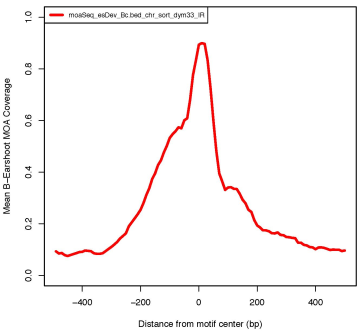

Motif Name	Total Number	In Repeats
**dym34**	**1,496**	**43%**
**Consensus**	waAATTTATtt	
**LOGO**	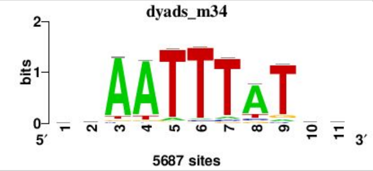	
**LOGO RC**	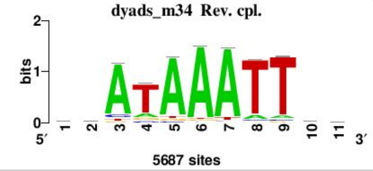	

	Frequency Distr. at TSS (FGS, B73v3)	Average Local Base Frequency (FGS, B73v3)
Median Position Relative to TSS = **-55**	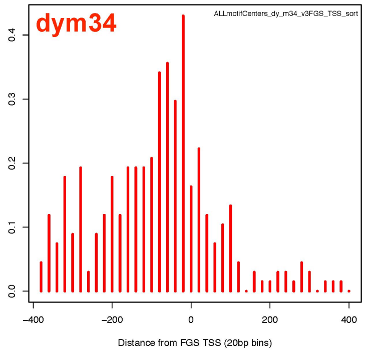	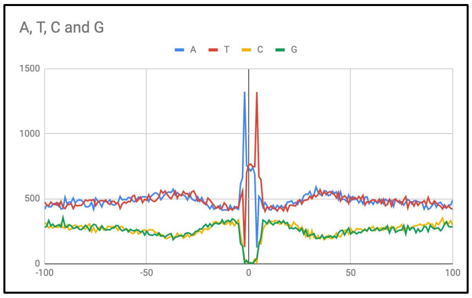

Average MOA Coverage Around Motif (RPM)

All	Not in Repeats (NR)	In Repeats (IR)
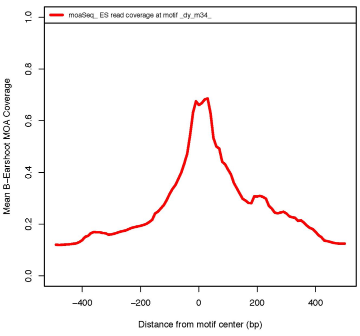	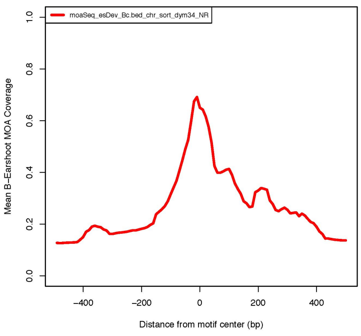	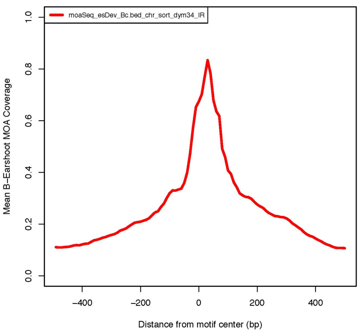

Motif Name	Total Number	In Repeats
dym35	3,479	8%
Consensus	csCACssCACvs	
LOGO	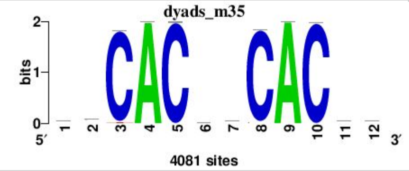	
LOGO RC	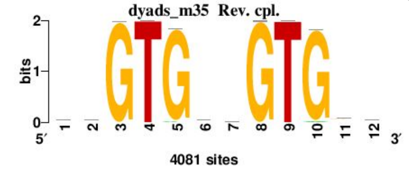	

	Frequency Distr. at TSS (FGS, B73v3)	Average Local Base Frequency (FGS, B73v3)
Median Position Relative to TSS =	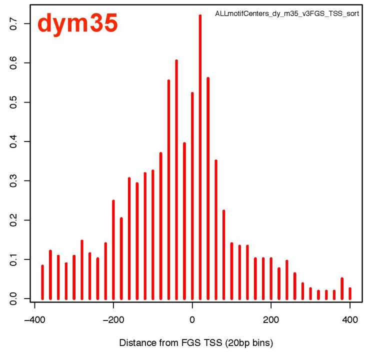	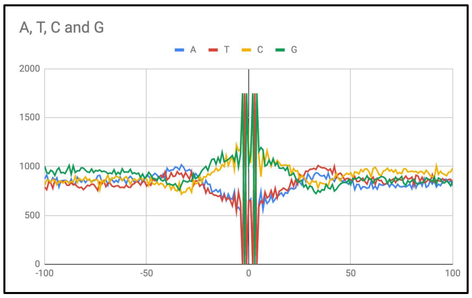
-61		

Average MOA Coverage Around Motif (RPM)

All	Not in Repeats (NR)	In Repeats (IR)
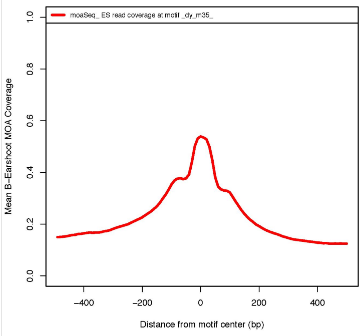	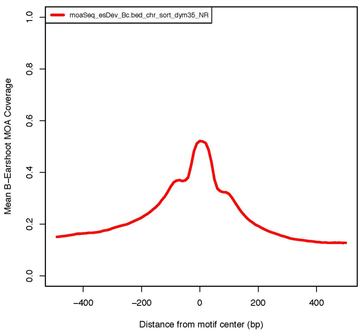	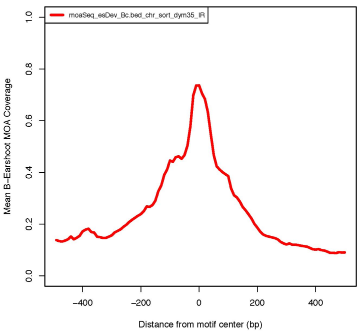

Motif Name	Total Number	In Repeats
dym36	1,828	12%
Consensus	rrAGArArAGArAGAGAGAGAGrg	
LOGO	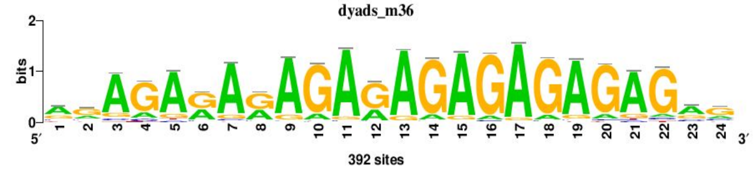	
LOGO RC	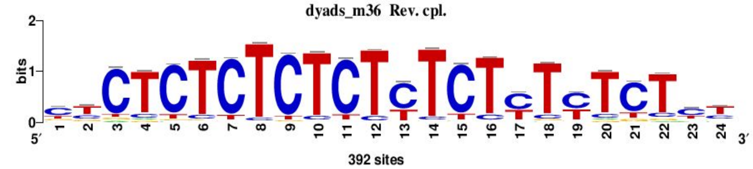	

	Frequency Distr. at TSS (FGS, B73v3)	Average Local Base Frequency (FGS, B73v3)
Median Position Relative to TSS =	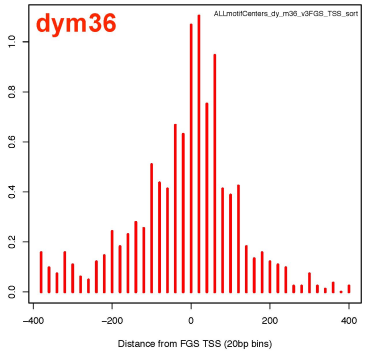	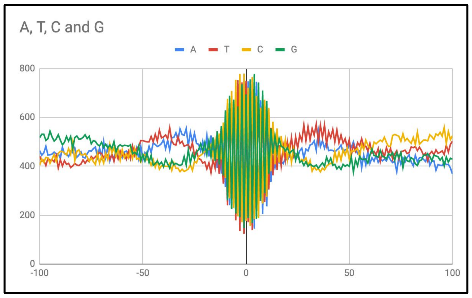
-25		

Average MOA Coverage Around Motif (RPM)

All	Not in Repeats (NR)	In Repeats (IR)
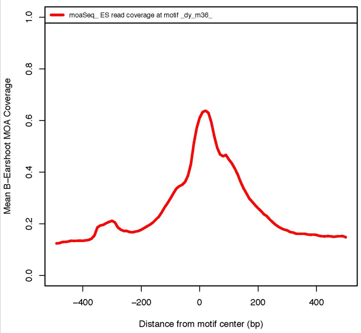	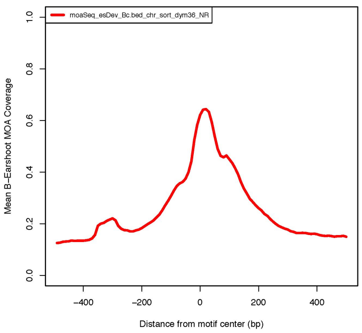	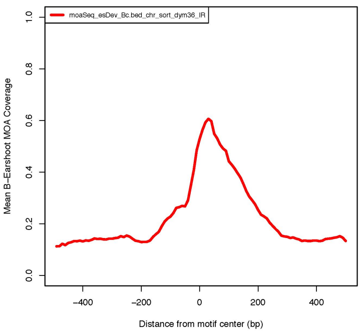

Motif Name	Total Number	In Repeats
dym37	656	11%
Consensus	sstCCCssCCCGGCCCGGsCCasc	

LOGO

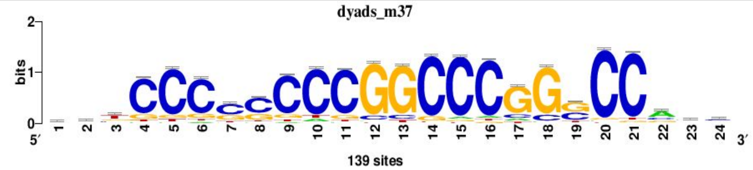

LOGO RC

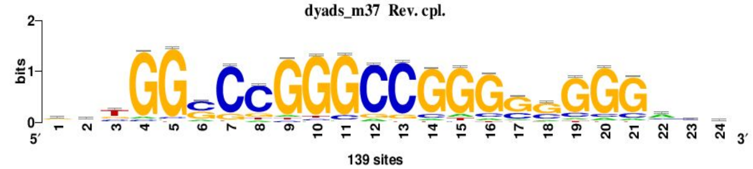

	Frequency Distr. at TSS (FGS, B73v3)	Average Local Base Frequency (FGS, B73v3)
Median Position Relative to TSS = -87	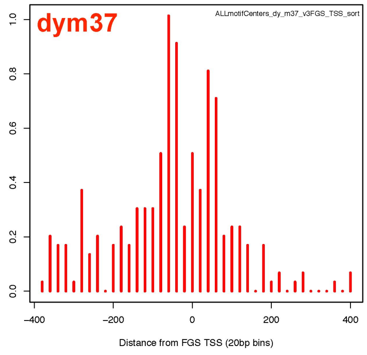	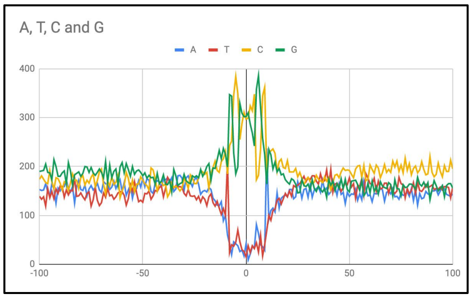

Average MOA Coverage Around Motif (RPM)

All	Not in Repeats (NR)	In Repeats (IR)
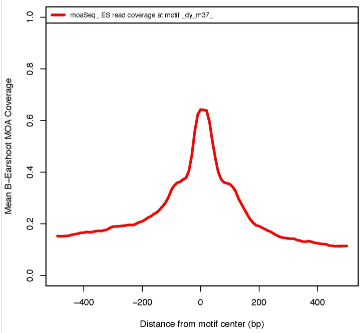	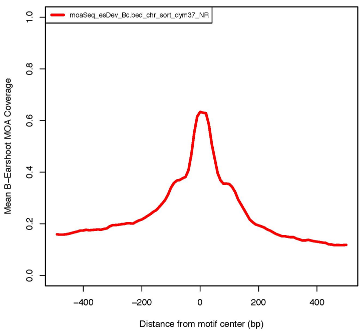	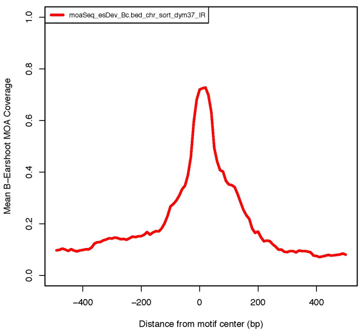

Motif Name	Total Number	In Repeats
dym38	1,591	71%
Consensus	rcCGTmsrwyyysgATCsr	
LOGO	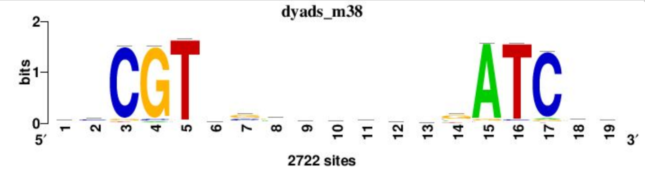	
LOGO RC	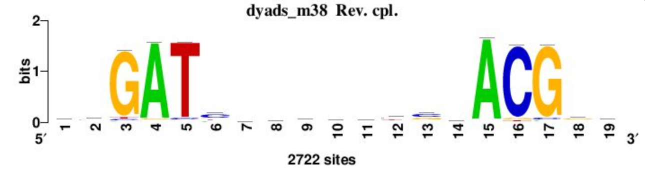	

	Frequency Distr. at TSS (FGS, B73v3)	Average Local Base Frequency (FGS, B73v3)
Median Position Relative to TSS = -97	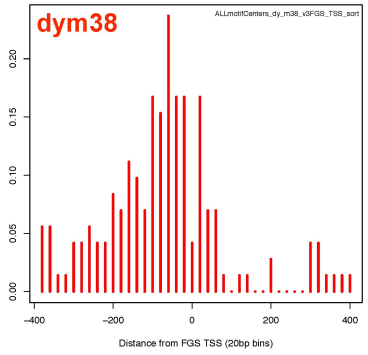	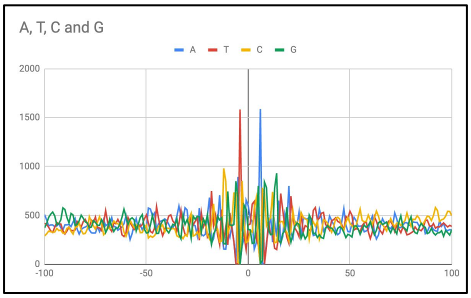

Average MOA Coverage Around Motif (RPM)

All	Not in Repeats (NR)	In Repeats (IR)
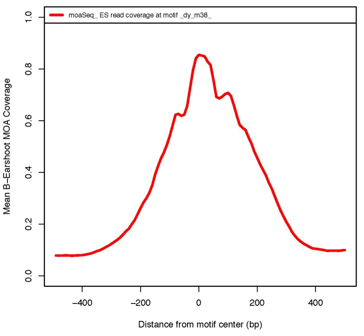	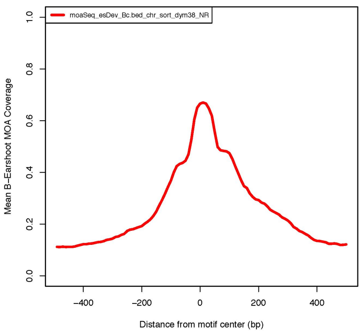	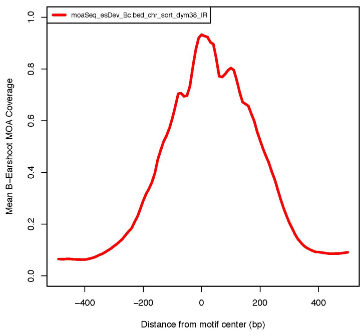

Motif Name	Total Number	In Repeats
dym39	**2,603**	**8%**
**Consensus**	**gsCAGssvssCAGss**	
**LOGO**	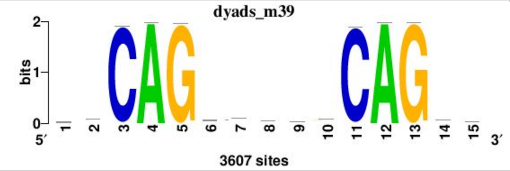	
**LOGO RC**	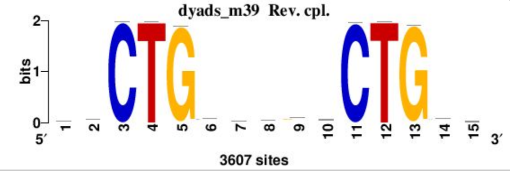	

	Frequency Distr. at TSS (FGS, B73v3)	Average Local Base Frequency (FGS, B73v3)
**Median Position Relative to TSS =** **-72**	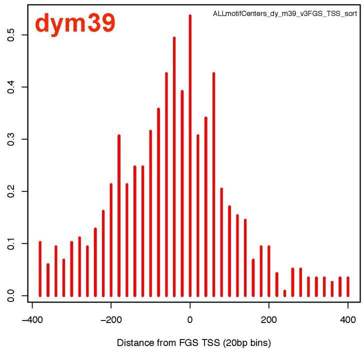	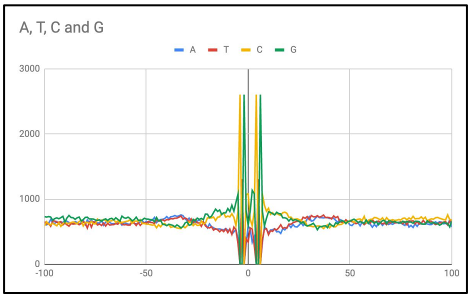

Average MOA Coverage Around Motif (RPM)

All	Not in Repeats (NR)	In Repeats (IR)
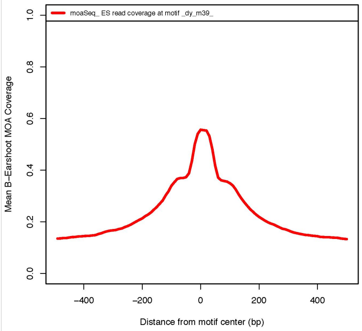	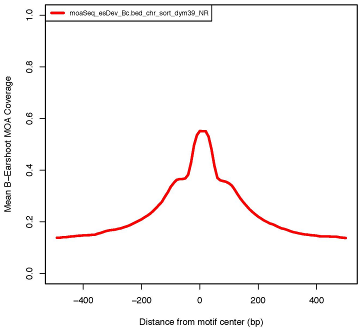	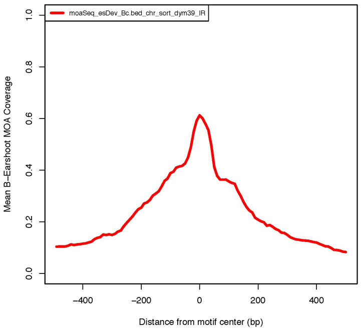

Motif Name	Total Number	In Repeats
**dym40**	**2,875**	**12%**
**Consensus**	ssCCAssssscCCAss	
**LOGO**	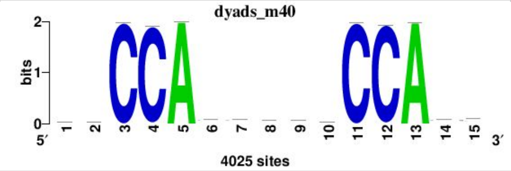	
**LOGO RC**	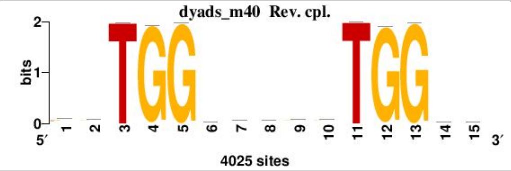	

	Frequency Distr. at TSS (FGS, B73v3)	Average Local Base Frequency (FGS, B73v3)
**Median Position Relative to TSS =**	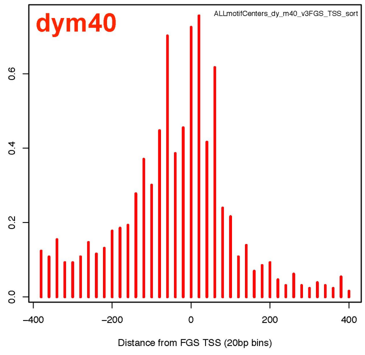	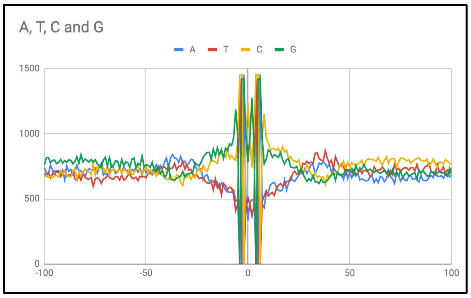
**-66**		

Average MOA Coverage Around Motif (RPM)

All	Not in Repeats (NR)	In Repeats (IR)
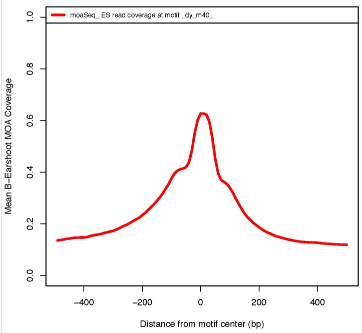	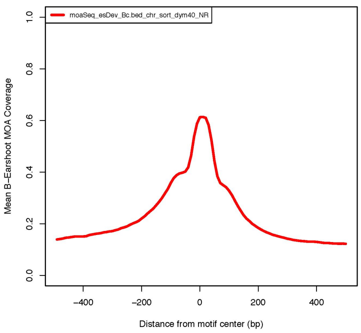	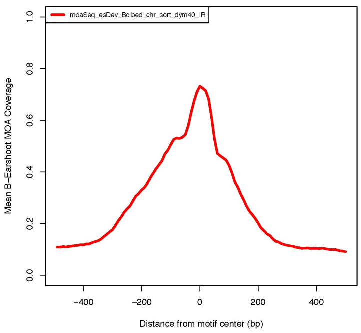

Motif Name	Total Number	In Repeats
dym41	3,700	26%
Consensus	aaAtAAAAACaa	
LOGO	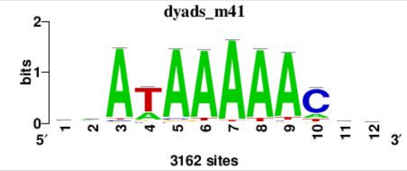	
LOGO RC	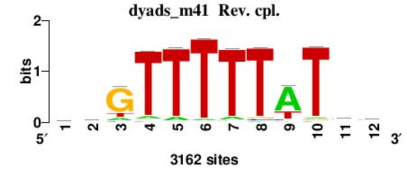	

	Frequency Distr. at TSS (FGS, B73v3)	Average Local Base Frequency (FGS, B73v3)
Median Position Relative to TSS = -79	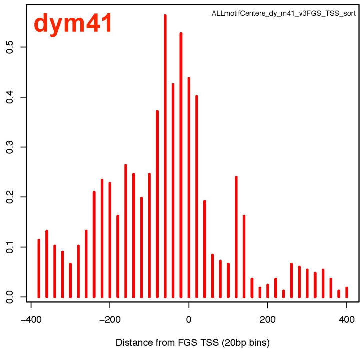	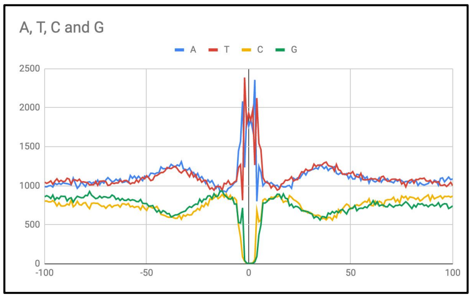

Average MOA Coverage Around Motif (RPM)

All	Not in Repeats (NR)	In Repeats (IR)
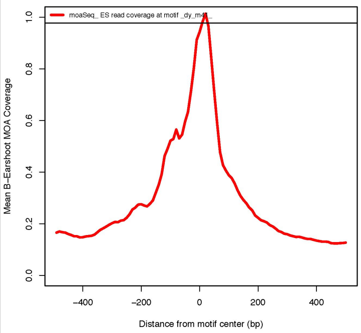	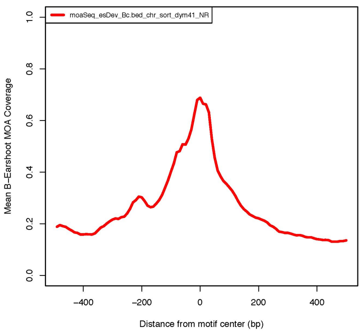	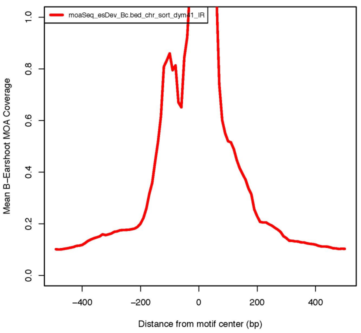

Motif Name	Total Number	In Repeats
dym42	734	14%
Consensus	rgcGGrGTGGGstGGGGGGGagg	
LOGO	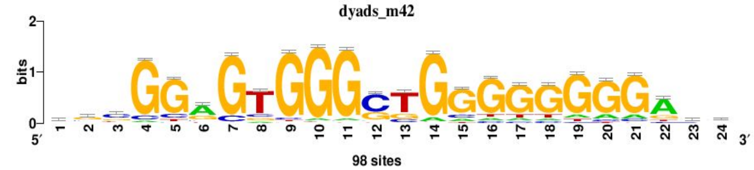	
LOGO RC	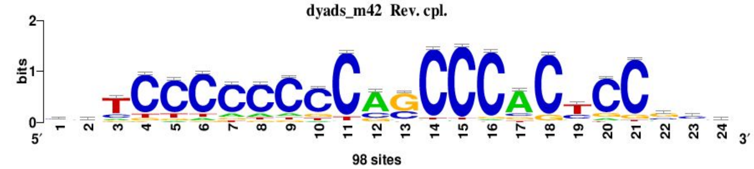	

	Frequency Distr. at TSS (FGS, B73v3)	Average Local Base Frequency (FGS, B73v3)
Median Position Relative to TSS =	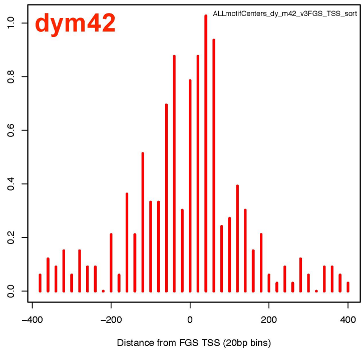	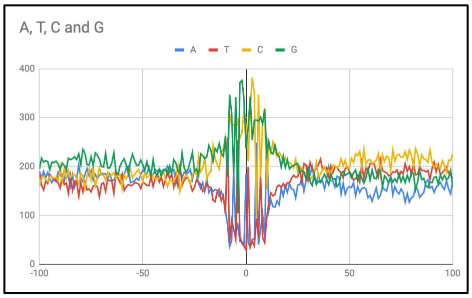
-20		

Average MOA Coverage Around Motif (RPM)

All	Not in Repeats (NR)	In Repeats (IR)
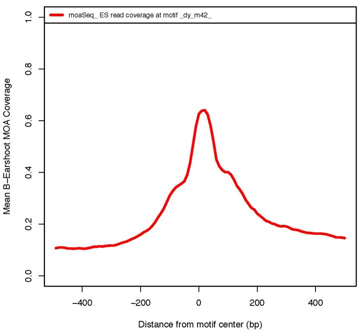	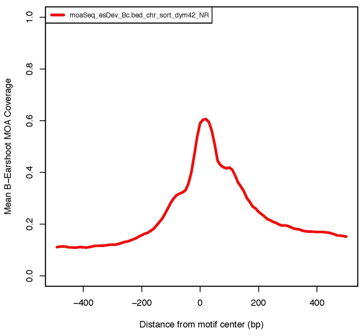	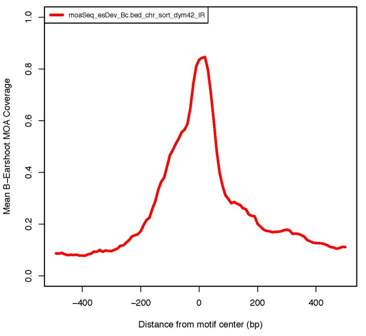

Motif Name	Total Number	In Repeats
dym43	634	88%
Consensus	tgATCsrrcggcysrgrtysvccsrTACcc	
LOGO	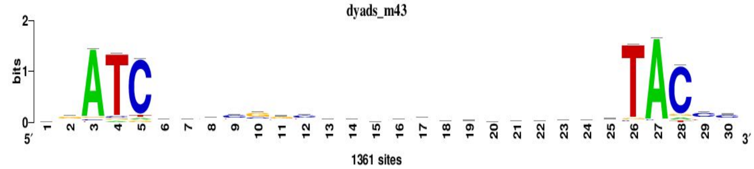	
LOGO RC	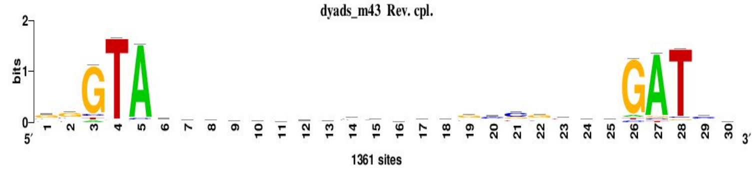	

	Frequency Distr. at TSS (FGS, B73v3)	Average Local Base Frequency (FGS, B73v3)
Median Position Relative to TSS =	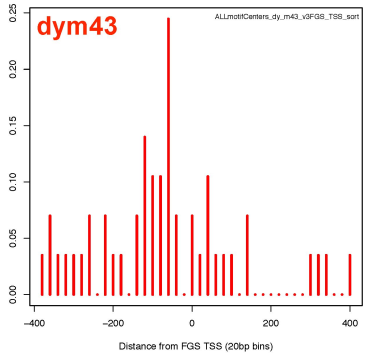	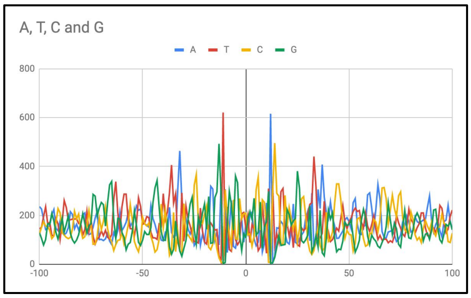
-80		

Average MOA Coverage Around Motif (RPM)

All	Not in Repeats (NR)	In Repeats (IR)
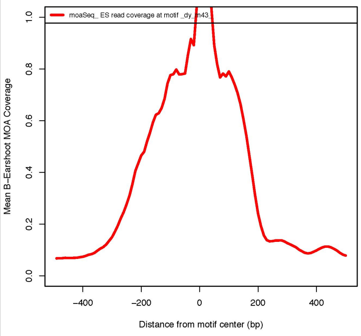	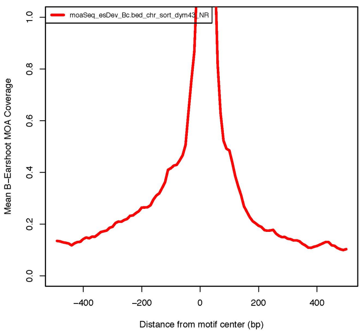	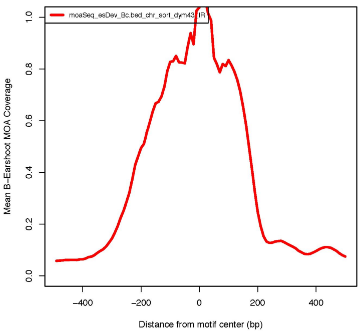

Motif Name	Total Number	In Repeats
dym44	**6,811**	**21%**
**Consensus**	**arAwAAAAAAAgra**	
**LOGO**	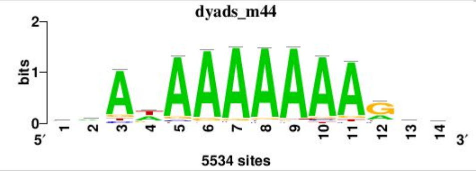	
**LOGO RC**	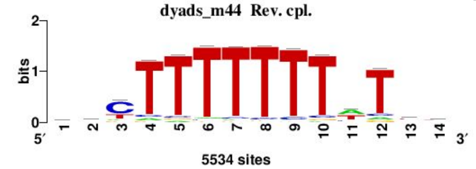	

	Frequency Distr. at TSS (FGS, B73v3)	Average Local Base Frequency (FGS, B73v3)
**Median Position Relative to TSS =** **-81**	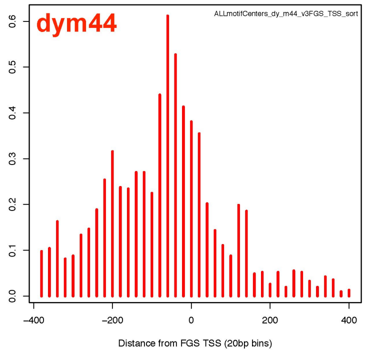	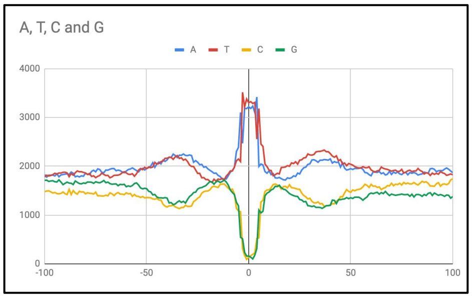

Average MOA Coverage Around Motif (RPM)

All	Not in Repeats (NR)	In Repeats (IR)
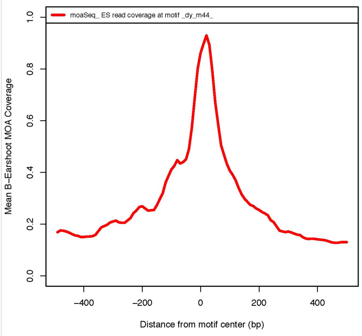	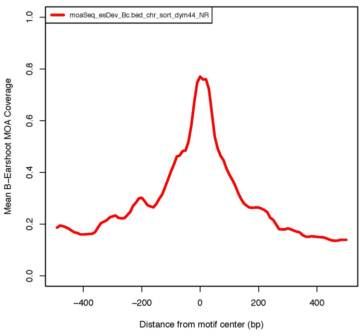	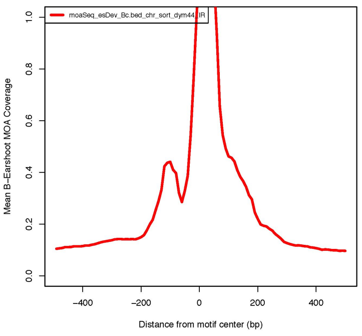

Motif Name	Total Number	In Repeats
dym45	3,058	9%
Consensus	stGCAsbGCAss	
LOGO	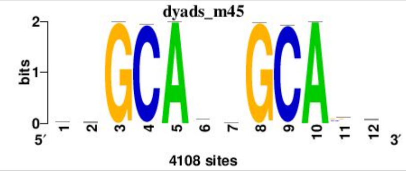	
LOGO RC	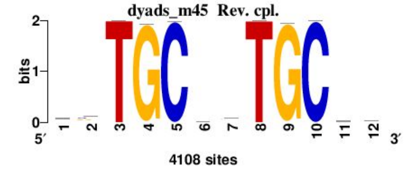	

	Frequency Distr. at TSS (FGS, B73v3)	Average Local Base Frequency (FGS, B73v3)
Median Position Relative to TSS =	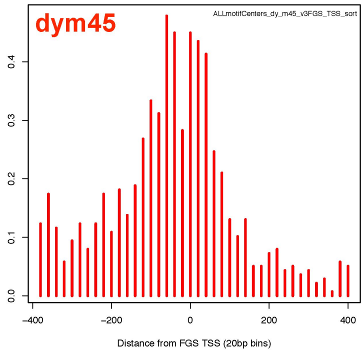	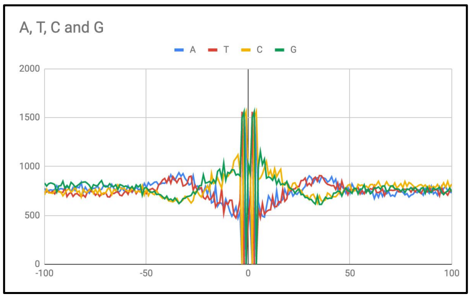
-70		

Average MOA Coverage Around Motif (RPM)

All	Not in Repeats (NR)	In Repeats (IR)
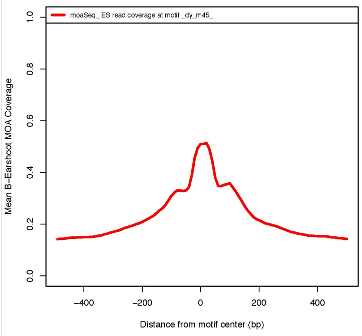	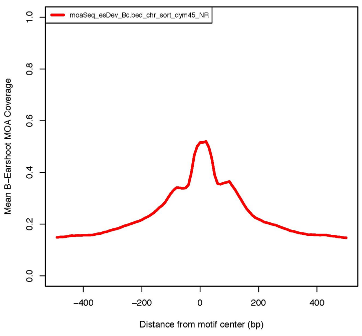	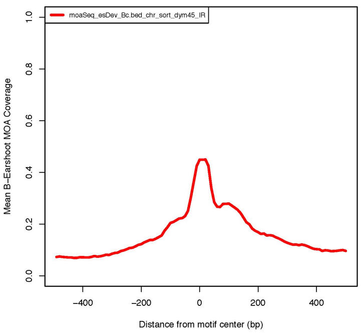

Motif Name	Total Number	In Repeats
dym46	**1,653**	**16%**
**Consensus**	**vyACAsvyACAsv**	
**LOGO**	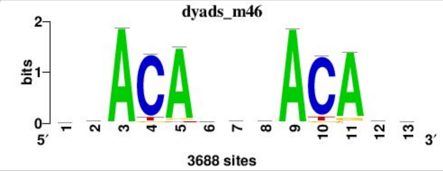	
**LOGO RC**	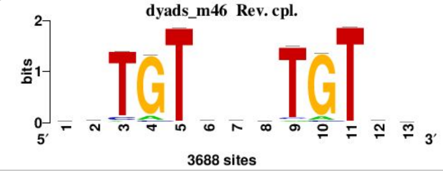	

	Frequency Distr. at TSS (FGS, B73v3)	Average Local Base Frequency (FGS, B73v3)
**Median Position Relative to TSS =** **-42**	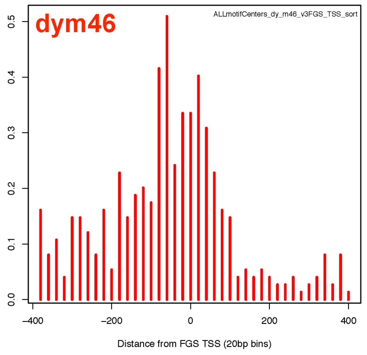	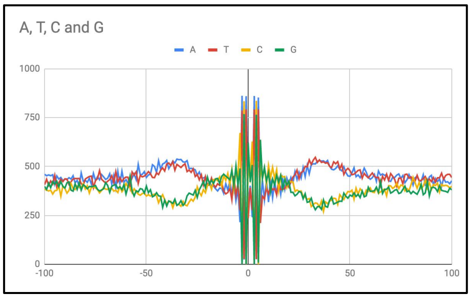

Average MOA Coverage Around Motif (RPM)

All	Not in Repeats (NR)	In Repeats (IR)
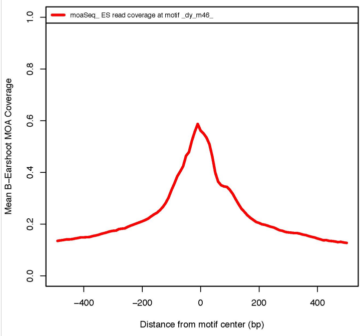	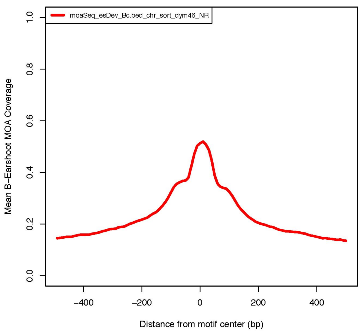	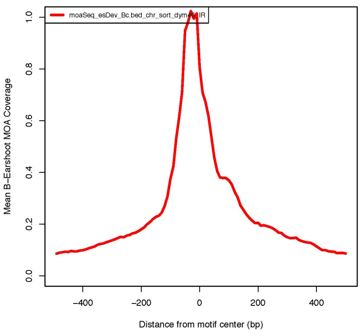

Motif Name	Total Number	In Repeats
dym47	**1,832**	**16%**
**Consensus**	gaGAGAGAGArAGArArArAkwrr	
**LOGO**	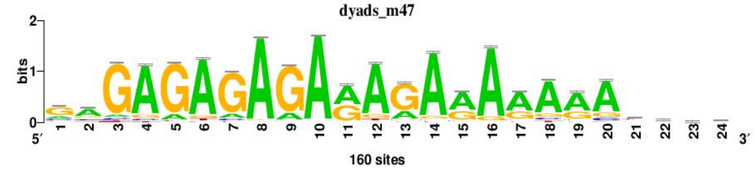	
**LOGO RC**	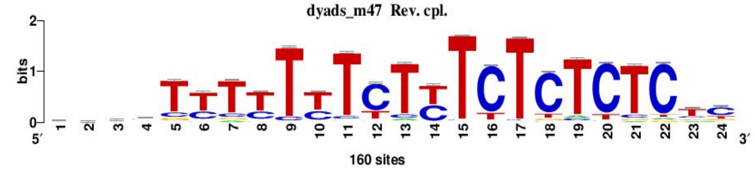	

	Frequency Distr. at TSS (FGS, B73v3)	Average Local Base Frequency (FGS, B73v3)
**Median Position Relative to TSS =** **-44**	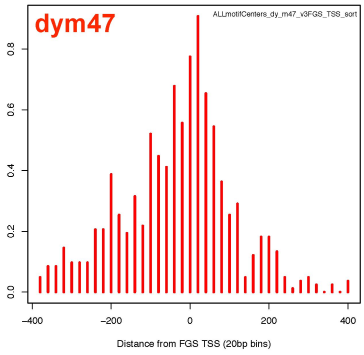	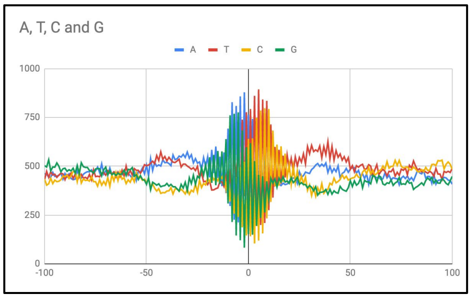

Average MOA Coverage Around Motif (RPM)

All	Not in Repeats (NR)	In Repeats (IR)
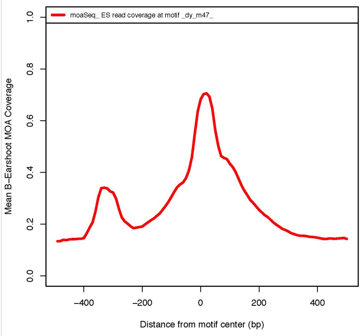	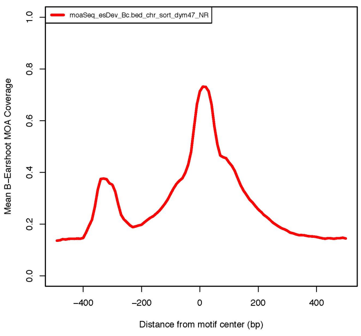	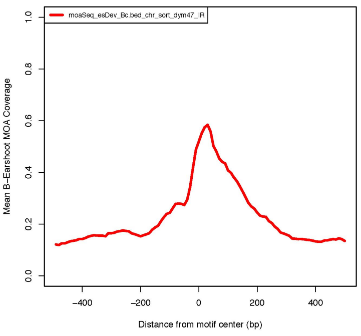

Motif Name	Total Number	In Repeats
dym48	774	19%
Consensus	ssCcCcCCaCGGCCCAGCCCaasc	
LOGO	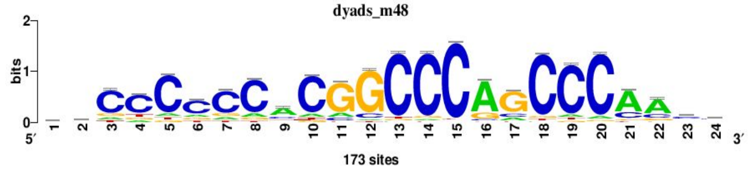	
LOGO RC	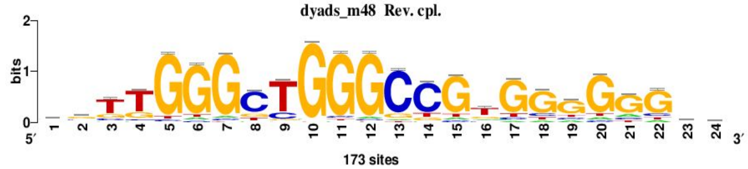	

	Frequency Distr. at TSS (FGS, B73v3)	Average Local Base Frequency (FGS, B73v3)
Median Position Relative to TSS = -65	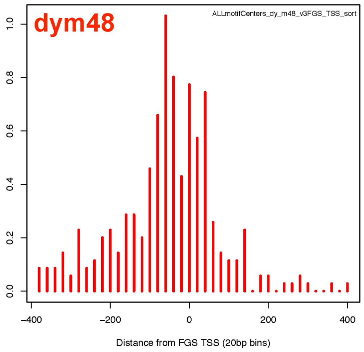	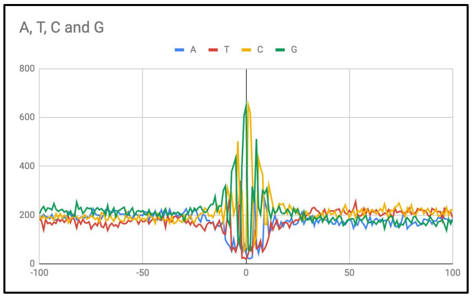

Average MOA Coverage Around Motif (RPM)

All	Not in Repeats (NR)	In Repeats (IR)
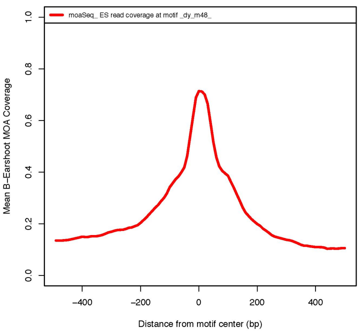	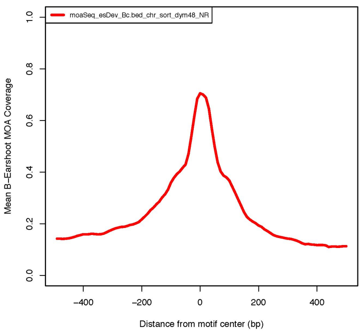	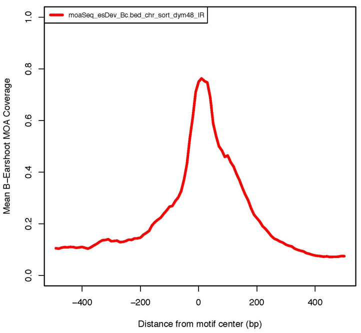

Motif Name	Total Number	In Repeats
dym49	751	11%
Consensus	scaGGCCCACCcCCCCACGccc	
LOGO	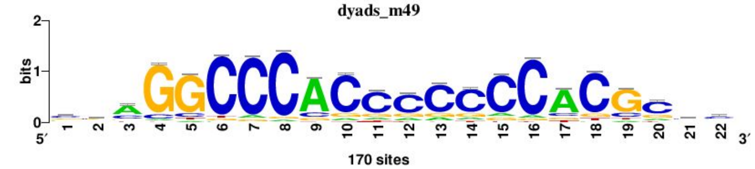	
LOGO RC	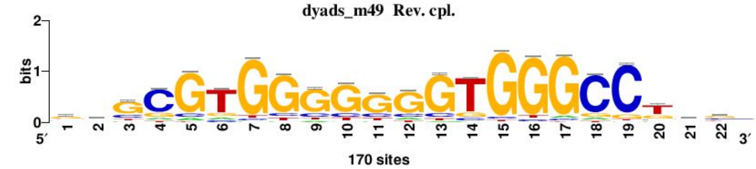	

	Frequency Distr. at TSS (FGS, B73v3)	Average Local Base Frequency (FGS, B73v3)
Median Position Relative to TSS =	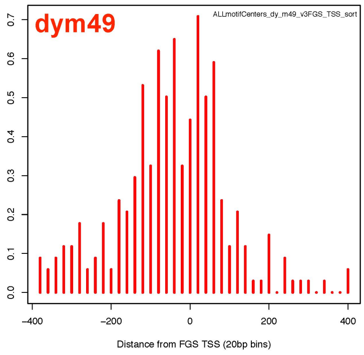	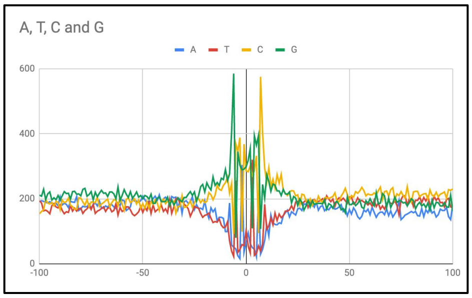
-100		

Average MOA Coverage Around Motif (RPM)

All	Not in Repeats (NR)	In Repeats (IR)
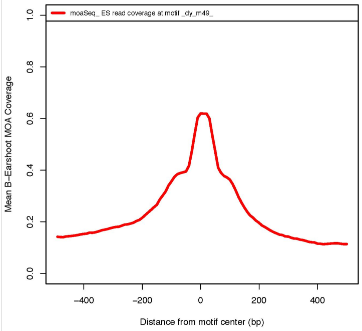	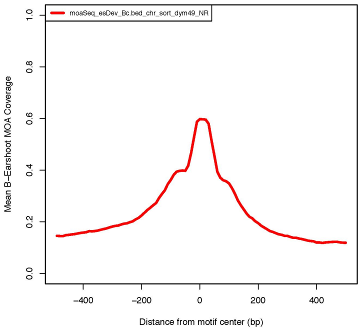	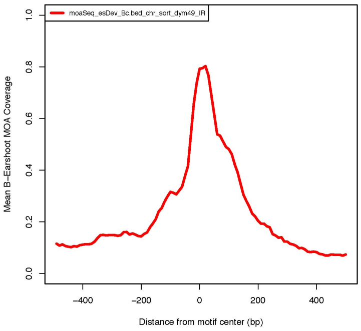

Motif Name	Total Number	In Repeats
dym50	6,175	20%
Consensus	aamAAAAAAAAArra	
LOGO	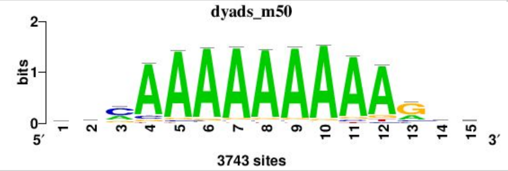	
LOGO RC	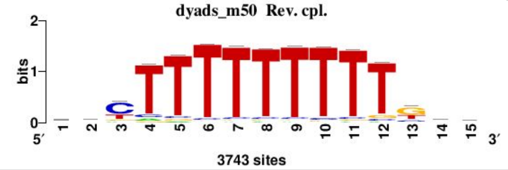	

	Frequency Distr. at TSS (FGS, B73v3)	Average Local Base Frequency (FGS, B73v3)
Median Position Relative to TSS = -81	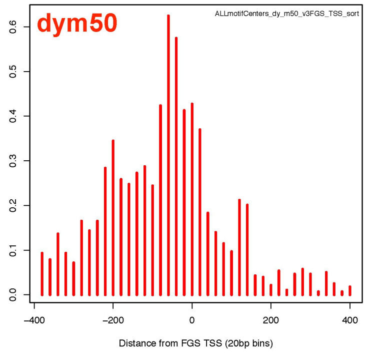	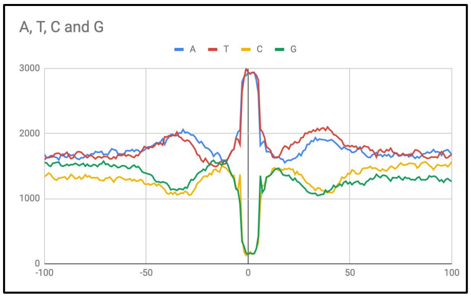

Average MOA Coverage Around Motif (RPM)

All	Not in Repeats (NR)	In Repeats (IR)
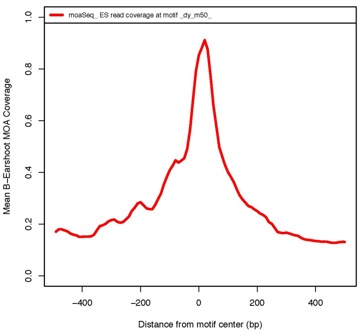	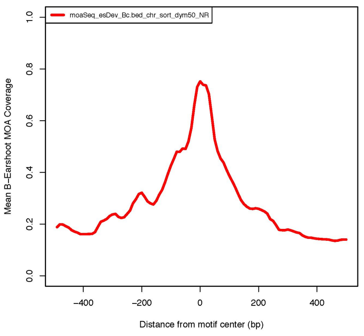	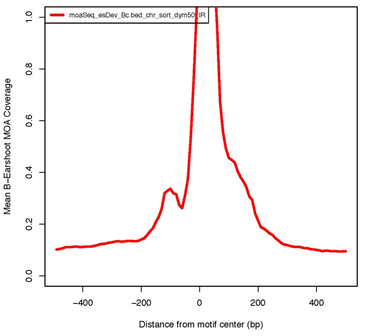

Motif Name	Total Number	In Repeats
dym51	1,867	8%
Consensus	ssGACgrsGACgs	
LOGO	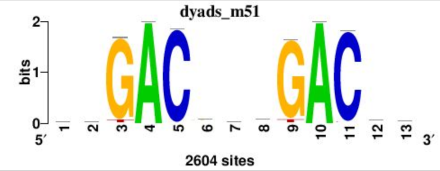	
LOGO RC	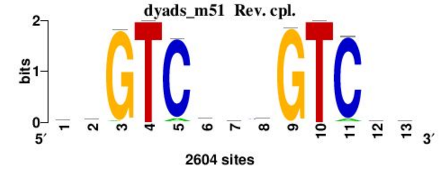	

	Frequency Distr. at TSS (FGS, B73v3)	Average Local Base Frequency (FGS, B73v3)
Median Position Relative to TSS =	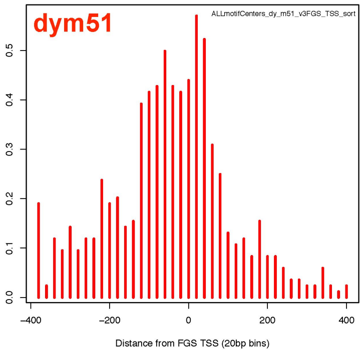	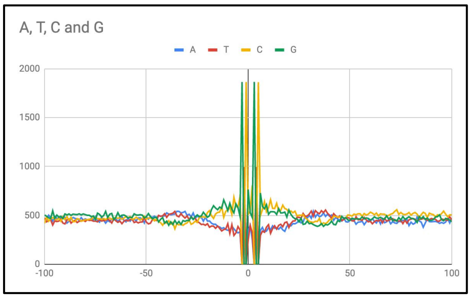
-102		

Average MOA Coverage Around Motif (RPM)

All	Not in Repeats (NR)	In Repeats (IR)
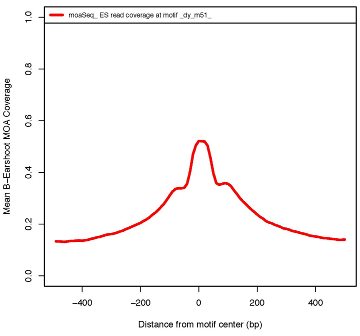	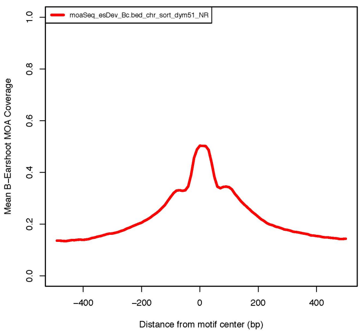	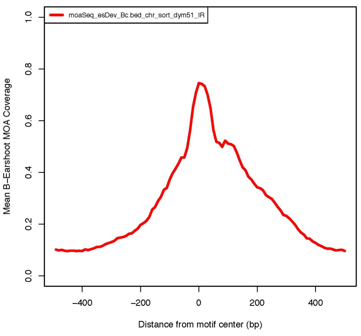

Motif Name	Total Number	In Repeats
dym52	1,435	86%
Consensus	brATCygrrccstmsrgyycrGATCcr	
LOGO	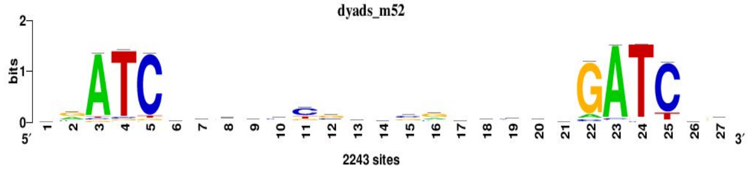	
LOGO RC	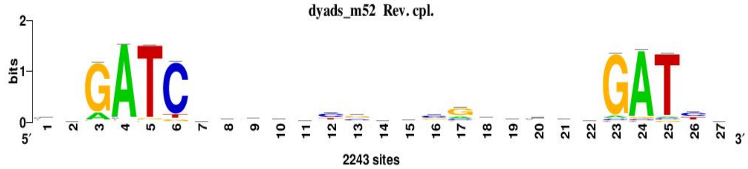	

	Frequency Distr. at TSS (FGS, B73v3)	Average Local Base Frequency (FGS, B73v3)
Median Position Relative to TSS = -79	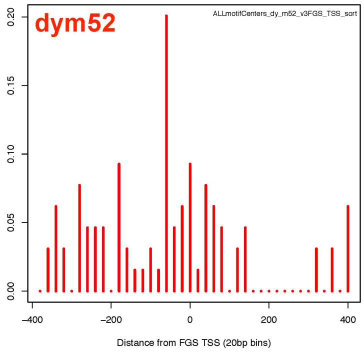	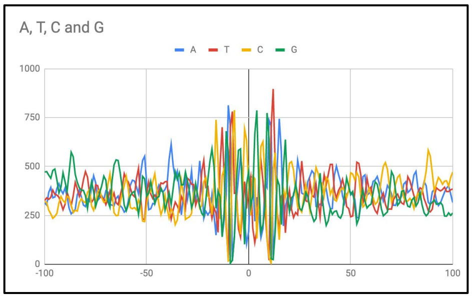

Average MOA Coverage Around Motif (RPM)

All	Not in Repeats (NR)	In Repeats (IR)
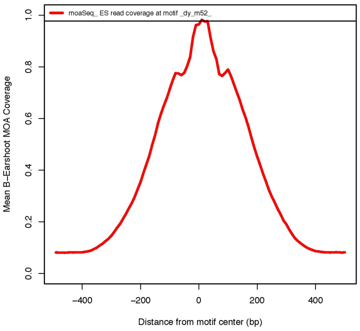	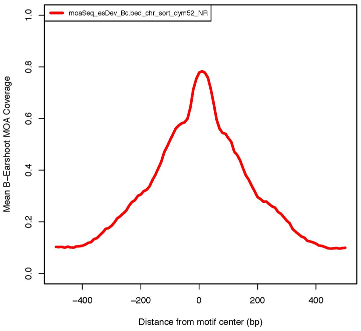	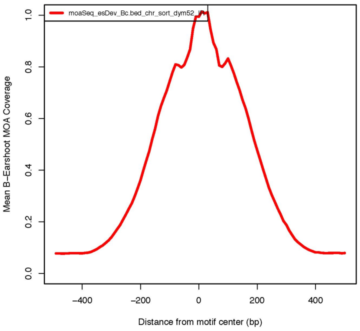

Motif Name	Total Number	In Repeats
**dym53**	**569**	**15%**
**Consensus**	mgcCcCCasGGCCCAGCCCAtCms	
**LOGO**	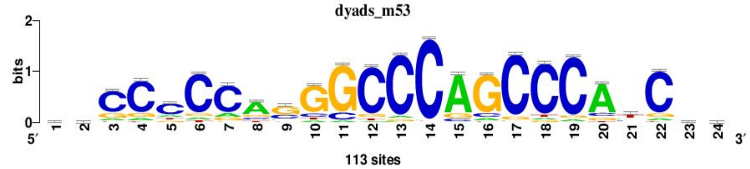	
**LOGO RC**	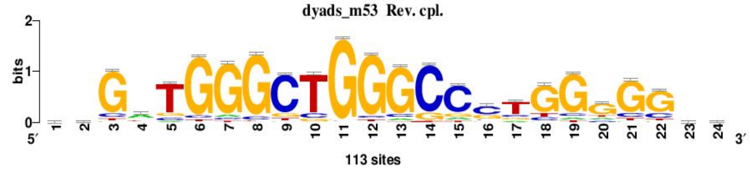	

	Frequency Distr. at TSS (FGS, B73v3)	Average Local Base Frequency (FGS, B73v3)
Median Position Relative to TSS =	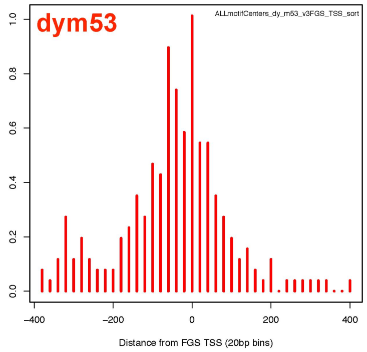	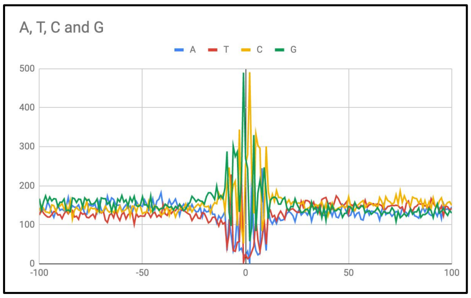
**-57**		

Average MOA Coverage Around Motif (RPM)

All	Not in Repeats (NR)	In Repeats (IR)
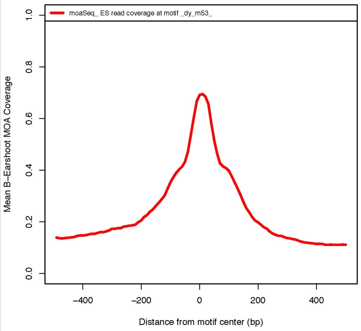	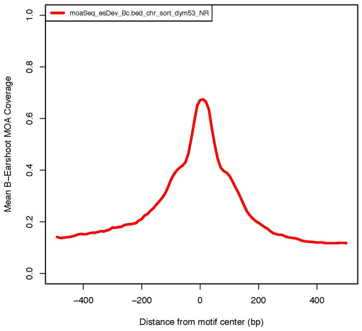	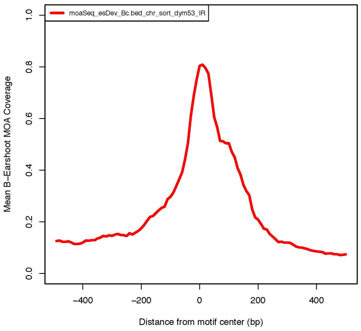

Motif Name	Total Number	In Repeats
dym54	3,450	25%
Consensus	rawAAArAAAAAAAAAwAAmrr	
LOGO	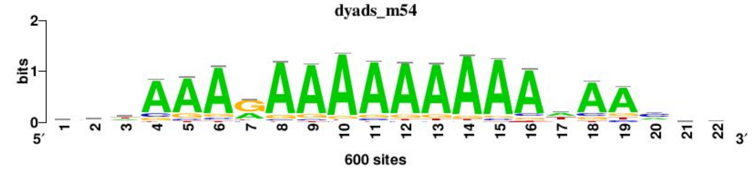	
LOGO RC	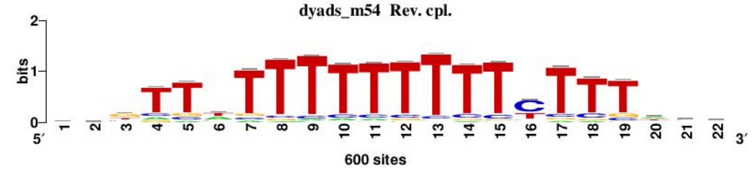	

	Frequency Distr. at TSS (FGS, B73v3)	Average Local Base Frequency (FGS, B73v3)
Median Position Relative to TSS =	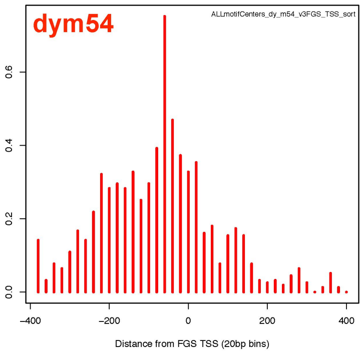	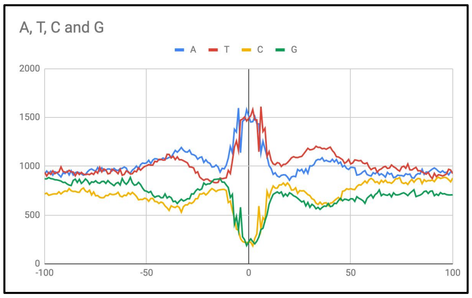
-85		

Average MOA Coverage Around Motif (RPM)

All	Not in Repeats (NR)	In Repeats (IR)
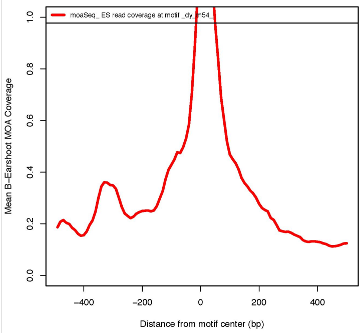	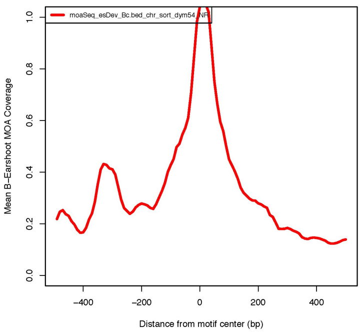	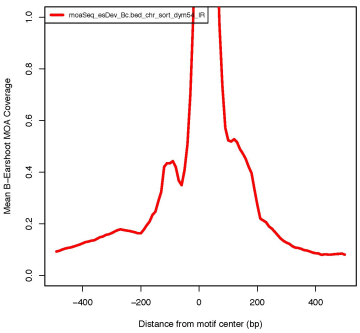

Motif Name	Total Number	In Repeats
dym55	884	8%
Consensus	scCCaCCaCCCCACCcCCCasCmc	
LOGO	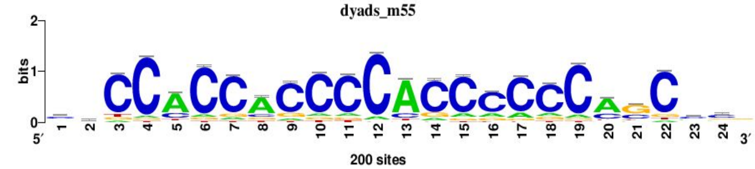	
LOGO RC	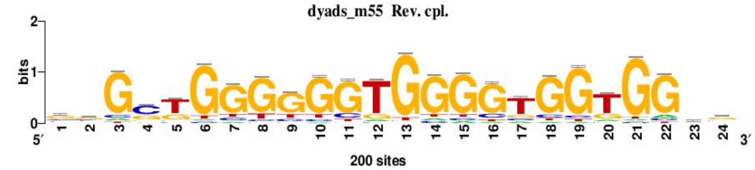	

	Frequency Distr. at TSS (FGS, B73v3)	Average Local Base Frequency (FGS, B73v3)
Median Position Relative to TSS =	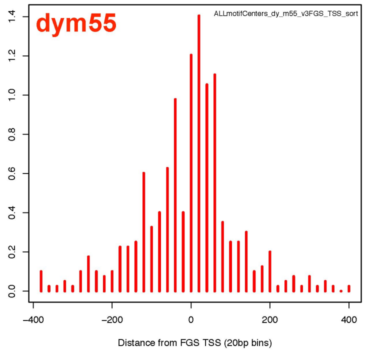	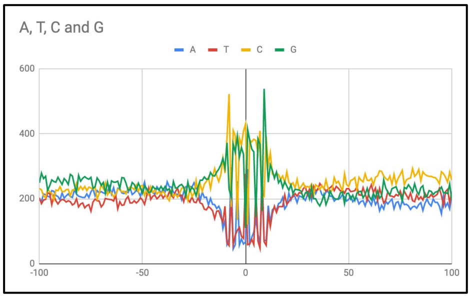
-16		

Average MOA Coverage Around Motif (RPM)

All	Not in Repeats (NR)	In Repeats (IR)
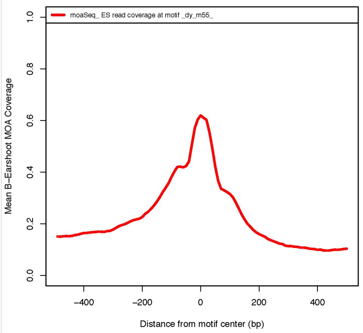	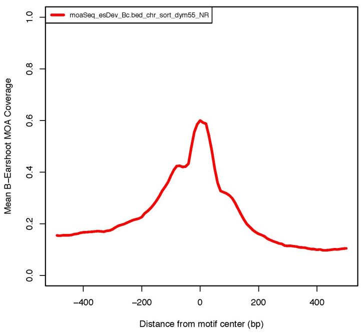	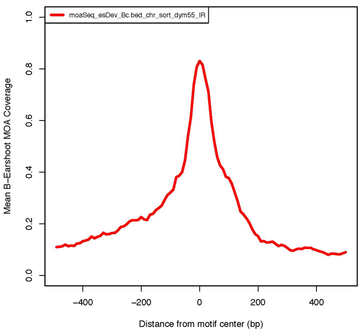

Motif Name	Total Number	In Repeats
dym56	1,889	19%
Consensus	wvCAAAAGmr	
LOGO	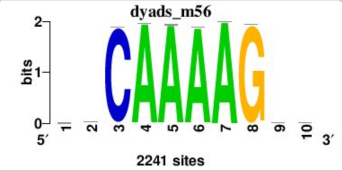	
LOGO RC	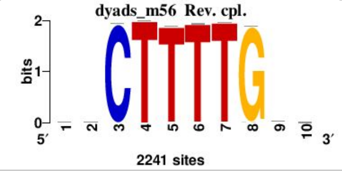	

	Frequency Distr. at TSS (FGS, B73v3)	Average Local Base Frequency (FGS, B73v3)
Median Position Relative to TSS =	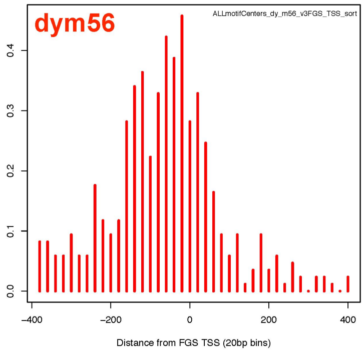	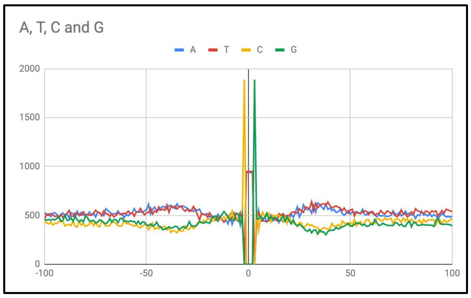
-49		

Average MOA Coverage Around Motif (RPM)

All	Not in Repeats (NR)	In Repeats (IR)
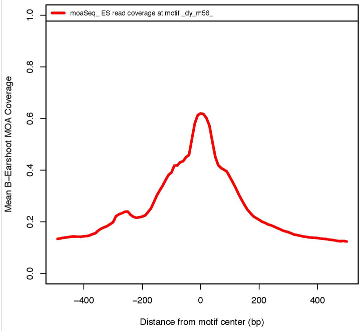	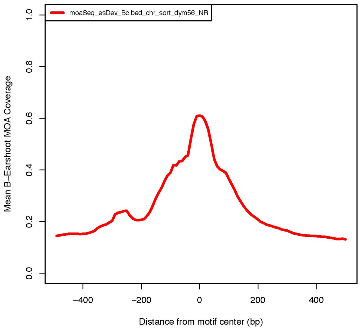	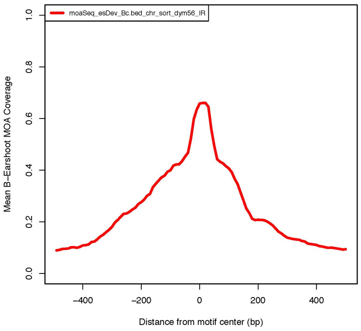

Motif Name	Total Number	In Repeats
dym57	2,382	13%
Consensus	msGACGACgr	
LOGO	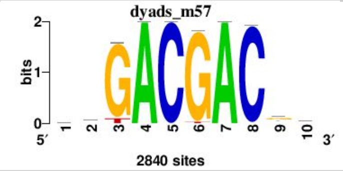	
LOGO RC	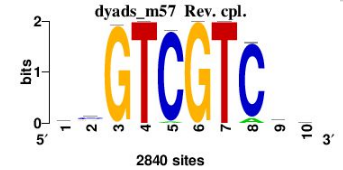	

	Frequency Distr. at TSS (FGS, B73v3)	Average Local Base Frequency (FGS, B73v3)
Median Position Relative to TSS =	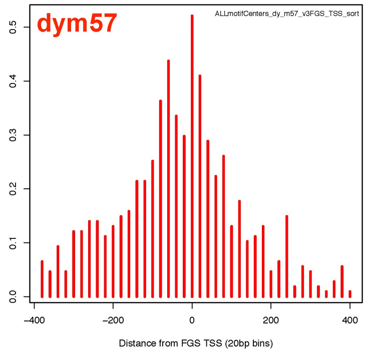	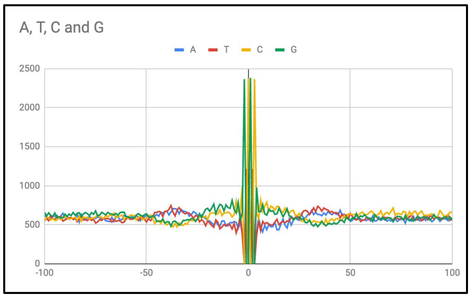
-83		

Average MOA Coverage Around Motif (RPM)

All	Not in Repeats (NR)	In Repeats (IR)
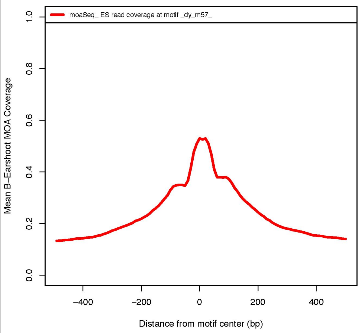	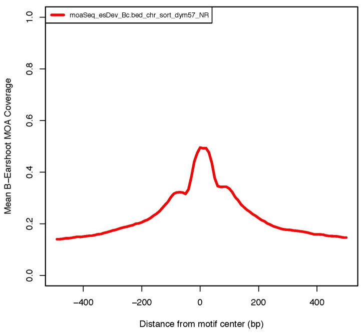	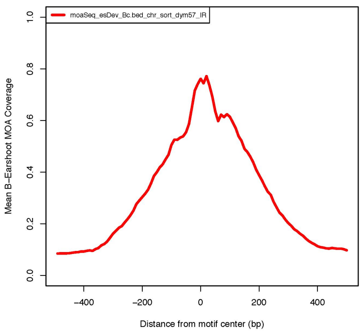

Motif Name	Total Number	In Repeats
dym58	1,676	9%
Consensus	agwGrGAgrGAGAGAGAGAGrrrg	
LOGO	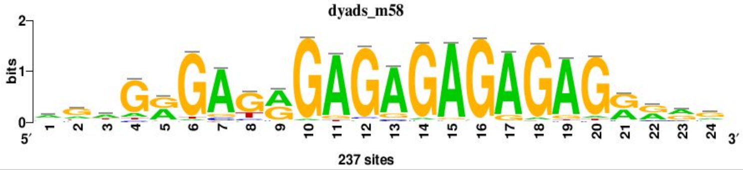	
LOGO RC	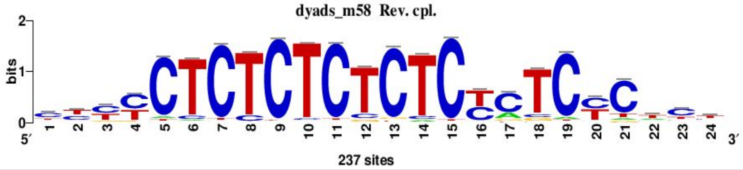	

	Frequency Distr. at TSS (FGS, B73v3)	Average Local Base Frequency (FGS, B73v3)
Median Position Relative to TSS =	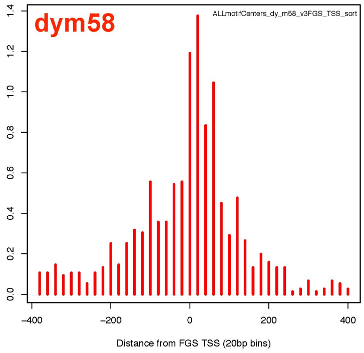	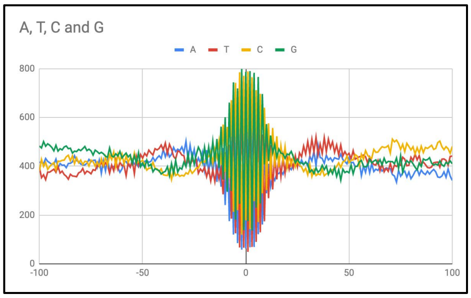
-17		

Average MOA Coverage Around Motif (RPM)

All	Not in Repeats (NR)	In Repeats (IR)
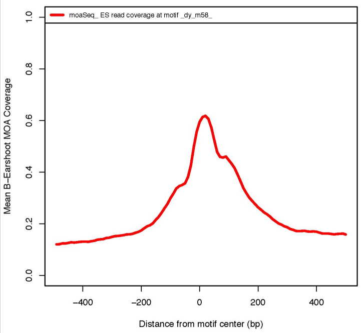	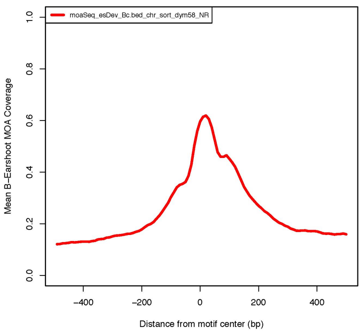	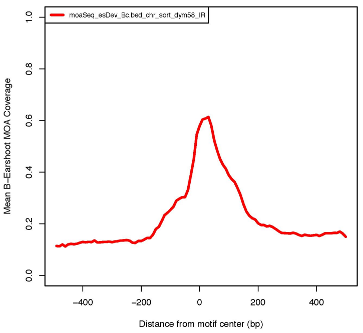

Motif Name	Total Number	In Repeats
dym59	1,829	12%
Consensus	svCGAssrCGAss	
LOGO	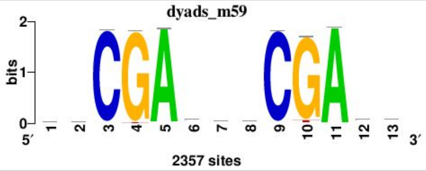	
LOGO RC	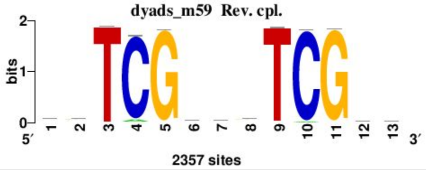	

	Frequency Distr. at TSS (FGS, B73v3)	Average Local Base Frequency (FGS, B73v3)
Median Position Relative to TSS =	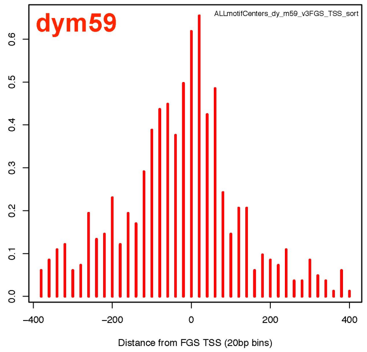	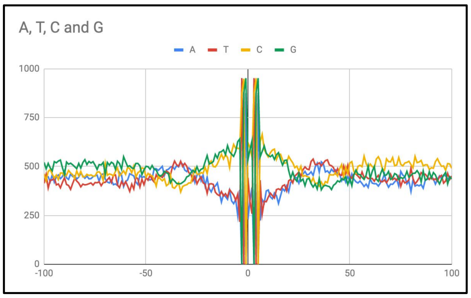
-65		

Average MOA Coverage Around Motif (RPM)

All	Not in Repeats (NR)	In Repeats (IR)
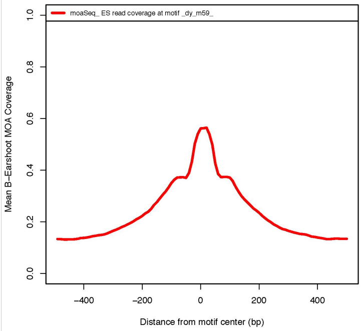	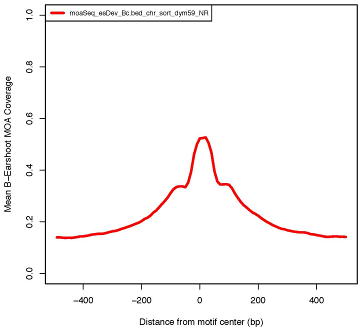	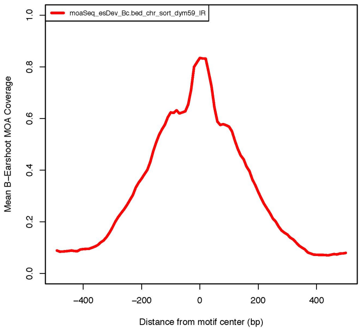

Motif Name	Total Number	In Repeats
dym60	**1,568**	**27%**
**Consensus**	**svTCGATCGAbs**	
**LOGO**	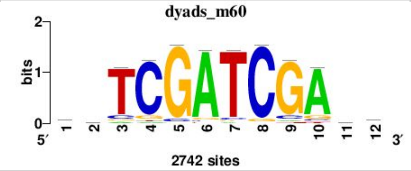	
**LOGO RC**	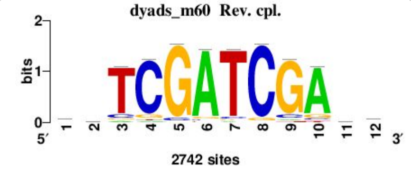	

	Frequency Distr. at TSS (FGS, B73v3)	Average Local Base Frequency (FGS, B73v3)
**Median Position Relative to TSS =**	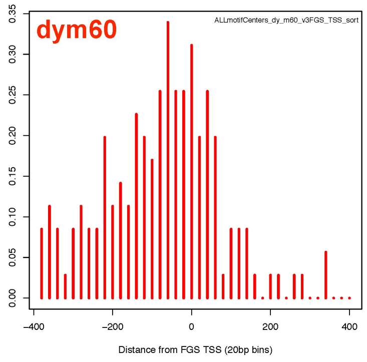	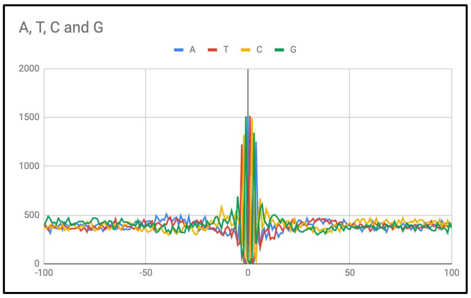
**-156**		

Average MOA Coverage Around Motif (RPM)

All	Not in Repeats (NR)	In Repeats (IR)
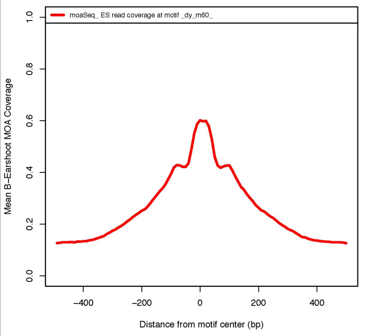	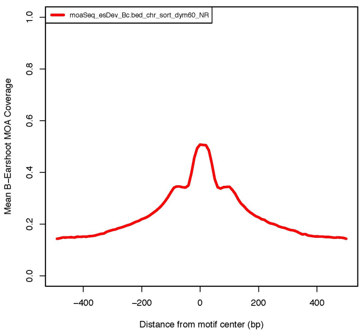	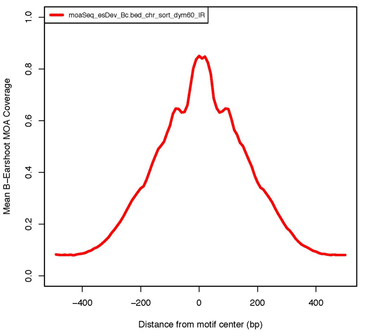

Motif Name	Total Number	In Repeats
**dym61**	**1,690**	**44%**
**Consensus**	**wwATTATTTAAww**	
**LOGO**	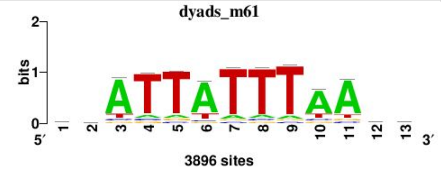	
**LOGO RC**	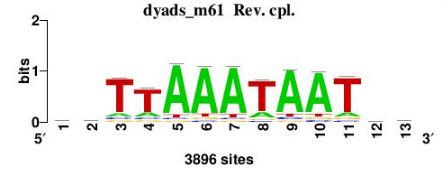	

	Frequency Distr. at TSS (FGS, B73v3)	Average Local Base Frequency (FGS, B73v3)
**Median Position Relative to TSS =** **-74**	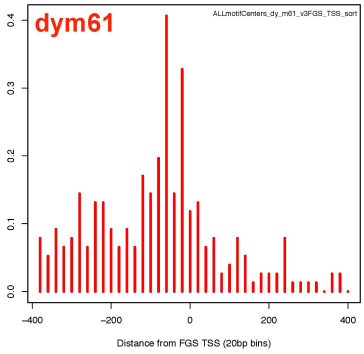	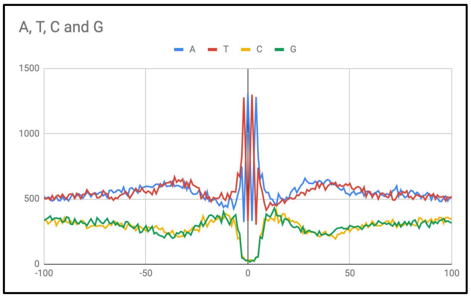

Average MOA Coverage Around Motif (RPM)

All	Not in Repeats (NR)	In Repeats (IR)
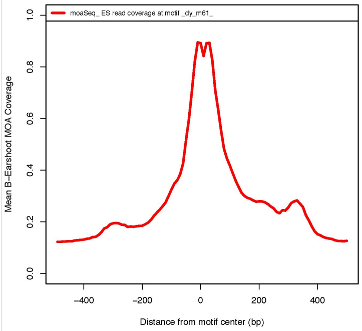	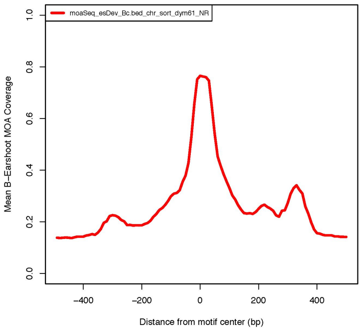	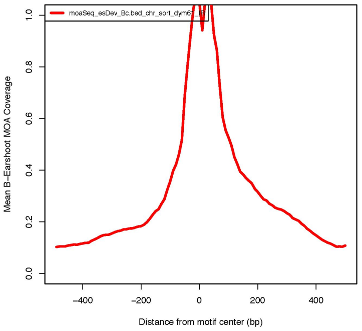

Motif Name	Total Number	In Repeats
dym62	884	18%
Consensus	ssCCCggCCGGCCCaCCCCcCCmc	
LOGO	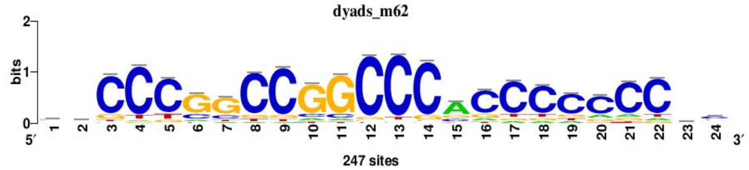	
LOGO RC	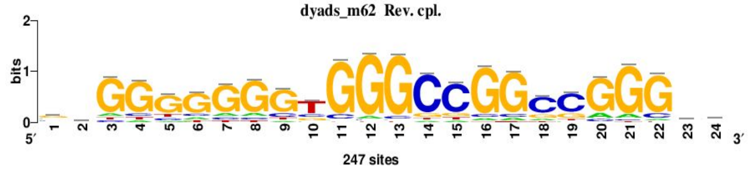	

	Frequency Distr. at TSS (FGS, B73v3)	Average Local Base Frequency (FGS, B73v3)
Median Position Relative to TSS =	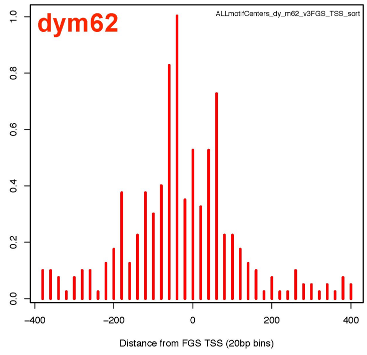	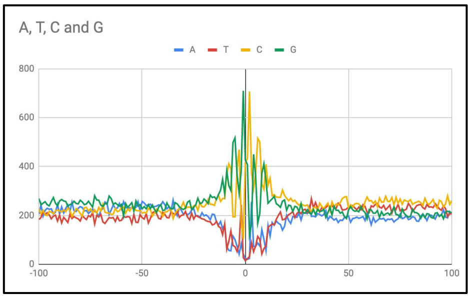
-63		

Average MOA Coverage Around Motif (RPM)

All	Not in Repeats (NR)	In Repeats (IR)
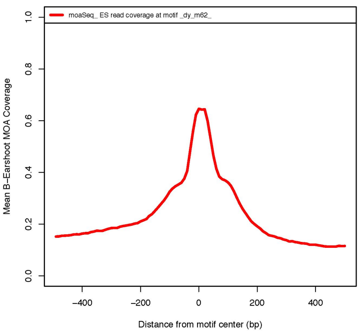	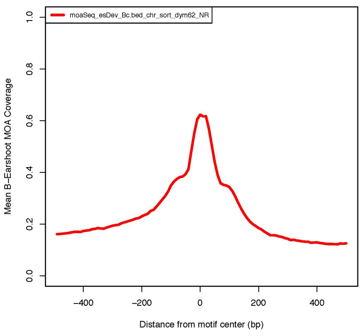	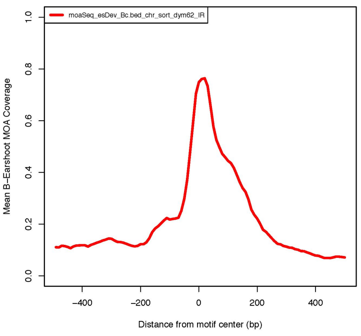

Motif Name	Total Number	In Repeats
dym63	**2,587**	**16%**
**Consensus**	gtGGTGGAsg	
**LOGO**	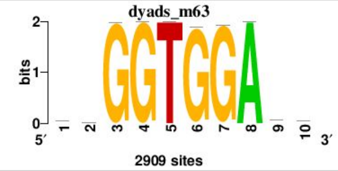	
**LOGO RC**	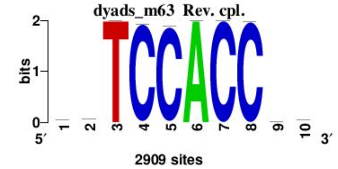	

	Frequency Distr. at TSS (FGS, B73v3)	Average Local Base Frequency (FGS, B73v3)
**Median Position Relative to TSS =** **-50**	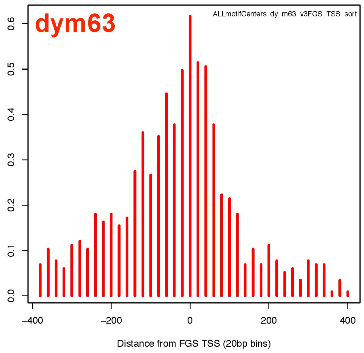	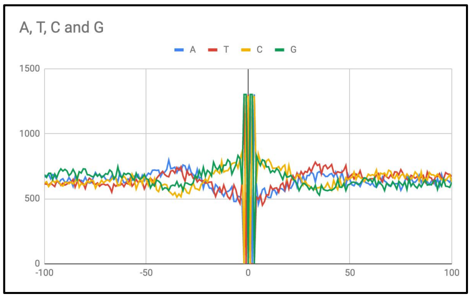

Average MOA Coverage Around Motif (RPM)

All	Not in Repeats (NR)	In Repeats (IR)
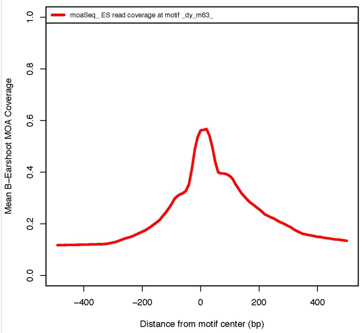	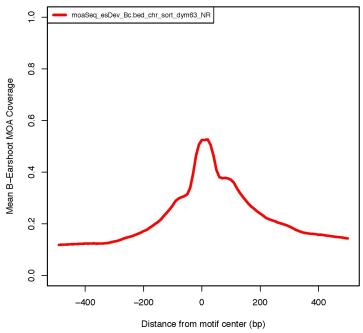	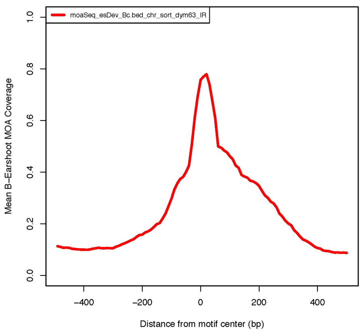

Motif Name	Total Number	In Repeats
dym64	1,841	10%
Consensus	sbACGvsbACGgv	
LOGO	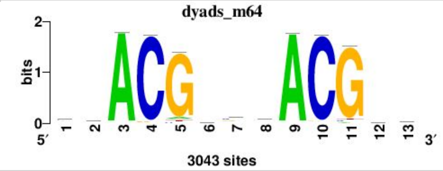	
LOGO RC	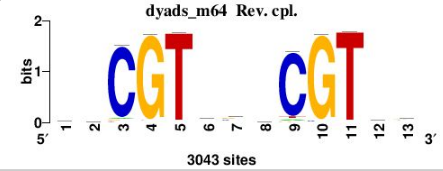	

	Frequency Distr. at TSS (FGS, B73v3)	Average Local Base Frequency (FGS, B73v3)
Median Position Relative to TSS = -79	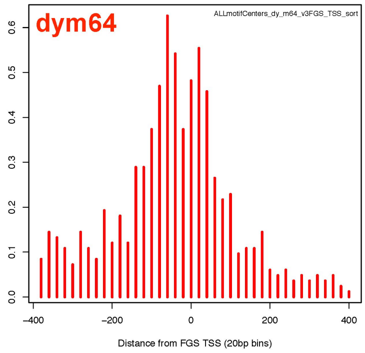	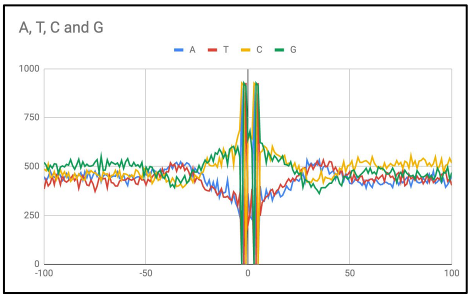

Average MOA Coverage Around Motif (RPM)

All	Not in Repeats (NR)	In Repeats (IR)
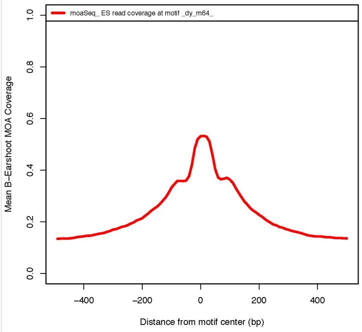	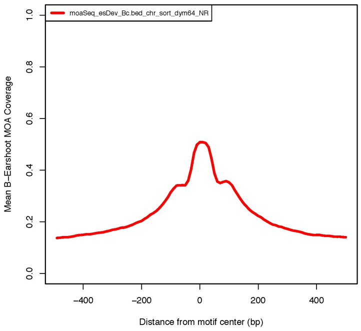	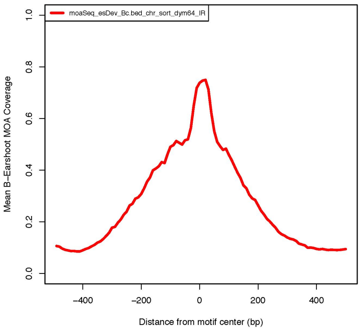

Motif Name	Total Number	In Repeats
dym65	1,404	17%
Consensus	vyACAsrmvyACAsv	
LOGO	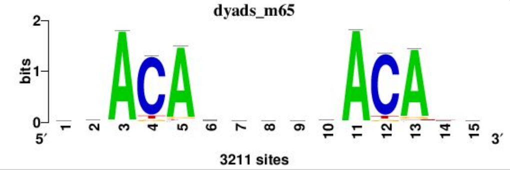	
LOGO RC	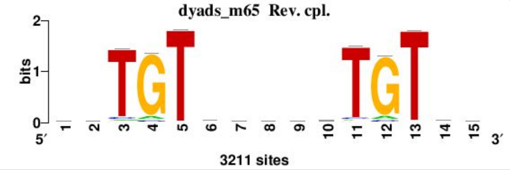	

	Frequency Distr. at TSS (FGS, B73v3)	Average Local Base Frequency (FGS, B73v3)
Median Position Relative to TSS = -30	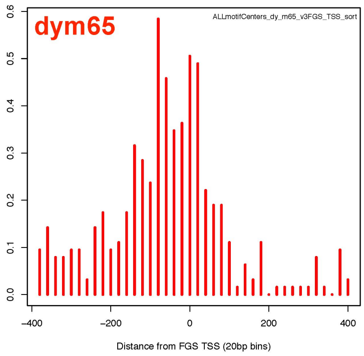	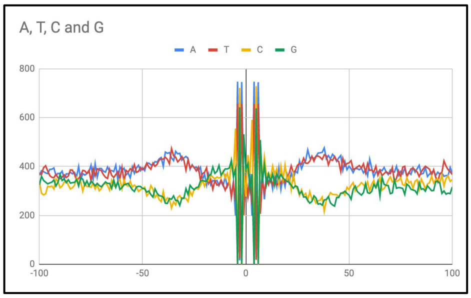

Average MOA Coverage Around Motif (RPM)

All	Not in Repeats (NR)	In Repeats (IR)
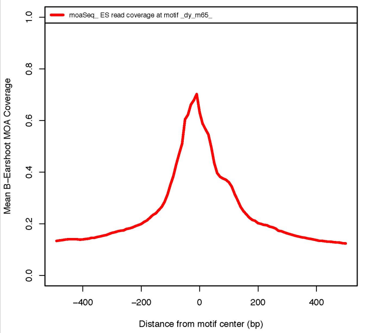	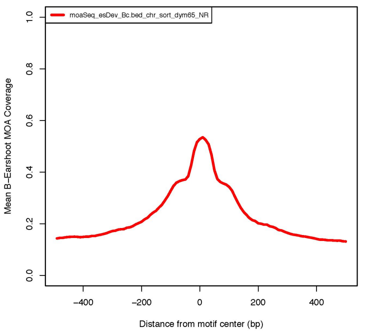	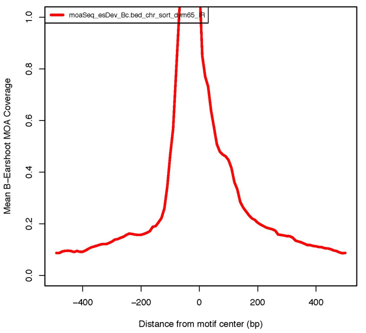

Motif Name	Total Number	In Repeats
dym66	2,269	9%
Consensus	grGgGrGAGAGGGAGGraGGaGrr	
LOGO	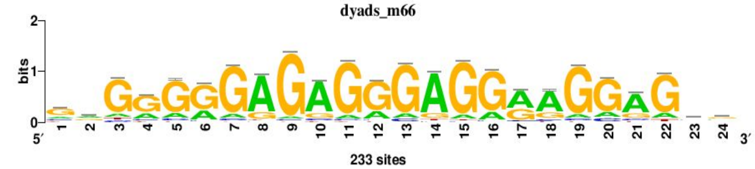	
LOGO RC	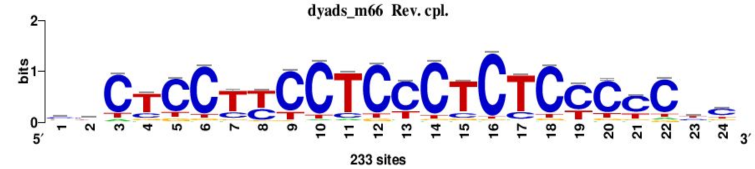	

	Frequency Distr. at TSS (FGS, B73v3)	Average Local Base Frequency (FGS, B73v3)
Median Position Relative to TSS =	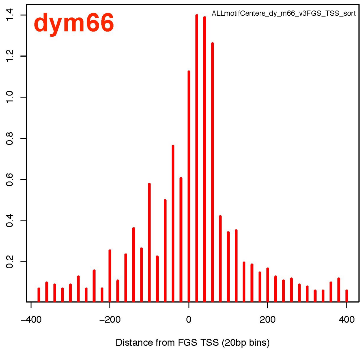	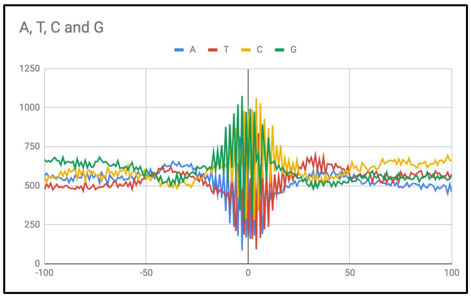
-6		

Average MOA Coverage Around Motif (RPM)

All	Not in Repeats (NR)	In Repeats (IR)
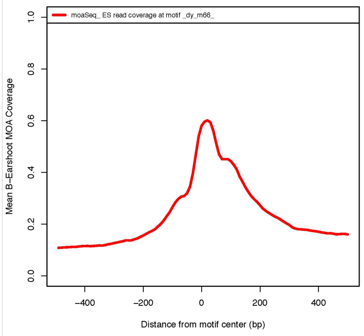	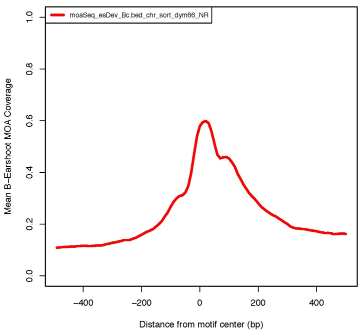	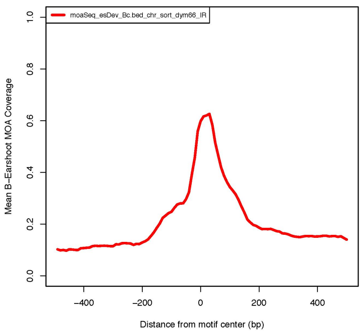

Motif Name	Total Number	In Repeats
dym67	1,222	8%
Consensus	ccCaCCCACCCcCCCACsCcc	
LOGO	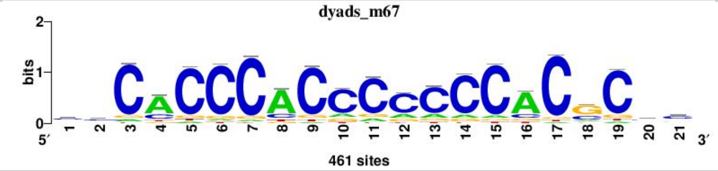	
LOGO RC	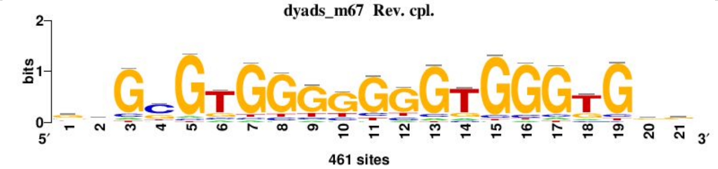	

	Frequency Distr. at TSS (FGS, B73v3)	Average Local Base Frequency (FGS, B73v3)
Median Position Relative to TSS = -21	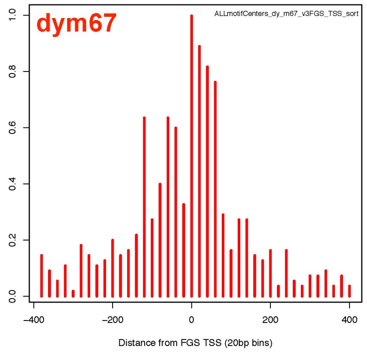	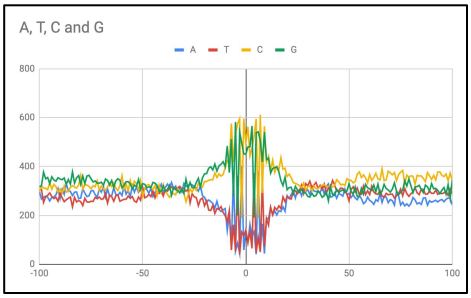

Average MOA Coverage Around Motif (RPM)

All	Not in Repeats (NR)	In Repeats (IR)
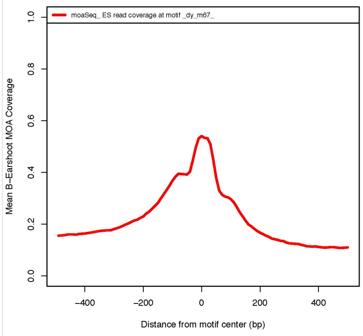	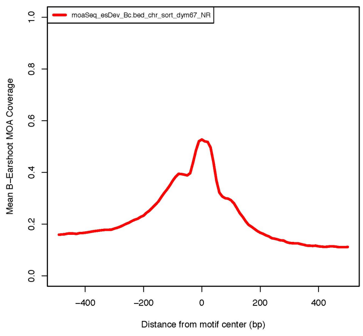	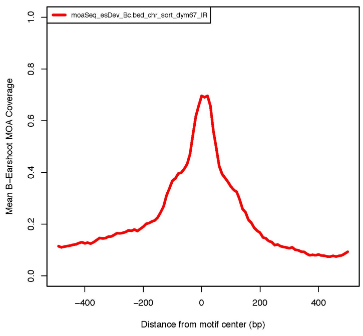

Motif Name	Total Number	In Repeats
dym68	2,515	9%
Consensus	csCACvsscCACss	
LOGO	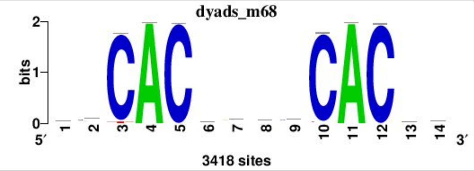	
LOGO RC	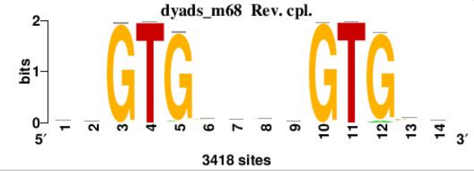	

	Frequency Distr. at TSS (FGS, B73v3)	Average Local Base Frequency (FGS, B73v3)
Median Position Relative to TSS =	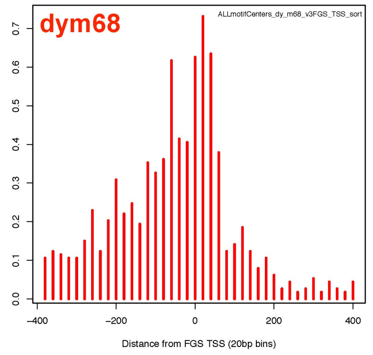	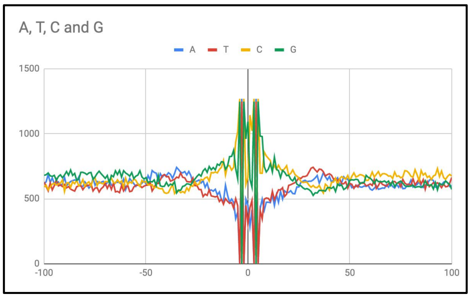
-74		

Average MOA Coverage Around Motif (RPM)

All	Not in Repeats (NR)	In Repeats (IR)
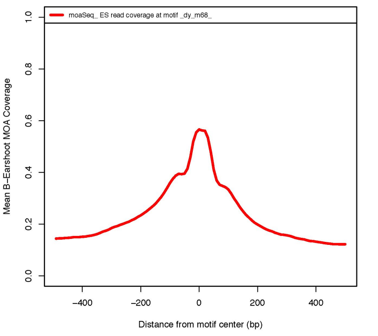	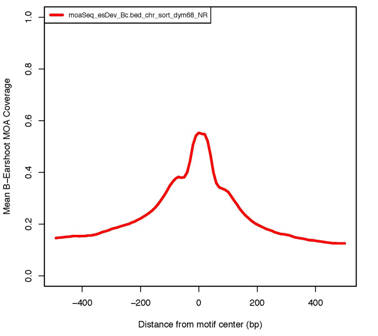	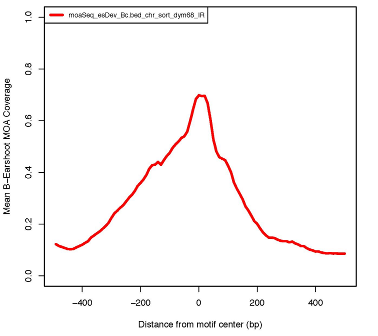

Motif Name	Total Number	In Repeats
**dym69**	**2,686**	**28%**
**Consensus**	watAArAAAAAAAwtAAAgwa	

LOGO

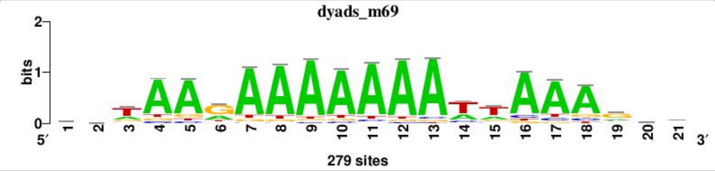

LOGO RC

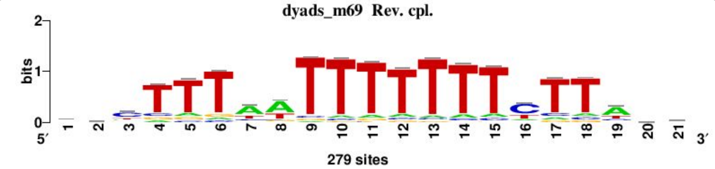

	Frequency Distr. at TSS (FGS, B73v3)	Average Local Base Frequency (FGS, B73v3)
**Median Position Relative to TSS =** **-89**		

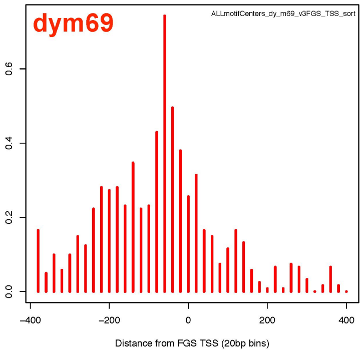

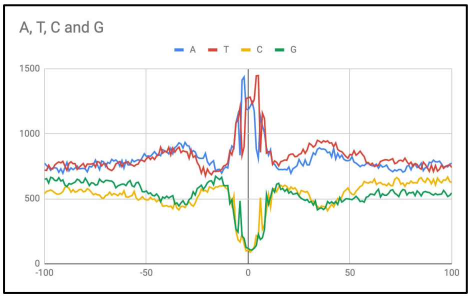

Average MOA Coverage Around Motif (RPM)

All	Not in Repeats (NR)	In Repeats (IR)

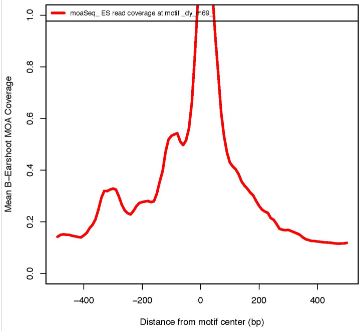

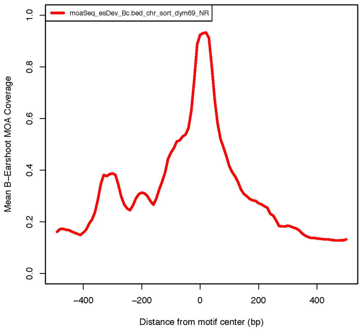

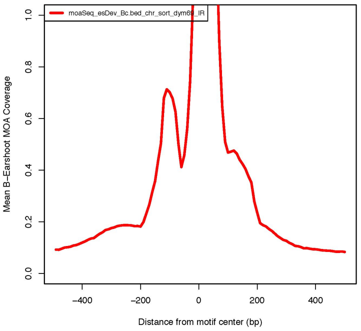

Motif Name	Total Number	In Repeats
dym70	1,234	32%
Consensus	rrGAAGAAATgr	
LOGO	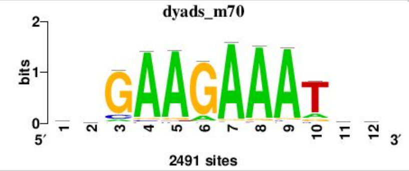	
LOGO RC	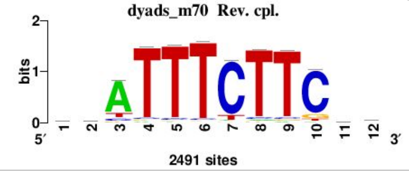	

	Frequency Distr. at TSS (FGS, B73v3)	Average Local Base Frequency (FGS, B73v3)
Median Position Relative to TSS =	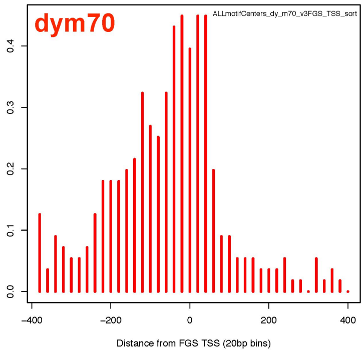	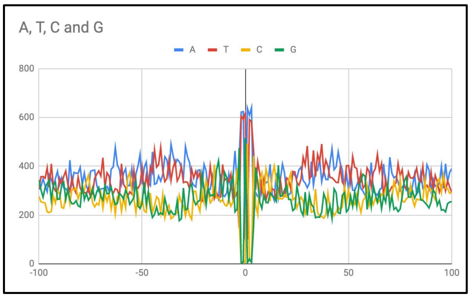
-39		

Average MOA Coverage Around Motif (RPM)

All	Not in Repeats (NR)	In Repeats (IR)
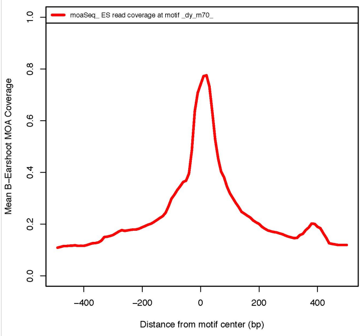	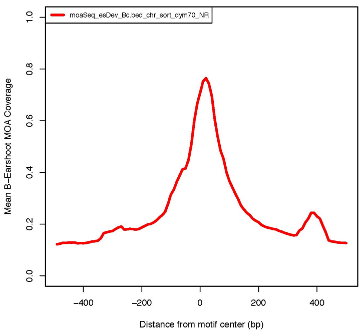	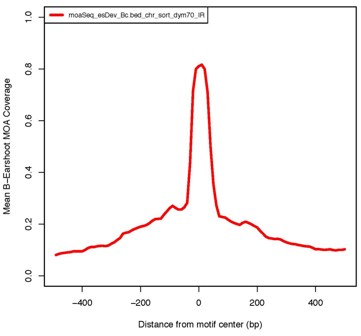

Motif Name	Total Number	In Repeats
dym71	2,797	7%
Consensus	ssCGCCGCss	

LOGO

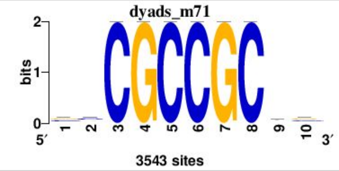

LOGO RC

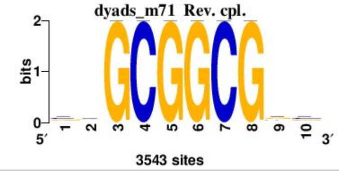

	Frequency Distr. at TSS (FGS, B73v3)	Average Local Base Frequency (FGS, B73v3)
Median Position Relative to TSS = -40		

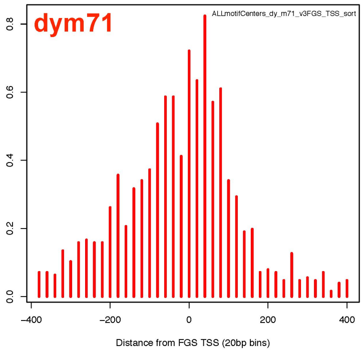

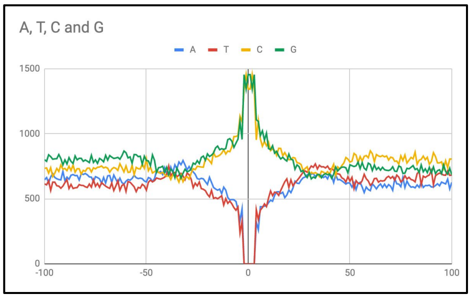

Average MOA Coverage Around Motif (RPM)

All	Not in Repeats (NR)	In Repeats (IR)

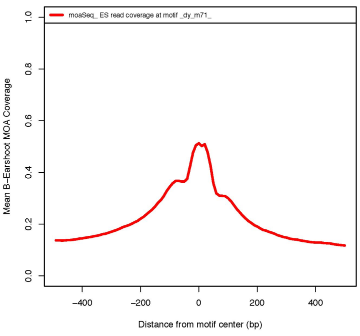

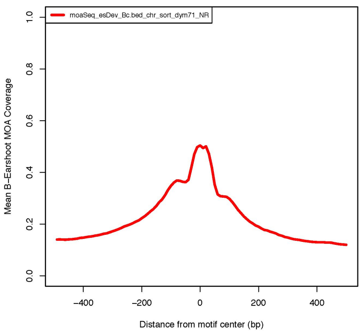

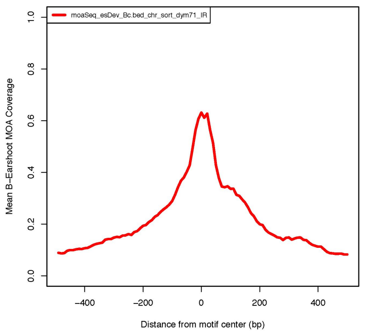

Motif Name	Total Number	In Repeats
dym72	1,931	13%
Consensus	gaGAGAGAGAGArArAGArArvrr	

LOGO

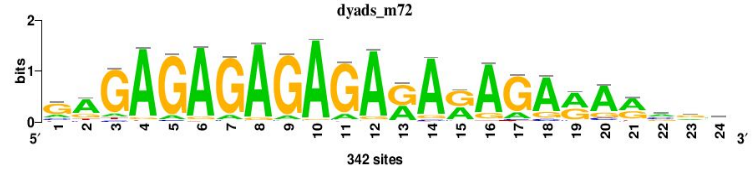

LOGO RC

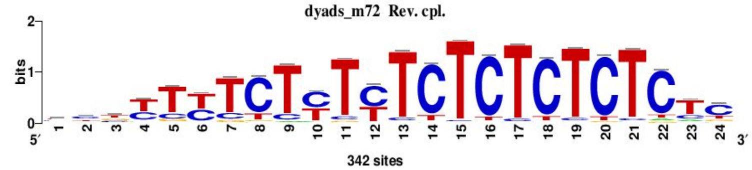

	Frequency Distr. at TSS (FGS, B73v3)	Average Local Base Frequency (FGS, B73v3)
Median Position Relative to TSS = -33	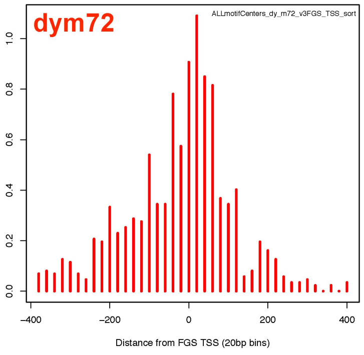	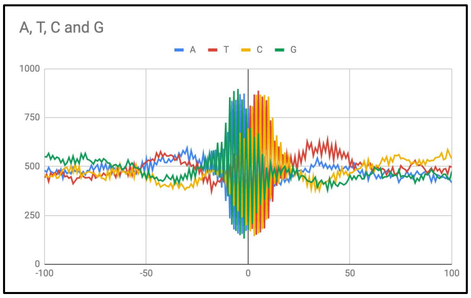

Average MOA Coverage Around Motif (RPM)

All	Not in Repeats (NR)	In Repeats (IR)
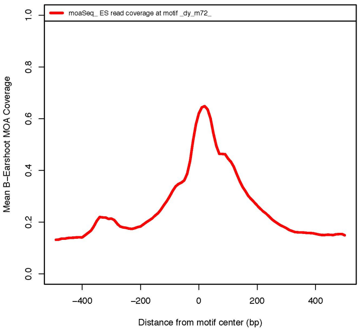	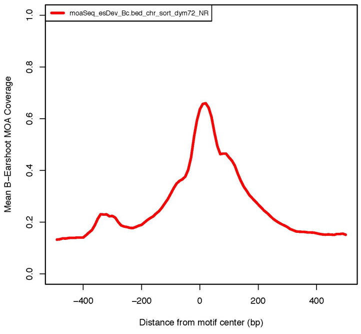	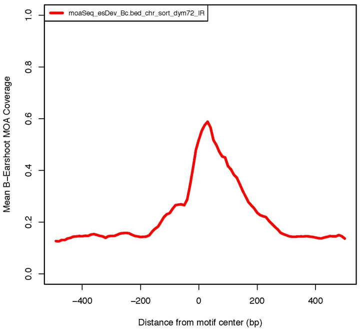

Motif Name	Total Number	In Repeats
**dym73**	**1,850**	**11%**

**Consensus**	agAGAGAGAGArAGArArAGAGrr
**LOGO**	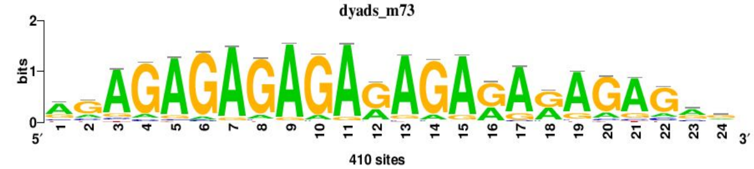
**LOGO RC**	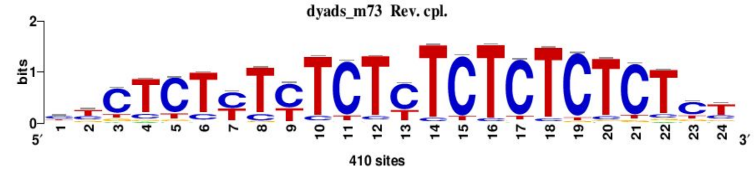

	Frequency Distr. at TSS (FGS, B73v3)	Average Local Base Frequency (FGS, B73v3)
**Median Position Relative to TSS =** **-27**	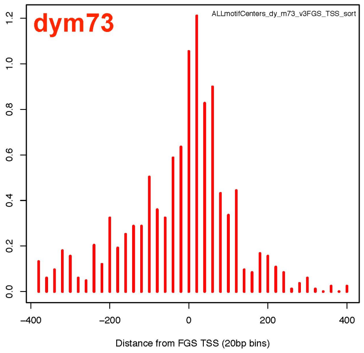	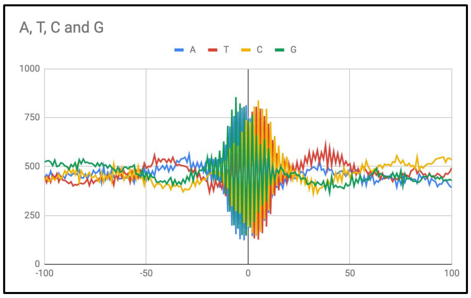

Average MOA Coverage Around Motif (RPM)

All	Not in Repeats (NR)	In Repeats (IR)
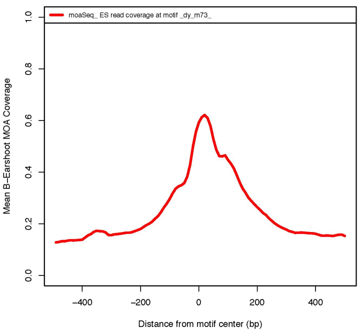	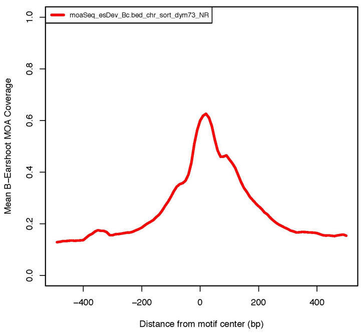	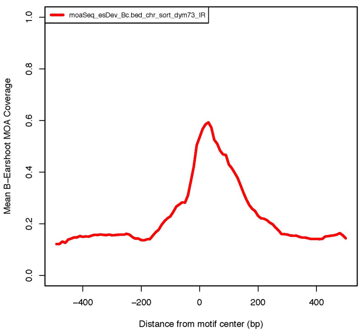

Motif Name	Total Number	In Repeats
dym74	1,494	20%
Consensus	rrGAArAAAAAArrAAAgGAGGsr	
LOGO	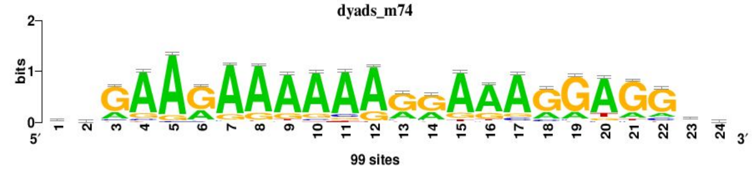	
LOGO RC	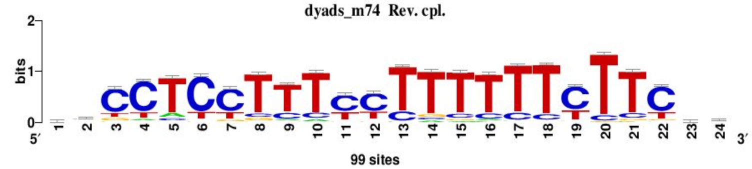	

	Frequency Distr. at TSS (FGS, B73v3)	Average Local Base Frequency (FGS, B73v3)
Median Position Relative to TSS =	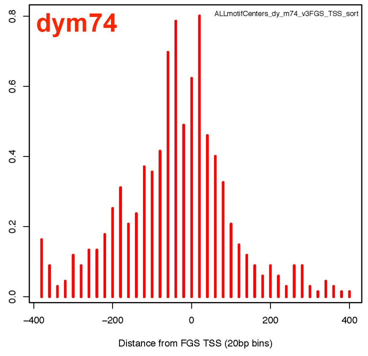	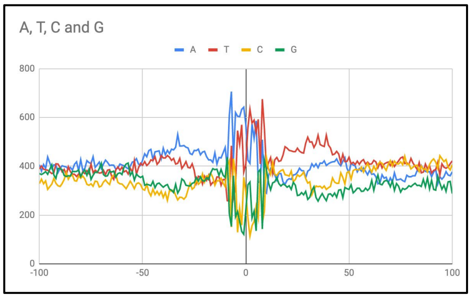
-48		

Average MOA Coverage Around Motif (RPM)

All	Not in Repeats (NR)	In Repeats (IR)
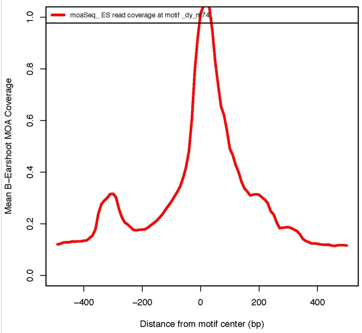	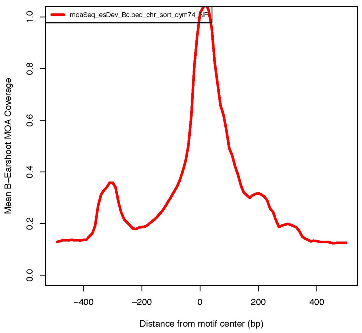	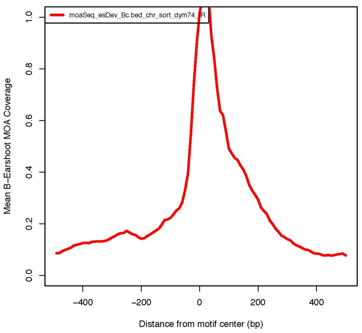

Motif Name	Total Number	In Repeats
dym75	1,753	17%
Consensus	scACCmscACCvs	
LOGO	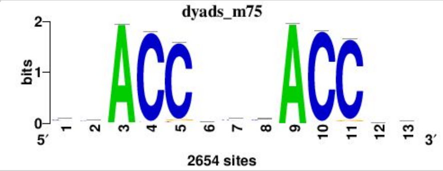	
LOGO RC	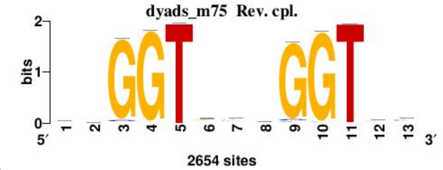	

	Frequency Distr. at TSS (FGS, B73v3)	Average Local Base Frequency (FGS, B73v3)
Median Position Relative to TSS =	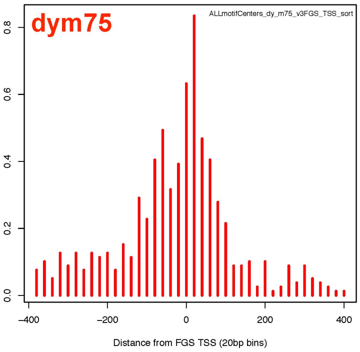	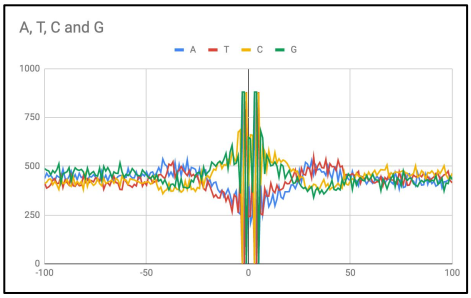
-35		

Average MOA Coverage Around Motif (RPM)

All	Not in Repeats (NR)	In Repeats (IR)
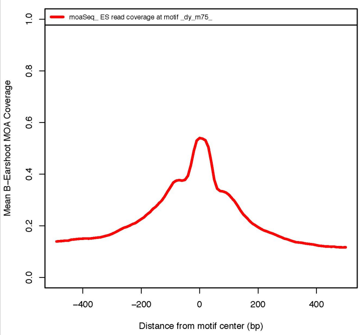	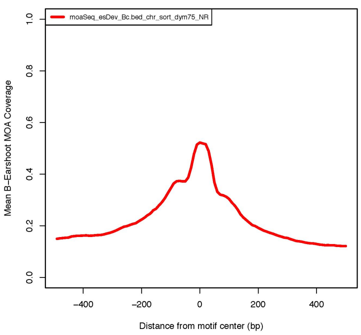	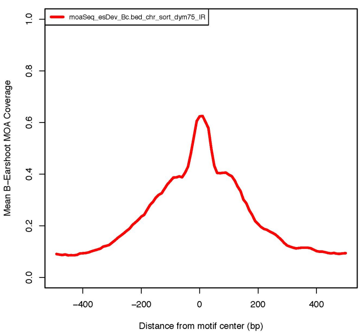
