# Supplementary material for: The native cistrome and sequence motif families of the maize ear
Source: PLoS Genet. 2021 Aug 12;17(8):e1009689. doi: 10.1371/journal.pgen.1009689 (PMC8360572; doi:10.1371/journal.pgen.1009689)
Supplement: S7 File — This PDF file provides a reference document, one page per motif, listing the assigned RSAT-oligo motif name (e.g. om001), total number of sites in B73v3, percentage found in annotated repeats, the consensus and sequence LOGOs from RSAT reports, the median TSS-relative position and motif frequency histograms around the TSSs of the filtered gene set (FGS), the local base count composition flanking the motif midpoints, and average local MOA-seq coverage centered on the motifs for all (All) motifs, or those motifs split into either not overlapping/in repeats (NR) or overlapping/in repeats (IR). (PDF) [file pgen.1009689.s014.pdf]

Motif Name	Total Number	In Repeats
om001	660	8%
Consensus	srCAGGGCAss	
LOGO	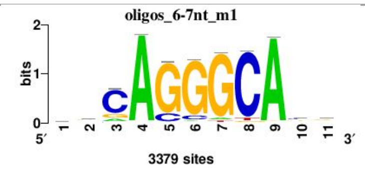	
LOGO RC	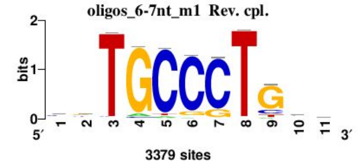	

	Frequency Distr. at TSS (FGS, B73v3)	Average Local Base Frequency (FGS, B73v3)
Median Position Relative to TSS = -25	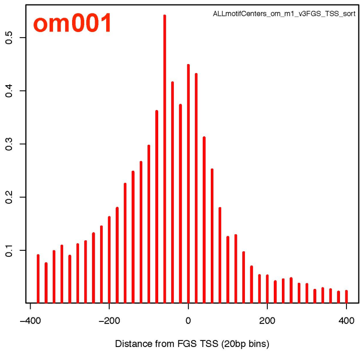	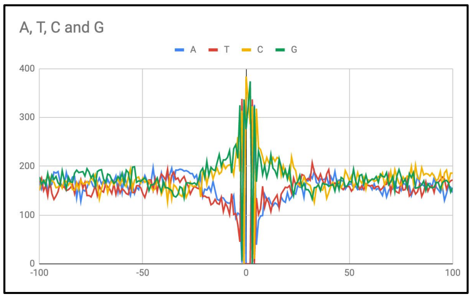

Average MOA Coverage Around Motif (RPM)

All	Not in Repeats (NR)	In Repeats (IR)
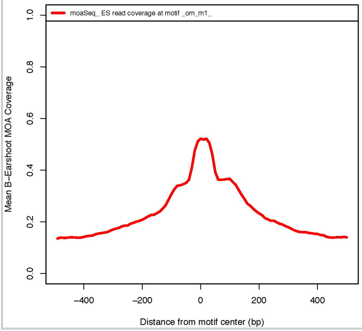	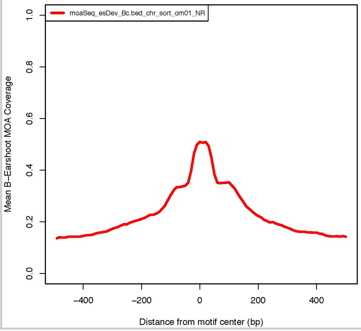	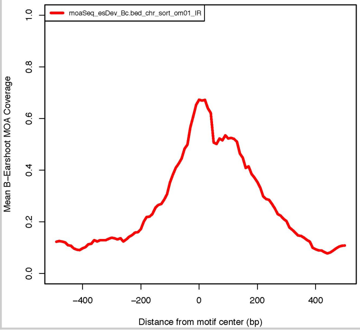

Motif Name	Total Number	In Repeats
om002	3,067	15%
Consensus	scAGCCCAgcm	
LOGO	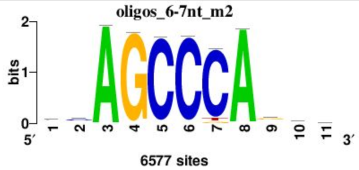	
LOGO RC	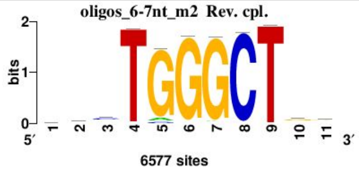	

	Frequency Distr. at TSS (FGS, B73v3)	Average Local Base Frequency (FGS, B73v3)
Median Position Relative to TSS =	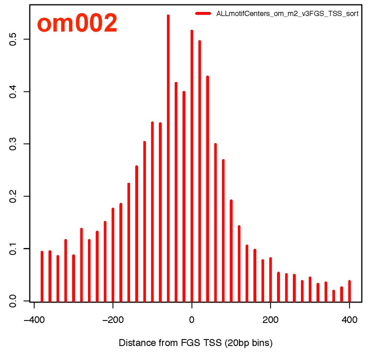	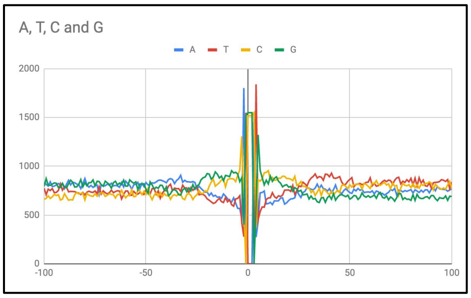
-82		

Average MOA Coverage Around Motif (RPM)

All	Not in Repeats (NR)	In Repeats (IR)
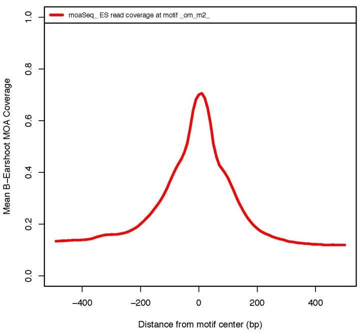	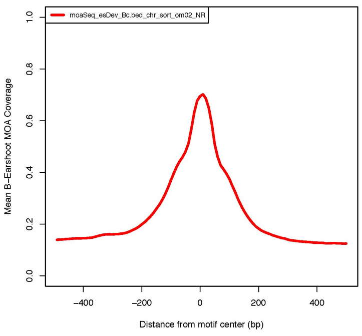	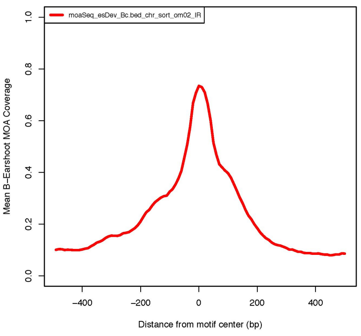

Motif Name	Total Number	In Repeats
om003	3,031	11%

Consensus	ssCCCCACcc
LOGO	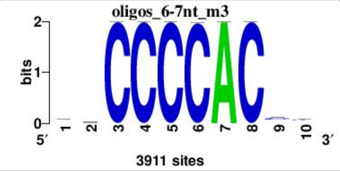
LOGO RC	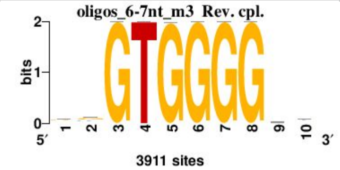

	Frequency Distr. at TSS (FGS, B73v3)	Average Local Base Frequency (FGS, B73v3)
Median Position Relative to TSS =	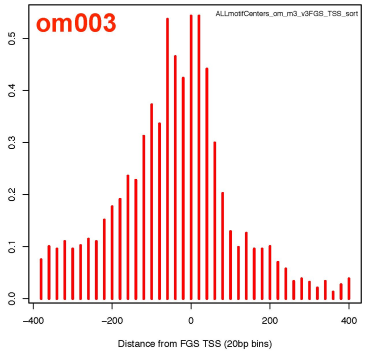	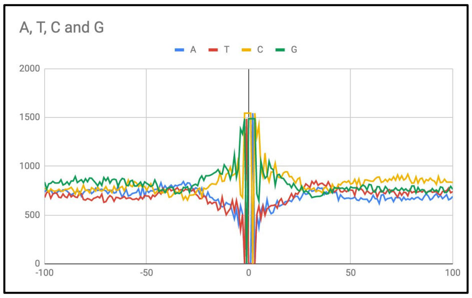
-62		

Average MOA Coverage Around Motif (RPM)

All	Not in Repeats (NR)	In Repeats (IR)
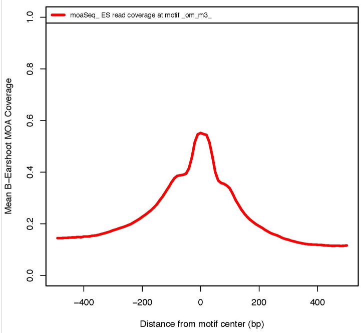	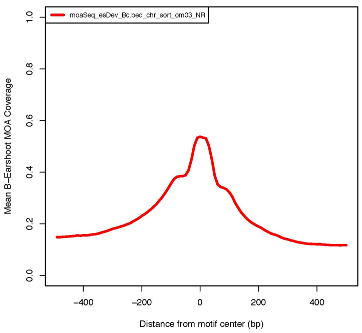	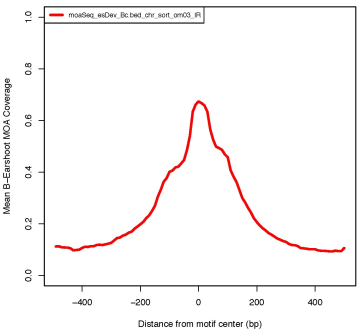

Motif Name	Total Number	In Repeats
om004	2,471	31%
Consensus	scCGGCCCAgsy	
LOGO	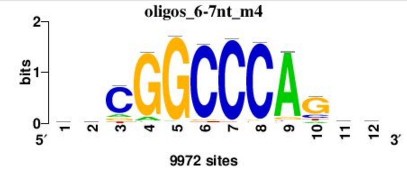	
LOGO RC	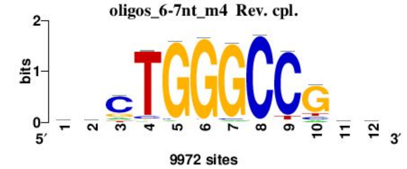	

	Frequency Distr. at TSS (FGS, B73v3)	Average Local Base Frequency (FGS, B73v3)
Median Position Relative to TSS =	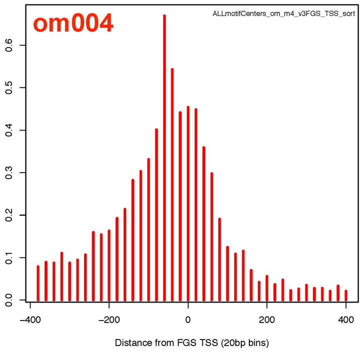	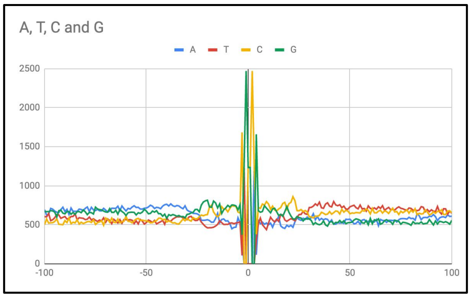
-87		

Average MOA Coverage Around Motif (RPM)		
All	Not in Repeats (NR)	In Repeats (IR)
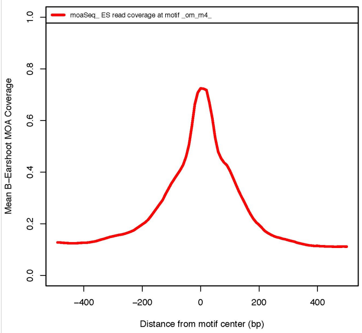	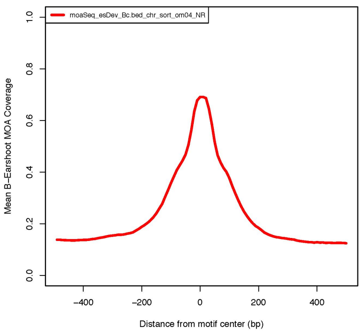	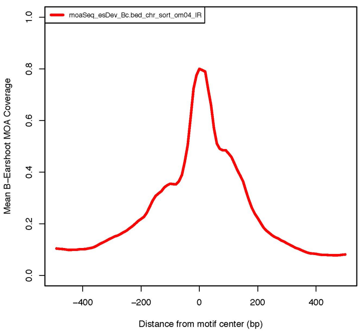

Motif Name	Total Number	In Repeats
om005	824	56%
Consensus	ssATCGGATsg	
LOGO	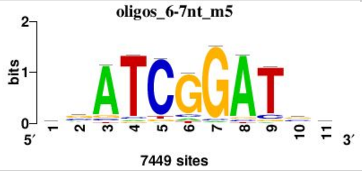	
LOGO RC	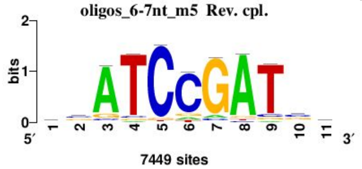	

	Frequency Distr. at TSS (FGS, B73v3)	Average Local Base Frequency (FGS, B73v3)
Median Position Relative to TSS =	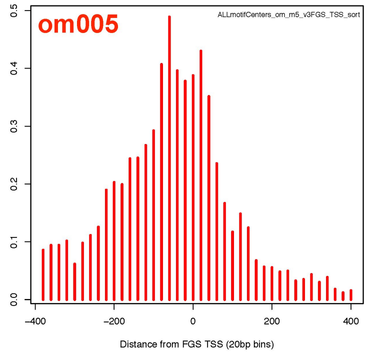	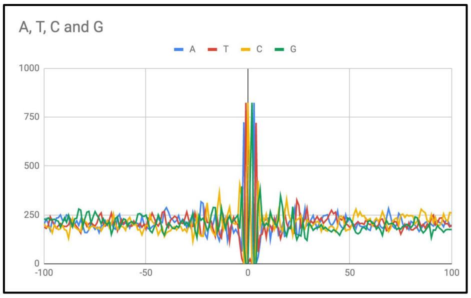
-87		

Average MOA Coverage Around Motif (RPM)

All	Not in Repeats (NR)	In Repeats (IR)
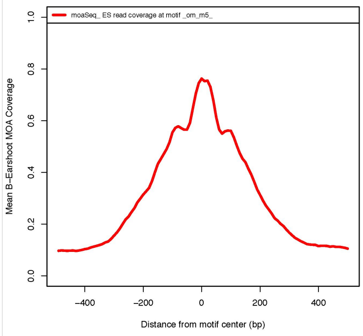	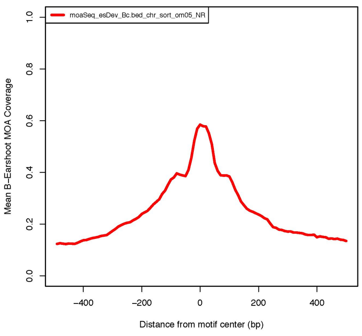	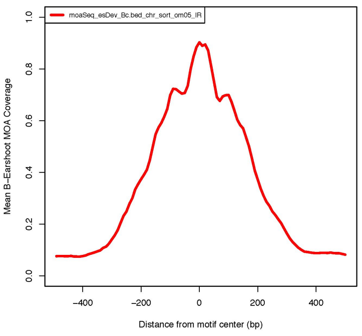

Motif Name	Total Number	In Repeats
om006	723	39%
Consensus	ymAACCCTAsc	

LOGO

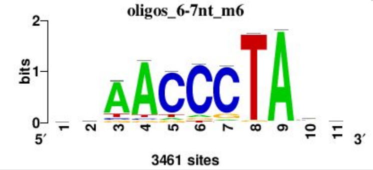

LOGO RC

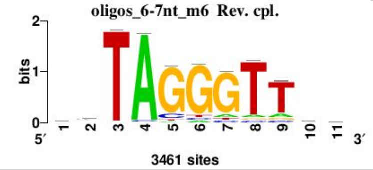

	Frequency Distr. at TSS (FGS, B73v3)	Average Local Base Frequency (FGS, B73v3)
Median Position Relative to TSS = -3	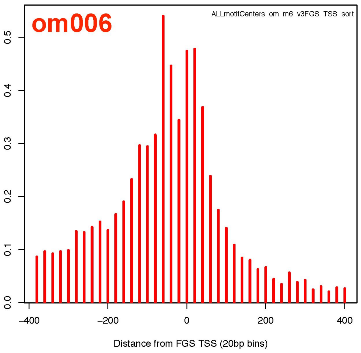	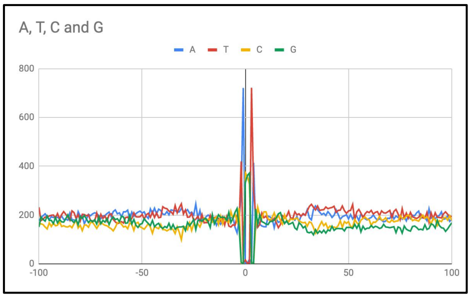

Average MOA Coverage Around Motif (RPM)

All	Not in Repeats (NR)	In Repeats (IR)
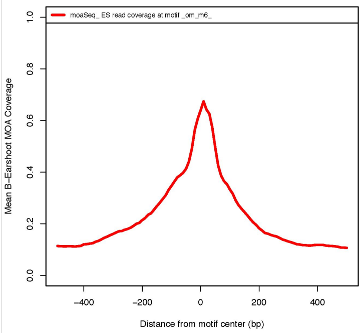	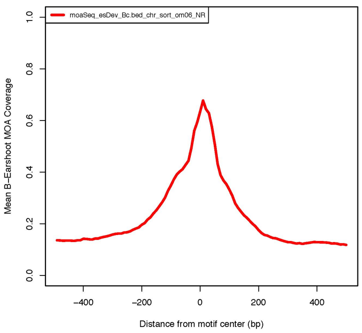	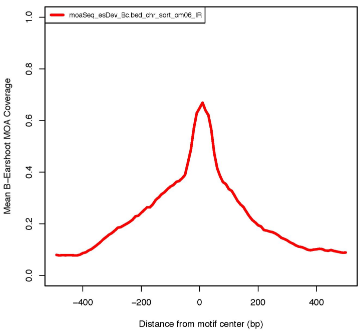

Motif Name	Total Number	In Repeats
om007	1,814	13%
Consensus	sccCGCCCGcs	
LOGO	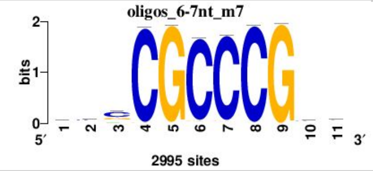	
LOGO RC	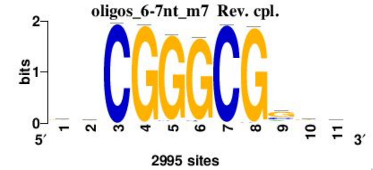	

	Frequency Distr. at TSS (FGS, B73v3)	Average Local Base Frequency (FGS, B73v3)
Median Position Relative to TSS = -87	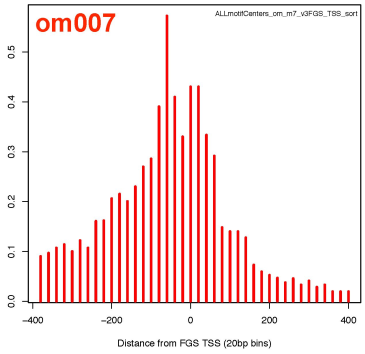	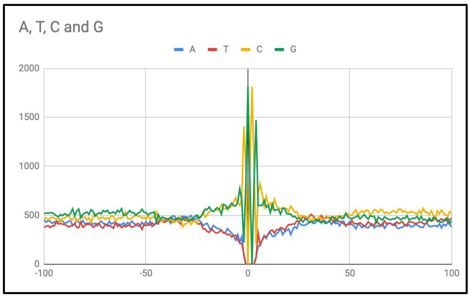

Average MOA Coverage Around Motif (RPM)		
All	Not in Repeats (NR)	In Repeats (IR)
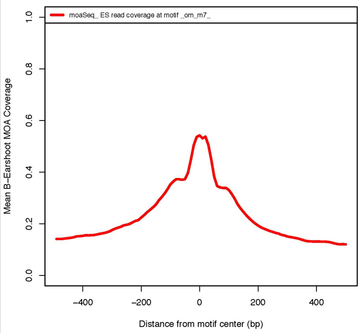	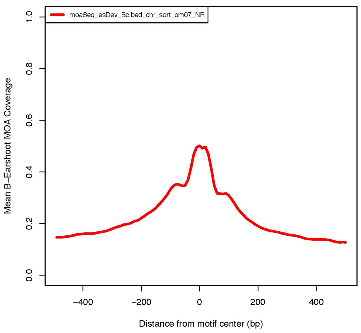	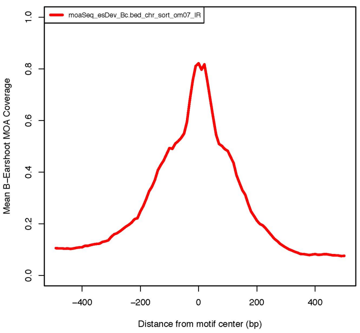

Motif Name	Total Number	In Repeats
om008	1,358	54%
Consensus	csATCCAAtcsc	
LOGO	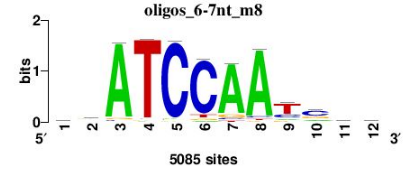	
LOGO RC	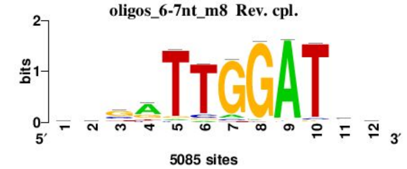	

	Frequency Distr. at TSS (FGS, B73v3)	Average Local Base Frequency (FGS, B73v3)
Median Position Relative to TSS =	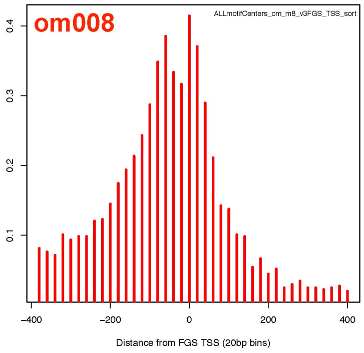	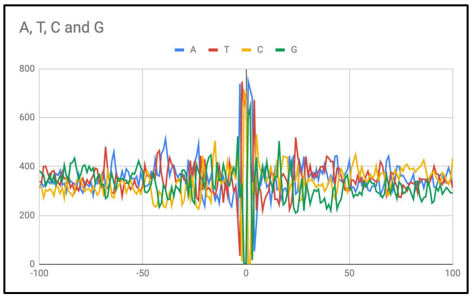
-78		

Average MOA Coverage Around Motif (RPM)

All	Not in Repeats (NR)	In Repeats (IR)
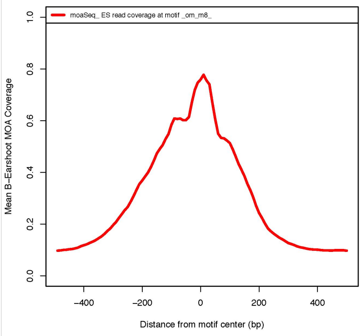	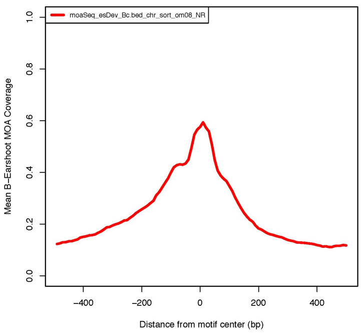	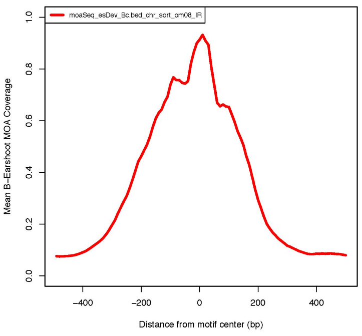

Motif Name	Total Number	In Repeats
**om009**	**395**	**17%**
**Consensus**	ccCATCTCAbc	
**LOGO**	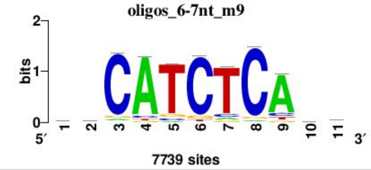	
**LOGO RC**	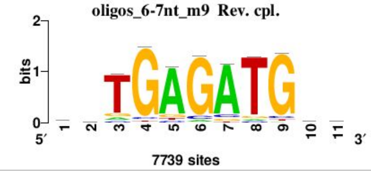	

	Frequency Distr. at TSS (FGS, B73v3)	Average Local Base Frequency (FGS, B73v3)
**Median Position Relative to TSS =**	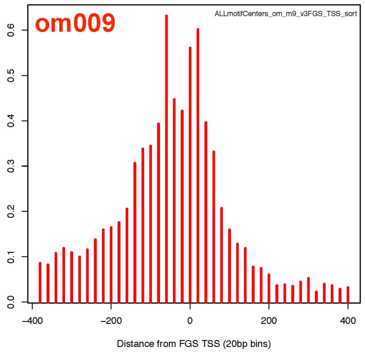	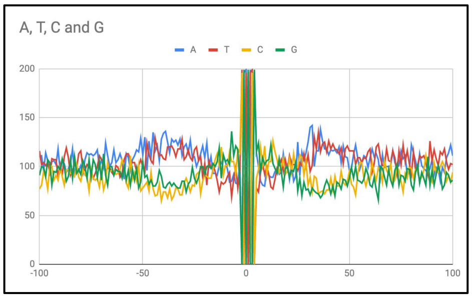
**-72**		

Average MOA Coverage Around Motif (RPM)

All	Not in Repeats (NR)	In Repeats (IR)
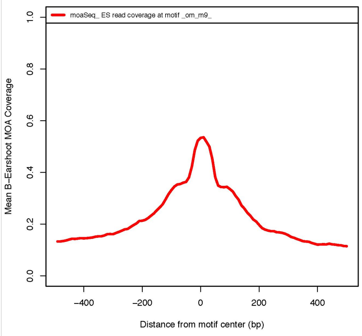	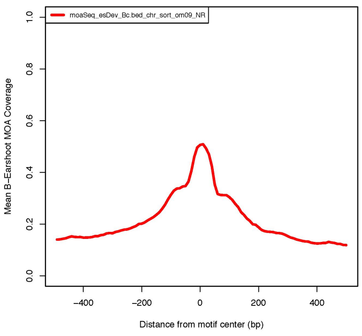	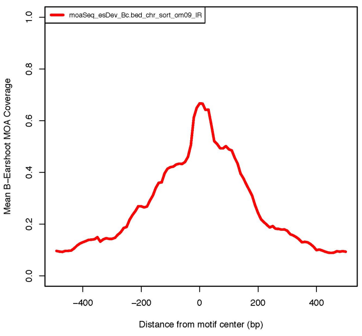

Motif Name	Total Number	In Repeats
om010	1,203	25%

Consensus ssCCGTCCGwy

LOGO

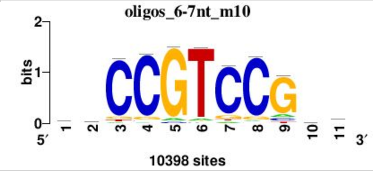

LOGO RC

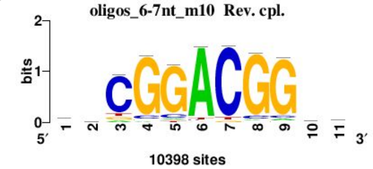

	Frequency Distr. at TSS (FGS, B73v3)	Average Local Base Frequency (FGS, B73v3)
Median Position Relative to TSS = -84	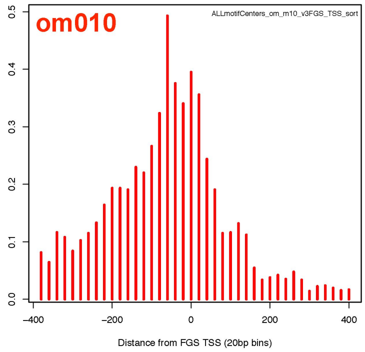	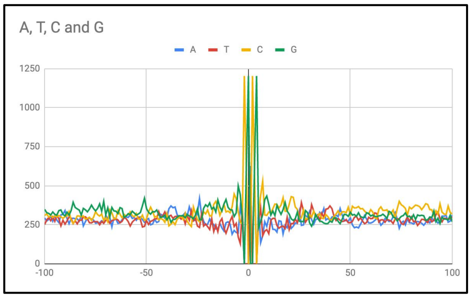

Average MOA Coverage Around Motif (RPM)

All	Not in Repeats (NR)	In Repeats (IR)
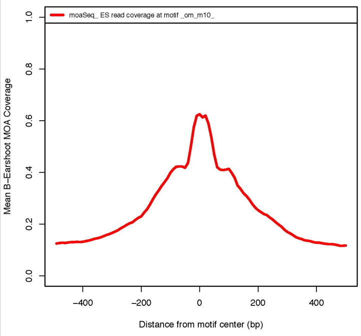	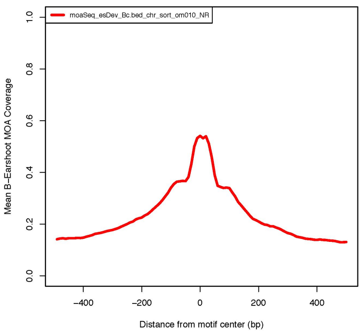	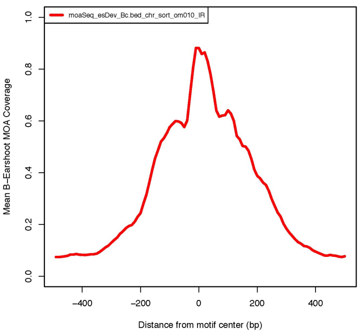

Motif Name	Total Number	In Repeats
om011	1,430	7%
Consensus	cscCGCCCCss	
LOGO	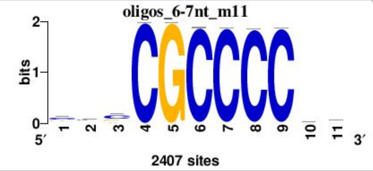	
LOGO RC	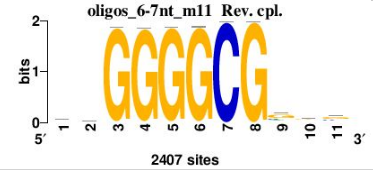	

	Frequency Distr. at TSS (FGS, B73v3)	Average Local Base Frequency (FGS, B73v3)
Median Position Relative to TSS =	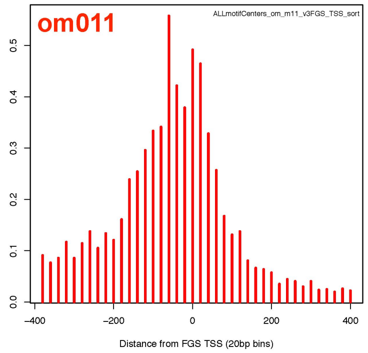	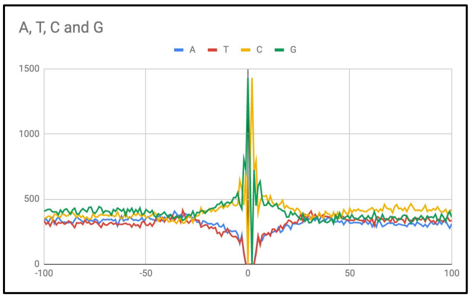
-46		

Average MOA Coverage Around Motif (RPM)

All	Not in Repeats (NR)	In Repeats (IR)
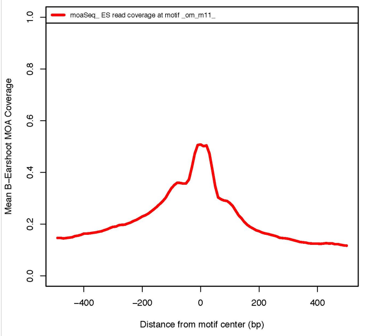	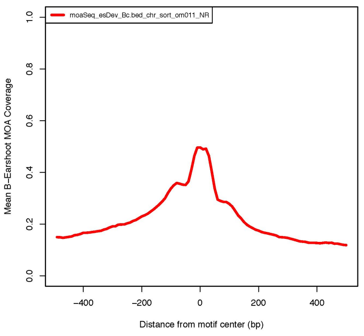	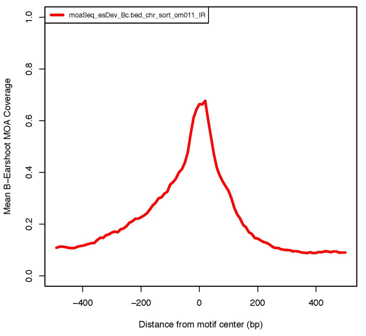

Motif Name	Total Number	In Repeats
om012	930	10%
Consensus	ssTCGCCTCGys	

LOGO

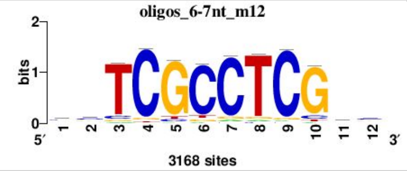

LOGO RC

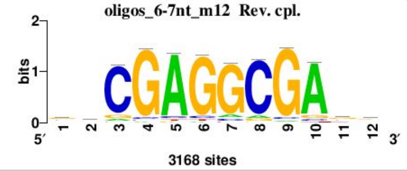

	Frequency Distr. at TSS (FGS, B73v3)	Average Local Base Frequency (FGS, B73v3)
Median Position Relative to TSS = -7	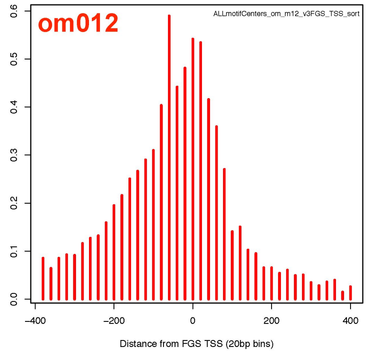	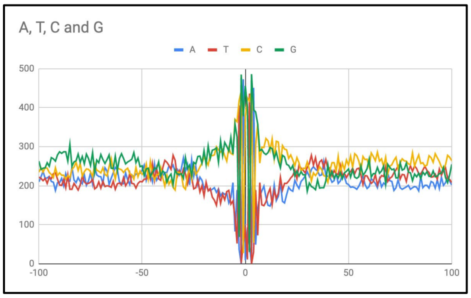

Average MOA Coverage Around Motif (RPM)

All	Not in Repeats (NR)	In Repeats (IR)
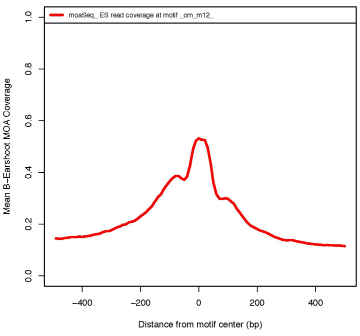	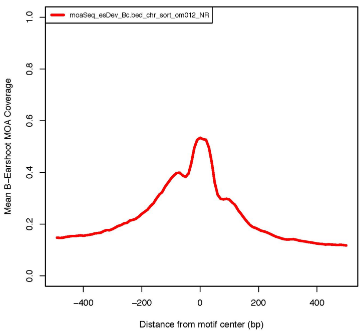	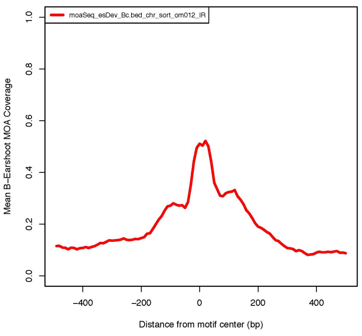

Motif Name	Total Number	In Repeats
om013	1,627	23%
Consensus	mmAACCAAmm	
LOGO	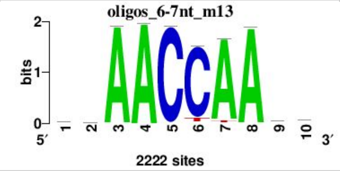	
LOGO RC	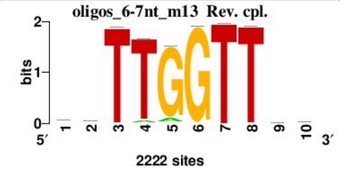	

	Frequency Distr. at TSS (FGS, B73v3)	Average Local Base Frequency (FGS, B73v3)
Median Position Relative to TSS =	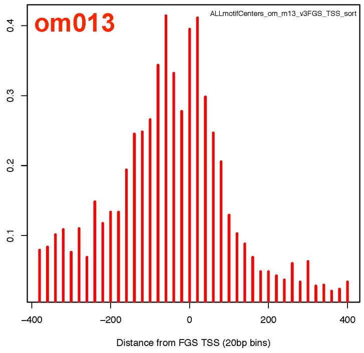	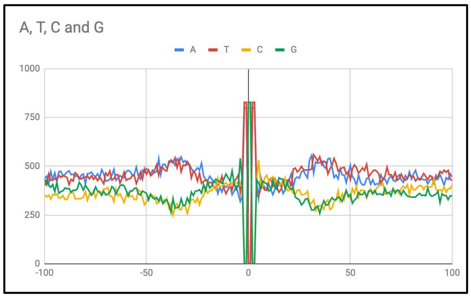
-47		

Average MOA Coverage Around Motif (RPM)

All	Not in Repeats (NR)	In Repeats (IR)
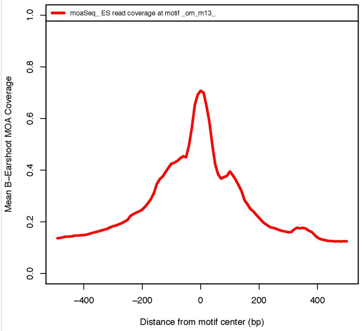	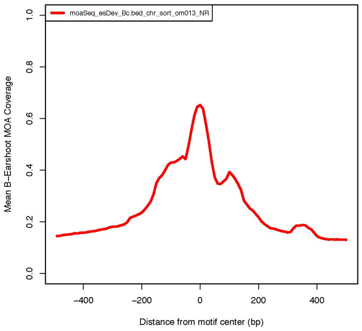	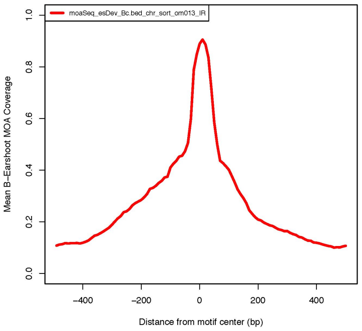

Motif Name	Total Number	In Repeats
om014	979	4%
Consensus	srGCAGAGCAgv	
LOGO	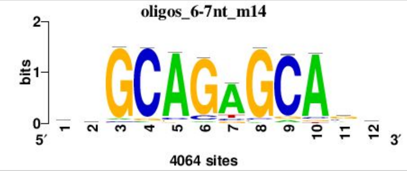	
LOGO RC	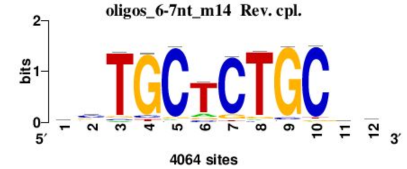	

	Frequency Distr. at TSS (FGS, B73v3)	Average Local Base Frequency (FGS, B73v3)
Median Position Relative to TSS =	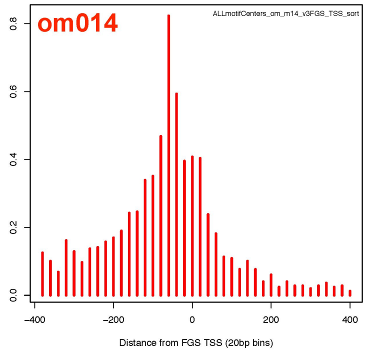	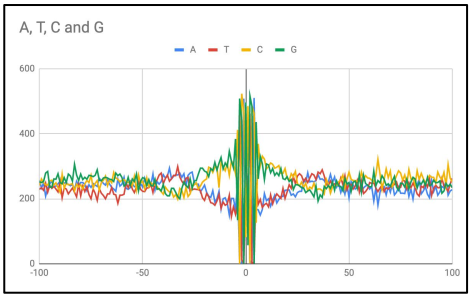
-75		

Average MOA Coverage Around Motif (RPM)

All	Not in Repeats (NR)	In Repeats (IR)
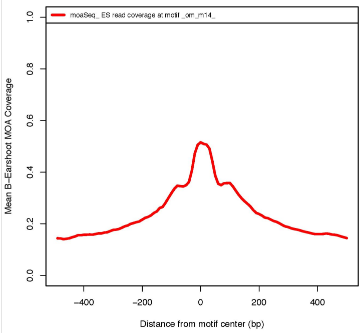	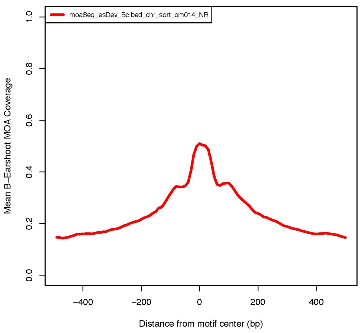	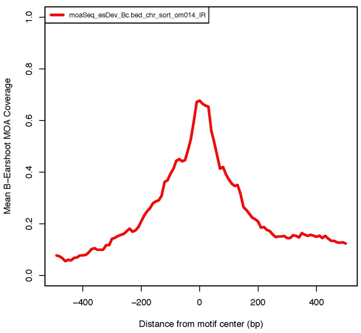

Motif Name	Total Number	In Repeats
om015	724	7%
Consensus	sstCACGTCAss	
LOGO	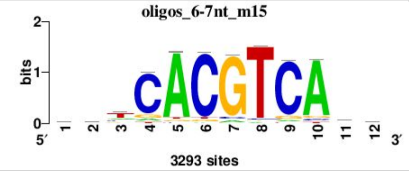	
LOGO RC	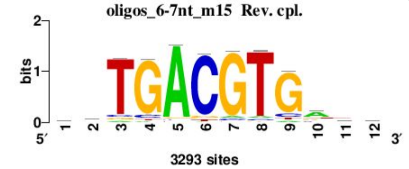	

	Frequency Distr. at TSS (FGS, B73v3)	Average Local Base Frequency (FGS, B73v3)
Median Position Relative to TSS =	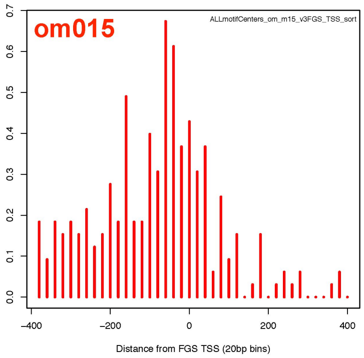	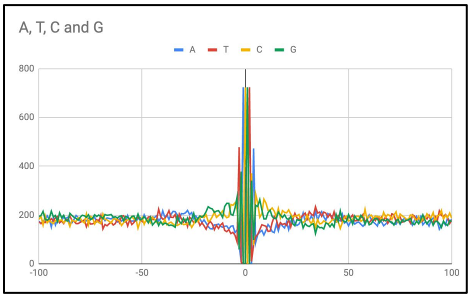
-121		

Average MOA Coverage Around Motif (RPM)

All	Not in Repeats (NR)	In Repeats (IR)
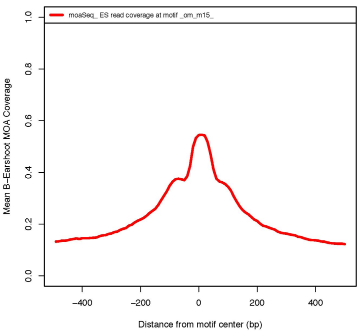	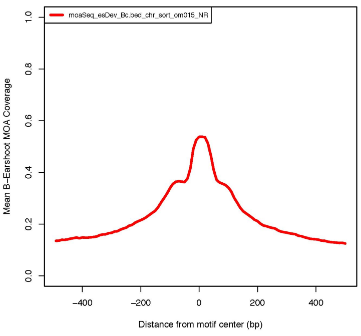	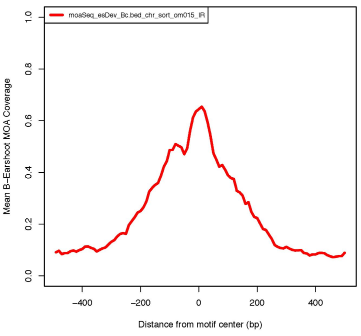

Motif Name	Total Number	In Repeats
om016	1,245	6%
Consensus	srGGGGCAgs	
LOGO	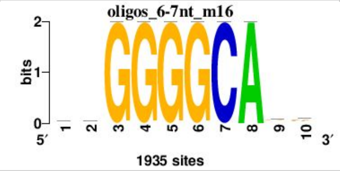	
LOGO RC	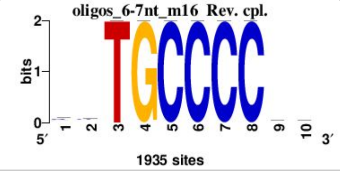	

	Frequency Distr. at TSS (FGS, B73v3)	Average Local Base Frequency (FGS, B73v3)
Median Position Relative to TSS =	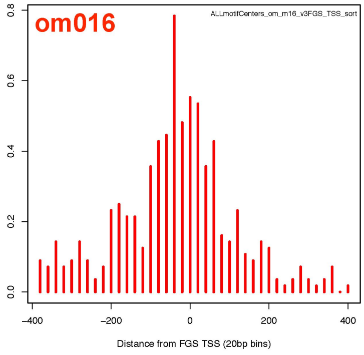	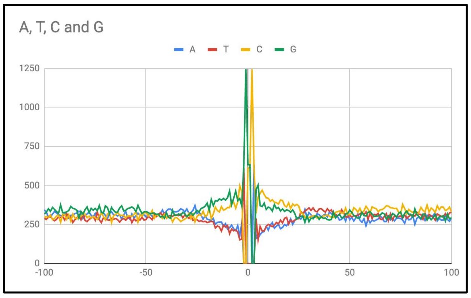
-55		

Average MOA Coverage Around Motif (RPM)

All	Not in Repeats (NR)	In Repeats (IR)
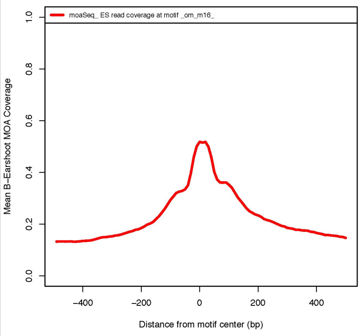	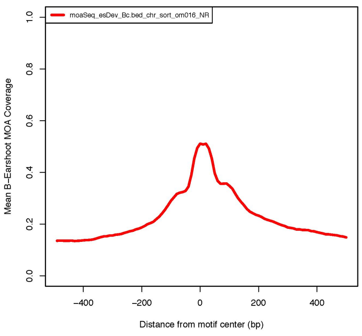	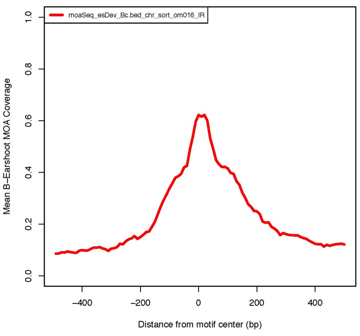

Motif Name	Total Number	In Repeats
om017	2,568	35%
Consensus	twTATTTTAtw	
LOGO	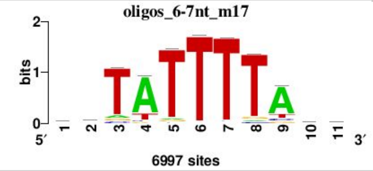	
LOGO RC	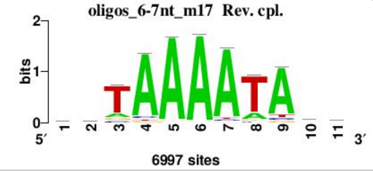	

	Frequency Distr. at TSS (FGS, B73v3)	Average Local Base Frequency (FGS, B73v3)
Median Position Relative to TSS =	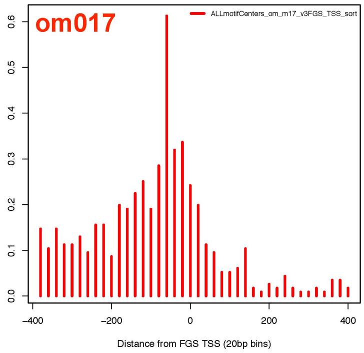	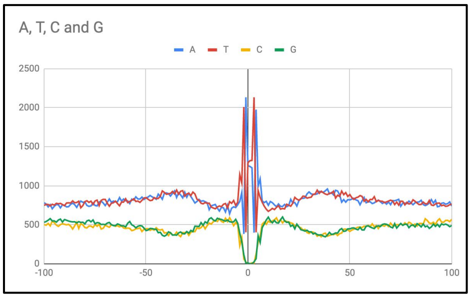
-91		

Average MOA Coverage Around Motif (RPM)

All	Not in Repeats (NR)	In Repeats (IR)
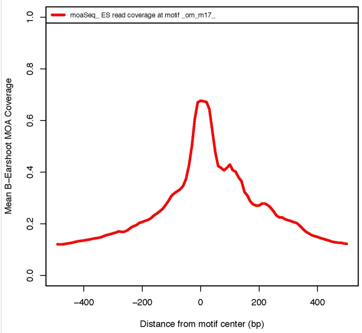	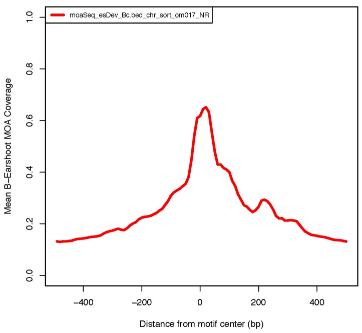	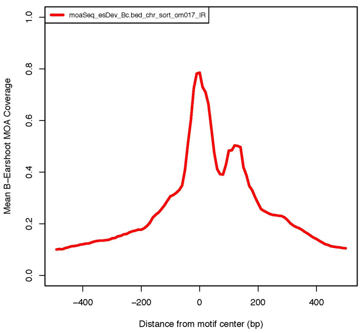

Motif Name	Total Number	In Repeats
om018	526	44%
Consensus	ssATCTGATsb	
LOGO	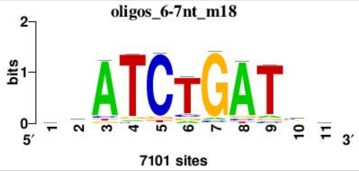	
LOGO RC	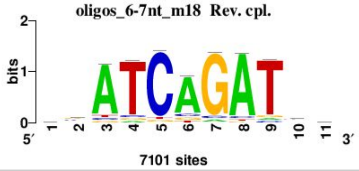	

	Frequency Distr. at TSS (FGS, B73v3)	Average Local Base Frequency (FGS, B73v3)
Median Position Relative to TSS =	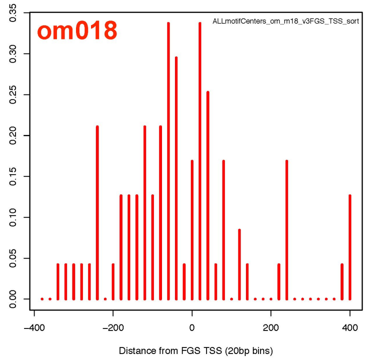	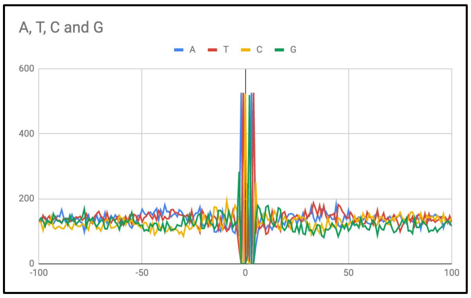
-47		

Average MOA Coverage Around Motif (RPM)

All	Not in Repeats (NR)	In Repeats (IR)
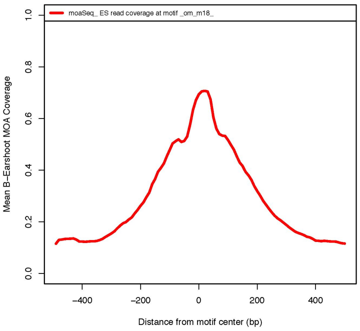	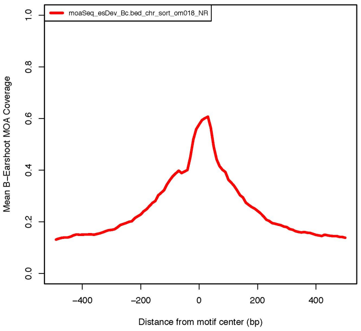	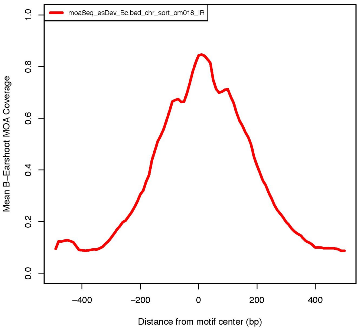

Motif Name	Total Number	In Repeats
om019	1,445	23%
Consensus	araACAAAACaa	
LOGO	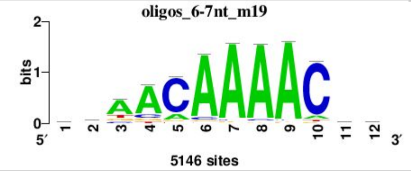	
LOGO RC	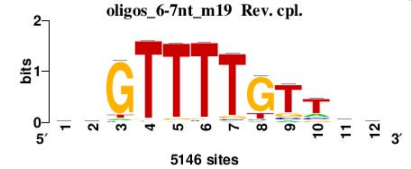	

	Frequency Distr. at TSS (FGS, B73v3)	Average Local Base Frequency (FGS, B73v3)
Median Position Relative to TSS =	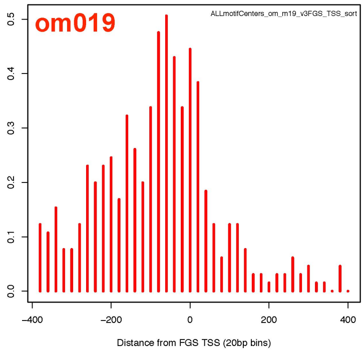	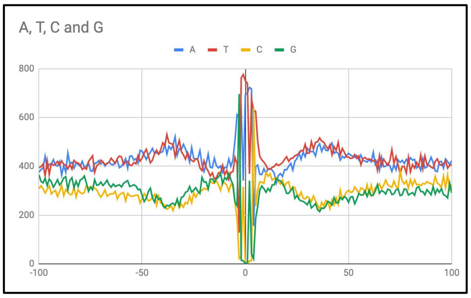
-83		

Average MOA Coverage Around Motif (RPM)

All	Not in Repeats (NR)	In Repeats (IR)
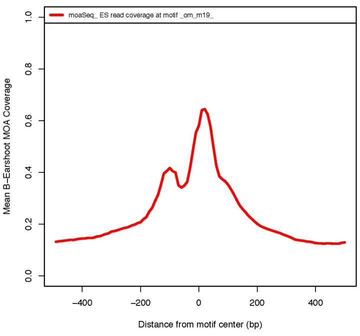	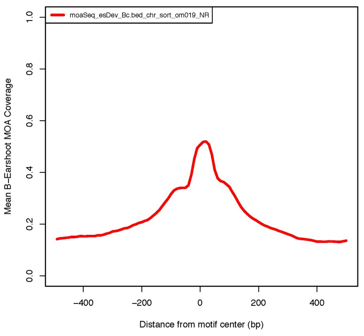	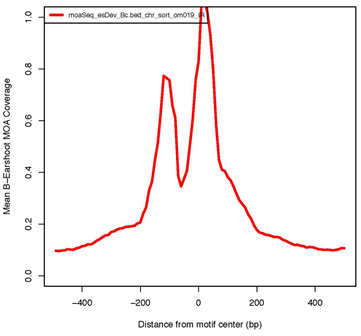

Motif Name	Total Number	In Repeats
om020	355	44%
Consensus	ycTATCCTAss	

LOGO

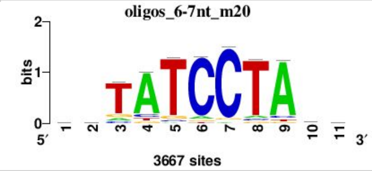

LOGO RC

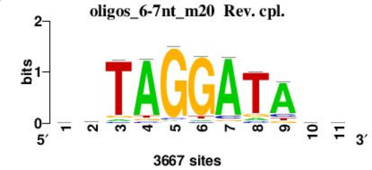

	Frequency Distr. at TSS (FGS, B73v3)	Average Local Base Frequency (FGS, B73v3)
Median Position Relative to TSS = -21	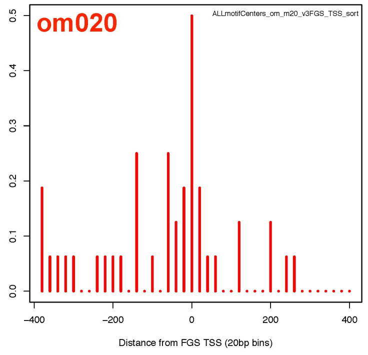	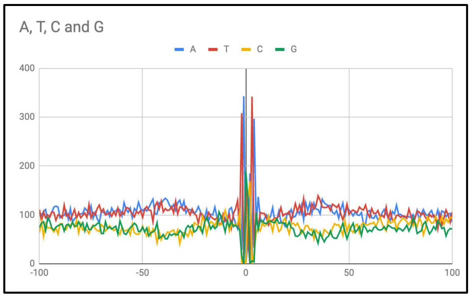

Average MOA Coverage Around Motif (RPM)

All	Not in Repeats (NR)	In Repeats (IR)
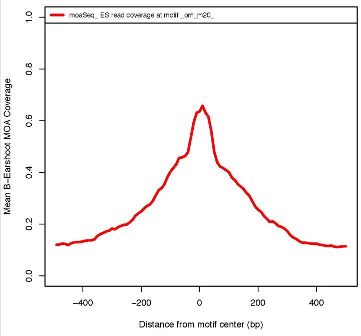	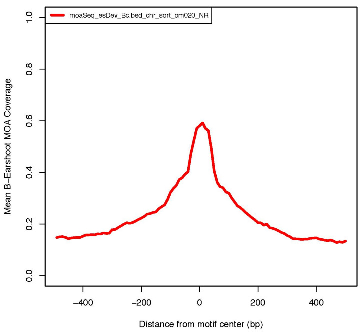	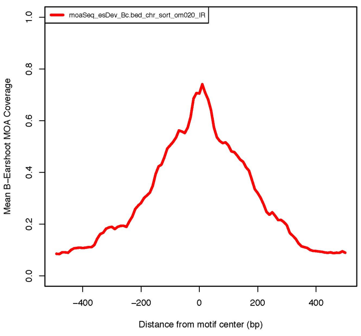

Motif Name	Total Number	In Repeats
om021	1,783	17%
Consensus	rrAAGGAArr	
LOGO	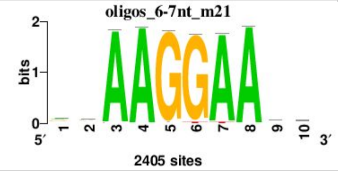	
LOGO RC	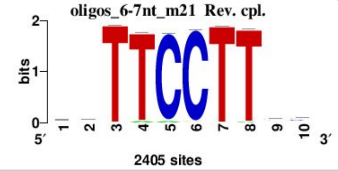	

	Frequency Distr. at TSS (FGS, B73v3)	Average Local Base Frequency (FGS, B73v3)
Median Position Relative to TSS = -61	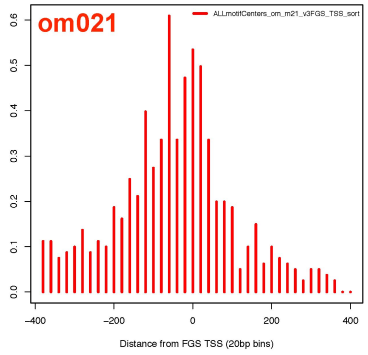	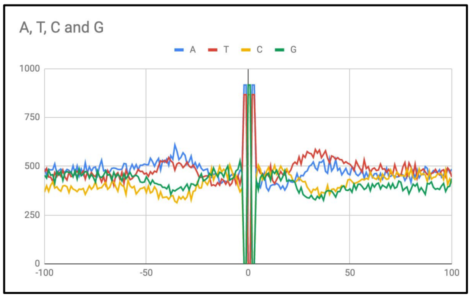

Average MOA Coverage Around Motif (RPM)

All	Not in Repeats (NR)	In Repeats (IR)
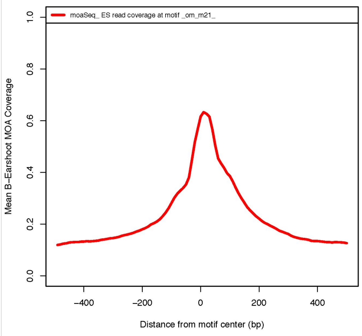	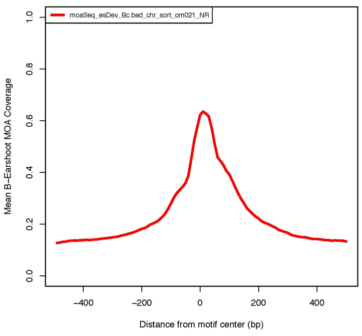	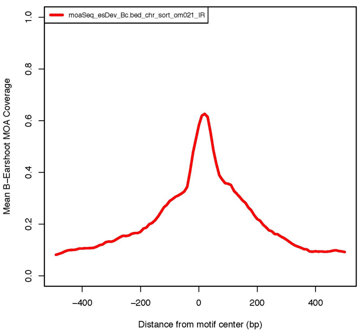

Motif Name	Total Number	In Repeats
om022	1,022	24%
Consensus	syCCAGTCCAssc	
LOGO	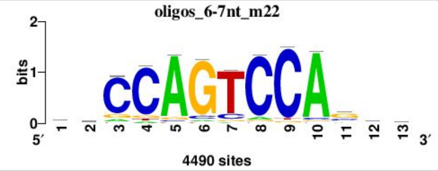	
LOGO RC	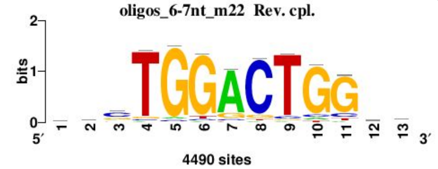	

	Frequency Distr. at TSS (FGS, B73v3)	Average Local Base Frequency (FGS, B73v3)
Median Position Relative to TSS =	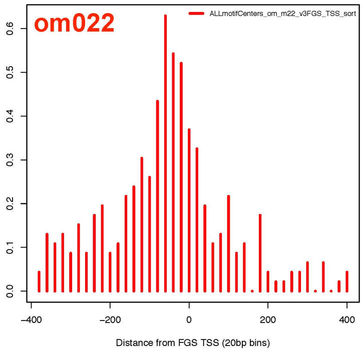	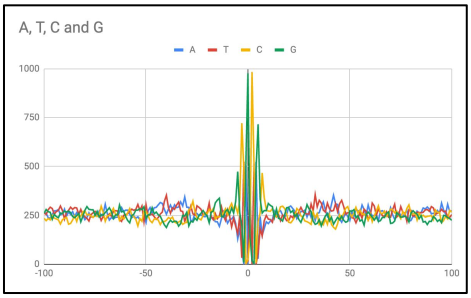
-74		

Average MOA Coverage Around Motif (RPM)

All	Not in Repeats (NR)	In Repeats (IR)
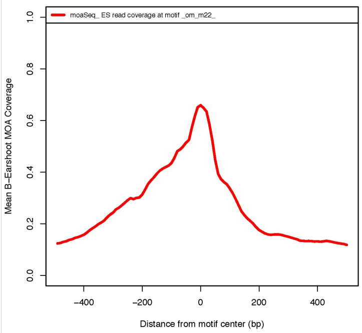	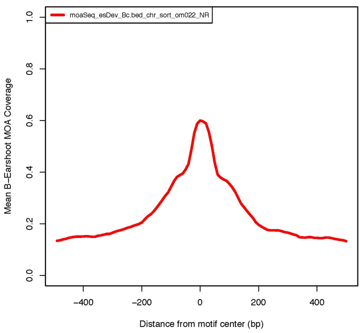	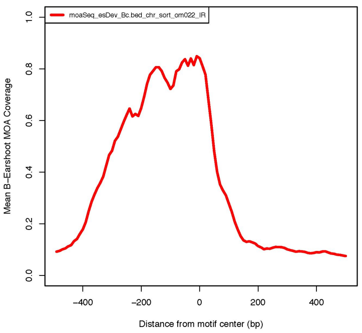

Motif Name	Total Number	In Repeats
**om023**	**2,797**	**7%**
**Consensus**	**ssCGCCGCss**	
**LOGO**	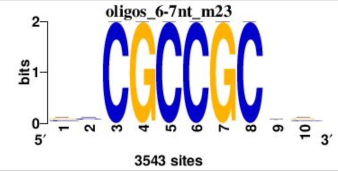	
**LOGO RC**	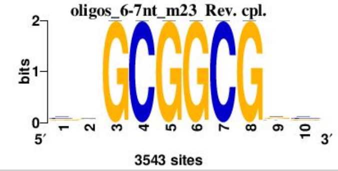	

	Frequency Distr. at TSS (FGS, B73v3)	Average Local Base Frequency (FGS, B73v3)
**Median Position Relative to TSS =**	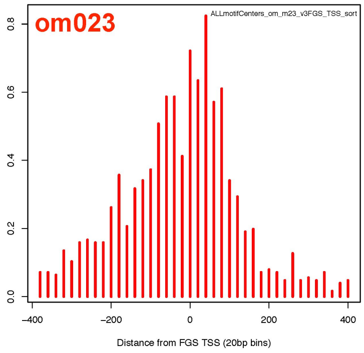	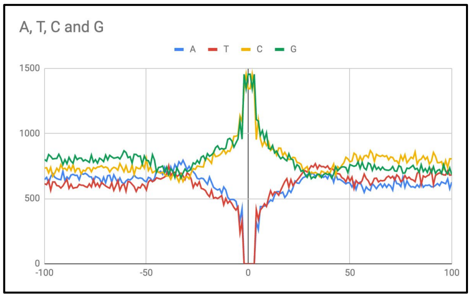
**-40**		

Average MOA Coverage Around Motif (RPM)

All	Not in Repeats (NR)	In Repeats (IR)
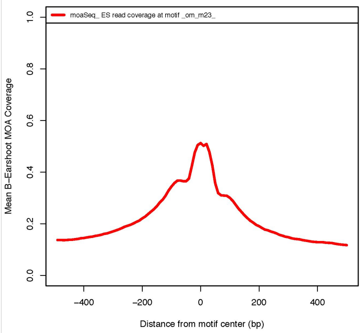	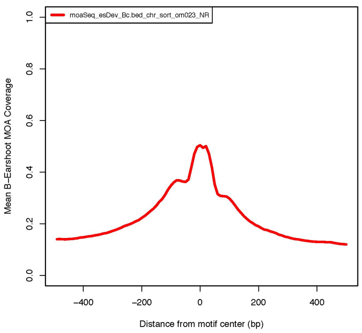	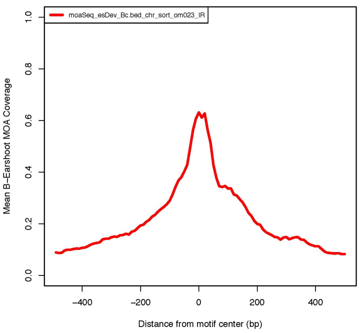

Motif Name	Total Number	In Repeats
om024	657	5%
Consensus	sbGCTCCGCTss	
LOGO	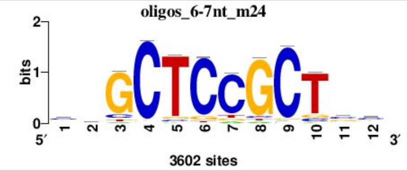	
LOGO RC	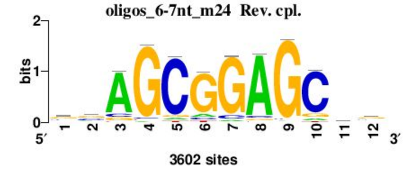	

	Frequency Distr. at TSS (FGS, B73v3)	Average Local Base Frequency (FGS, B73v3)
Median Position Relative to TSS =	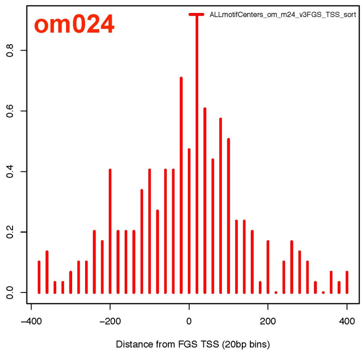	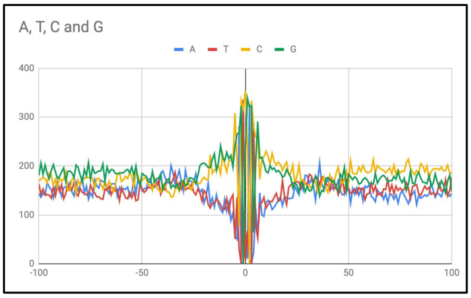
-30		

Average MOA Coverage Around Motif (RPM)

All	Not in Repeats (NR)	In Repeats (IR)
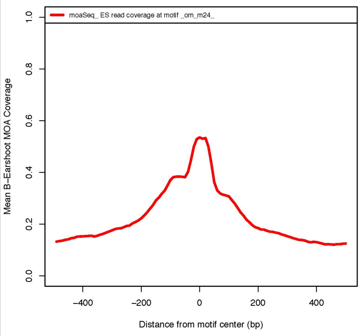	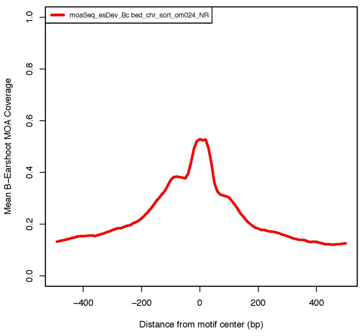	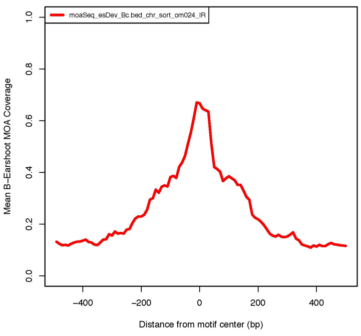

Motif Name	Total Number	In Repeats
om025	530	5%
Consensus	vvCAGGACAss	
LOGO	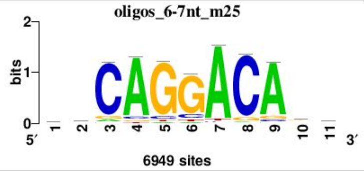	
LOGO RC	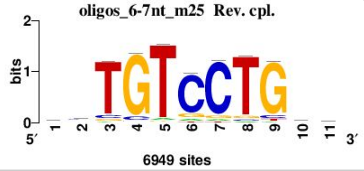	

	Frequency Distr. at TSS (FGS, B73v3)	Average Local Base Frequency (FGS, B73v3)
Median Position Relative to TSS =	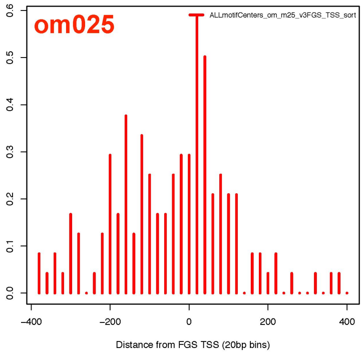	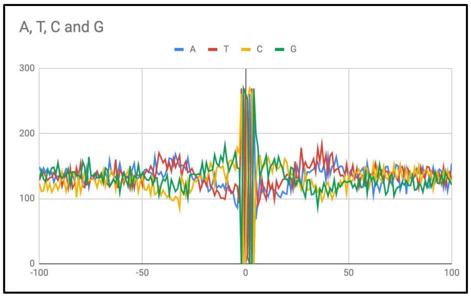
-129		

Average MOA Coverage Around Motif (RPM)

All	Not in Repeats (NR)	In Repeats (IR)

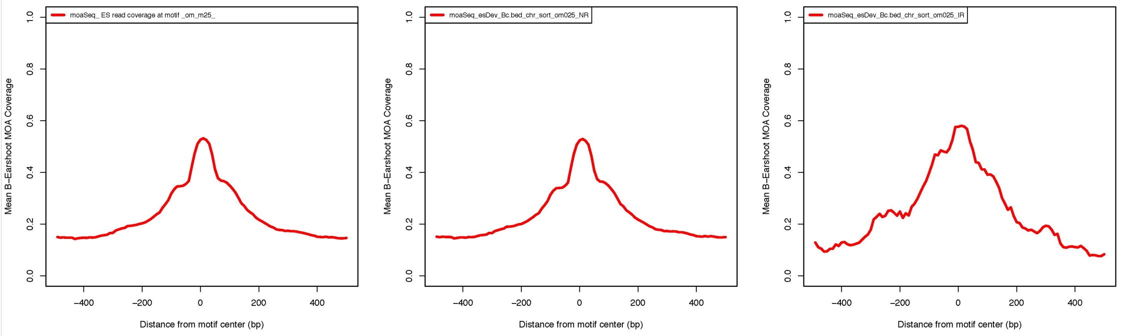

Motif Name	Total Number	In Repeats
om026	1,739	10%
Consensus	yycTCTTCTCyy	

LOGO

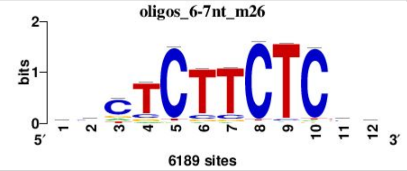

LOGO RC

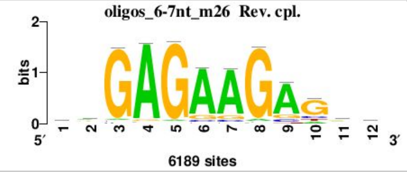

	Frequency Distr. at TSS (FGS, B73v3)	Average Local Base Frequency (FGS, B73v3)
Median Position Relative to TSS = +1	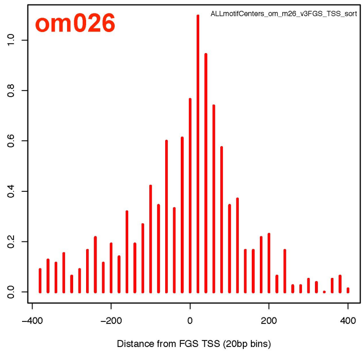	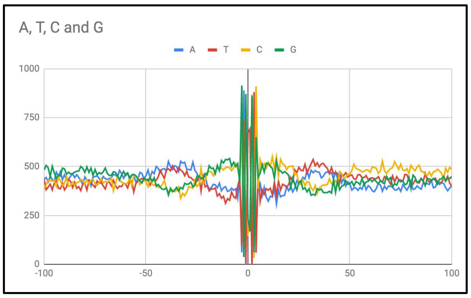

Average MOA Coverage Around Motif (RPM)

All	Not in Repeats (NR)	In Repeats (IR)
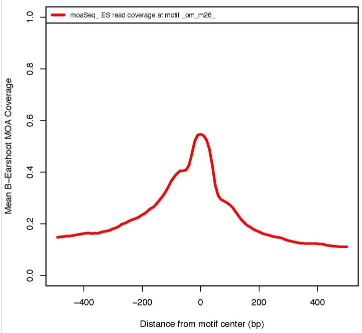	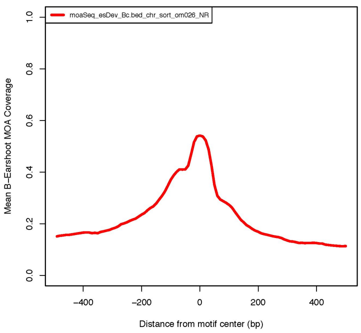	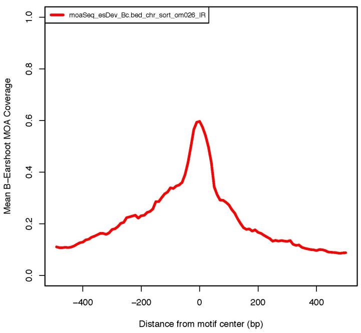

Motif Name	Total Number	In Repeats
om027	2,770	9%
Consensus	gcATGCATgc	
LOGO	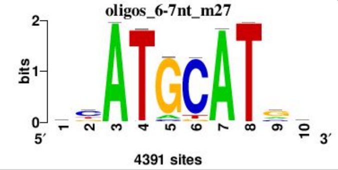	
LOGO RC	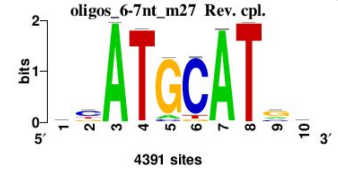	

	Frequency Distr. at TSS (FGS, B73v3)	Average Local Base Frequency (FGS, B73v3)
Median Position Relative to TSS = -154	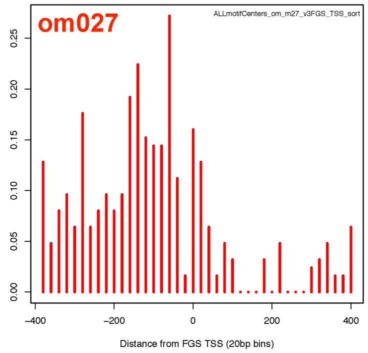	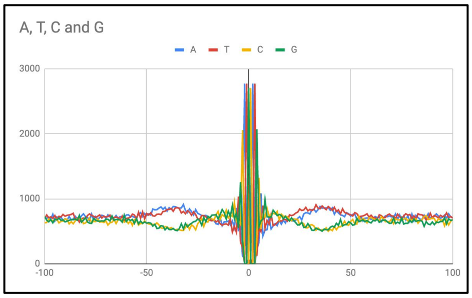

Average MOA Coverage Around Motif (RPM)

All	Not in Repeats (NR)	In Repeats (IR)
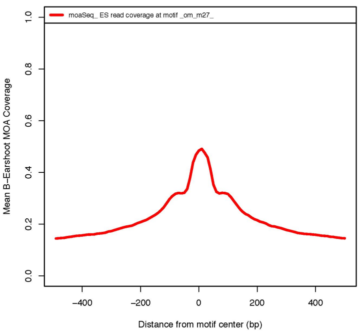	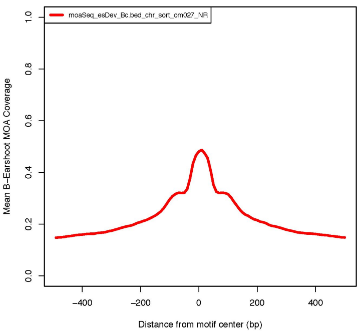	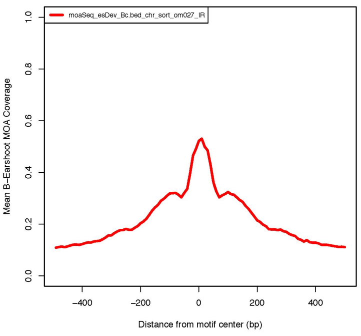

Motif Name	Total Number	In Repeats
om028	1,413	12%
Consensus	scAGCGCAss	
LOGO	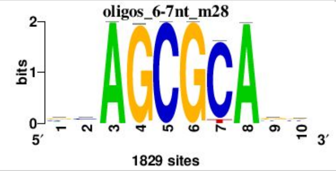	
LOGO RC	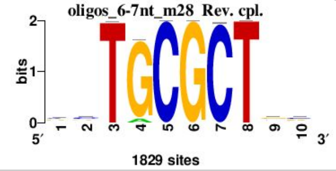	

	Frequency Distr. at TSS (FGS, B73v3)	Average Local Base Frequency (FGS, B73v3)
Median Position Relative to TSS =	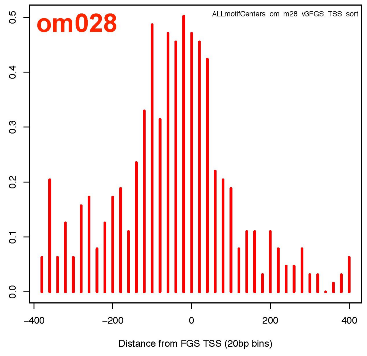	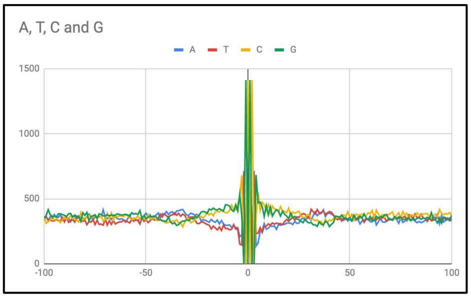
-83		

Average MOA Coverage Around Motif (RPM)

All	Not in Repeats (NR)	In Repeats (IR)
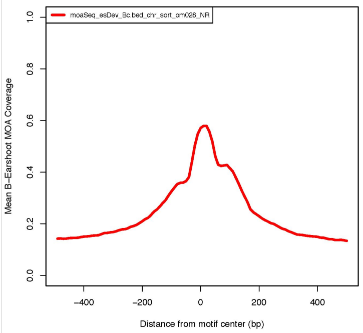	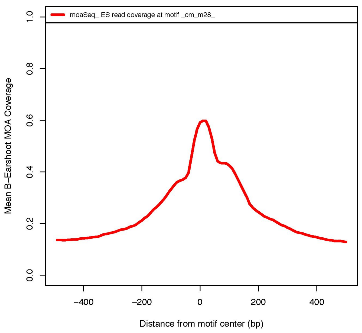	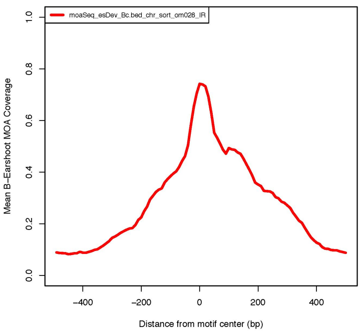

Motif Name	Total Number	In Repeats
om029	546	16%
Consensus	cctCACCTCAcsy	
LOGO	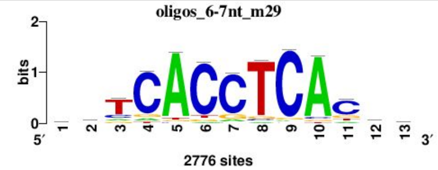	
LOGO RC	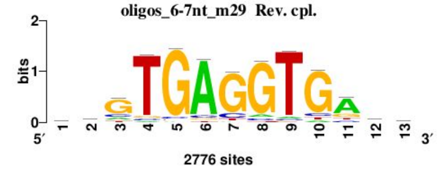	

	Frequency Distr. at TSS (FGS, B73v3)	Average Local Base Frequency (FGS, B73v3)
Median Position Relative to TSS =	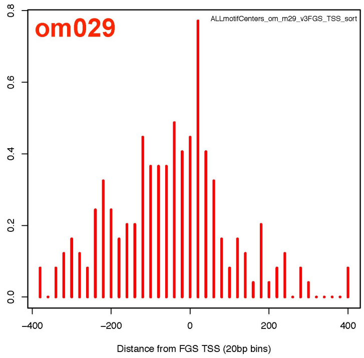	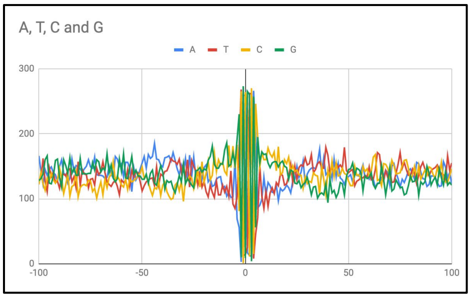
-50		

Average MOA Coverage Around Motif (RPM)

All	Not in Repeats (NR)	In Repeats (IR)
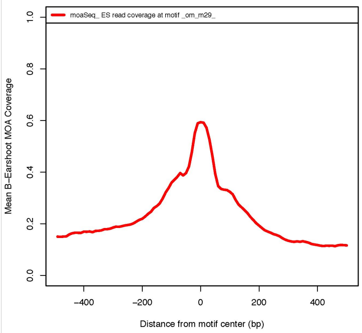	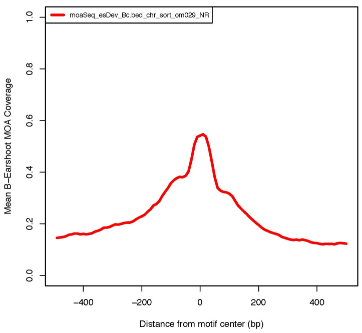	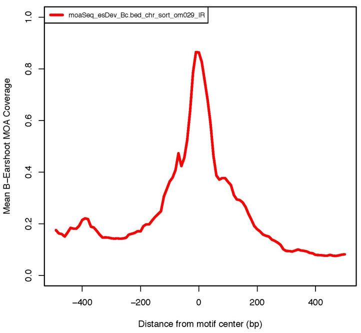

Motif Name	Total Number	In Repeats
om030	1,370	47%
Consensus	ykTTGCAAmr	
LOGO	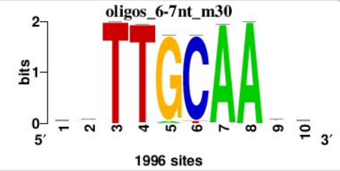	
LOGO RC	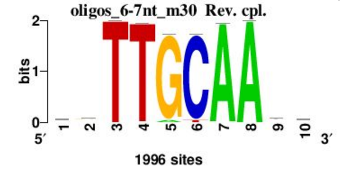	

	Frequency Distr. at TSS (FGS, B73v3)	Average Local Base Frequency (FGS, B73v3)
Median Position Relative to TSS =	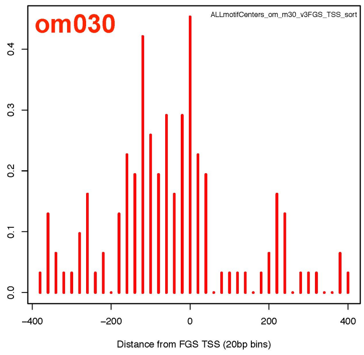	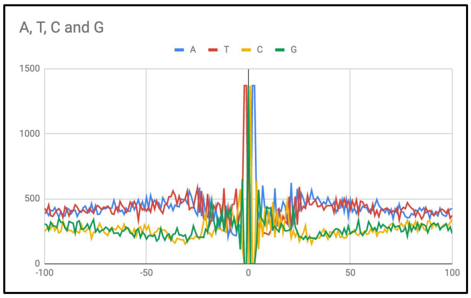
-10		

Average MOA Coverage Around Motif (RPM)

All	Not in Repeats (NR)	In Repeats (IR)
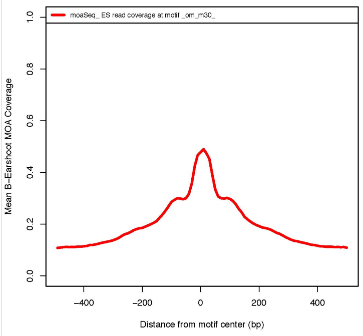	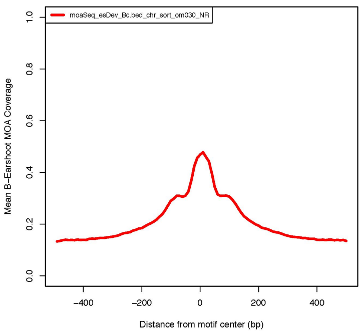	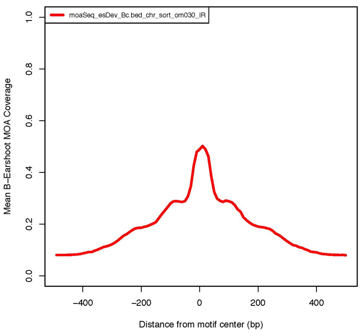

Motif Name	Total Number	In Repeats
om031	535	9%
Consensus	scACTCCACss	

LOGO

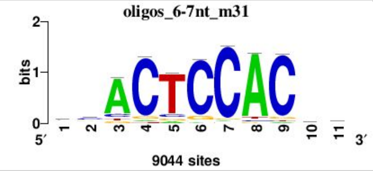

LOGO RC

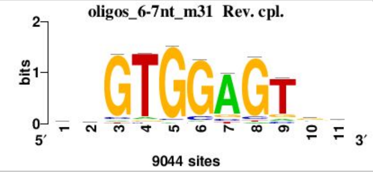

	Frequency Distr. at TSS (FGS, B73v3)	Average Local Base Frequency (FGS, B73v3)

Median Position Relative to TSS = -22

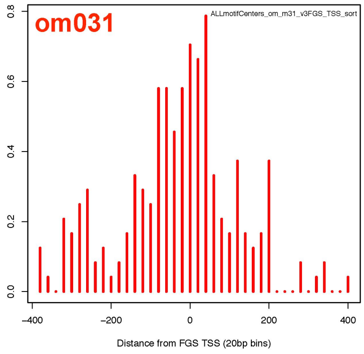

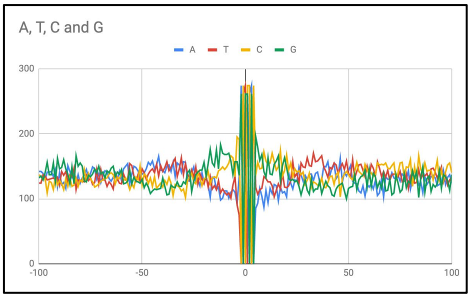

Average MOA Coverage Around Motif (RPM)

All	Not in Repeats (NR)	In Repeats (IR)

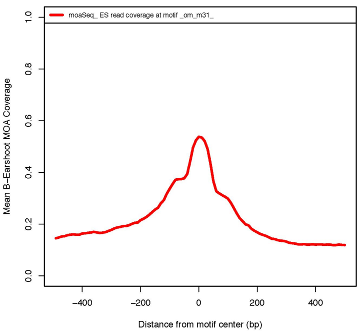

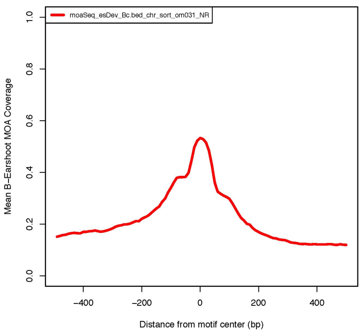

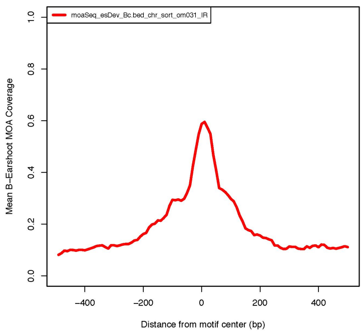

Motif Name	Total Number	In Repeats
om032	823	7%
Consensus	cyGCACCGCAss	
LOGO	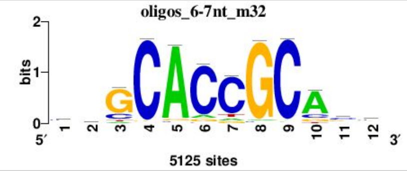	
LOGO RC	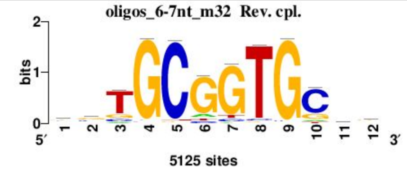	

	Frequency Distr. at TSS (FGS, B73v3)	Average Local Base Frequency (FGS, B73v3)
Median Position Relative to TSS =	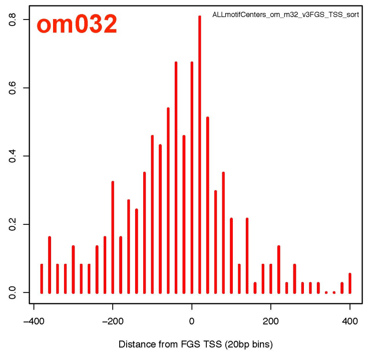	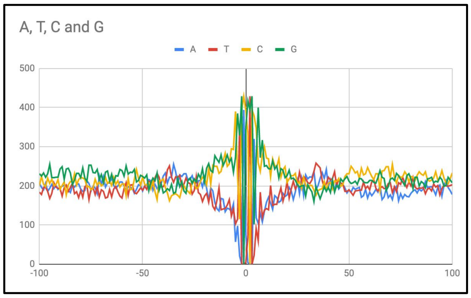
-98		

Average MOA Coverage Around Motif (RPM)

All	Not in Repeats (NR)	In Repeats (IR)
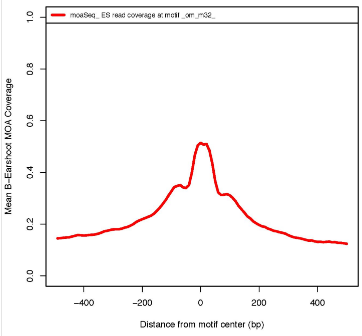	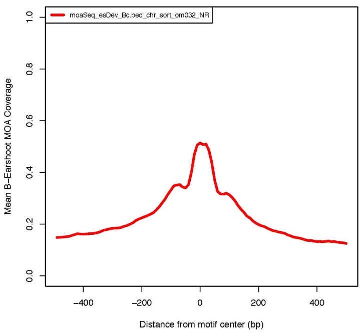	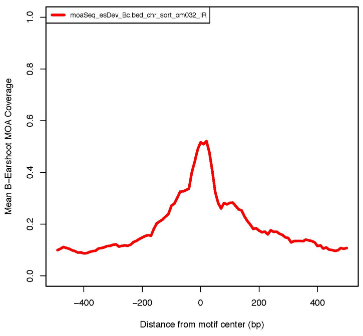

Motif Name	Total Number	In Repeats
om033	822	21%
Consensus	ssCTCGGCTCagCccs	
LOGO	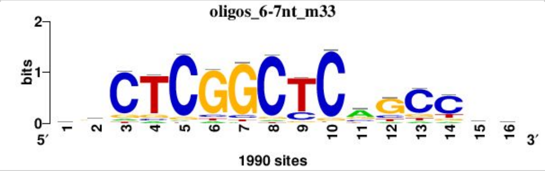	
LOGO RC	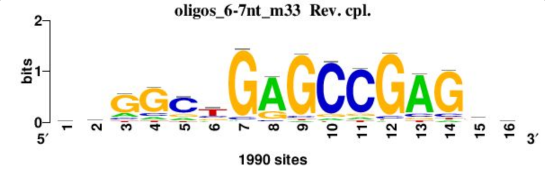	

	Frequency Distr. at TSS (FGS, B73v3)	Average Local Base Frequency (FGS, B73v3)
Median Position Relative to TSS =	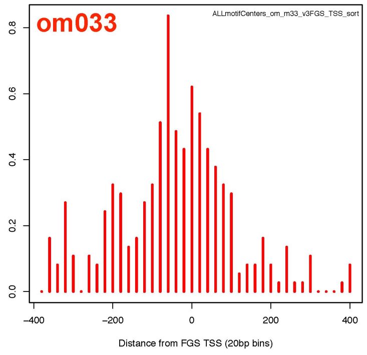	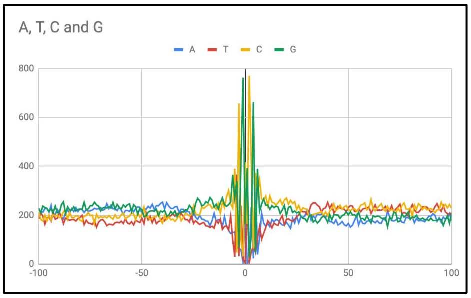
-68		

Average MOA Coverage Around Motif (RPM)

All	Not in Repeats (NR)	In Repeats (IR)
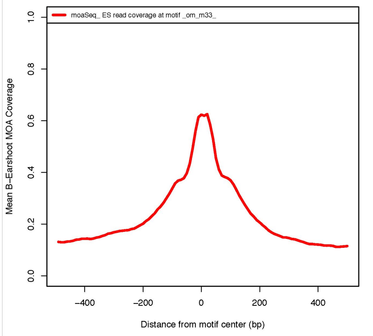	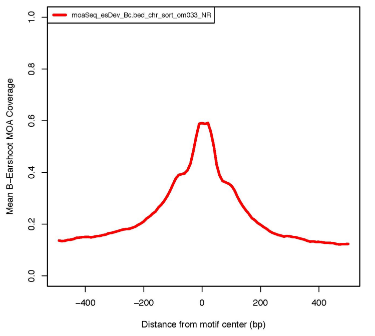	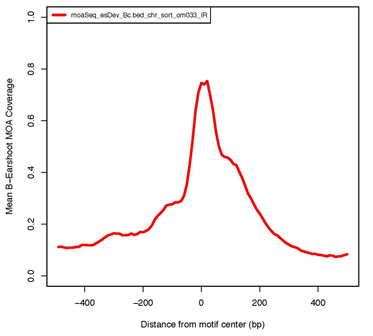

Motif Name	Total Number	In Repeats
om034	1,231	19%
Consensus	vrAACGAAmv	
LOGO	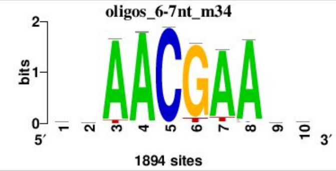	
LOGO RC	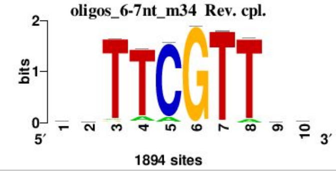	

	Frequency Distr. at TSS (FGS, B73v3)	Average Local Base Frequency (FGS, B73v3)
Median Position Relative to TSS =	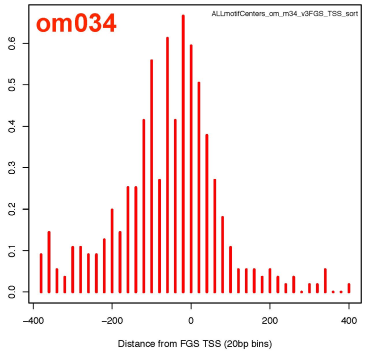	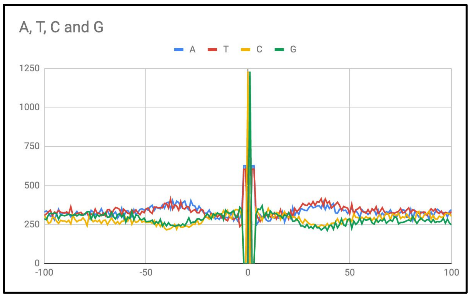
-71		

Average MOA Coverage Around Motif (RPM)

All	Not in Repeats (NR)	In Repeats (IR)
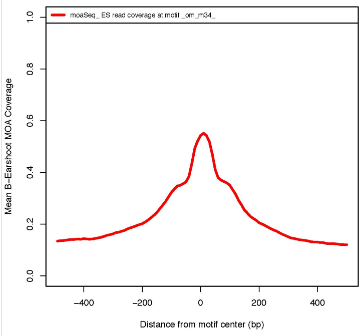	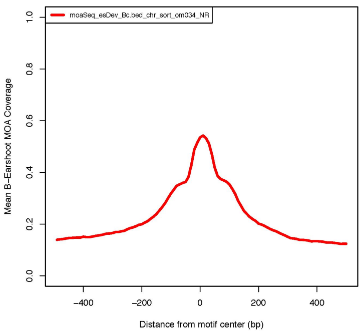	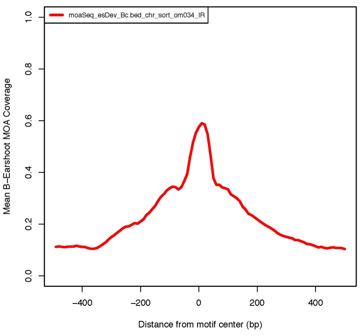

Motif Name	Total Number	In Repeats
om035	410	8%
Consensus	bcGCTTTGCbb	
LOGO	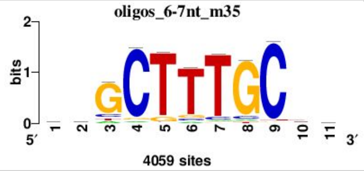	
LOGO RC	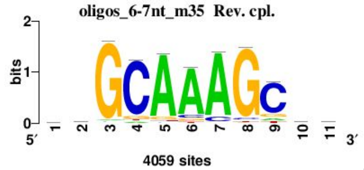	

	Frequency Distr. at TSS (FGS, B73v3)	Average Local Base Frequency (FGS, B73v3)
Median Position Relative to TSS = -94	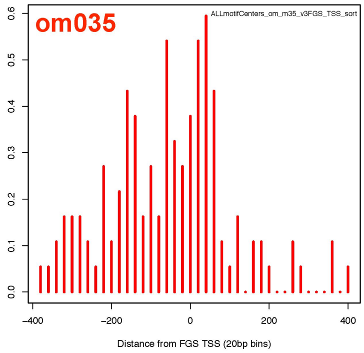	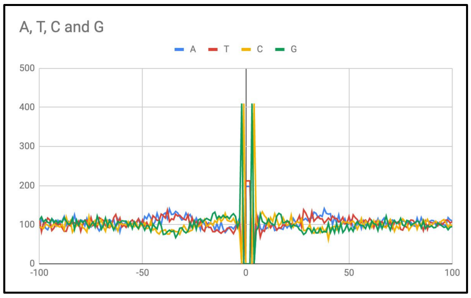

Average MOA Coverage Around Motif (RPM)

All	Not in Repeats (NR)	In Repeats (IR)
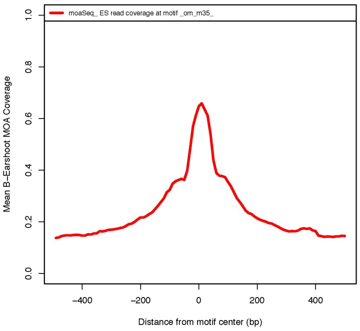	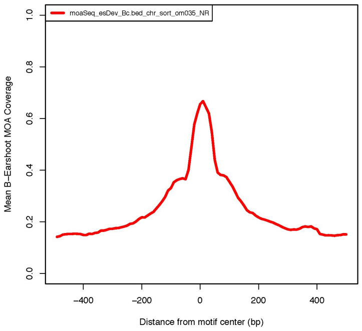	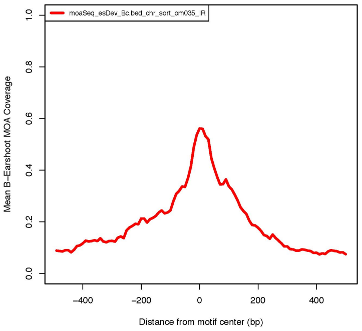

Motif Name	Total Number	In Repeats
om036	1,563	13%
Consensus	rvAAGCAArv	
LOGO	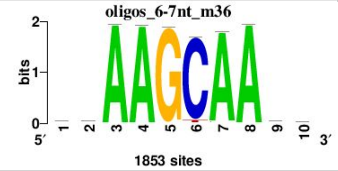	
LOGO RC	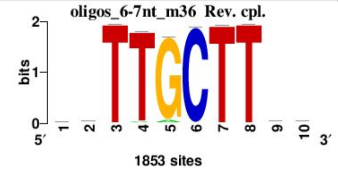	

	Frequency Distr. at TSS (FGS, B73v3)	Average Local Base Frequency (FGS, B73v3)
Median Position Relative to TSS = -38	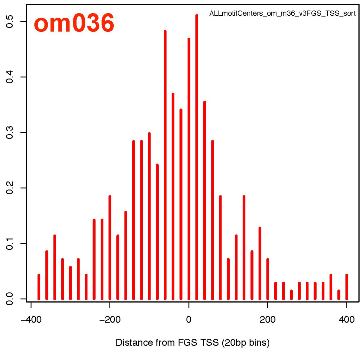	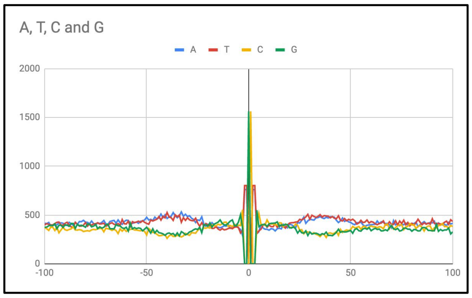

Average MOA Coverage Around Motif (RPM)

All	Not in Repeats (NR)	In Repeats (IR)
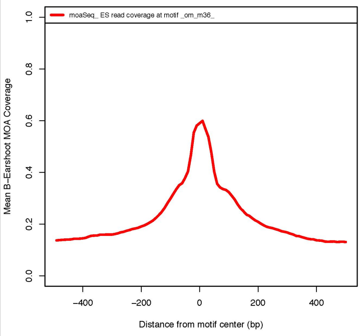	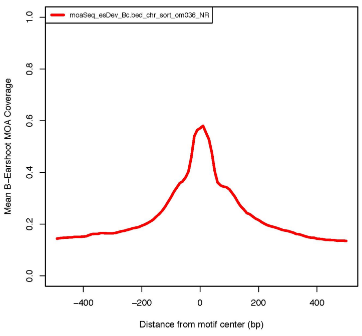	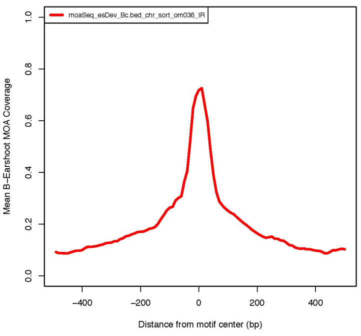

Motif Name	Total Number	In Repeats
om037	784	11%
Consensus	ssCGCACGGCaCss	
LOGO	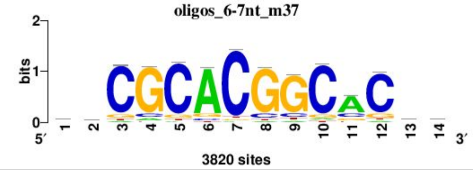	
LOGO RC	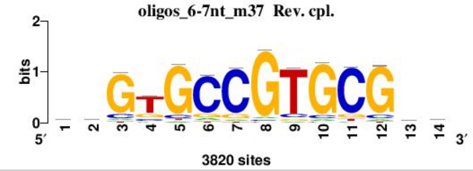	

	Frequency Distr. at TSS (FGS, B73v3)	Average Local Base Frequency (FGS, B73v3)
Median Position Relative to TSS =	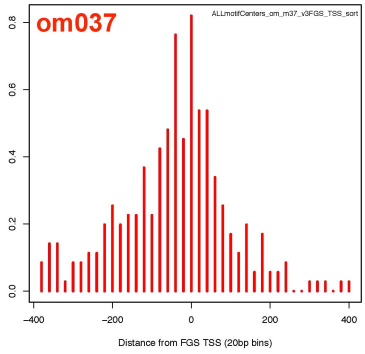	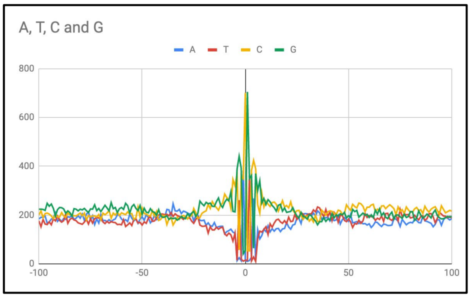
-68		

Average MOA Coverage Around Motif (RPM)

All	Not in Repeats (NR)	In Repeats (IR)
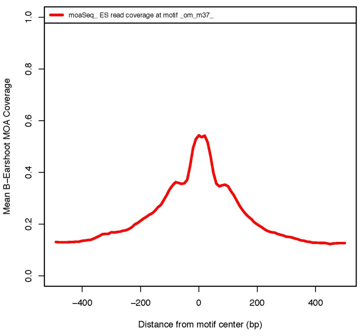	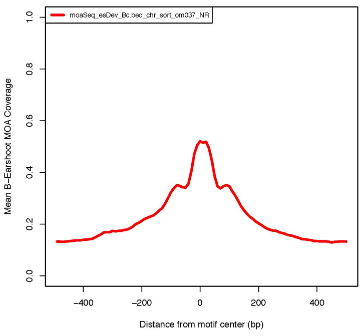	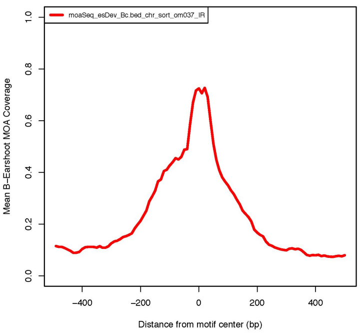

Motif Name	Total Number	In Repeats
om038	893	24%
Consensus	ctACTCTAsw	
LOGO	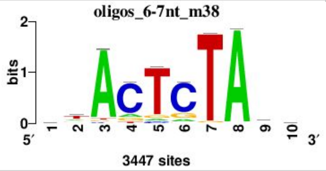	
LOGO RC	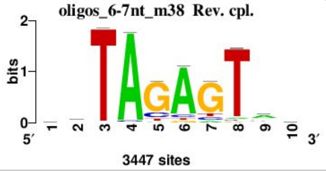	

	Frequency Distr. at TSS (FGS, B73v3)	Average Local Base Frequency (FGS, B73v3)
Median Position Relative to TSS =	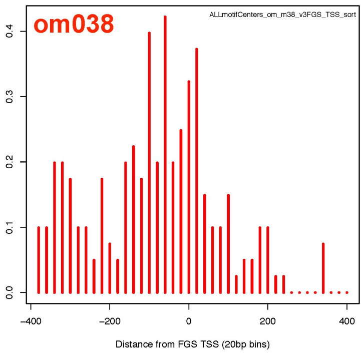	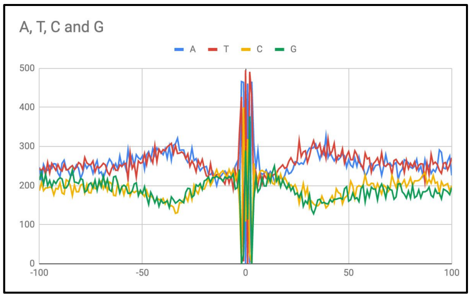
-102		

Average MOA Coverage Around Motif (RPM)

All	Not in Repeats (NR)	In Repeats (IR)
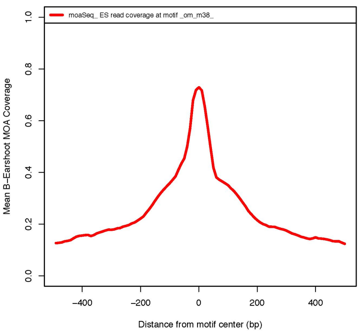	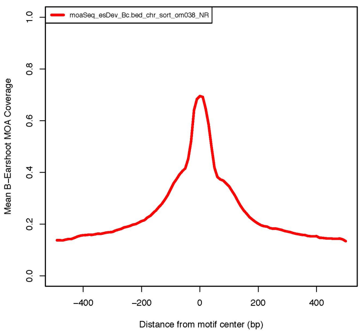	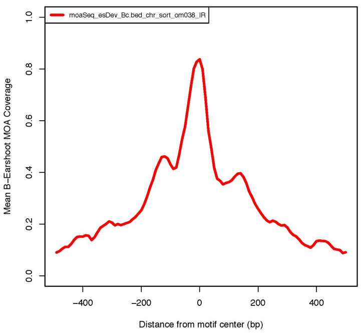

Motif Name	Total Number	In Repeats
om039	2,451	5%
Consensus	bsCCTGCCys	
LOGO	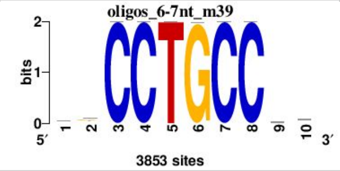	
LOGO RC	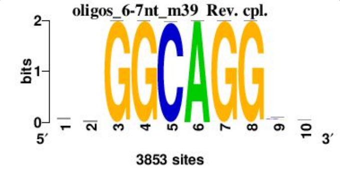	

	Frequency Distr. at TSS (FGS, B73v3)	Average Local Base Frequency (FGS, B73v3)
Median Position Relative to TSS =	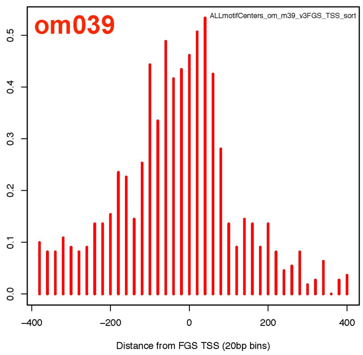	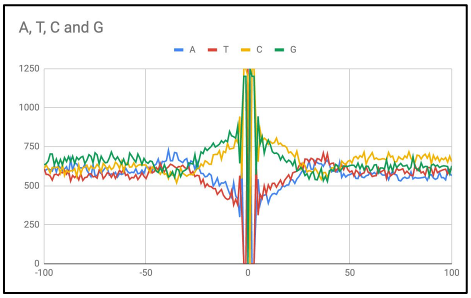
-65		

Average MOA Coverage Around Motif (RPM)

All	Not in Repeats (NR)	In Repeats (IR)
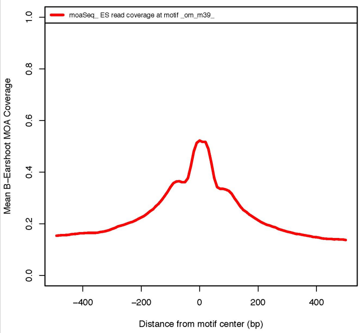	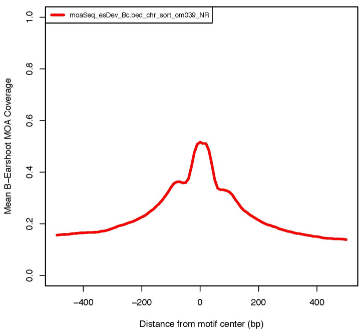	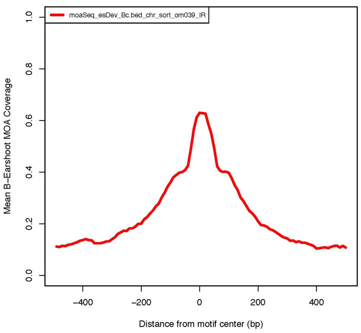

Motif Name	Total Number	In Repeats
om040	890	6%
Consensus	ssACGCCACGss	
LOGO	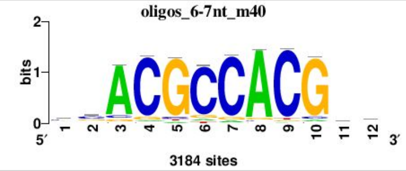	
LOGO RC	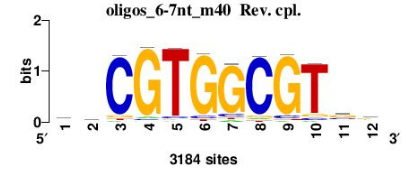	

	Frequency Distr. at TSS (FGS, B73v3)	Average Local Base Frequency (FGS, B73v3)
Median Position Relative to TSS =	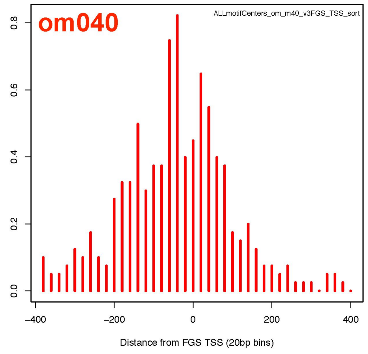	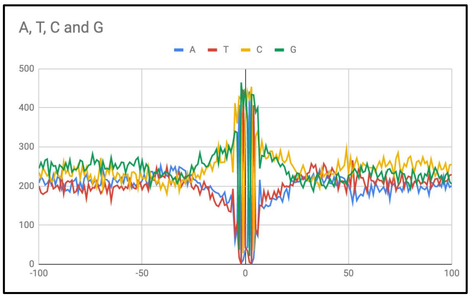
-76		

Average MOA Coverage Around Motif (RPM)

All	Not in Repeats (NR)	In Repeats (IR)
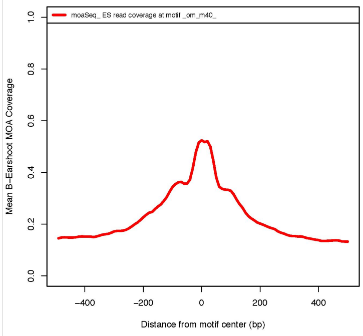	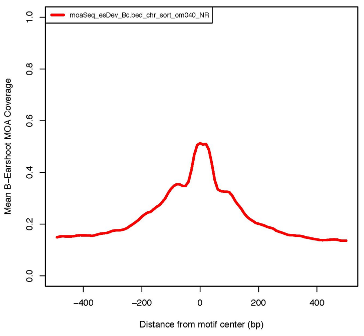	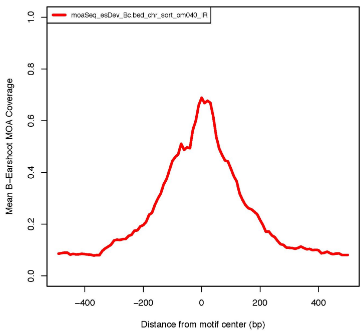

Motif Name	Total Number	In Repeats
om041	3,243	12%
Consensus	ssCCAGCCmv	
LOGO	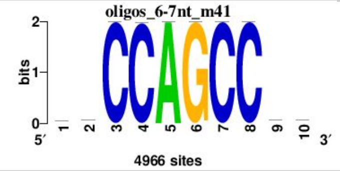	
LOGO RC	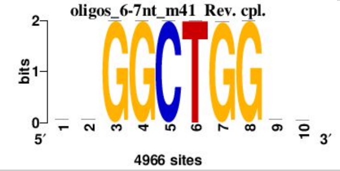	

	Frequency Distr. at TSS (FGS, B73v3)	Average Local Base Frequency (FGS, B73v3)
Median Position Relative to TSS = -80	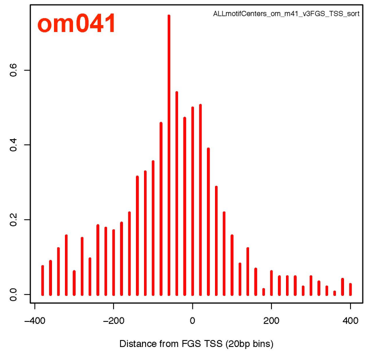	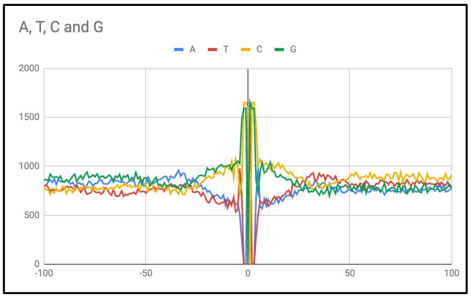

Average MOA Coverage Around Motif (RPM)

All	Not in Repeats (NR)	In Repeats (IR)
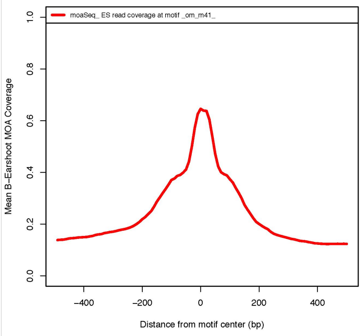	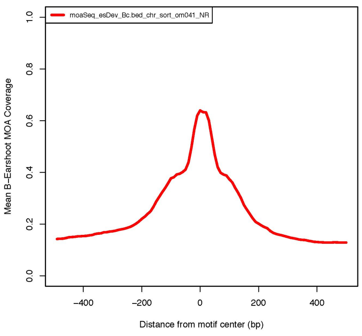	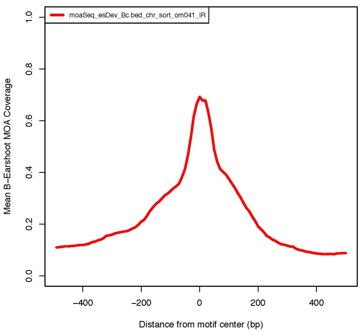

Motif Name	Total Number	In Repeats
om042	1,167	8%
Consensus	grCGAGACGAsr	
LOGO	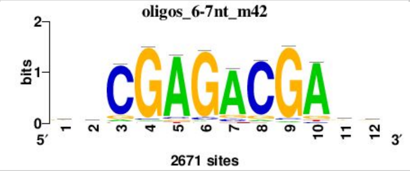	
LOGO RC	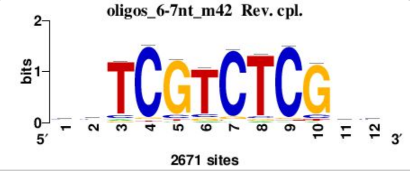	

	Frequency Distr. at TSS (FGS, B73v3)	Average Local Base Frequency (FGS, B73v3)
Median Position Relative to TSS =	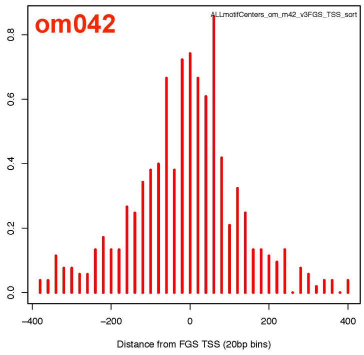	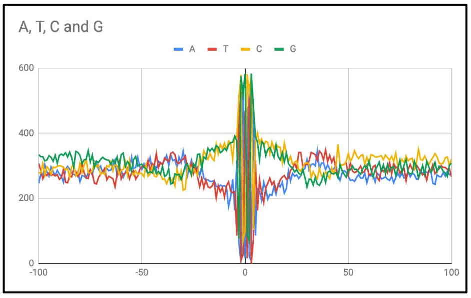
-20		

Average MOA Coverage Around Motif (RPM)

All	Not in Repeats (NR)	In Repeats (IR)
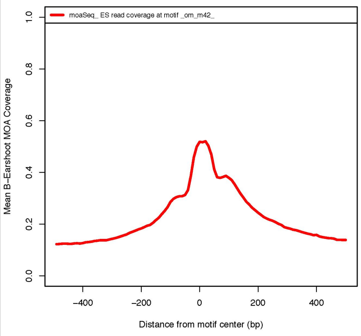	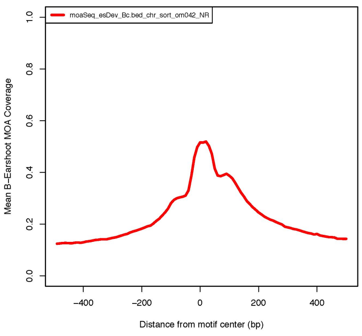	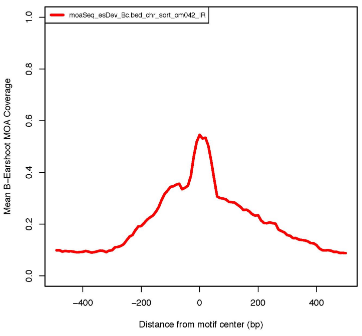

Motif Name	Total Number	In Repeats
om043	447	7%
Consensus	ssACGGGACss	
LOGO	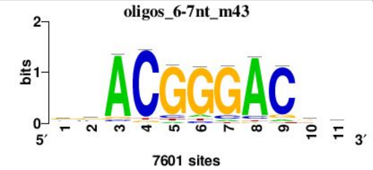	
LOGO RC	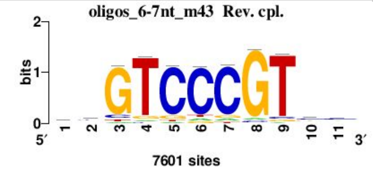	

	Frequency Distr. at TSS (FGS, B73v3)	Average Local Base Frequency (FGS, B73v3)
Median Position Relative to TSS =	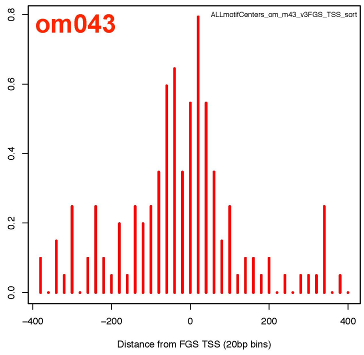	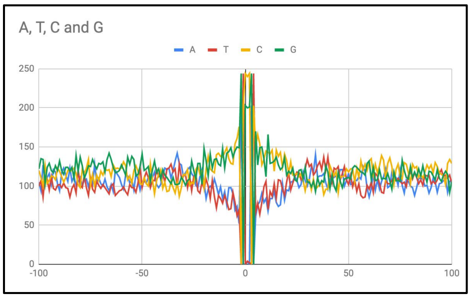
-60		

Average MOA Coverage Around Motif (RPM)

All	Not in Repeats (NR)	In Repeats (IR)
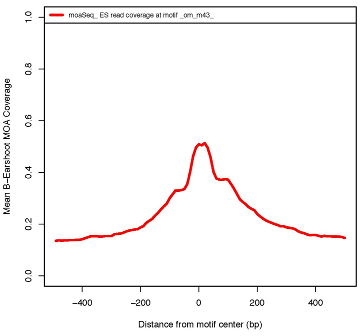	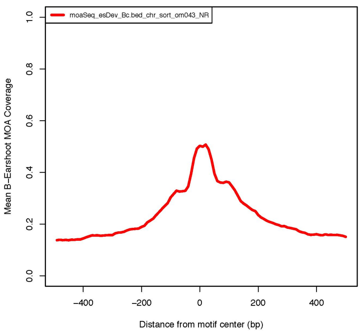	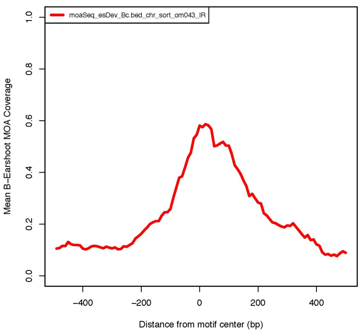

Motif Name	Total Number	In Repeats
om044	578	74%
Consensus	raGGGGTATsg	
LOGO	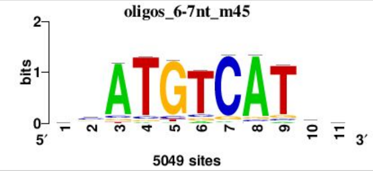	
LOGO RC	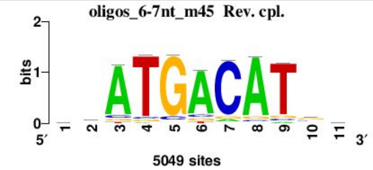	

	Frequency Distr. at TSS (FGS, B73v3)	Average Local Base Frequency (FGS, B73v3)
Median Position Relative to TSS =	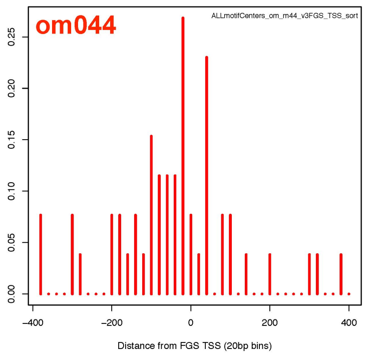	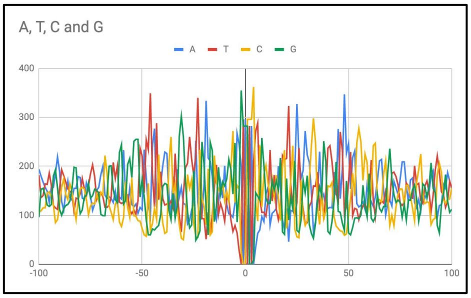
-26		

Average MOA Coverage Around Motif (RPM)

All	Not in Repeats (NR)	In Repeats (IR)
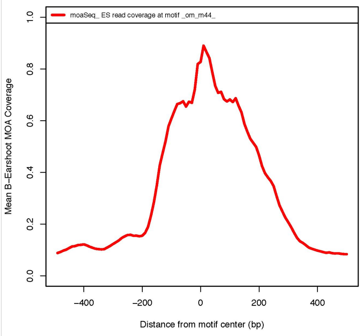	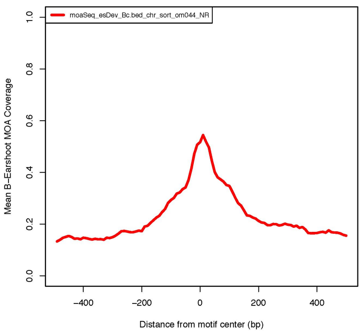	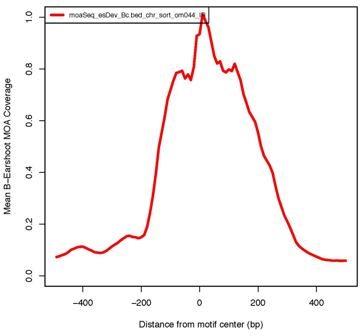

Motif Name	Total Number	In Repeats
om045	281	19%
Consensus	rsATGTCATsb	
LOGO	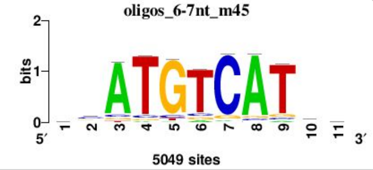	
LOGO RC	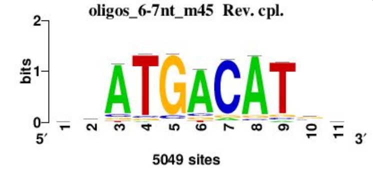	

	Frequency Distr. at TSS (FGS, B73v3)	Average Local Base Frequency (FGS, B73v3)
Median Position Relative to TSS = -173	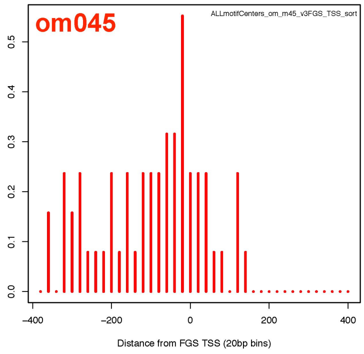	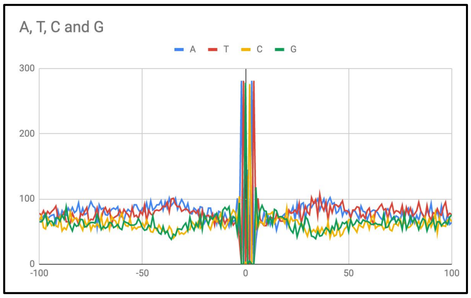

Average MOA Coverage Around Motif (RPM)

All	Not in Repeats (NR)	In Repeats (IR)
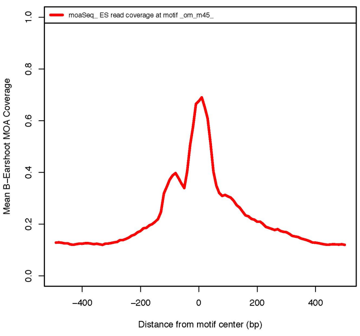	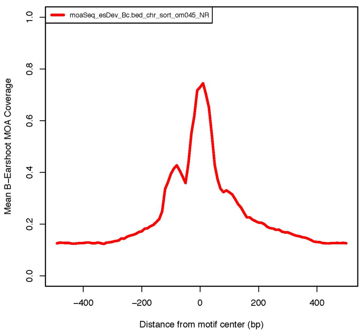	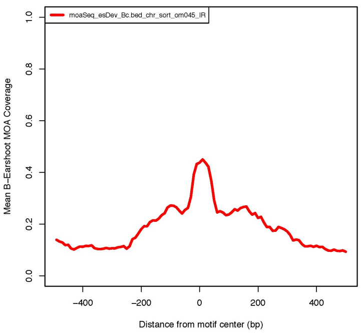

Motif Name	Total Number	In Repeats
om046	397	25%
Consensus	ycCATTTCAby	
LOGO	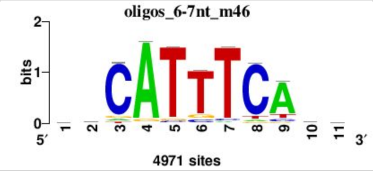	
LOGO RC	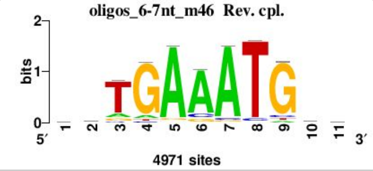	

	Frequency Distr. at TSS (FGS, B73v3)	Average Local Base Frequency (FGS, B73v3)
Median Position Relative to TSS =	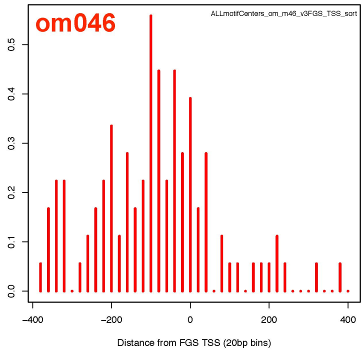	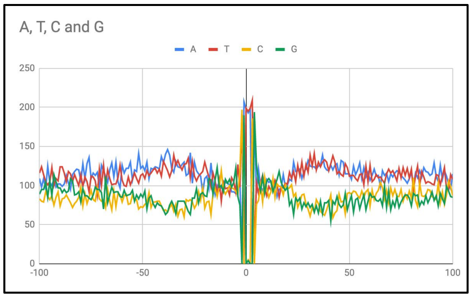
-49		

Average MOA Coverage Around Motif (RPM)

All	Not in Repeats (NR)	In Repeats (IR)
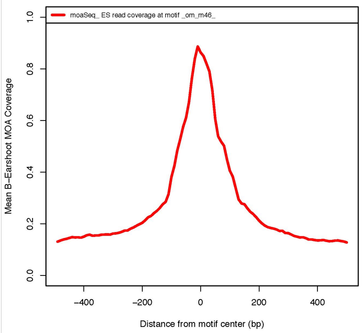	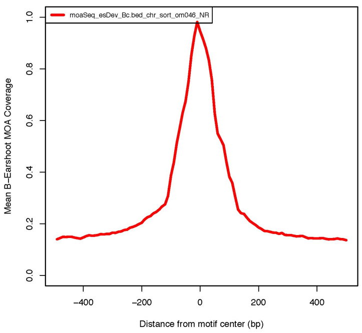	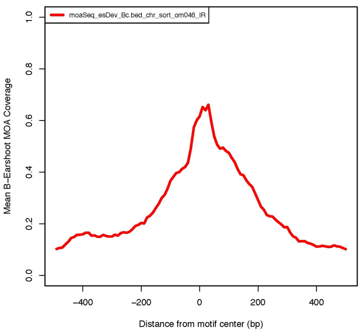

Motif Name	Total Number	In Repeats
om047	671	25%
Consensus	ssTCGCATCGCAys	
LOGO	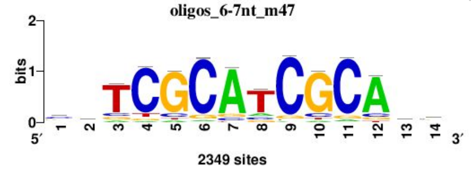	
LOGO RC	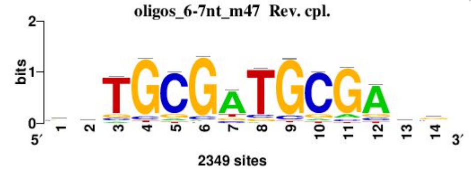	

	Frequency Distr. at TSS (FGS, B73v3)	Average Local Base Frequency (FGS, B73v3)
Median Position Relative to TSS =	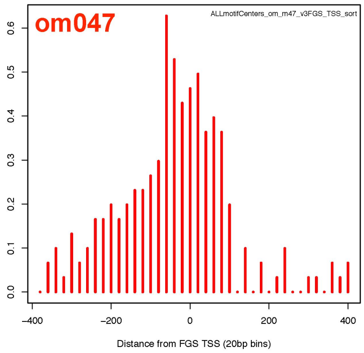	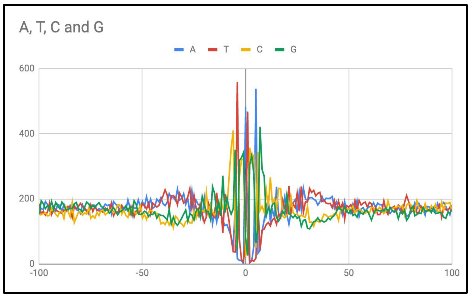
-101		

Average MOA Coverage Around Motif (RPM)

All	Not in Repeats (NR)	In Repeats (IR)
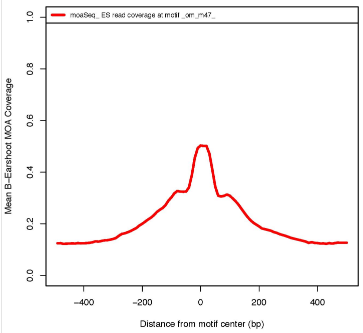	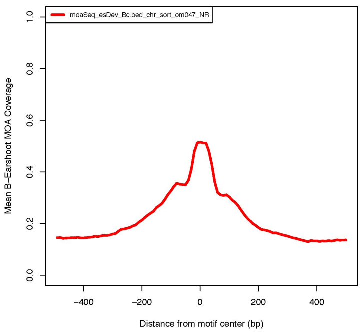	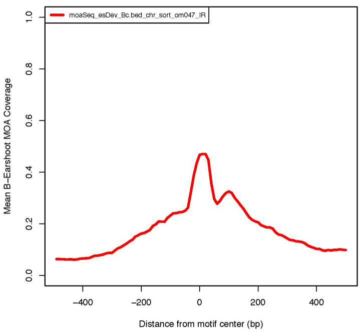

Motif Name	Total Number	In Repeats
om048	2,022	19%
Consensus	ssGACCCAss	
LOGO	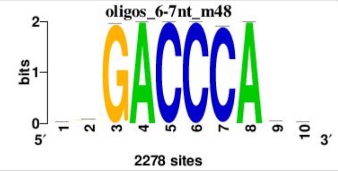	
LOGO RC	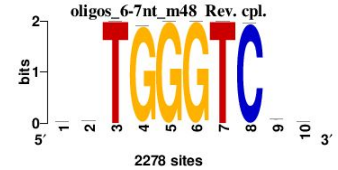	

	Frequency Distr. at TSS (FGS, B73v3)	Average Local Base Frequency (FGS, B73v3)
Median Position Relative to TSS =	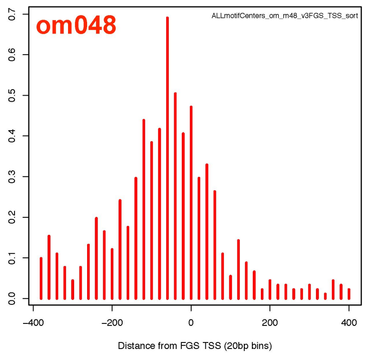	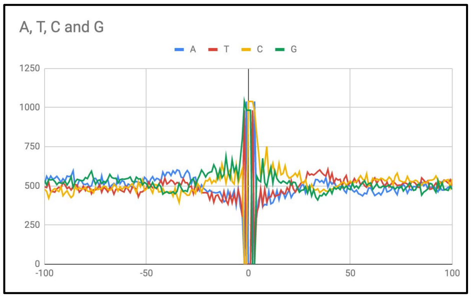
-82		

Average MOA Coverage Around Motif (RPM)

All	Not in Repeats (NR)	In Repeats (IR)
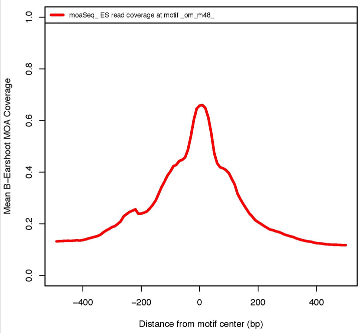	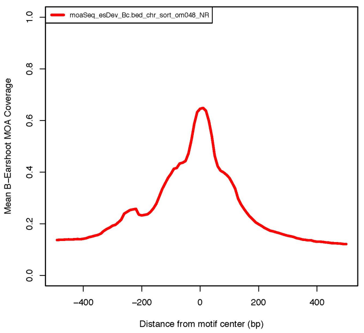	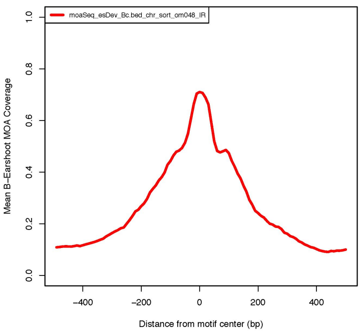

Motif Name	Total Number	In Repeats
om049	485	10%
Consensus	rvCAGAACAGvv	
LOGO	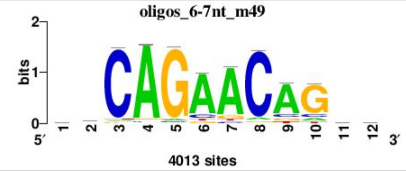	
LOGO RC	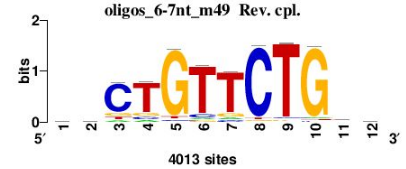	

	Frequency Distr. at TSS (FGS, B73v3)	Average Local Base Frequency (FGS, B73v3)
Median Position Relative to TSS =	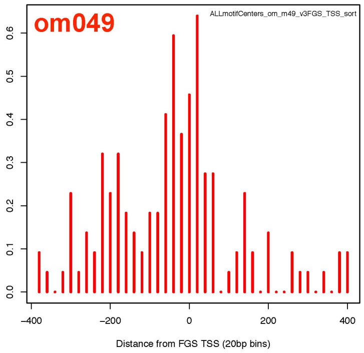	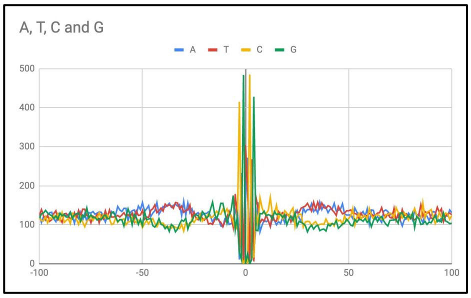
-13		

Average MOA Coverage Around Motif (RPM)

All	Not in Repeats (NR)	In Repeats (IR)
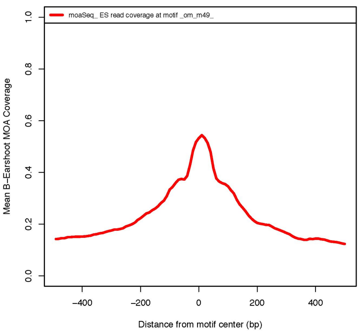	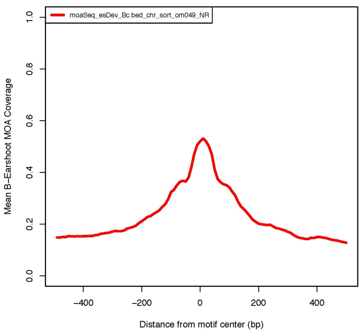	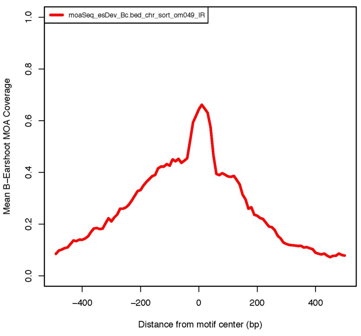

Motif Name	Total Number	In Repeats
om050	633	5%
Consensus	sgCTGCACTGCAGTGCAGcs	
LOGO	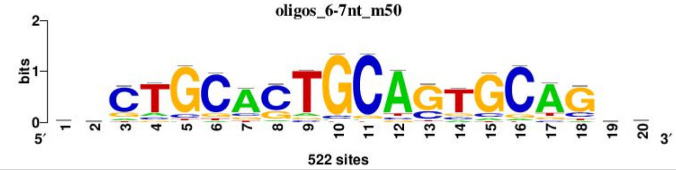	
LOGO RC	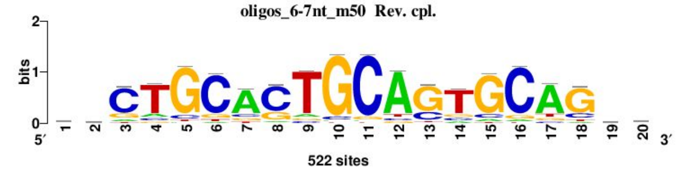	

	Frequency Distr. at TSS (FGS, B73v3)	Average Local Base Frequency (FGS, B73v3)
Median Position Relative to TSS =	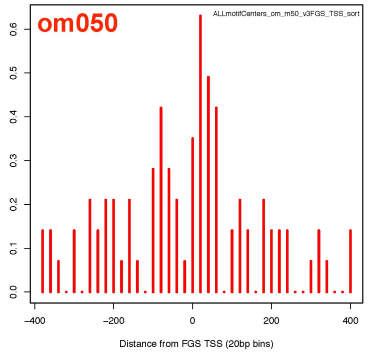	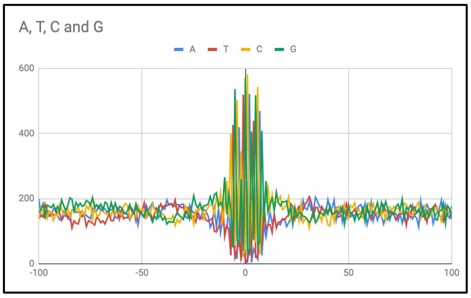
-4		

Average MOA Coverage Around Motif (RPM)

All	Not in Repeats (NR)	In Repeats (IR)
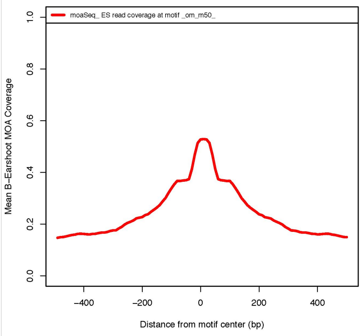	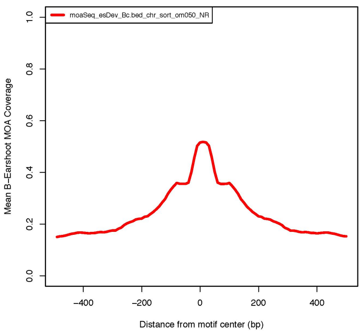	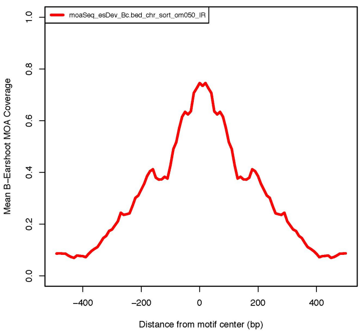

Motif Name	Total Number	In Repeats
om051	848	11%
Consensus	sgTACGTAcs	
LOGO	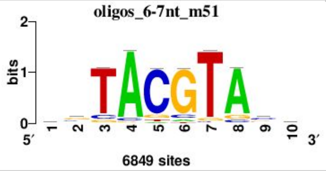	
LOGO RC	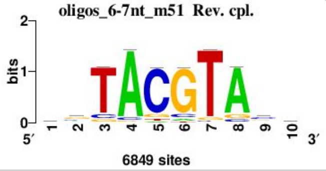	

	Frequency Distr. at TSS (FGS, B73v3)	Average Local Base Frequency (FGS, B73v3)
Median Position Relative to TSS =	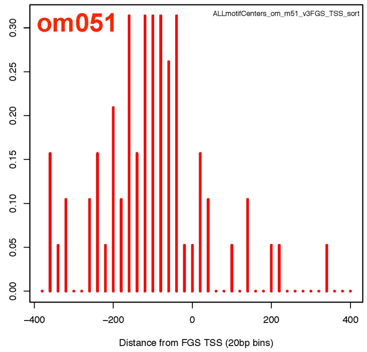	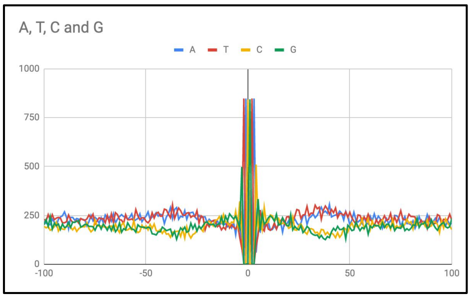
-108		

Average MOA Coverage Around Motif (RPM)

All	Not in Repeats (NR)	In Repeats (IR)
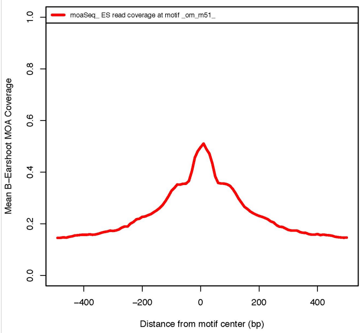	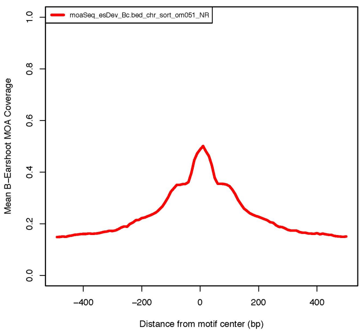	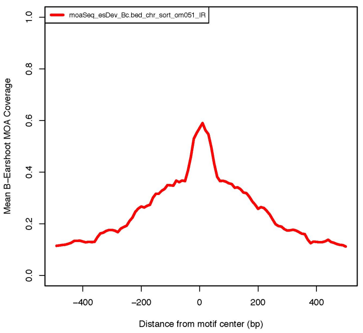

Motif Name	Total Number	In Repeats
om052	4,859	21%

Consensus yyTCTTTTCTTTyy

LOGO

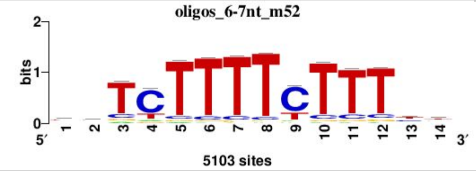

LOGO RC

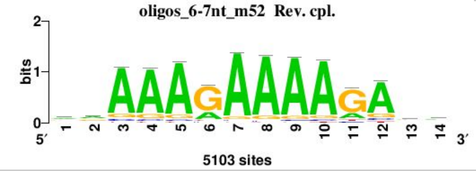

	Frequency Distr. at TSS (FGS, B73v3)	Average Local Base Frequency (FGS, B73v3)
Median Position Relative to TSS = -68	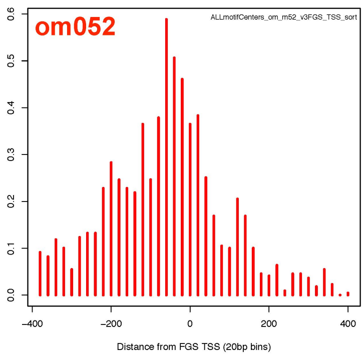	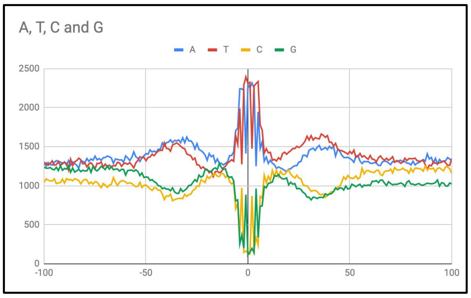

Average MOA Coverage Around Motif (RPM)

All	Not in Repeats (NR)	In Repeats (IR)
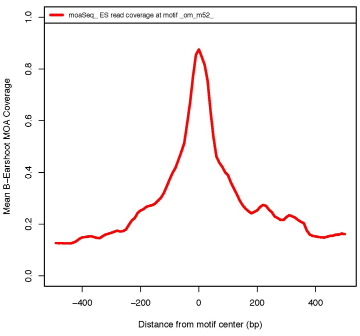	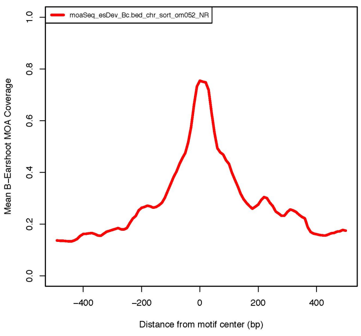	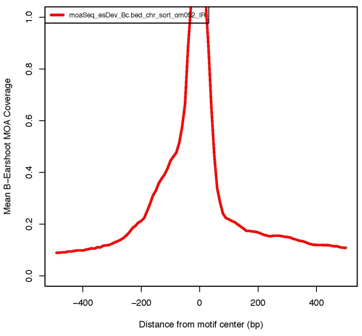

Motif Name	Total Number	In Repeats
om053	2,045	7%
Consensus	yytCTCCTCtcy	

LOGO

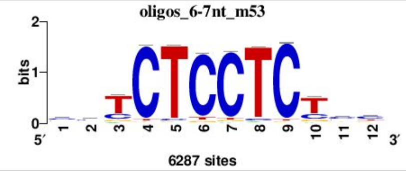

LOGO RC

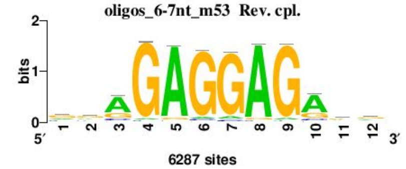

	Frequency Distr. at TSS (FGS, B73v3)	Average Local Base Frequency (FGS, B73v3)
Median Position Relative to TSS = -9	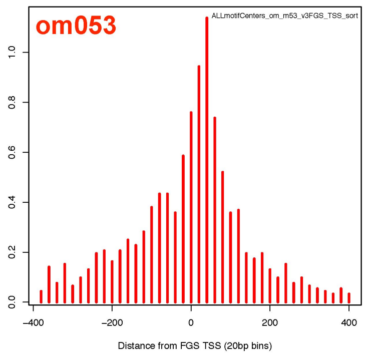	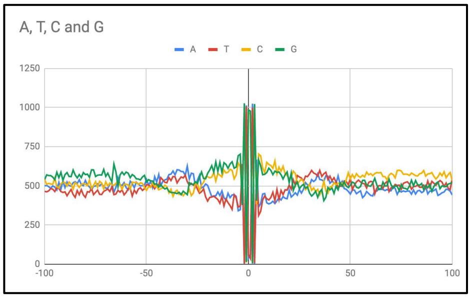

Average MOA Coverage Around Motif (RPM)

All	Not in Repeats (NR)	In Repeats (IR)
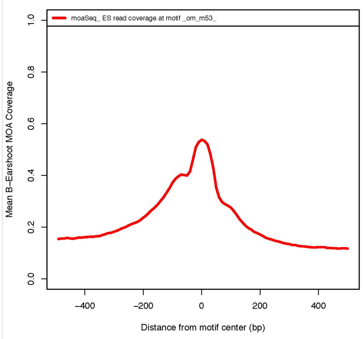	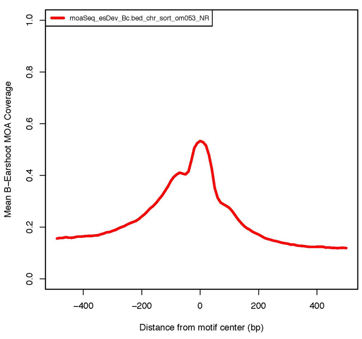	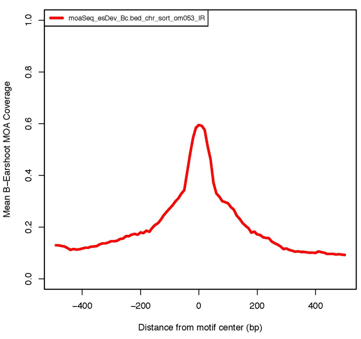

Motif Name	Total Number	In Repeats
om054	1,257	42%
Consensus	aaAAACTAAam	
LOGO	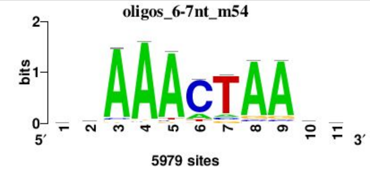	
LOGO RC	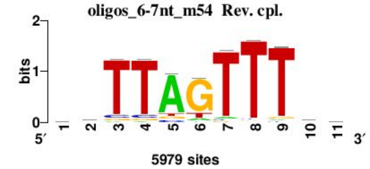	

	Frequency Distr. at TSS (FGS, B73v3)	Average Local Base Frequency (FGS, B73v3)
Median Position Relative to TSS =	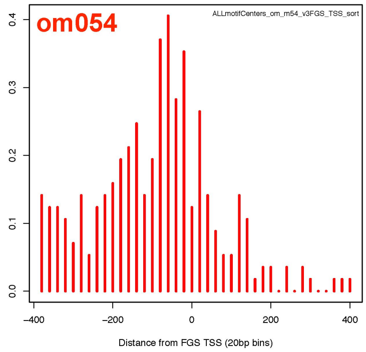	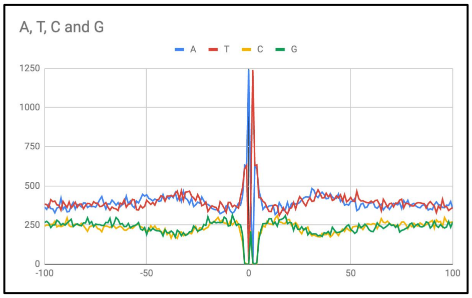
-69		

Average MOA Coverage Around Motif (RPM)		
All	Not in Repeats (NR)	In Repeats (IR)
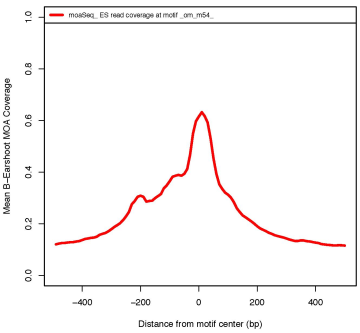	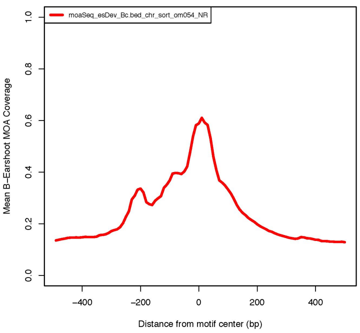	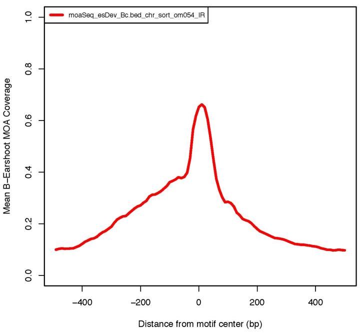

Motif Name	Total Number	In Repeats
om055	1,941	22%
Consensus	rmCCAAACmv	
LOGO	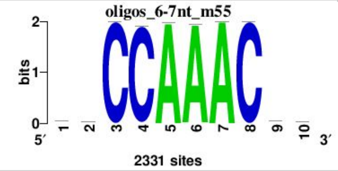	
LOGO RC	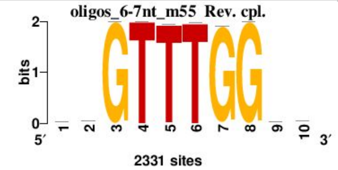	

	Frequency Distr. at TSS (FGS, B73v3)	Average Local Base Frequency (FGS, B73v3)
Median Position Relative to TSS = -79	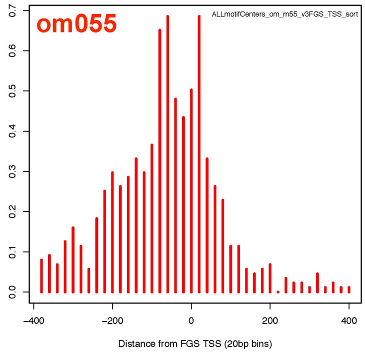	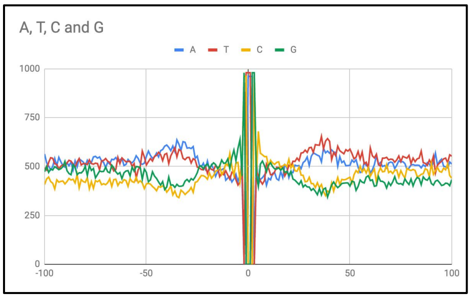

Average MOA Coverage Around Motif (RPM)		
All	Not in Repeats (NR)	In Repeats (IR)
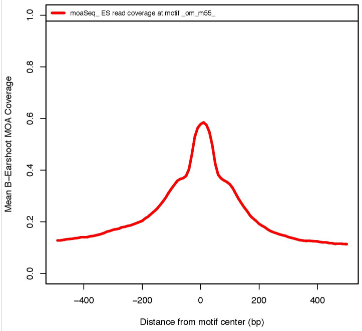	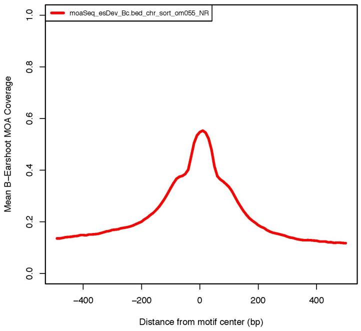	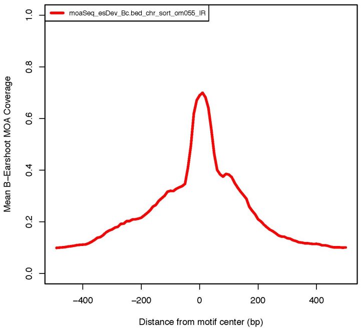

Motif Name	Total Number	In Repeats
om056	1,255	16%
Consensus	stACCGTAss	
LOGO	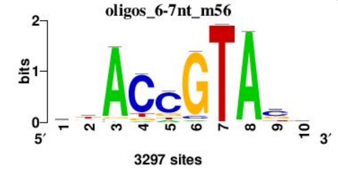	
LOGO RC	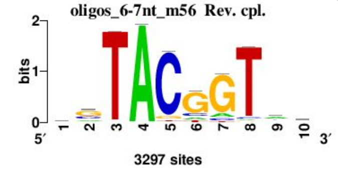	

	Frequency Distr. at TSS (FGS, B73v3)	Average Local Base Frequency (FGS, B73v3)
Median Position Relative to TSS =	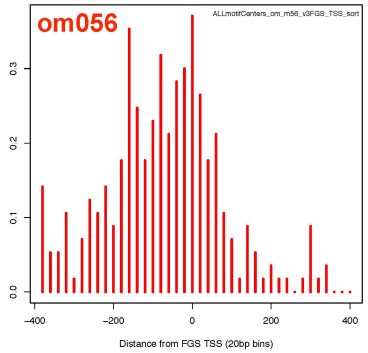	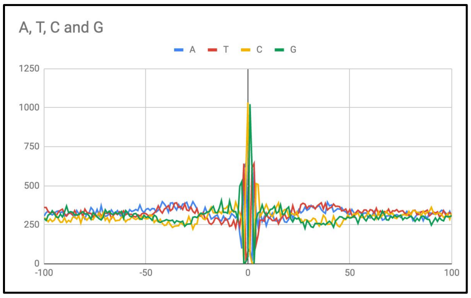
-126		

Average MOA Coverage Around Motif (RPM)

All	Not in Repeats (NR)	In Repeats (IR)
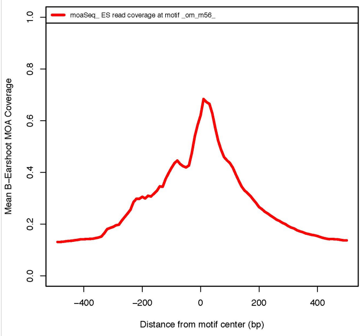	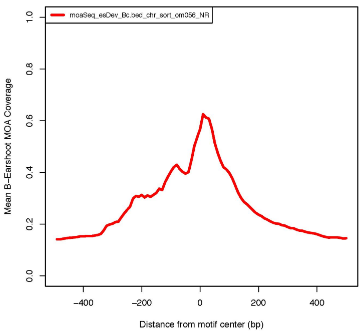	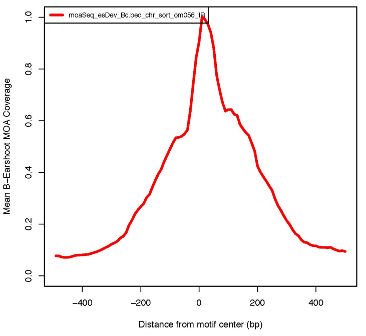

Motif Name	Total Number	In Repeats
om057	1,494	12%
Consensus	scCCAtCCCAcs	
LOGO	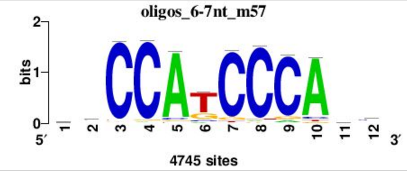	
LOGO RC	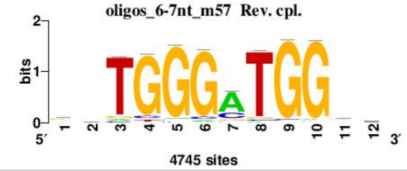	

	Frequency Distr. at TSS (FGS, B73v3)	Average Local Base Frequency (FGS, B73v3)
Median Position Relative to TSS = -72	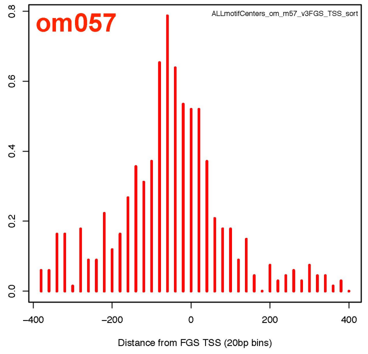	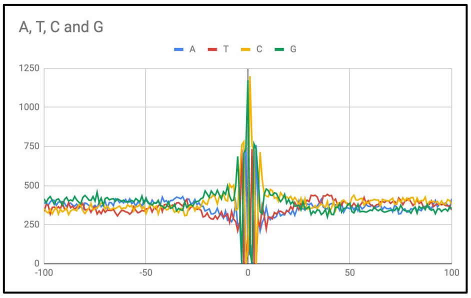

Average MOA Coverage Around Motif (RPM)

All	Not in Repeats (NR)	In Repeats (IR)
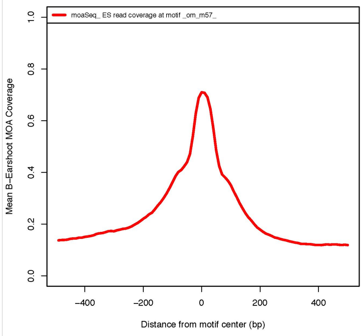	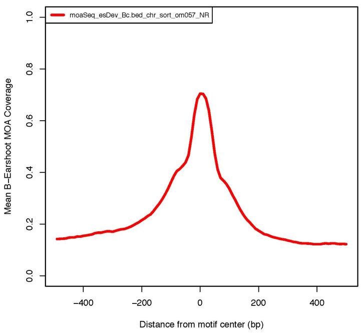	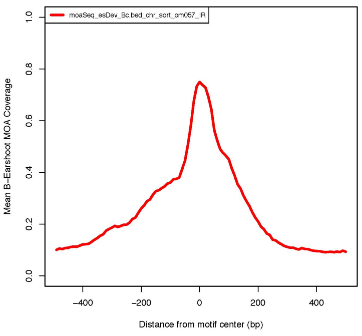

Motif Name	Total Number	In Repeats
om058	1,788	14%
Consensus	kcTAGCTAgm	
LOGO	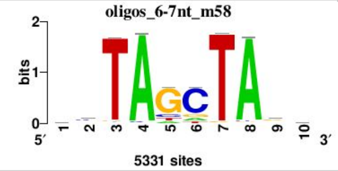	
LOGO RC	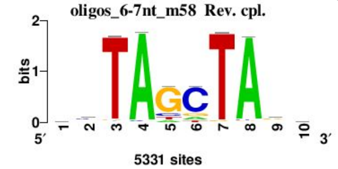	

	Frequency Distr. at TSS (FGS, B73v3)	Average Local Base Frequency (FGS, B73v3)
Median Position Relative to TSS = -106	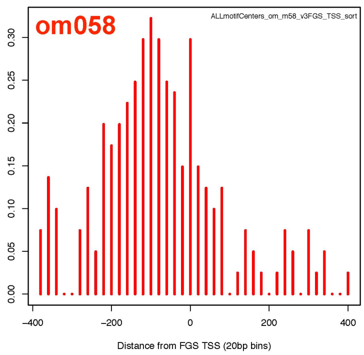	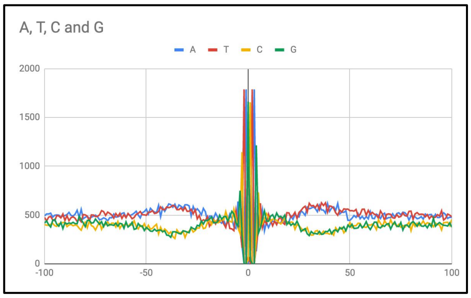

Average MOA Coverage Around Motif (RPM)

All	Not in Repeats (NR)	In Repeats (IR)
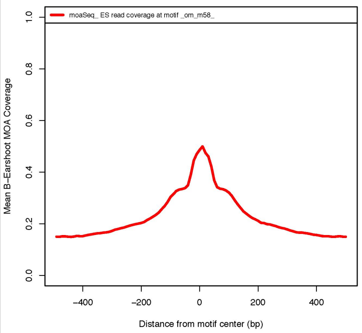	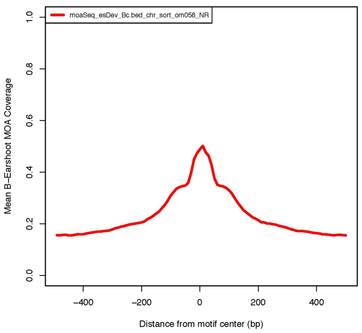	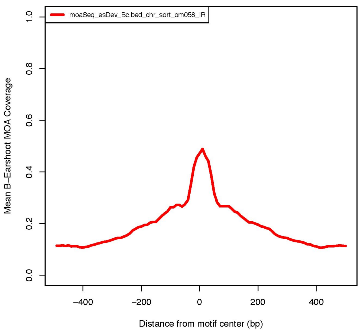

Motif Name	Total Number	In Repeats
om059	1,410	11%
Consensus	ymCTTGTCyy	
LOGO	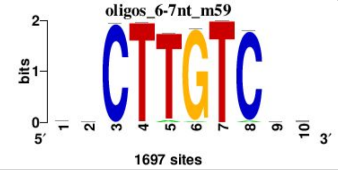	
LOGO RC	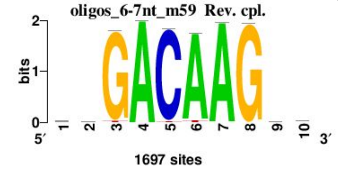	

	Frequency Distr. at TSS (FGS, B73v3)	Average Local Base Frequency (FGS, B73v3)
Median Position Relative to TSS =	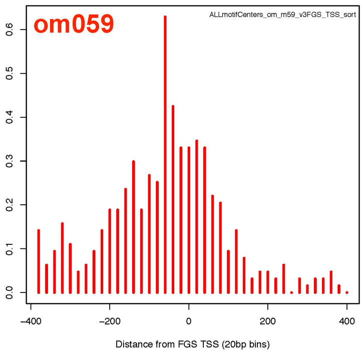	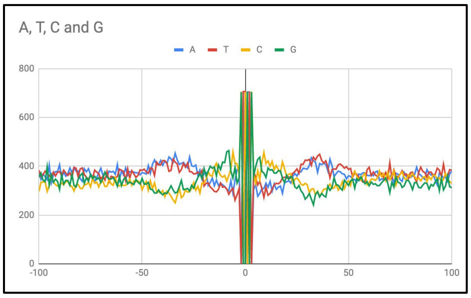
-62		

Average MOA Coverage Around Motif (RPM)

All	Not in Repeats (NR)	In Repeats (IR)
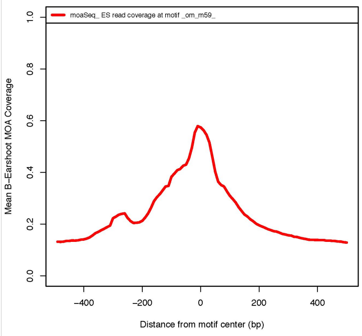	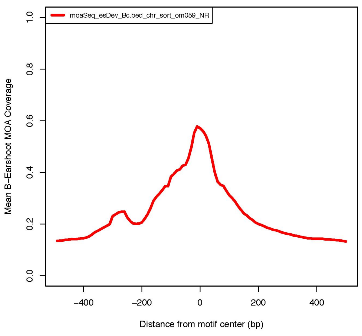	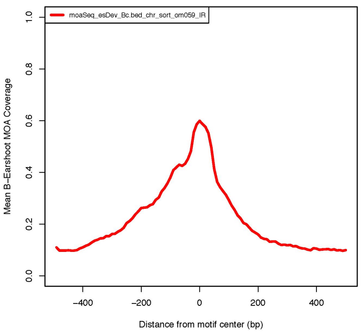

Motif Name	Total Number	In Repeats
om060	942	6%
Consensus	skGCTGTGCTgs	

LOGO

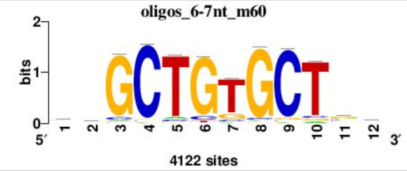

LOGO RC

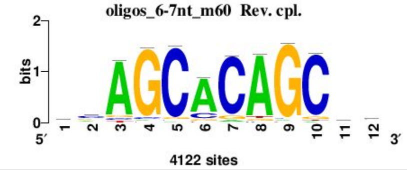

	Frequency Distr. at TSS (FGS, B73v3)	Average Local Base Frequency (FGS, B73v3)
Median Position Relative to TSS =		
-65		

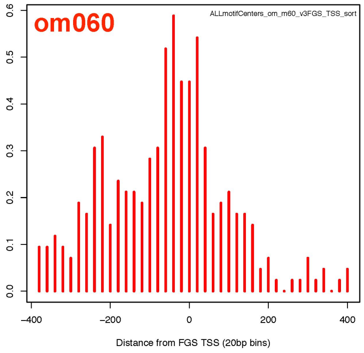

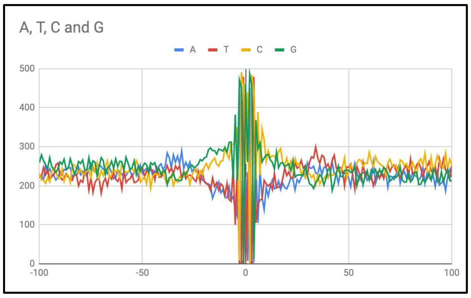

Average MOA Coverage Around Motif (RPM)

All	Not in Repeats (NR)	In Repeats (IR)

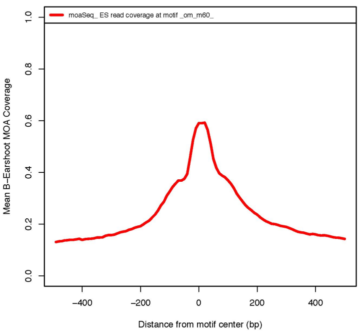

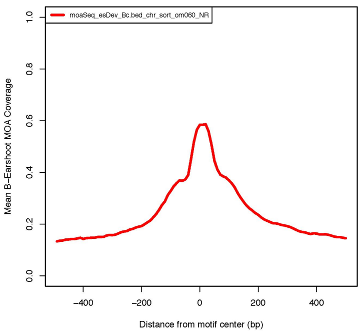

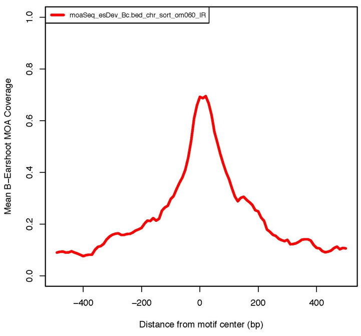

Motif Name	Total Number	In Repeats
om061	423	60%
Consensus	mtCTAATCTss	
LOGO	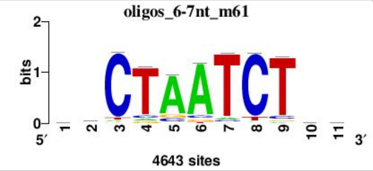	
LOGO RC	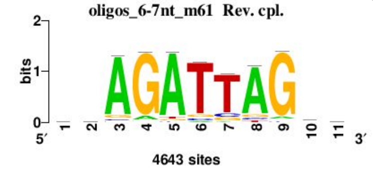	

	Frequency Distr. at TSS (FGS, B73v3)	Average Local Base Frequency (FGS, B73v3)
Median Position Relative to TSS =	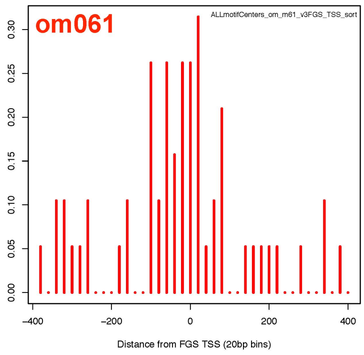	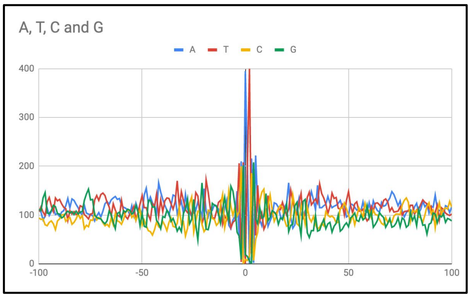
-30		

Average MOA Coverage Around Motif (RPM)

All	Not in Repeats (NR)	In Repeats (IR)
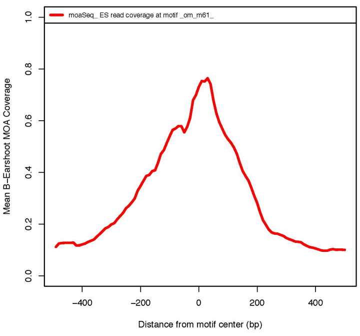	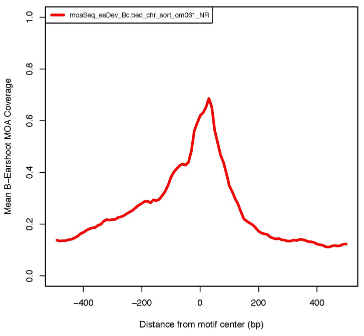	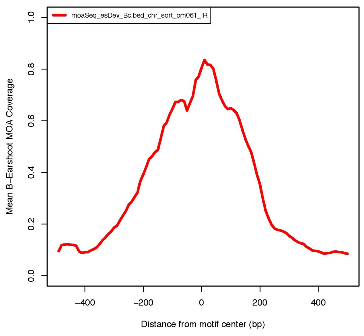

Motif Name	Total Number	In Repeats
om062	1,371	14%
Consensus	sgACCGGAcs	
LOGO	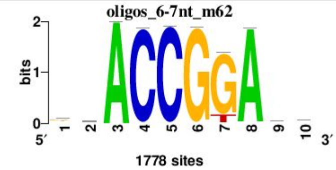	
LOGO RC	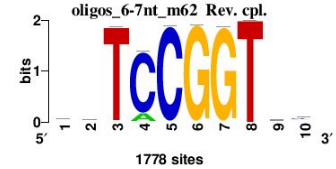	

	Frequency Distr. at TSS (FGS, B73v3)	Average Local Base Frequency (FGS, B73v3)
Median Position Relative to TSS =	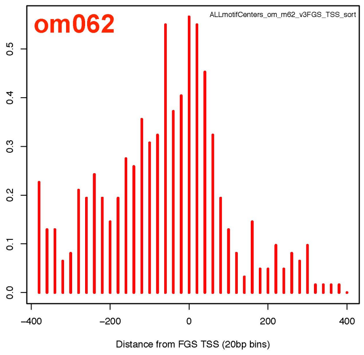	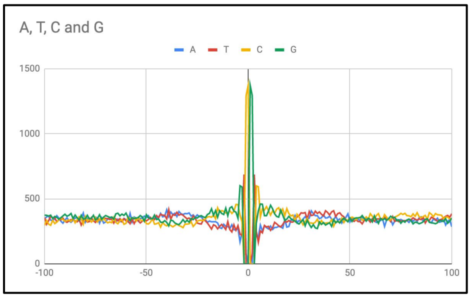
-79		

Average MOA Coverage Around Motif (RPM)

All	Not in Repeats (NR)	In Repeats (IR)
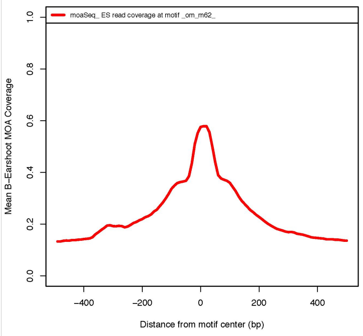	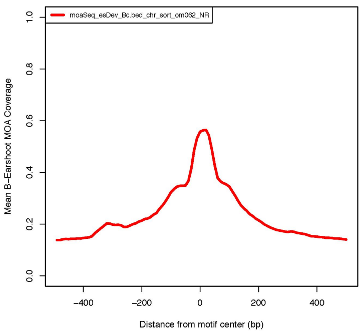	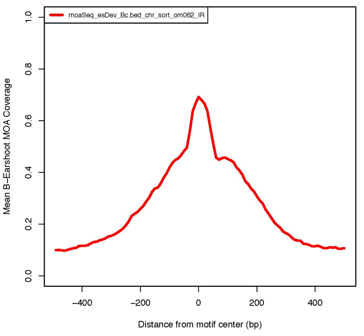

Motif Name	Total Number	In Repeats
om063	494	11%
Consensus	dsTACTGTAsy	
LOGO	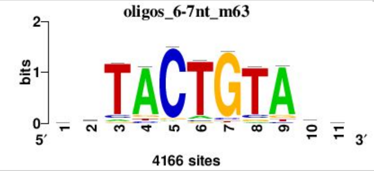	
LOGO RC	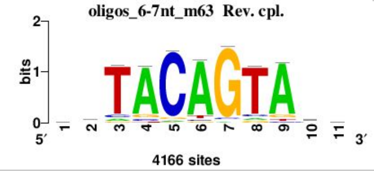	

	Frequency Distr. at TSS (FGS, B73v3)	Average Local Base Frequency (FGS, B73v3)
Median Position Relative to TSS =	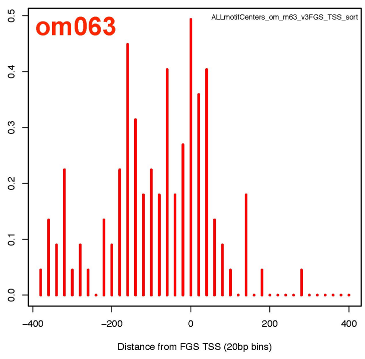	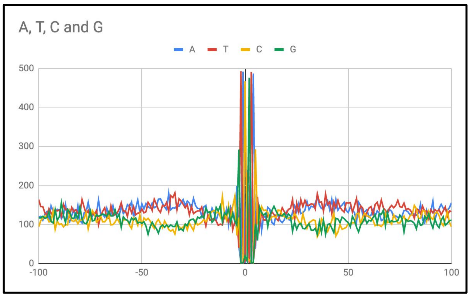
-125		

Average MOA Coverage Around Motif (RPM)

All	Not in Repeats (NR)	In Repeats (IR)
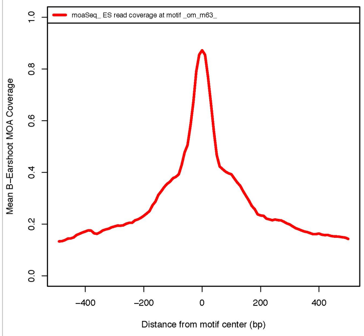	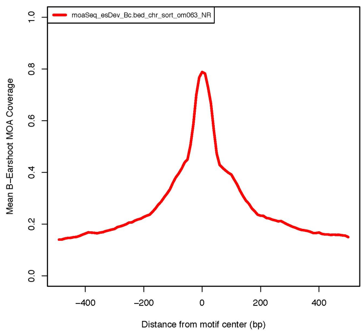	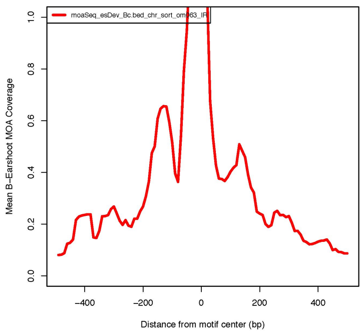

Motif Name	Total Number	In Repeats
om064	721	13%
Consensus	yyCCCTTCCsy	
LOGO	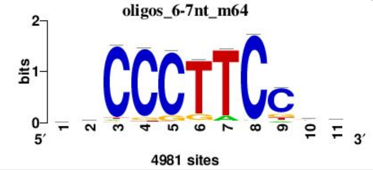	
LOGO RC	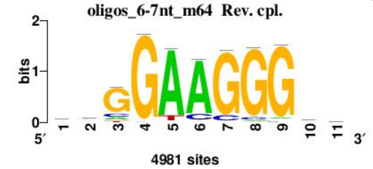	

	Frequency Distr. at TSS (FGS, B73v3)	Average Local Base Frequency (FGS, B73v3)
Median Position Relative to TSS =	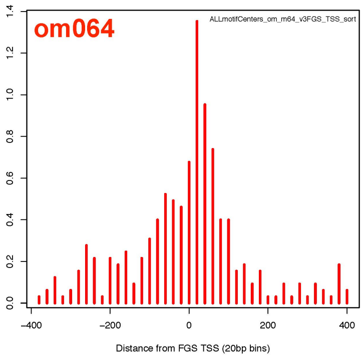	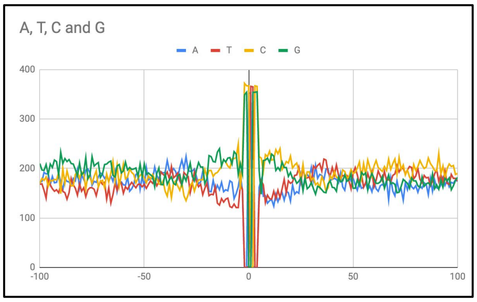
-13		

Average MOA Coverage Around Motif (RPM)

All	Not in Repeats (NR)	In Repeats (IR)
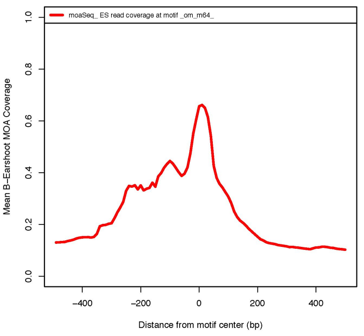	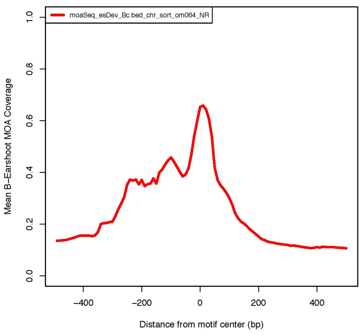	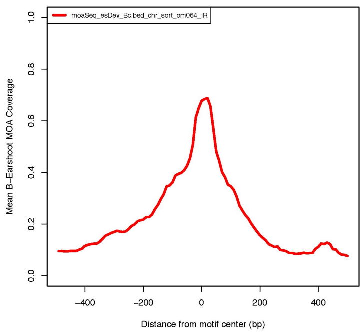

Motif Name	Total Number	In Repeats
om065	418	25%
Consensus	gcTGAACTGArs	
LOGO	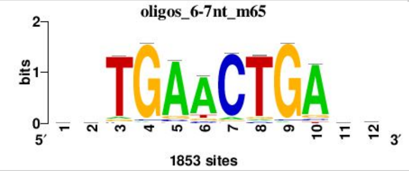	
LOGO RC	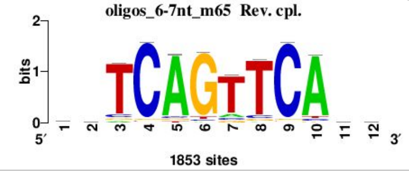	

	Frequency Distr. at TSS (FGS, B73v3)	Average Local Base Frequency (FGS, B73v3)
Median Position Relative to TSS = -33	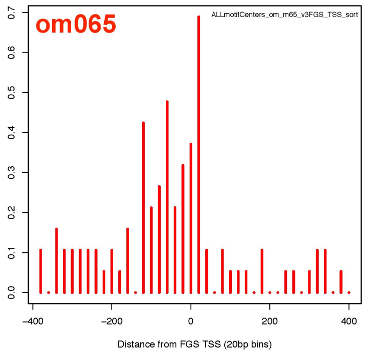	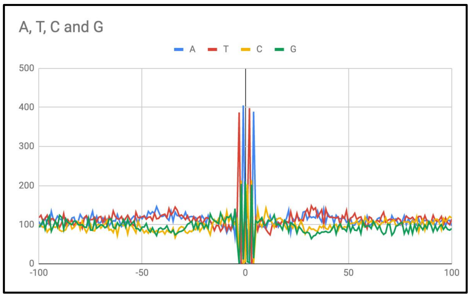

Average MOA Coverage Around Motif (RPM)

All	Not in Repeats (NR)	In Repeats (IR)
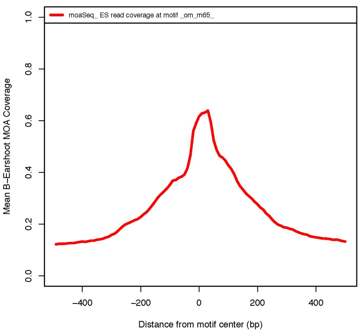	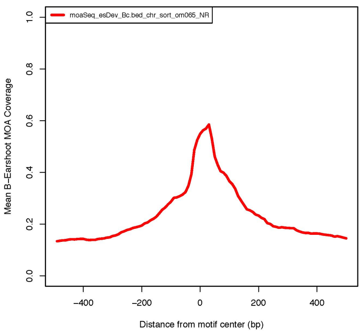	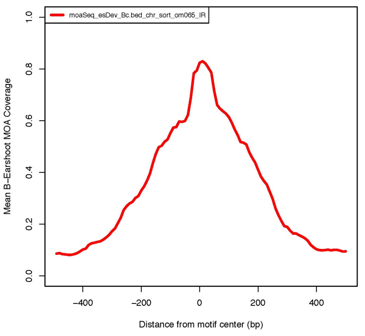

Motif Name	Total Number	In Repeats
om066	1,767	44%

Consensus	wtATTTGAth
LOGO	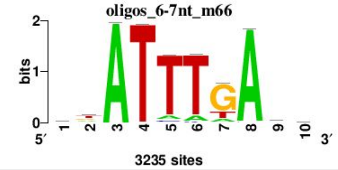
LOGO RC	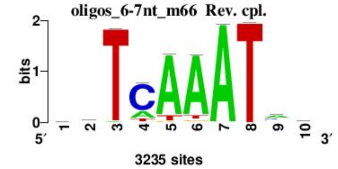

	Frequency Distr. at TSS (FGS, B73v3)	Average Local Base Frequency (FGS, B73v3)
Median Position Relative to TSS = -70	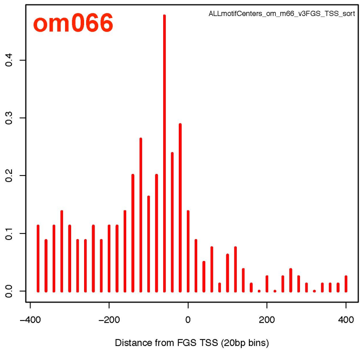	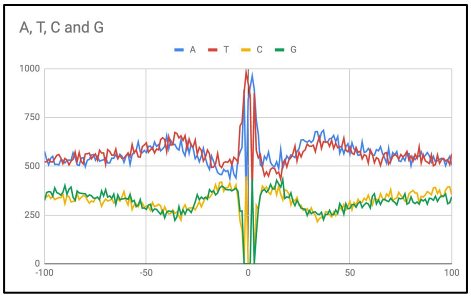

Average MOA Coverage Around Motif (RPM)

All	Not in Repeats (NR)	In Repeats (IR)
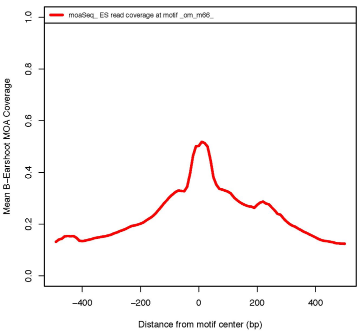	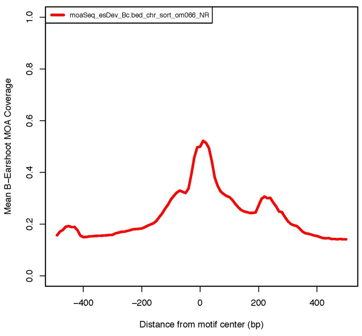	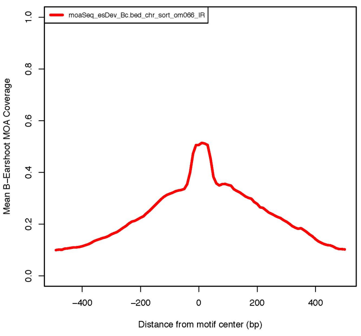

Motif Name	Total Number	In Repeats
om067	389	25%
Consensus	ssTCGGTTCss	

LOGO

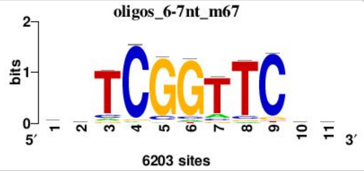

LOGO RC

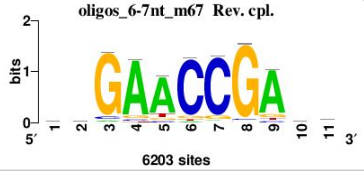

	Frequency Distr. at TSS (FGS, B73v3)	Average Local Base Frequency (FGS, B73v3)
Median Position Relative to TSS = -66		

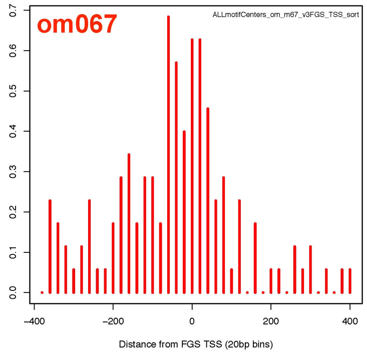

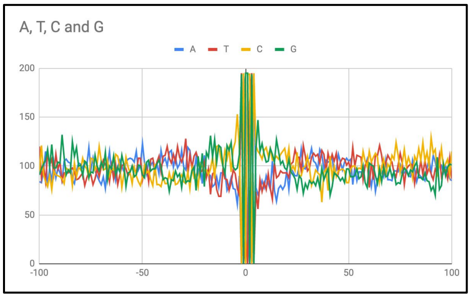

Average MOA Coverage Around Motif (RPM)

All	Not in Repeats (NR)	In Repeats (IR)

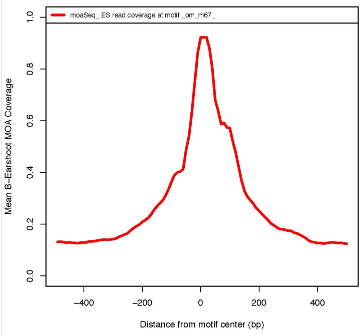

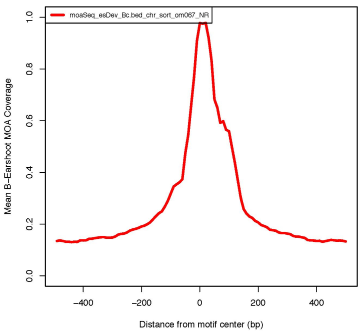

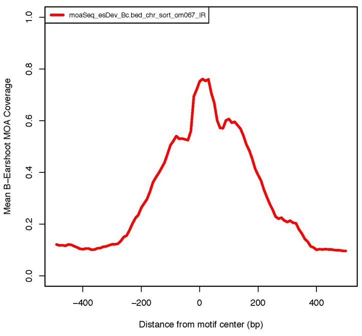

Motif Name	Total Number	In Repeats
om068	2,298	10%
Consensus	ssCCCGGCcs	
LOGO	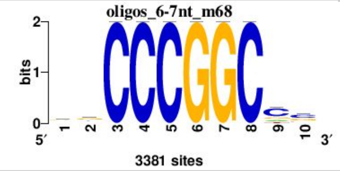	
LOGO RC	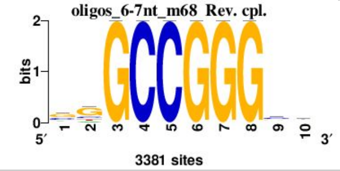	

	Frequency Distr. at TSS (FGS, B73v3)	Average Local Base Frequency (FGS, B73v3)
Median Position Relative to TSS =	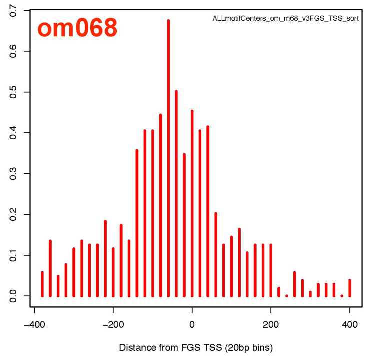	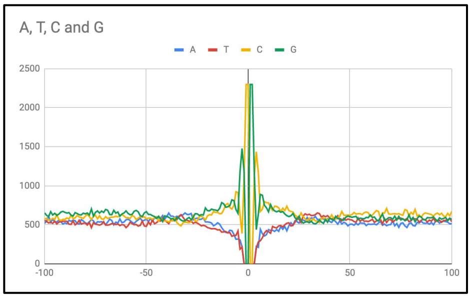
-110		

Average MOA Coverage Around Motif (RPM)

All	Not in Repeats (NR)	In Repeats (IR)
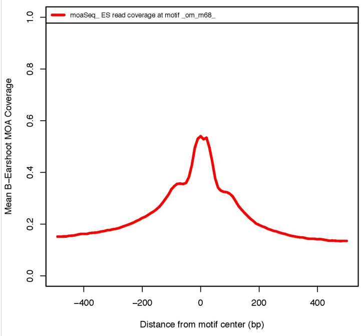	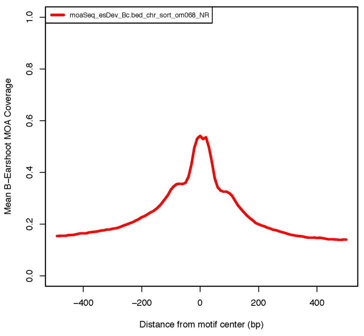	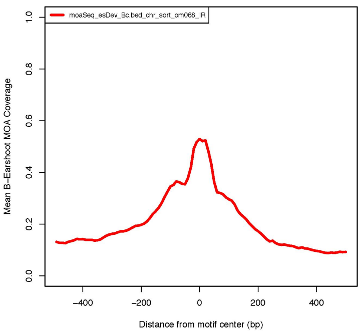

Motif Name	Total Number	In Repeats
om069	1,540	14%
Consensus	sgGTCCCAcc	
LOGO	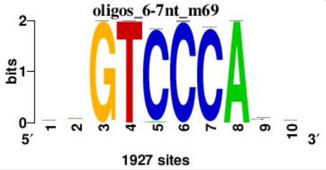	
LOGO RC	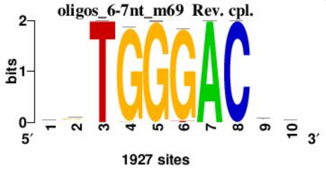	

	Frequency Distr. at TSS (FGS, B73v3)	Average Local Base Frequency (FGS, B73v3)
Median Position Relative to TSS =	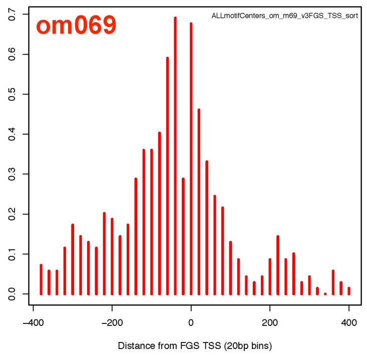	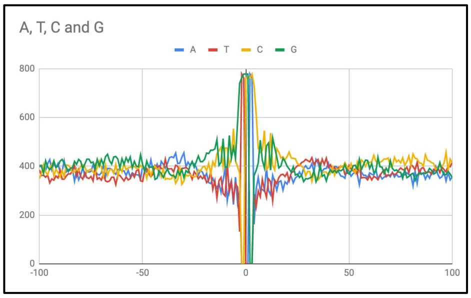
-80		

Average MOA Coverage Around Motif (RPM)

All	Not in Repeats (NR)	In Repeats (IR)
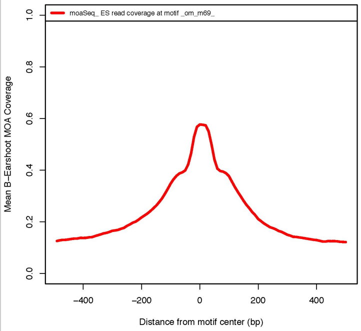	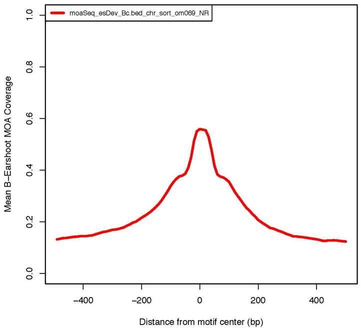	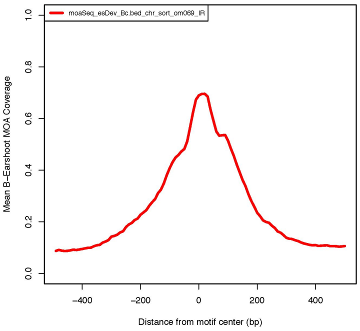

Motif Name	Total Number	In Repeats
om070	1,917	8%
Consensus	yaCGTACGtr	
LOGO	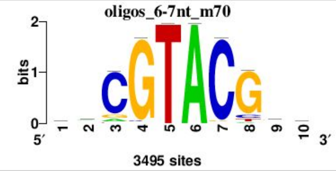	
LOGO RC	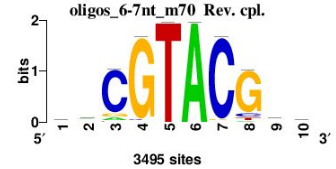	

	Frequency Distr. at TSS (FGS, B73v3)	Average Local Base Frequency (FGS, B73v3)
Median Position Relative to TSS =	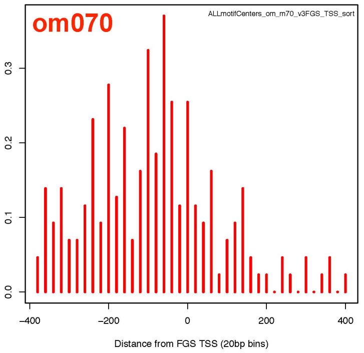	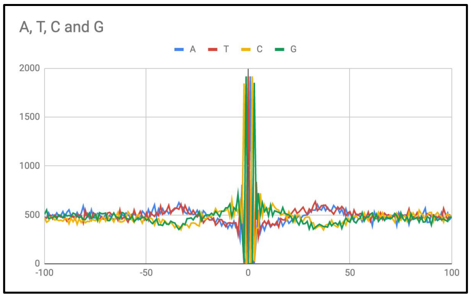
-206		

Average MOA Coverage Around Motif (RPM)

All	Not in Repeats (NR)	In Repeats (IR)
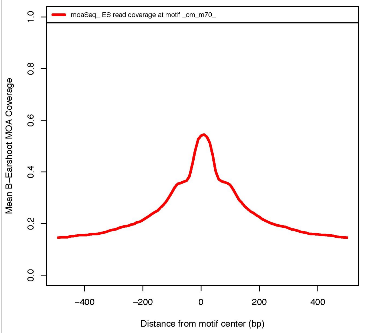	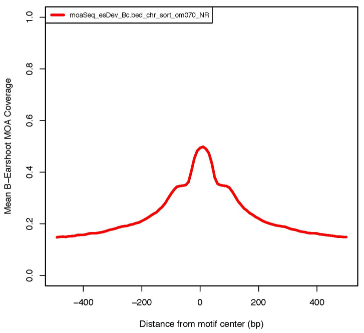	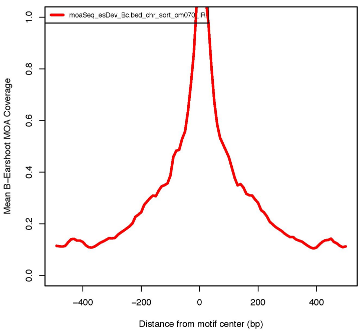

Motif Name	Total Number	In Repeats
om071	304	22%
Consensus	rvCGAAACGrv	
LOGO	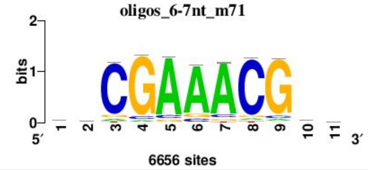	
LOGO RC	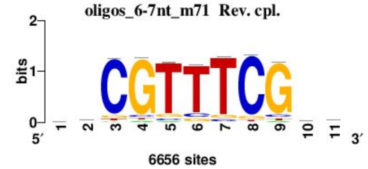	

	Frequency Distr. at TSS (FGS, B73v3)	Average Local Base Frequency (FGS, B73v3)
Median Position Relative to TSS =	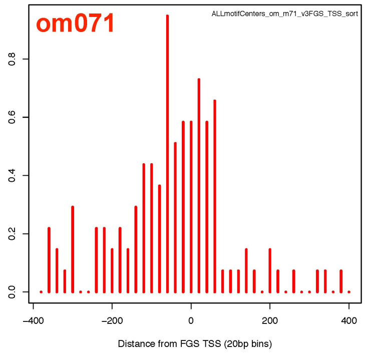	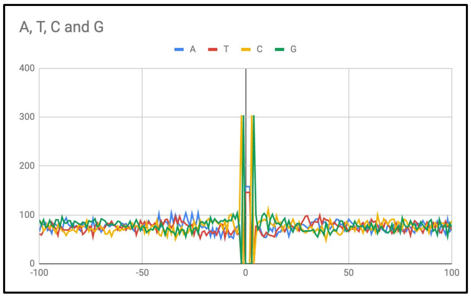
-79		

Average MOA Coverage Around Motif (RPM)		
All	Not in Repeats (NR)	In Repeats (IR)
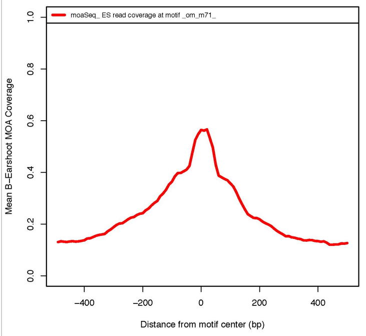	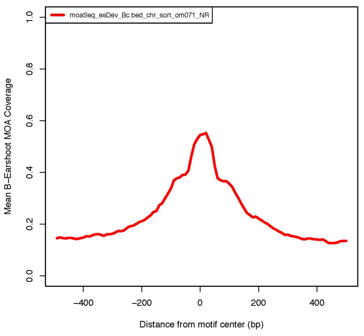	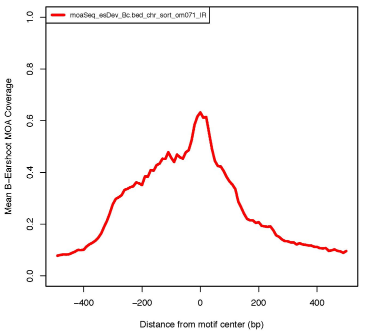

Motif Name	Total Number	In Repeats
om072	1,445	40%
Consensus	ttAcTTTAyw	
LOGO	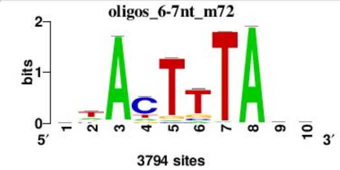	
LOGO RC	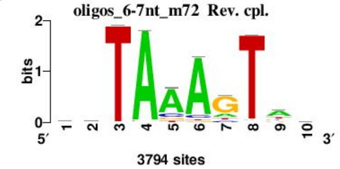	

	Frequency Distr. at TSS (FGS, B73v3)	Average Local Base Frequency (FGS, B73v3)
Median Position Relative to TSS = -113	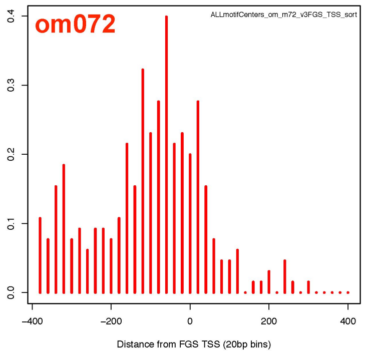	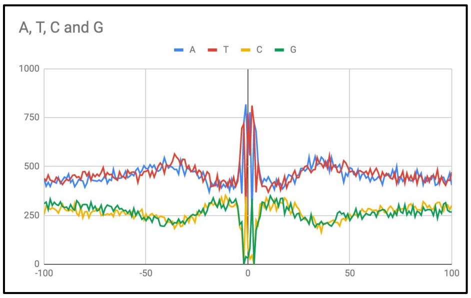

Average MOA Coverage Around Motif (RPM)

All	Not in Repeats (NR)	In Repeats (IR)
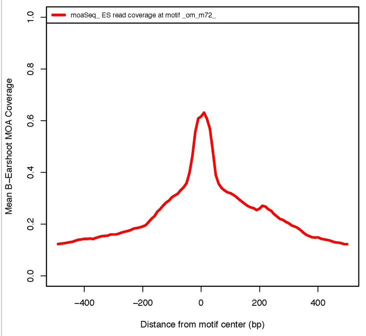	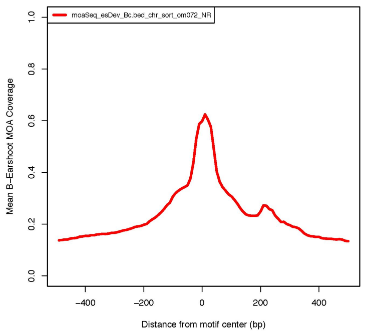	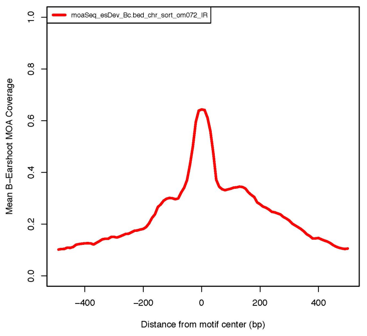

Motif Name	Total Number	In Repeats
om073	1,085	28%
Consensus	mtATTCCAkm	

LOGO

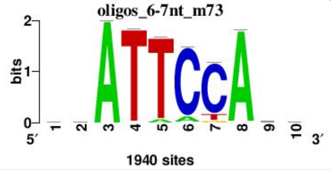

LOGO RC

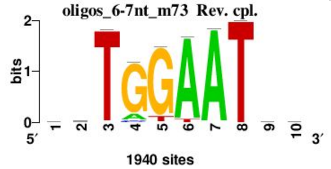

	Frequency Distr. at TSS (FGS, B73v3)	Average Local Base Frequency (FGS, B73v3)
Median Position Relative to TSS = -66	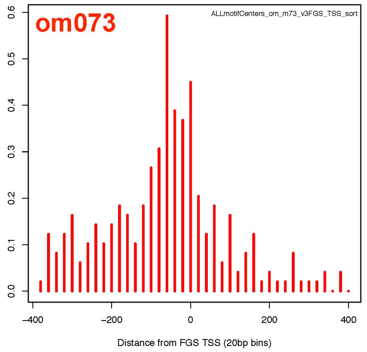	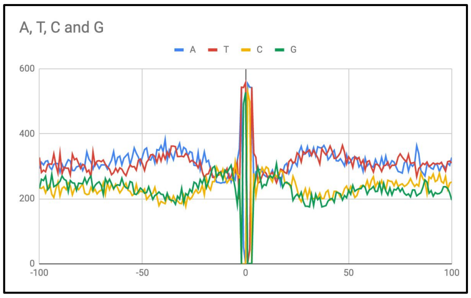

Average MOA Coverage Around Motif (RPM)

All	Not in Repeats (NR)	In Repeats (IR)
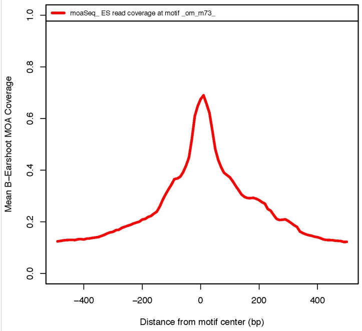	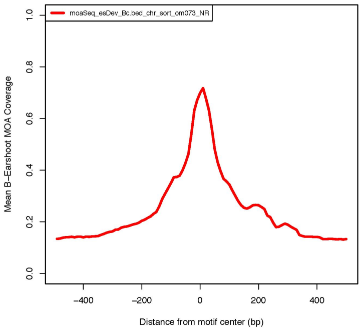	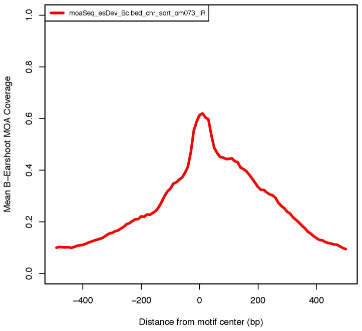

Motif Name	Total Number	In Repeats
om074	5,028	24%
Consensus	aaAAmAAAArAAAAgrr	
LOGO	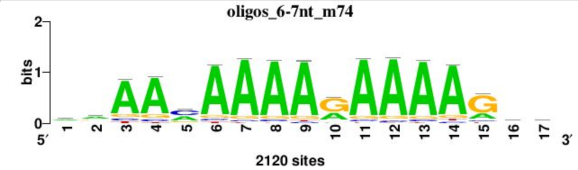	
LOGO RC	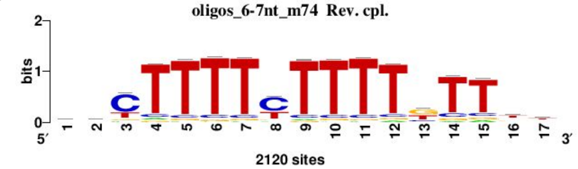	

	Frequency Distr. at TSS (FGS, B73v3)	Average Local Base Frequency (FGS, B73v3)
Median Position Relative to TSS =	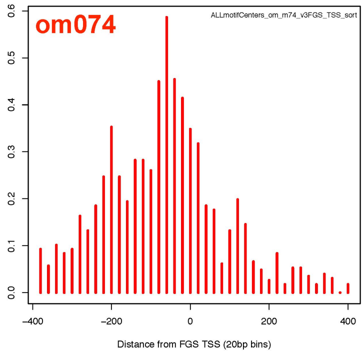	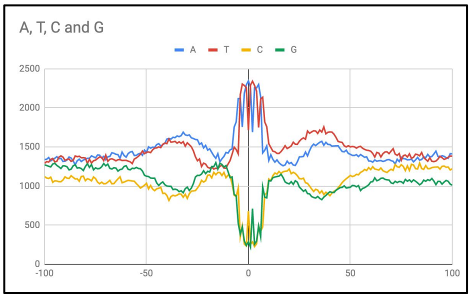
-78		

Average MOA Coverage Around Motif (RPM)

All	Not in Repeats (NR)	In Repeats (IR)
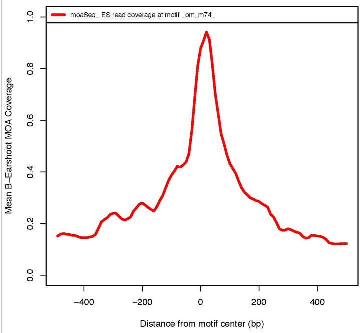	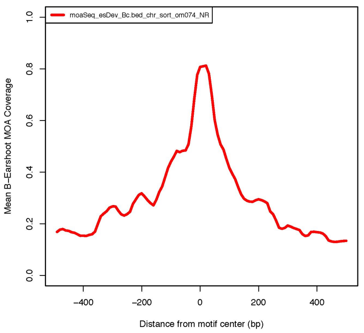	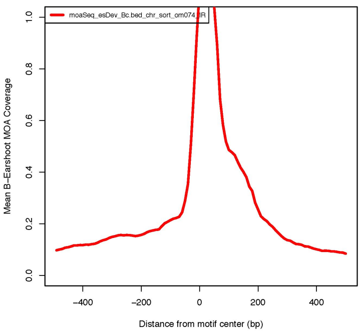

Motif Name	Total Number	In Repeats
om075	2,048	13%
Consensus	vgCGAGCGvs	
LOGO	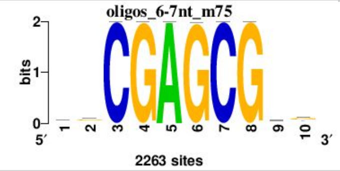	
LOGO RC	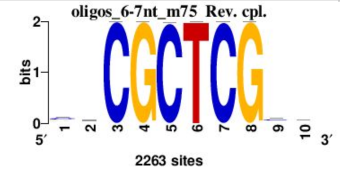	

	Frequency Distr. at TSS (FGS, B73v3)	Average Local Base Frequency (FGS, B73v3)
Median Position Relative to TSS = -90	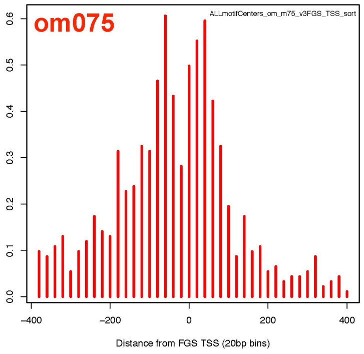	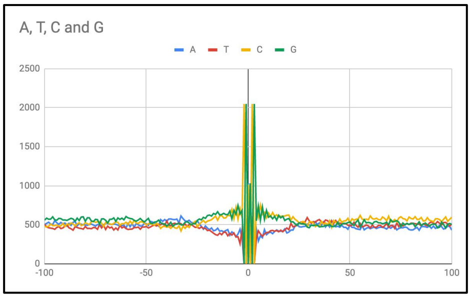

Average MOA Coverage Around Motif (RPM)

All	Not in Repeats (NR)	In Repeats (IR)
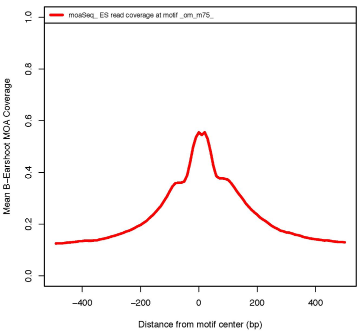	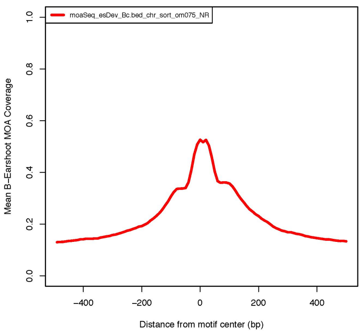	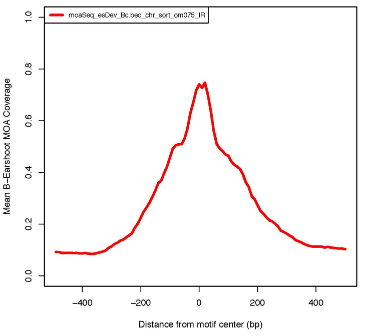

Motif Name	Total Number	In Repeats
om076	1,214	13%
Consensus	sbACGCGAss	
LOGO	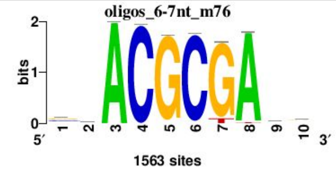	
LOGO RC	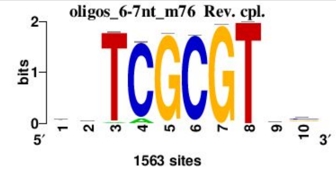	

	Frequency Distr. at TSS (FGS, B73v3)	Average Local Base Frequency (FGS, B73v3)
Median Position Relative to TSS =	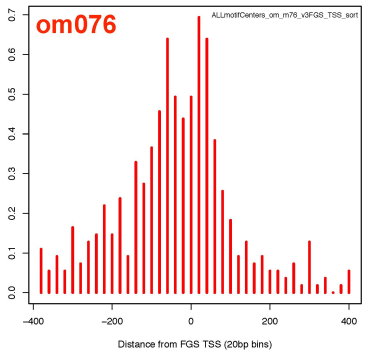	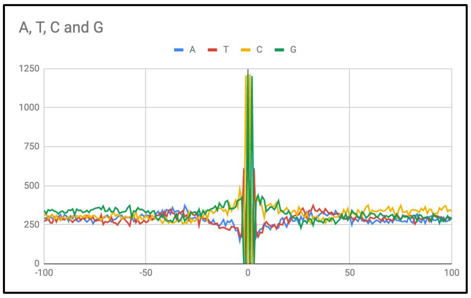
-63		

Average MOA Coverage Around Motif (RPM)

All	Not in Repeats (NR)	In Repeats (IR)
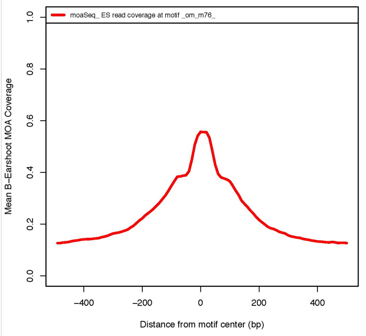	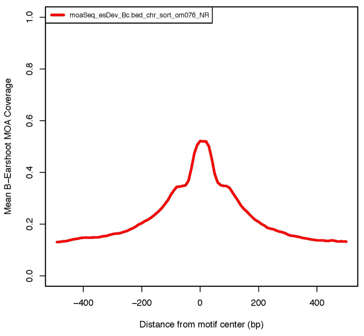	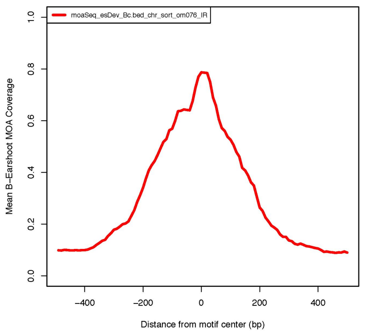

Motif Name	Total Number	In Repeats
**om077**	**745**	**17%**
**Consensus**	sctCAGCTCAGCCcv	
**LOGO**	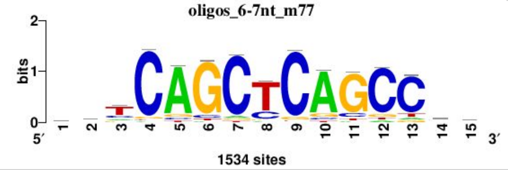	
**LOGO RC**	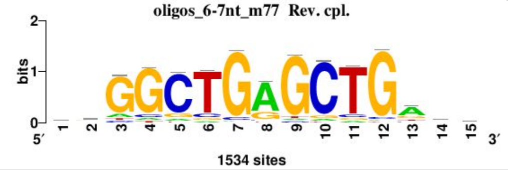	

	Frequency Distr. at TSS (FGS, B73v3)	Average Local Base Frequency (FGS, B73v3)
Median Position Relative to TSS =	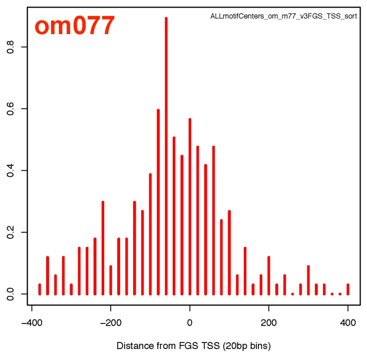	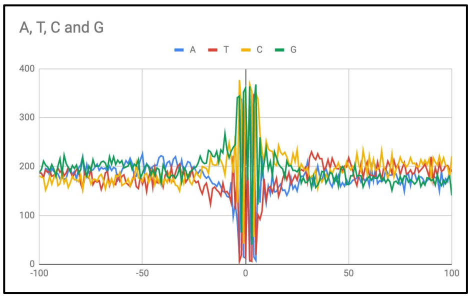
**-57**		

Average MOA Coverage Around Motif (RPM)

All	Not in Repeats (NR)	In Repeats (IR)
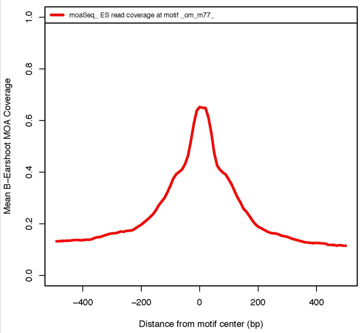	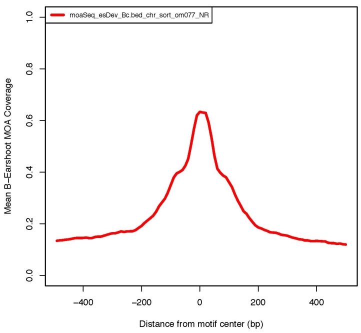	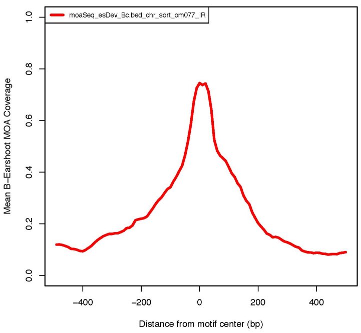

Motif Name	Total Number	In Repeats
om078	762	31%
Consensus	ctAGTCTAbr	
LOGO	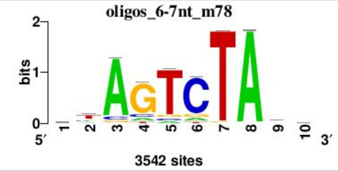	
LOGO RC	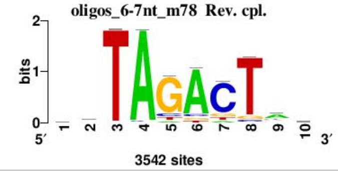	

	Frequency Distr. at TSS (FGS, B73v3)	Average Local Base Frequency (FGS, B73v3)
Median Position Relative to TSS =	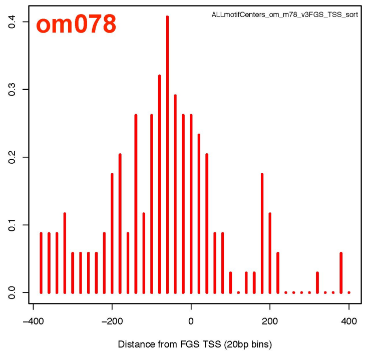	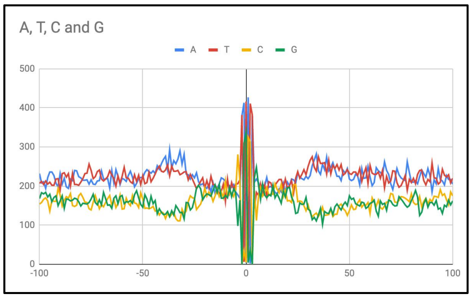
-75		

Average MOA Coverage Around Motif (RPM)

All	Not in Repeats (NR)	In Repeats (IR)
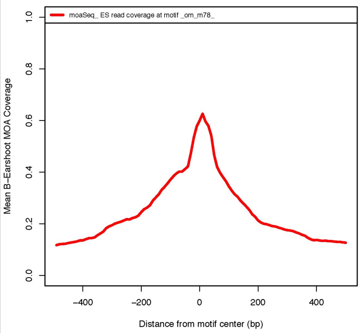	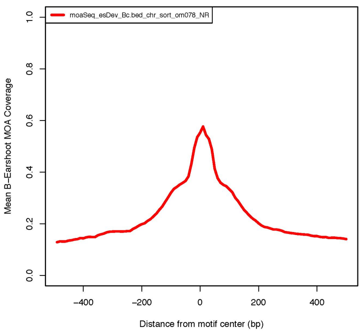	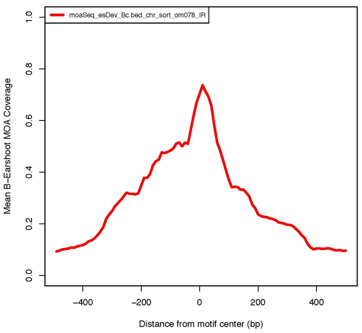

Motif Name	Total Number	In Repeats
om079	3,108	10%
Consensus	mcCACCACcm	
LOGO	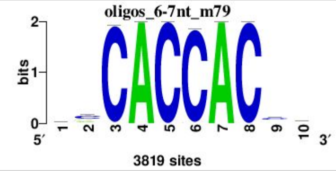	
LOGO RC	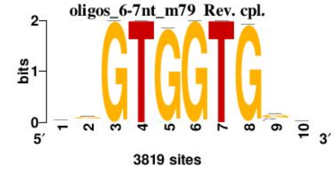	

	Frequency Distr. at TSS (FGS, B73v3)	Average Local Base Frequency (FGS, B73v3)
Median Position Relative to TSS =	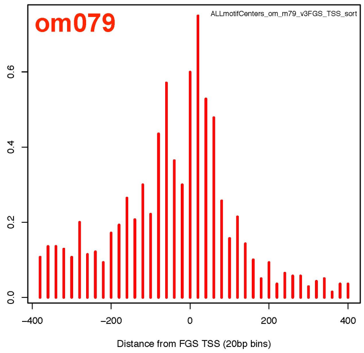	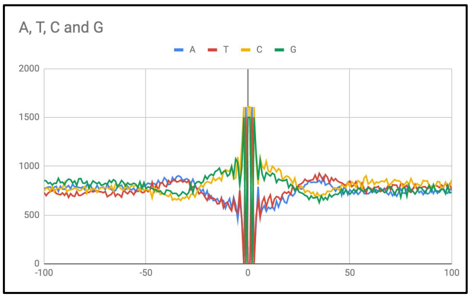
-57		

Average MOA Coverage Around Motif (RPM)

All	Not in Repeats (NR)	In Repeats (IR)
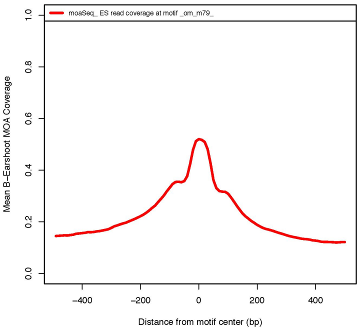	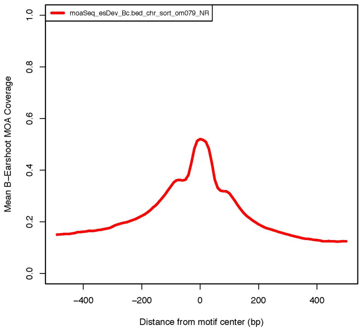	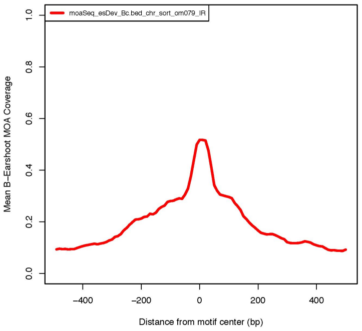

Motif Name	Total Number	In Repeats
om080	589	24%
Consensus	gbATGTGATGTrr	
LOGO	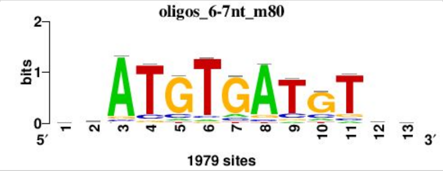	
LOGO RC	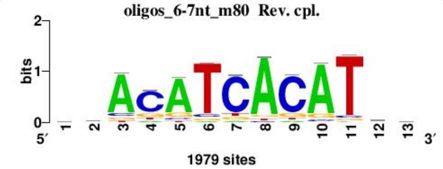	

	Frequency Distr. at TSS (FGS, B73v3)	Average Local Base Frequency (FGS, B73v3)
Median Position Relative to TSS = -75	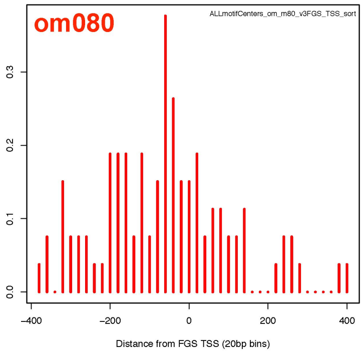	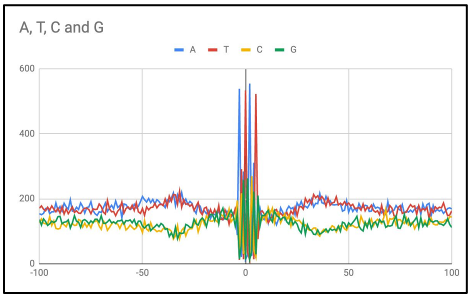

Average MOA Coverage Around Motif (RPM)

All	Not in Repeats (NR)	In Repeats (IR)
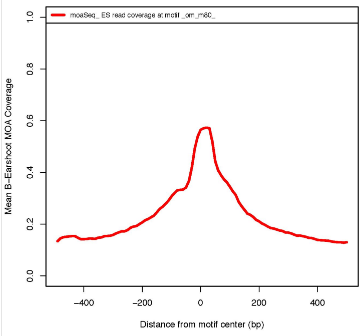	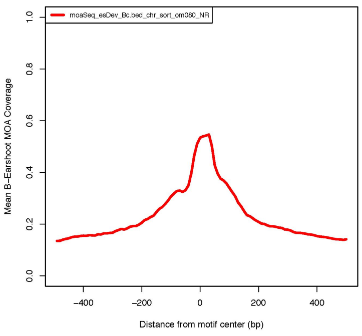	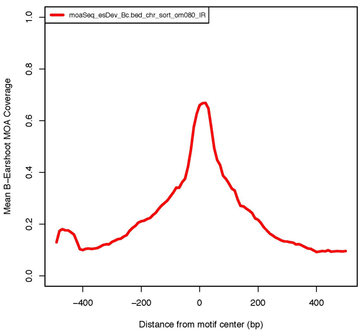

Motif Name	Total Number	In Repeats
om081	1,917	10%
Consensus	vcAGGCCAsv	
LOGO	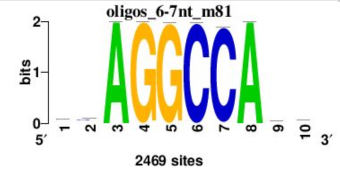	
LOGO RC	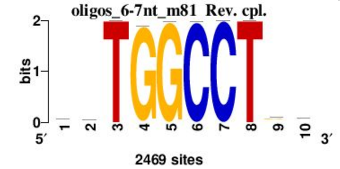	

	Frequency Distr. at TSS (FGS, B73v3)	Average Local Base Frequency (FGS, B73v3)
Median Position Relative to TSS = -92	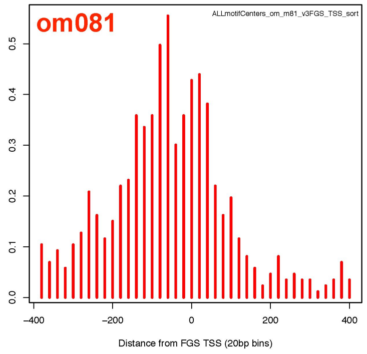	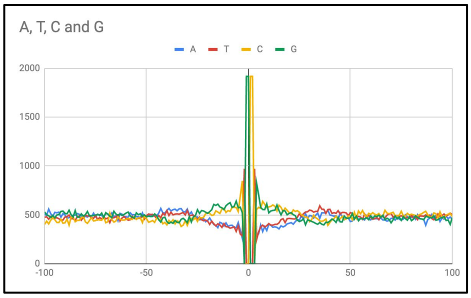

Average MOA Coverage Around Motif (RPM)

All	Not in Repeats (NR)	In Repeats (IR)
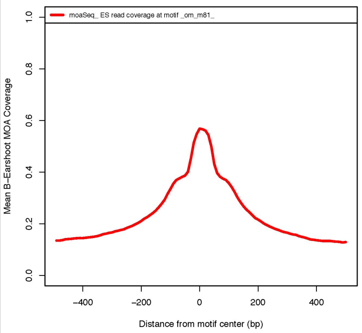	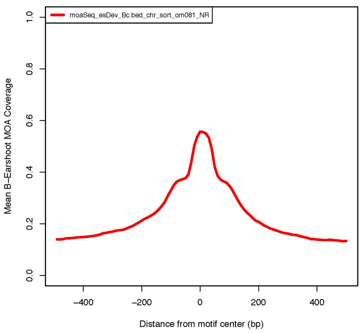	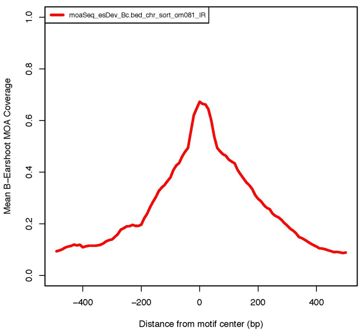

Motif Name	Total Number	In Repeats
om082	522	61%
Consensus	mvAATCAACss	
LOGO	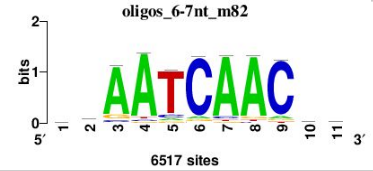	
LOGO RC	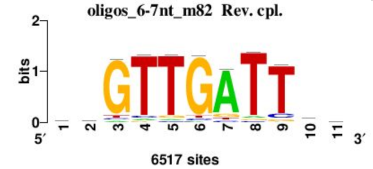	

	Frequency Distr. at TSS (FGS, B73v3)	Average Local Base Frequency (FGS, B73v3)
Median Position Relative to TSS =	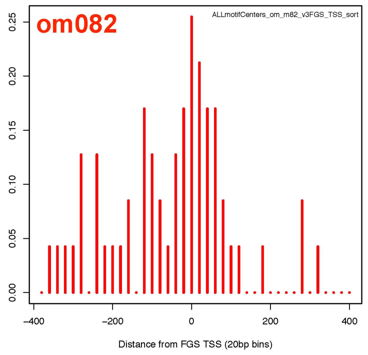	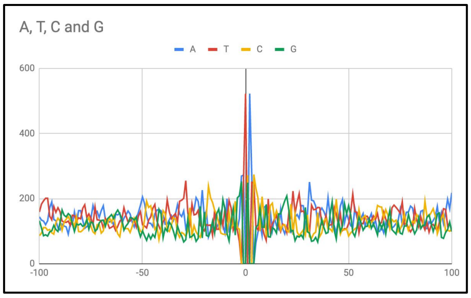
-9		

Average MOA Coverage Around Motif (RPM)

All	Not in Repeats (NR)	In Repeats (IR)
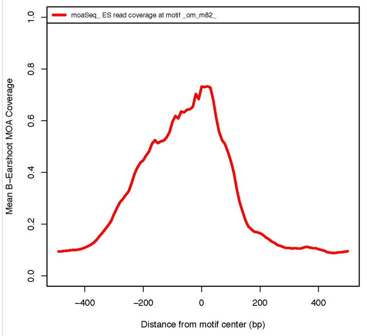	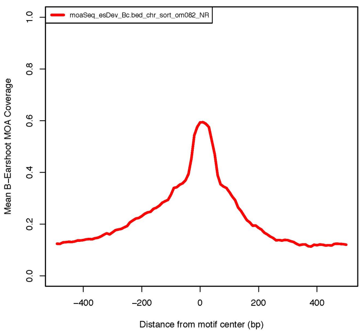	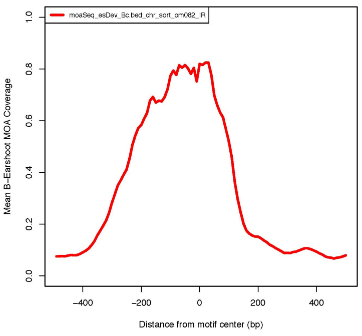

Motif Name	Total Number	In Repeats
om083	361	7%

Consensus	ssACGGAACss
LOGO	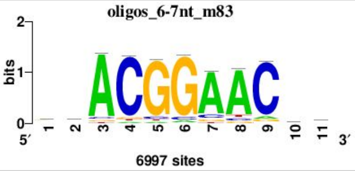
LOGO RC	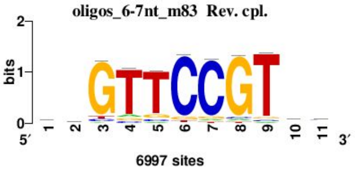

	Frequency Distr. at TSS (FGS, B73v3)	Average Local Base Frequency (FGS, B73v3)
Median Position Relative to TSS = -71	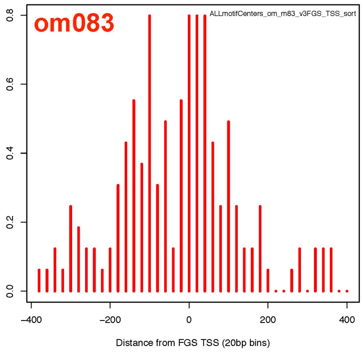	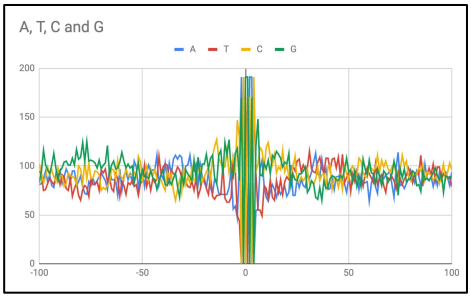

Average MOA Coverage Around Motif (RPM)

All	Not in Repeats (NR)	In Repeats (IR)
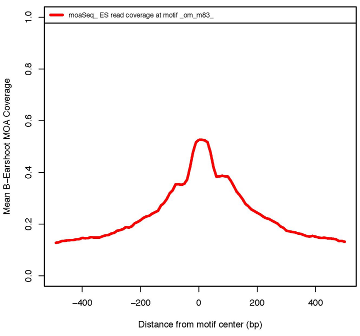	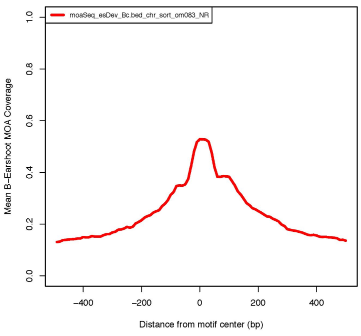	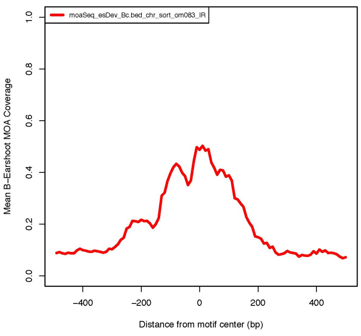

Motif Name	Total Number	In Repeats
**om084**	**262**	**21%**
**Consensus**	ssTAGCCTAsc	
**LOGO**	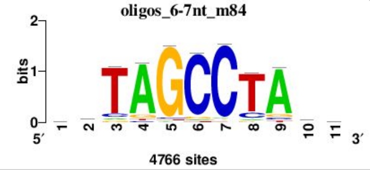	
**LOGO RC**	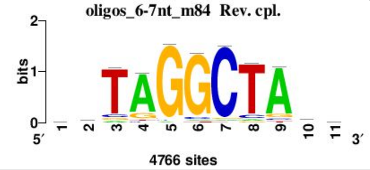	

	Frequency Distr. at TSS (FGS, B73v3)	Average Local Base Frequency (FGS, B73v3)
**Median Position Relative to TSS =**	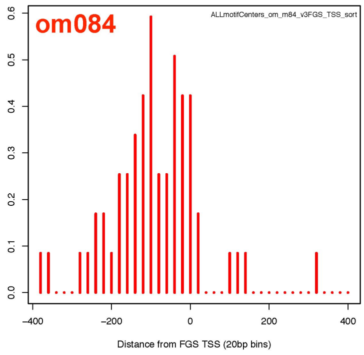	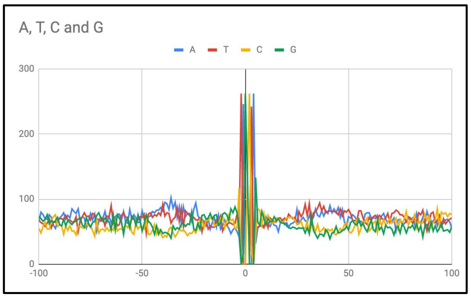
**-239**		

Average MOA Coverage Around Motif (RPM)

All	Not in Repeats (NR)	In Repeats (IR)
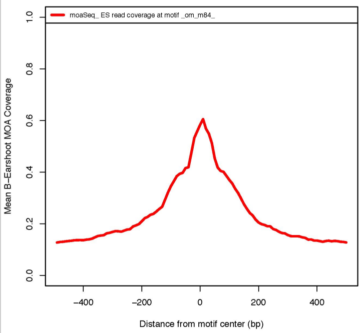	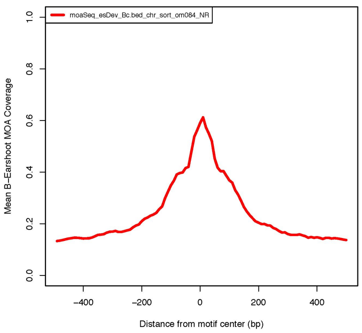	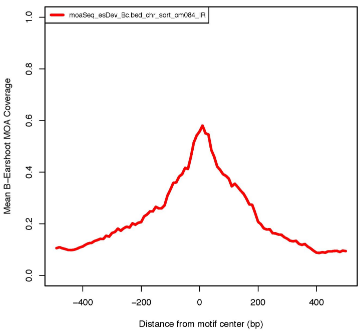

Motif Name	Total Number	In Repeats
om085	449	13%
Consensus	ssCTTGGCTss	
LOGO	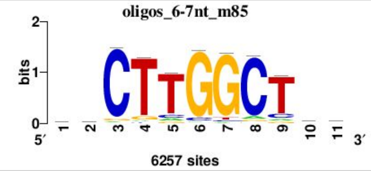	
LOGO RC	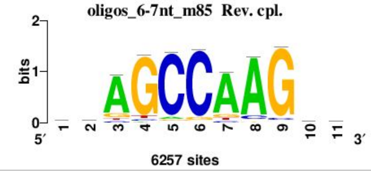	

	Frequency Distr. at TSS (FGS, B73v3)	Average Local Base Frequency (FGS, B73v3)
Median Position Relative to TSS =	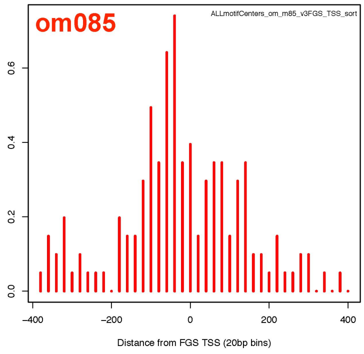	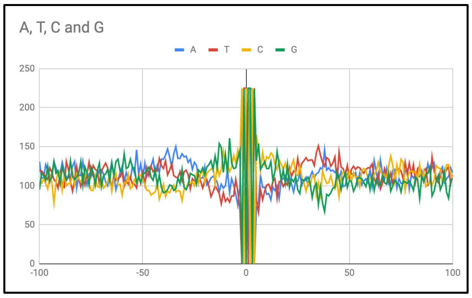
-46		

Average MOA Coverage Around Motif (RPM)

All	Not in Repeats (NR)	In Repeats (IR)
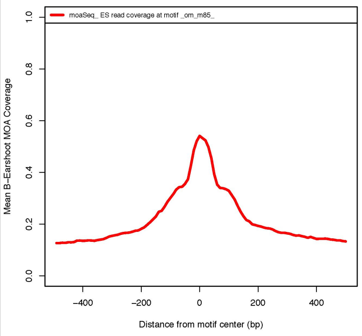	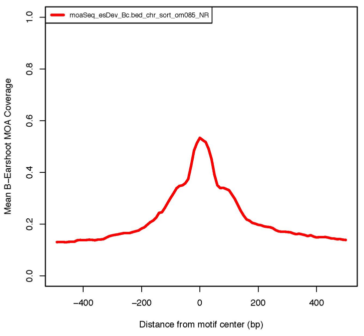	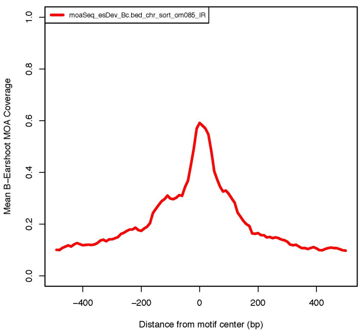

Motif Name	Total Number	In Repeats
om086	1,062	12%
Consensus	ysCACTTCACyb	
LOGO	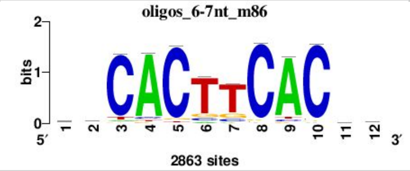	
LOGO RC	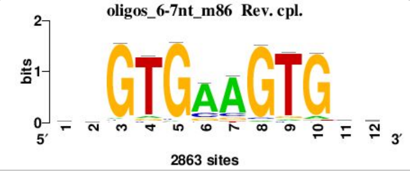	

	Frequency Distr. at TSS (FGS, B73v3)	Average Local Base Frequency (FGS, B73v3)
Median Position Relative to TSS =	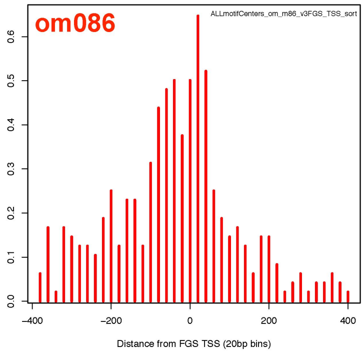	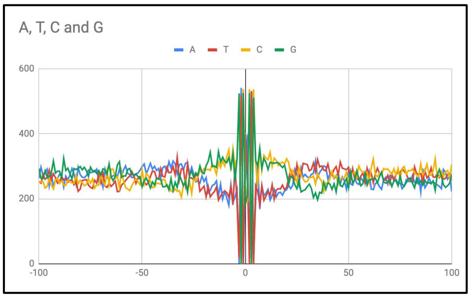
-48		

Average MOA Coverage Around Motif (RPM)

All	Not in Repeats (NR)	In Repeats (IR)
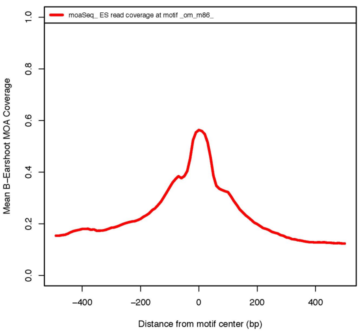	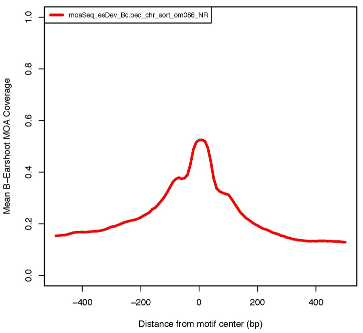	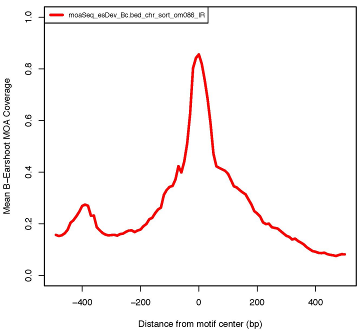

Motif Name	Total Number	In Repeats
om087	568	24%
Consensus	wwCTTATCya	
LOGO	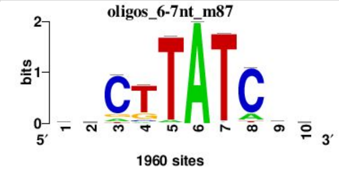	
LOGO RC	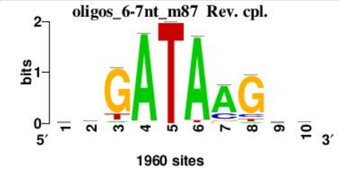	

	Frequency Distr. at TSS (FGS, B73v3)	Average Local Base Frequency (FGS, B73v3)
Median Position Relative to TSS = -27	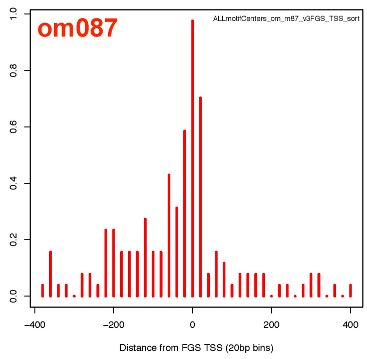	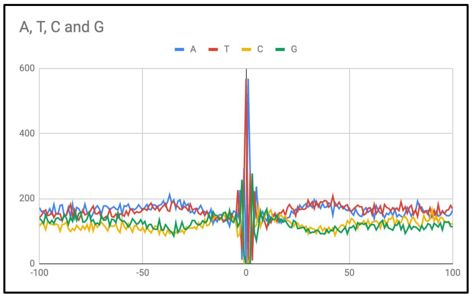

Average MOA Coverage Around Motif (RPM)

All	Not in Repeats (NR)	In Repeats (IR)
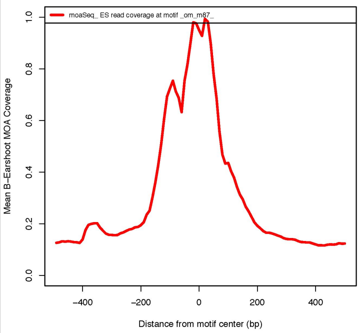	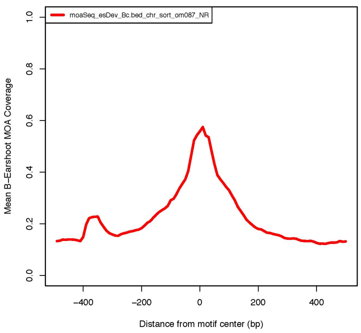	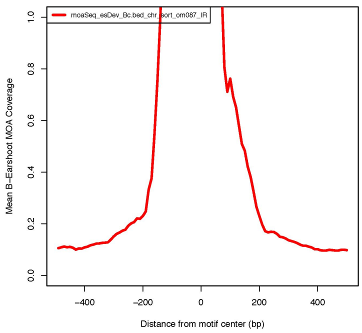

Motif Name	Total Number	In Repeats
om088	1,275	17%
Consensus	vtAGTACAbr	
LOGO	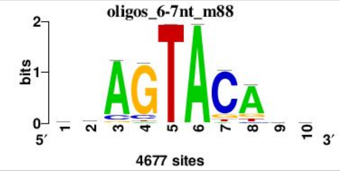	
LOGO RC	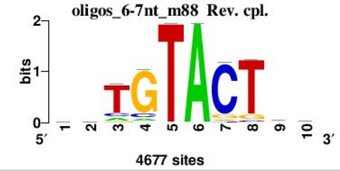	

	Frequency Distr. at TSS (FGS, B73v3)	Average Local Base Frequency (FGS, B73v3)
Median Position Relative to TSS =	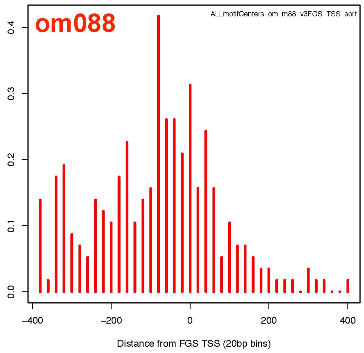	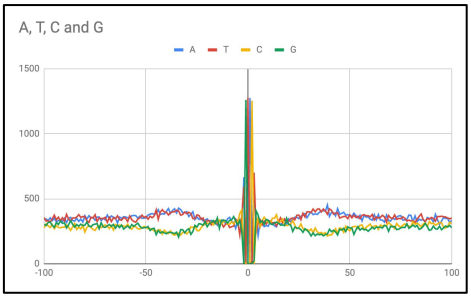
-101		

Average MOA Coverage Around Motif (RPM)

All	Not in Repeats (NR)	In Repeats (IR)
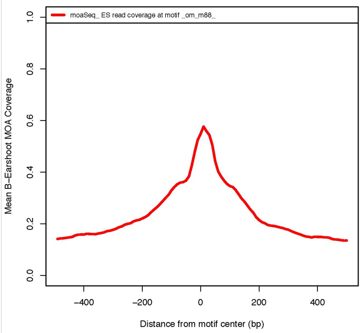	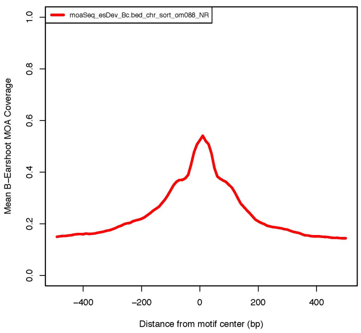	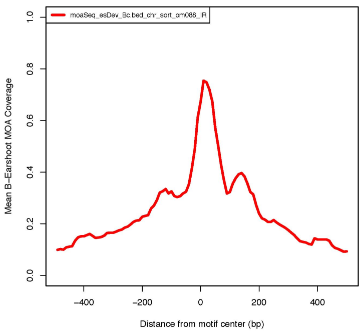

Motif Name	Total Number	In Repeats
om089	674	26%
Consensus	cyGCAACGCAss	
LOGO	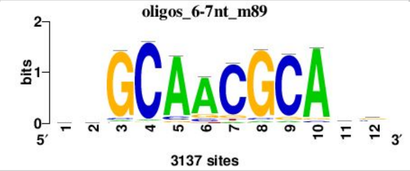	
LOGO RC	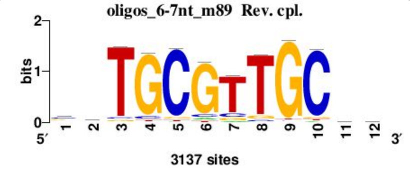	

	Frequency Distr. at TSS (FGS, B73v3)	Average Local Base Frequency (FGS, B73v3)
Median Position Relative to TSS =	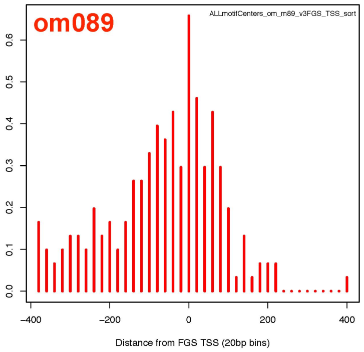	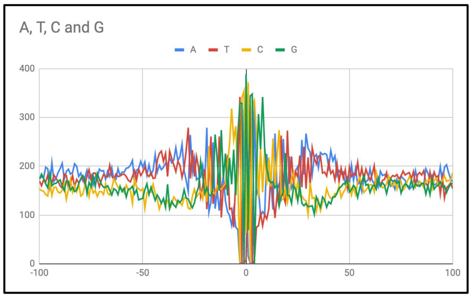
-40		

Average MOA Coverage Around Motif (RPM)

All	Not in Repeats (NR)	In Repeats (IR)
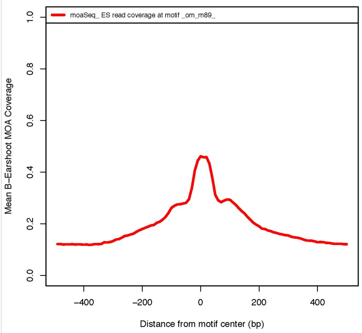	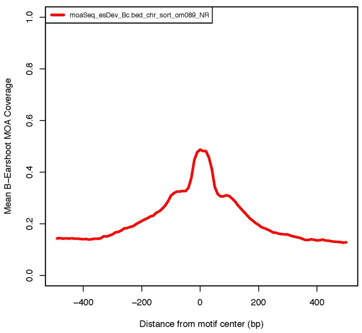	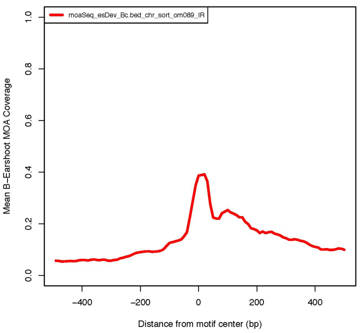

Motif Name	Total Number	In Repeats
om090	2,194	8%
Consensus	ggGAGGGGAGGGArr	
LOGO	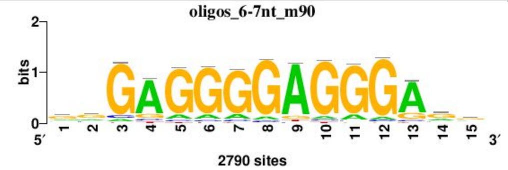	
LOGO RC	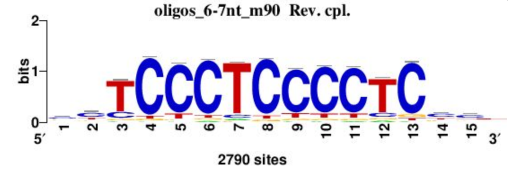	

	Frequency Distr. at TSS (FGS, B73v3)	Average Local Base Frequency (FGS, B73v3)
Median Position Relative to TSS =	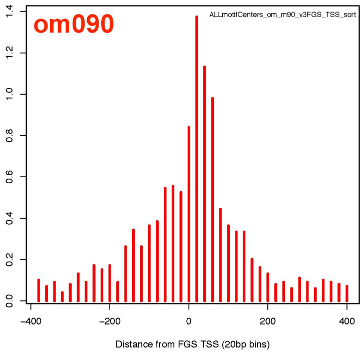	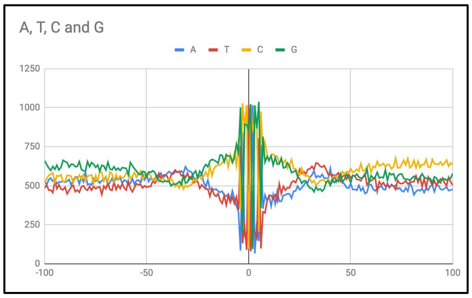
+1		

Average MOA Coverage Around Motif (RPM)

All	Not in Repeats (NR)	In Repeats (IR)
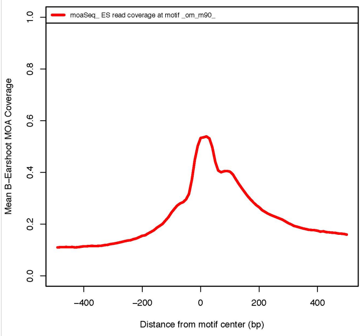	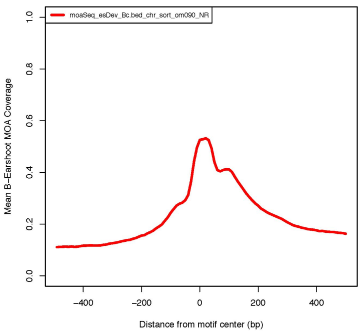	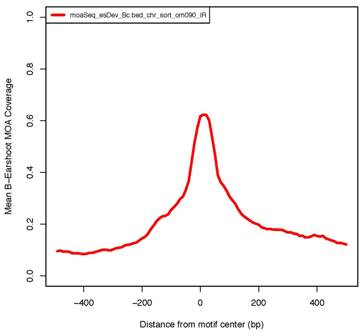

Motif Name	Total Number	In Repeats
om091	1,138	26%
Consensus	sbACCCGAys	
LOGO	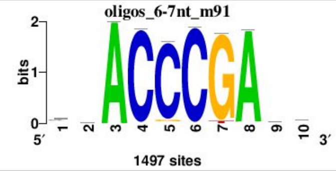	
LOGO RC	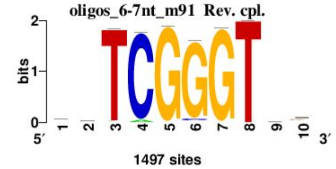	

	Frequency Distr. at TSS (FGS, B73v3)	Average Local Base Frequency (FGS, B73v3)
Median Position Relative to TSS =	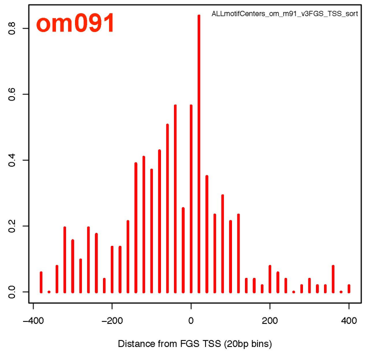	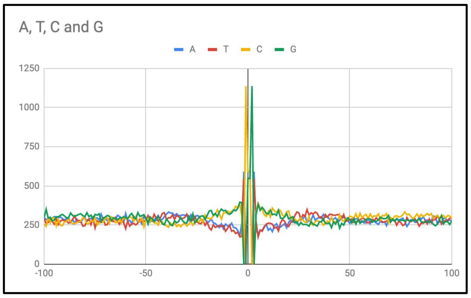
-56		

Average MOA Coverage Around Motif (RPM)

All	Not in Repeats (NR)	In Repeats (IR)
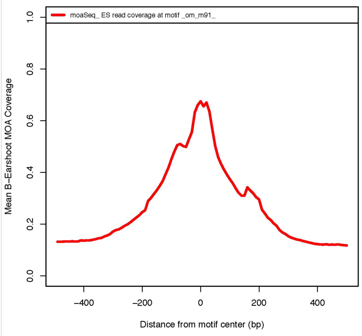	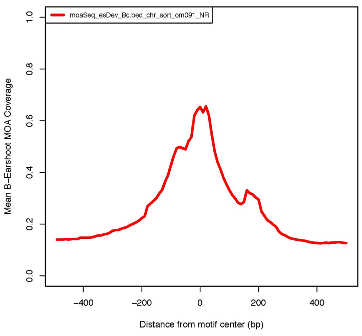	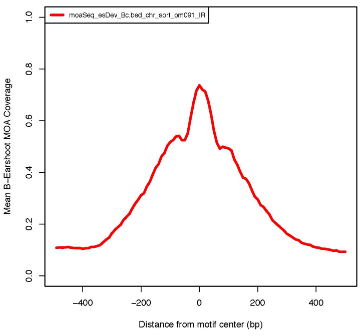

Motif Name	Total Number	In Repeats
om092	408	19%
Consensus	ssTCAGGTCAss	
LOGO	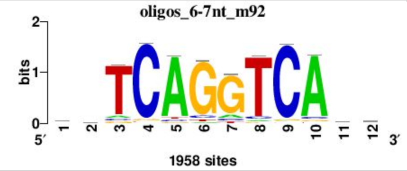	
LOGO RC	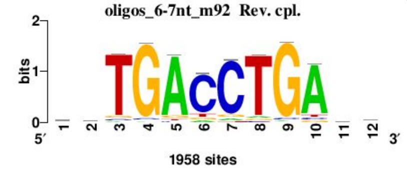	

	Frequency Distr. at TSS (FGS, B73v3)	Average Local Base Frequency (FGS, B73v3)
Median Position Relative to TSS =	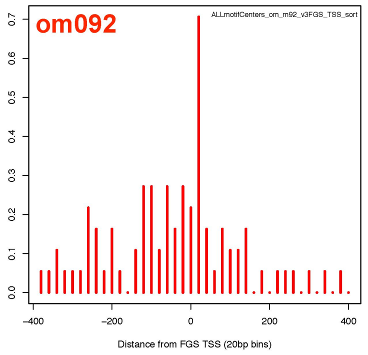	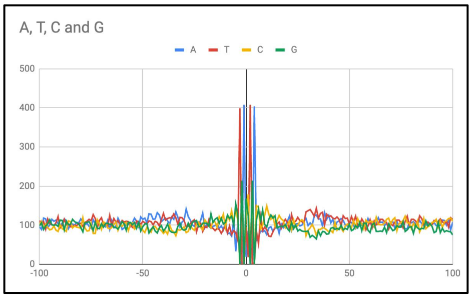
-105		

Average MOA Coverage Around Motif (RPM)

All	Not in Repeats (NR)	In Repeats (IR)
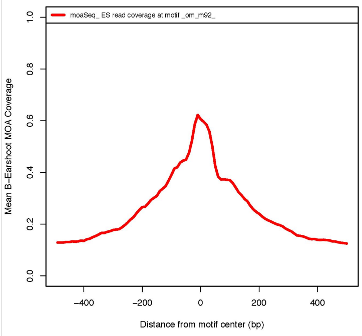	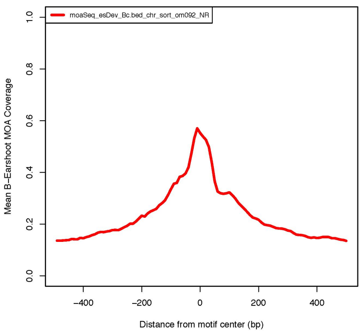	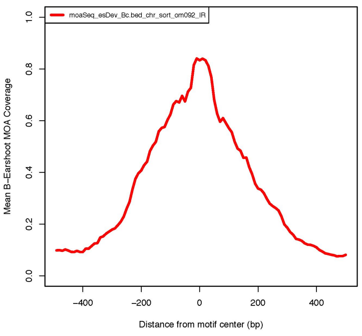

Motif Name	Total Number	In Repeats
om093	467	6%
Consensus	ssATGCCATGss	
LOGO	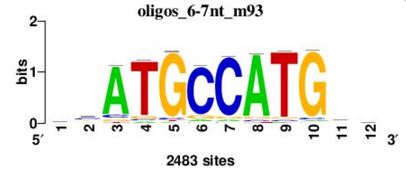	
LOGO RC	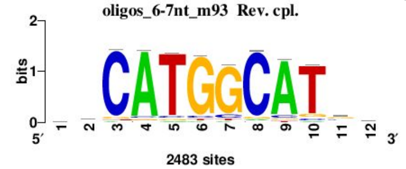	

	Frequency Distr. at TSS (FGS, B73v3)	Average Local Base Frequency (FGS, B73v3)
Median Position Relative to TSS =	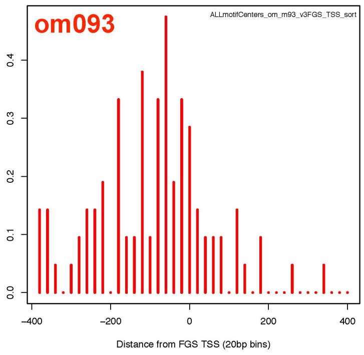	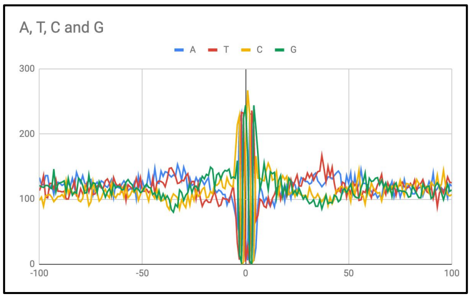
-177		

Average MOA Coverage Around Motif (RPM)

All	Not in Repeats (NR)	In Repeats (IR)
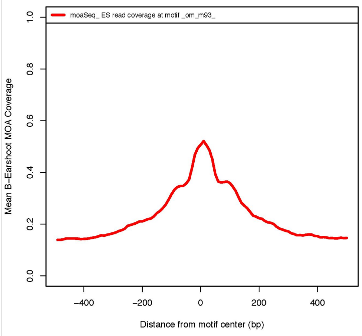	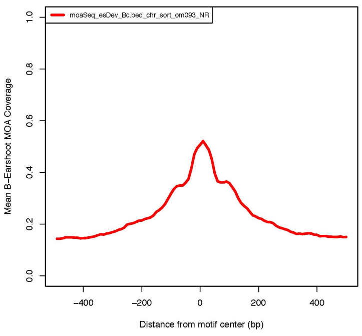	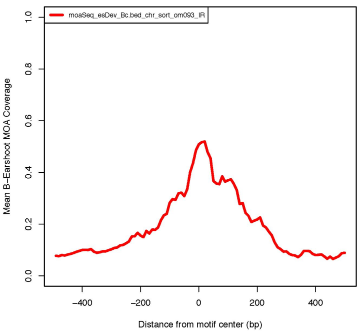

Motif Name	Total Number	In Repeats
om094	232	23%
Consensus	byCGATTCGrb	

LOGO

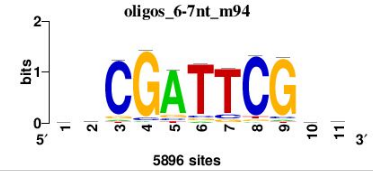

LOGO RC

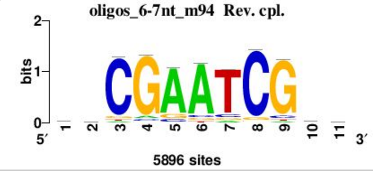

	Frequency Distr. at TSS (FGS, B73v3)	Average Local Base Frequency (FGS, B73v3)
Median Position Relative to TSS = -20	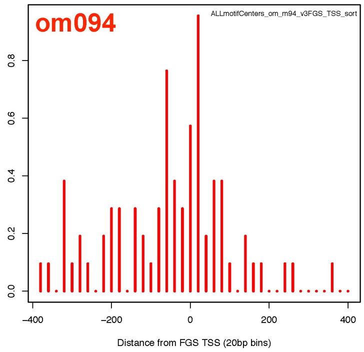	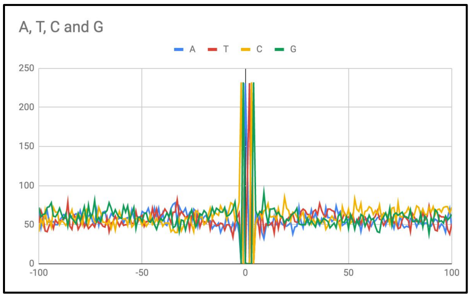

Average MOA Coverage Around Motif (RPM)

All	Not in Repeats (NR)	In Repeats (IR)
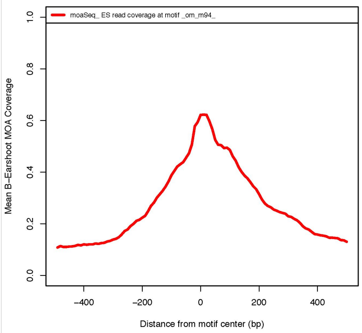	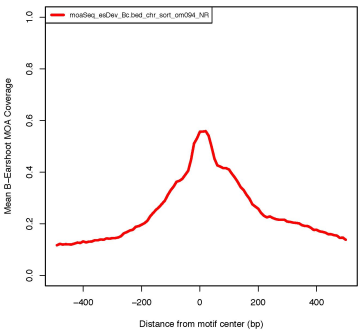	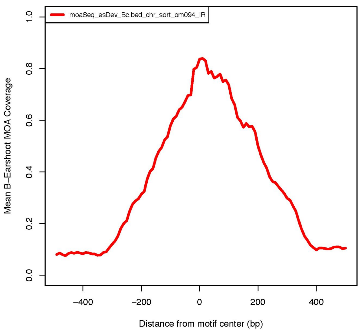

Motif Name	Total Number	In Repeats
om095	1,624	24%
Consensus	stACCAGAbv	
LOGO	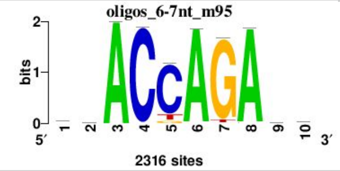	
LOGO RC	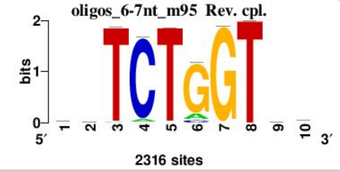	

	Frequency Distr. at TSS (FGS, B73v3)	Average Local Base Frequency (FGS, B73v3)
Median Position Relative to TSS =	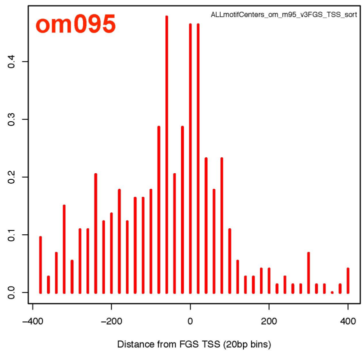	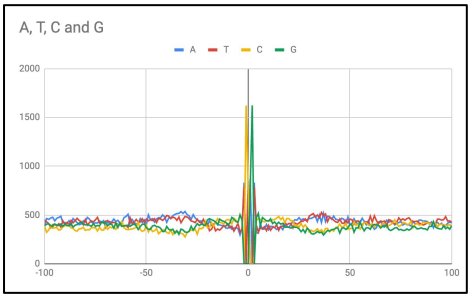
-59		

Average MOA Coverage Around Motif (RPM)

All	Not in Repeats (NR)	In Repeats (IR)
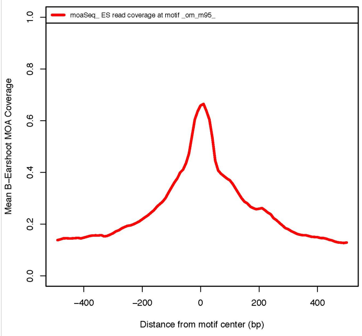	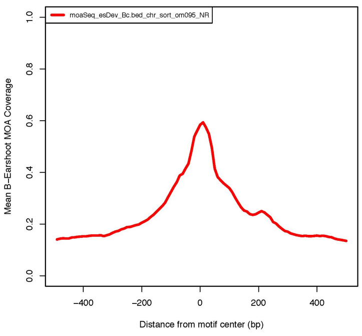	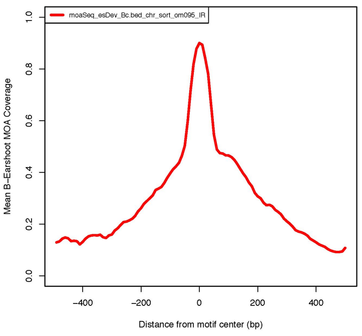

Motif Name	Total Number	In Repeats
om096	759	15%
Consensus	stACGCTAss	
LOGO	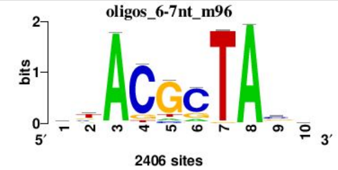	
LOGO RC	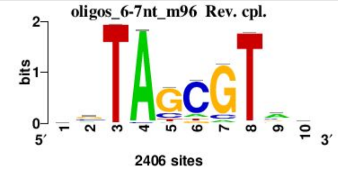	

	Frequency Distr. at TSS (FGS, B73v3)	Average Local Base Frequency (FGS, B73v3)
Median Position Relative to TSS =	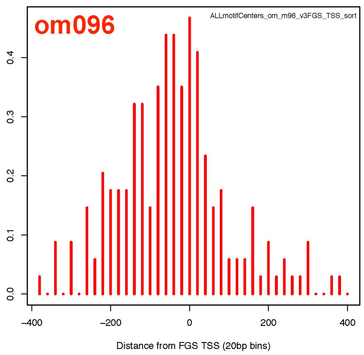	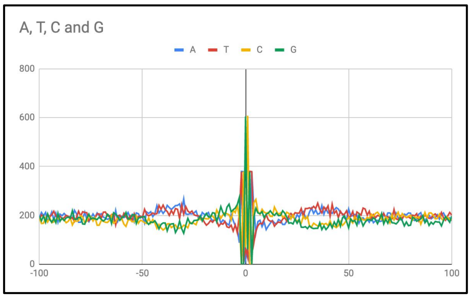
-71		

Average MOA Coverage Around Motif (RPM)

All	Not in Repeats (NR)	In Repeats (IR)
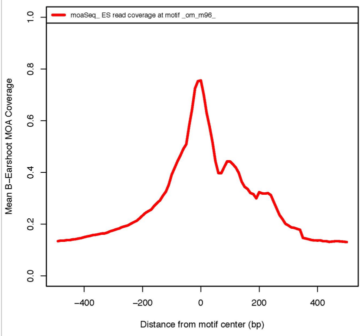	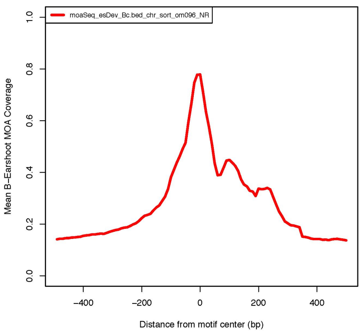	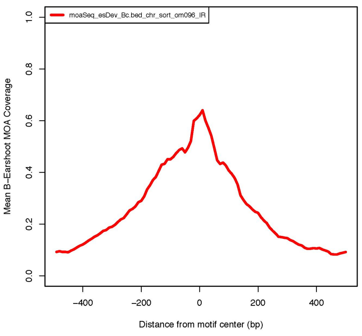

Motif Name	Total Number	In Repeats
**om097**	**3,554**	**15%**
**Consensus**	scAGCCCAasm	
**LOGO**	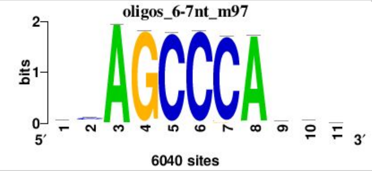	
**LOGO RC**	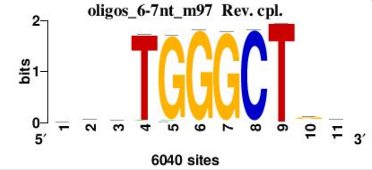	

	Frequency Distr. at TSS (FGS, B73v3)	Average Local Base Frequency (FGS, B73v3)
**Median Position Relative to TSS =**	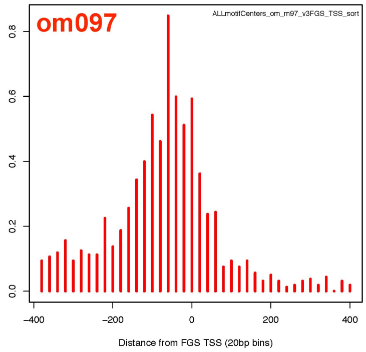	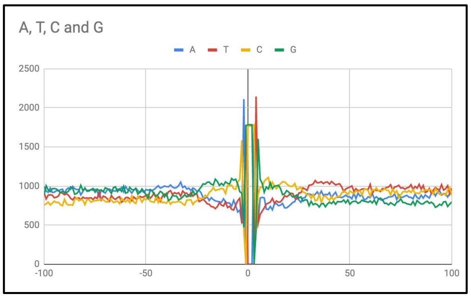
**-88**		

Average MOA Coverage Around Motif (RPM)

All	Not in Repeats (NR)	In Repeats (IR)
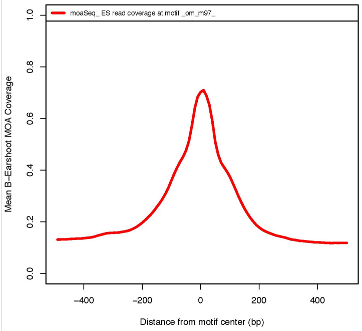	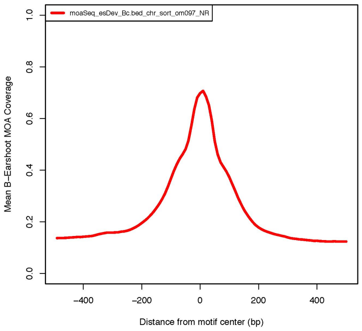	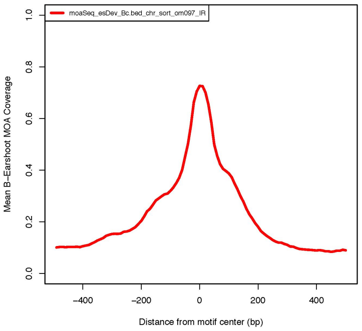

Motif Name	Total Number	In Repeats
om098	1,800	10%
Consensus	bmCGTGTCss	
LOGO	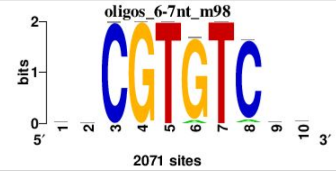	
LOGO RC	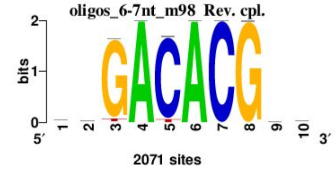	

	Frequency Distr. at TSS (FGS, B73v3)	Average Local Base Frequency (FGS, B73v3)
Median Position Relative to TSS =	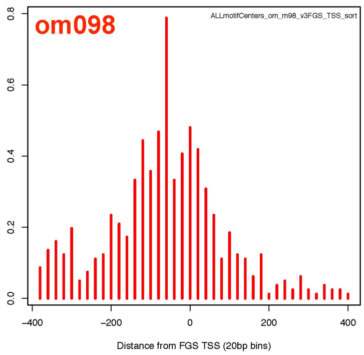	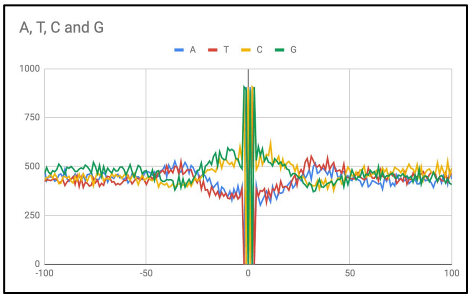
-100		

Average MOA Coverage Around Motif (RPM)

All	Not in Repeats (NR)	In Repeats (IR)
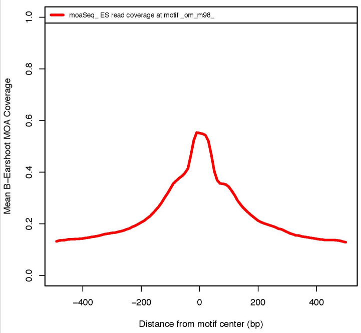	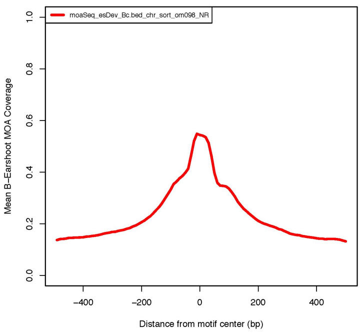	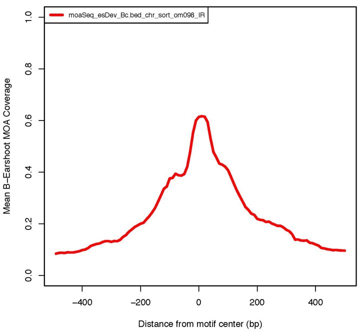

Motif Name	Total Number	In Repeats
om099	1,472	14%
Consensus	ymCCTACCks	
LOGO	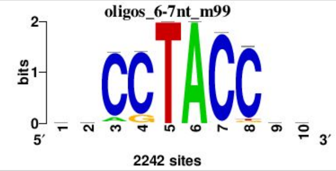	
LOGO RC	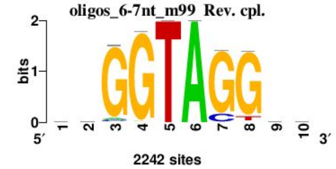	

	Frequency Distr. at TSS (FGS, B73v3)	Average Local Base Frequency (FGS, B73v3)
Median Position Relative to TSS =	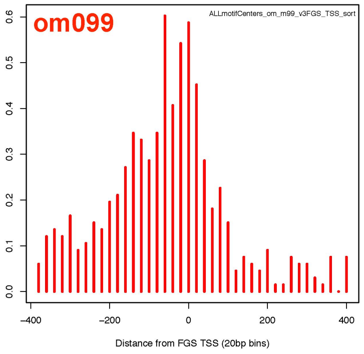	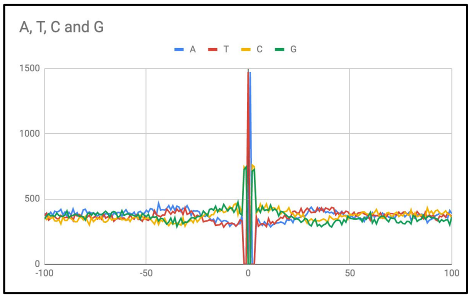
-72		

Average MOA Coverage Around Motif (RPM)

All	Not in Repeats (NR)	In Repeats (IR)
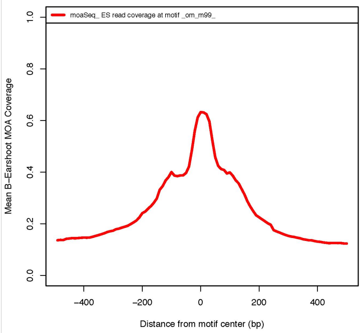	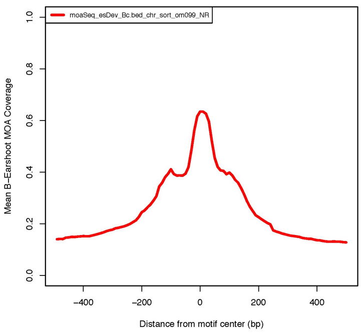	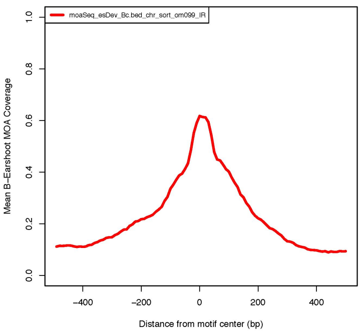

Motif Name	Total Number	In Repeats
om100	966	23%
Consensus	ctACACTAsh	
LOGO	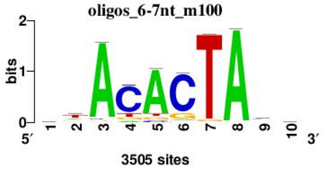	
LOGO RC	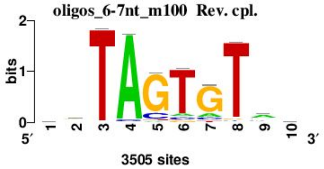	

	Frequency Distr. at TSS (FGS, B73v3)	Average Local Base Frequency (FGS, B73v3)
Median Position Relative to TSS =	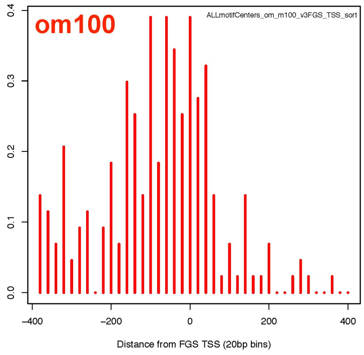	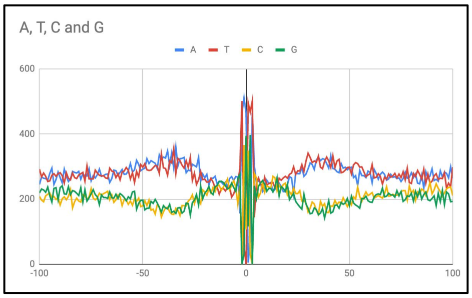
-103		

Average MOA Coverage Around Motif (RPM)

All	Not in Repeats (NR)	In Repeats (IR)
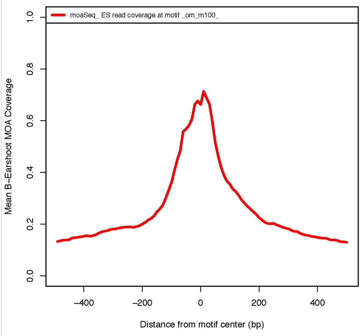	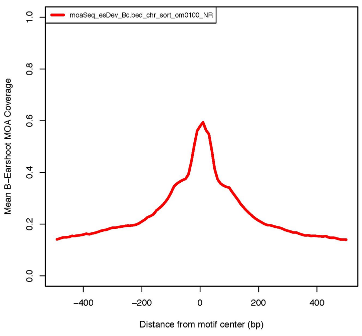	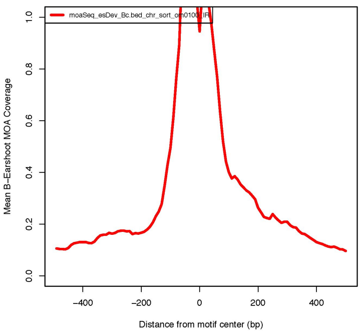

Motif Name	Total Number	In Repeats
om101	1,001	42%
Consensus	wtAAGTAAww	
LOGO	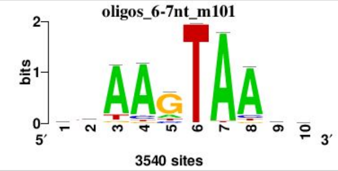	
LOGO RC	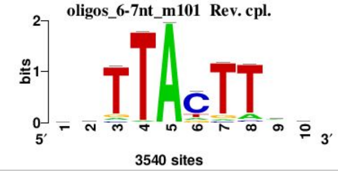	

	Frequency Distr. at TSS (FGS, B73v3)	Average Local Base Frequency (FGS, B73v3)
Median Position Relative to TSS =	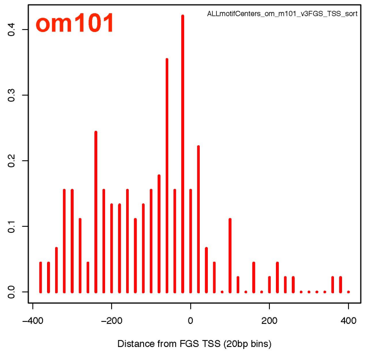	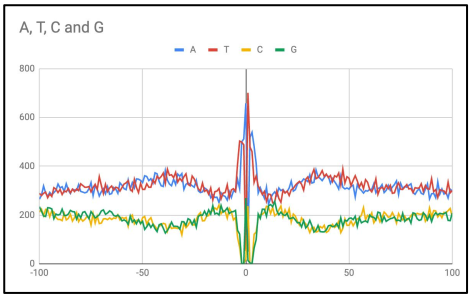
-71		

Average MOA Coverage Around Motif (RPM)		
All	Not in Repeats (NR)	In Repeats (IR)
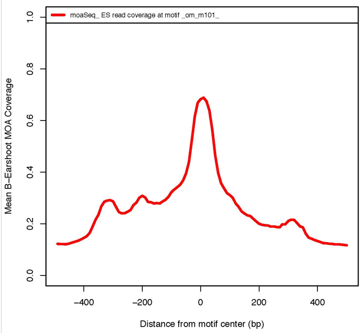	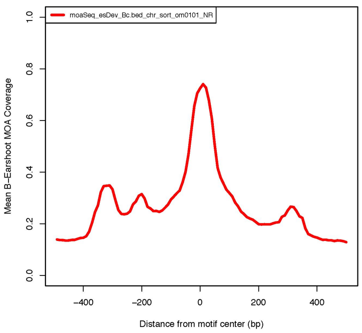	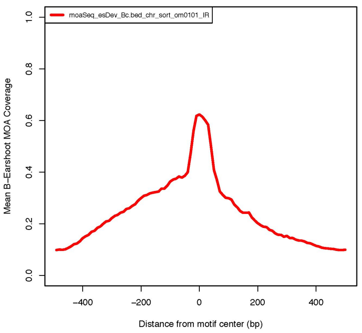

Motif Name	Total Number	In Repeats
om102	1,640	11%
Consensus	scGGCGCCgs	
LOGO	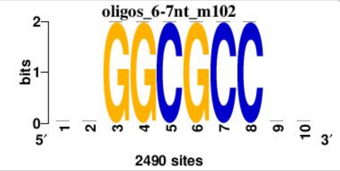	
LOGO RC	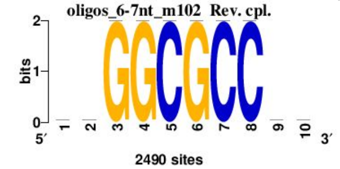	

	Frequency Distr. at TSS (FGS, B73v3)	Average Local Base Frequency (FGS, B73v3)
Median Position Relative to TSS =	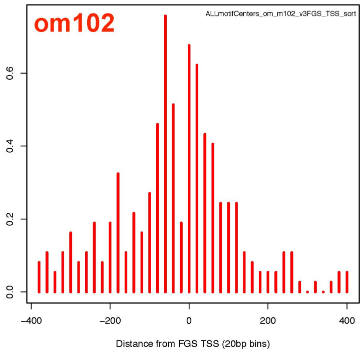	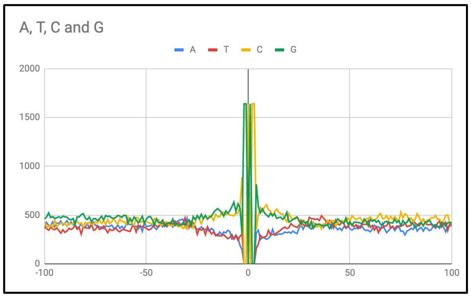
-59		

Average MOA Coverage Around Motif (RPM)

All	Not in Repeats (NR)	In Repeats (IR)
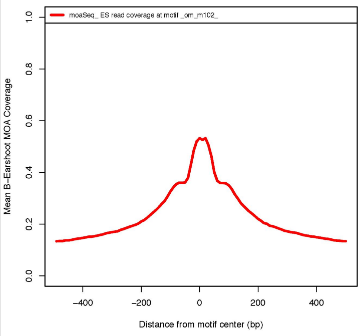	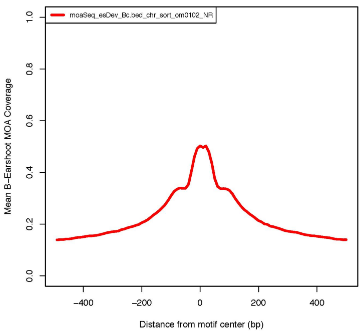	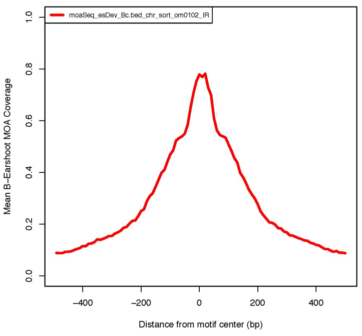

Motif Name	Total Number	In Repeats
om103	329	8%
Consensus	syGCATTGCrb	
LOGO	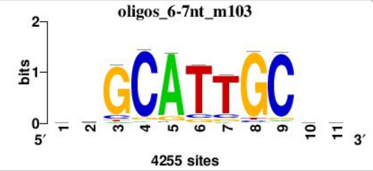	
LOGO RC	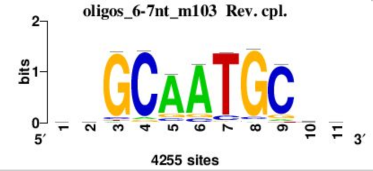	

	Frequency Distr. at TSS (FGS, B73v3)	Average Local Base Frequency (FGS, B73v3)
Median Position Relative to TSS =	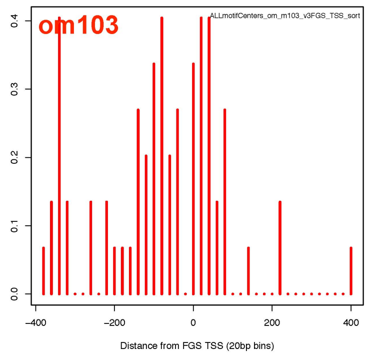	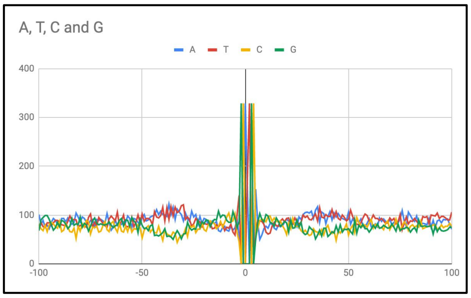
-65		

Average MOA Coverage Around Motif (RPM)

All	Not in Repeats (NR)	In Repeats (IR)
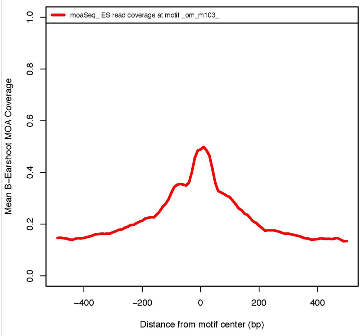	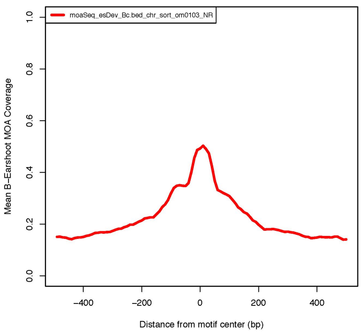	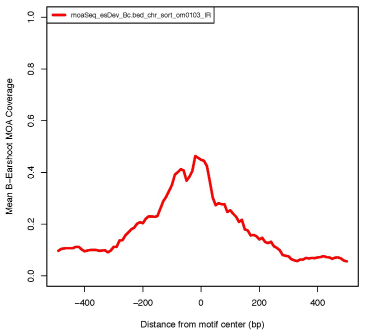

Motif Name	Total Number	In Repeats
om104	4,425	21%
Consensus	rrGAAAArAAAAcar	

LOGO

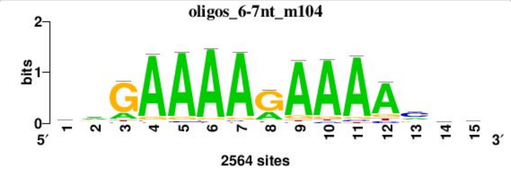

LOGO RC

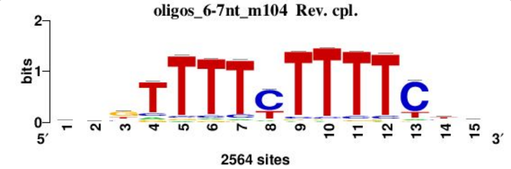

	Frequency Distr. at TSS (FGS, B73v3)	Average Local Base Frequency (FGS, B73v3)
Median Position Relative to TSS = -74	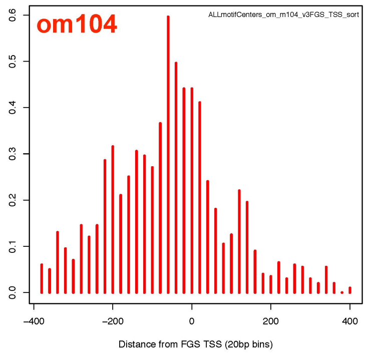	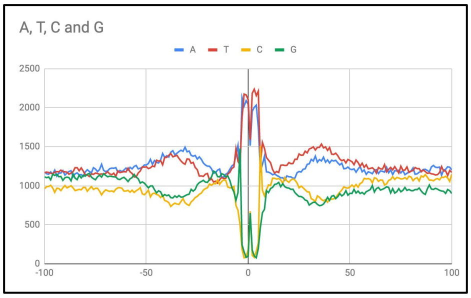

Average MOA Coverage Around Motif (RPM)

All	Not in Repeats (NR)	In Repeats (IR)
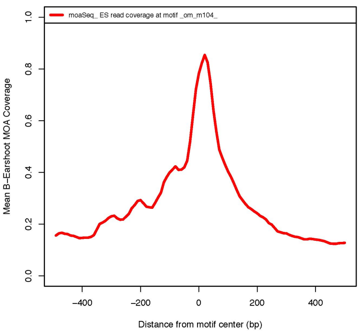	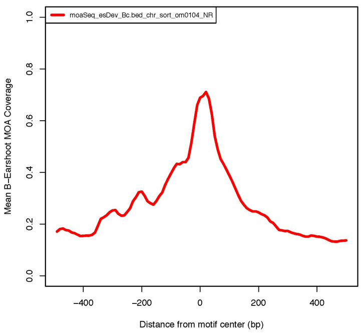	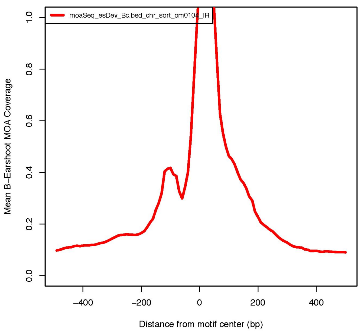

Motif Name	Total Number	In Repeats
om105	1,941	12%
Consensus	gsCCTGGCcs	
LOGO	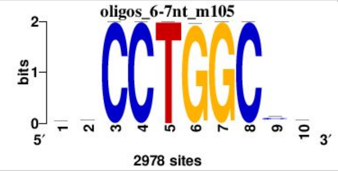	
LOGO RC	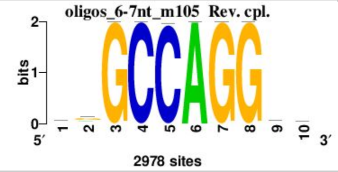	

	Frequency Distr. at TSS (FGS, B73v3)	Average Local Base Frequency (FGS, B73v3)
Median Position Relative to TSS =	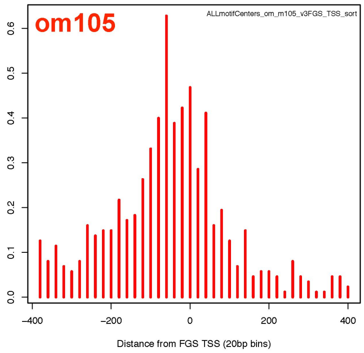	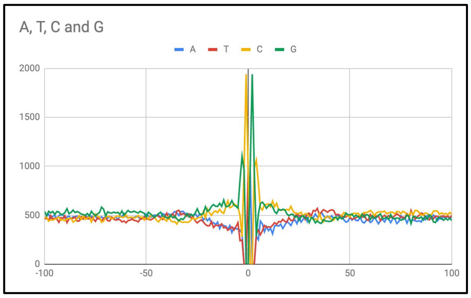
-87		

Average MOA Coverage Around Motif (RPM)

All	Not in Repeats (NR)	In Repeats (IR)
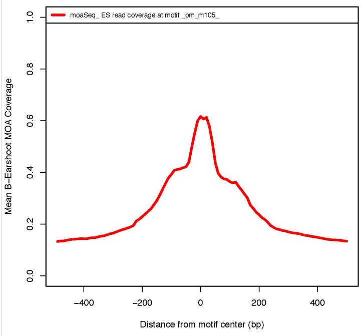	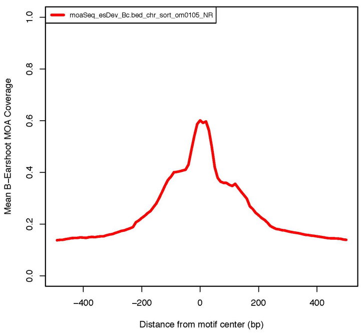	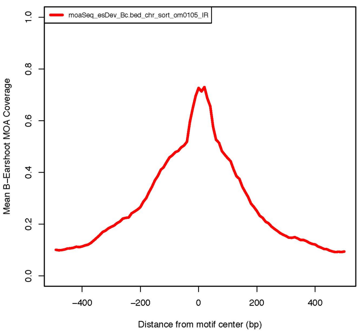

Motif Name	Total Number	In Repeats
**om106**	**1,901**	**41%**
**Consensus**	**wwAATgAAwa**	
**LOGO**	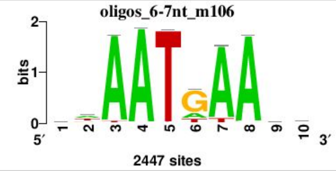	
**LOGO RC**	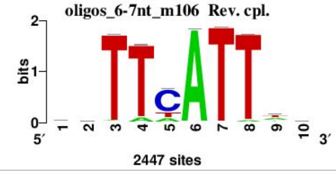	

	Frequency Distr. at TSS (FGS, B73v3)	Average Local Base Frequency (FGS, B73v3)
Median Position Relative to TSS =	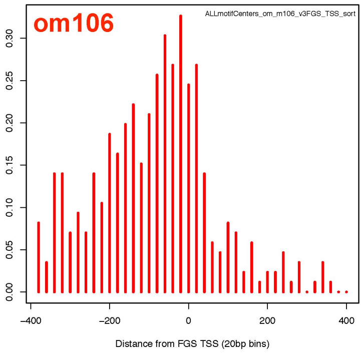	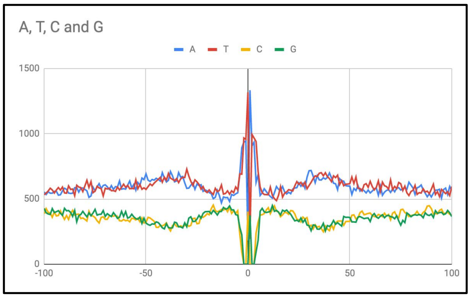
**-66**		

Average MOA Coverage Around Motif (RPM)

All	Not in Repeats (NR)	In Repeats (IR)
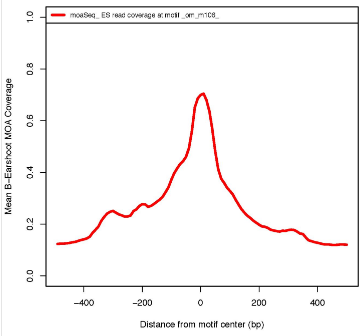	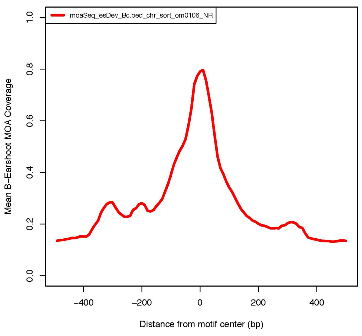	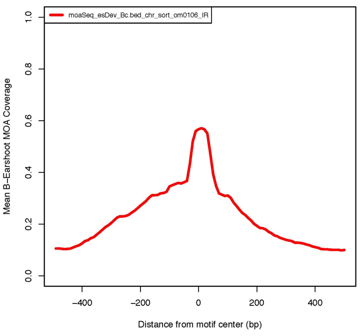

Motif Name	Total Number	In Repeats
om107	239	13%
Consensus	cmGTAGCATss	
LOGO	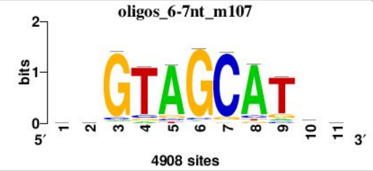	
LOGO RC	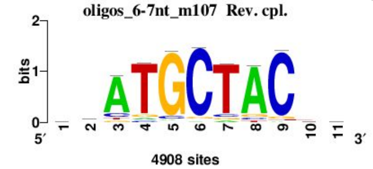	

	Frequency Distr. at TSS (FGS, B73v3)	Average Local Base Frequency (FGS, B73v3)
Median Position Relative to TSS =	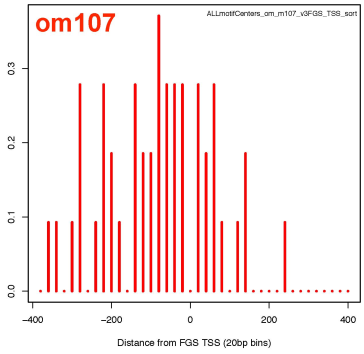	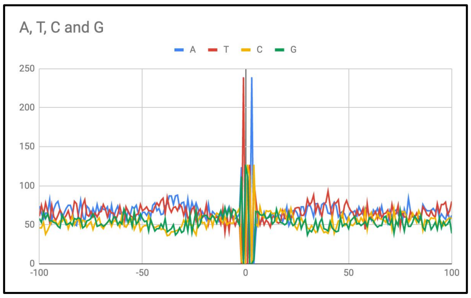
-105		

Average MOA Coverage Around Motif (RPM)

All	Not in Repeats (NR)	In Repeats (IR)
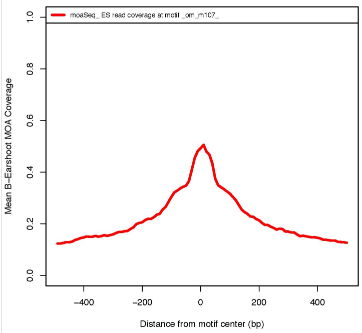	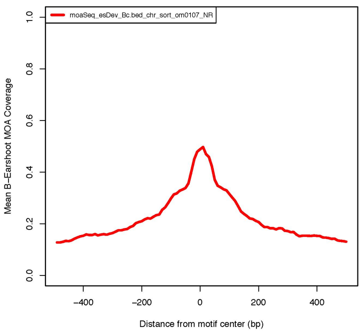	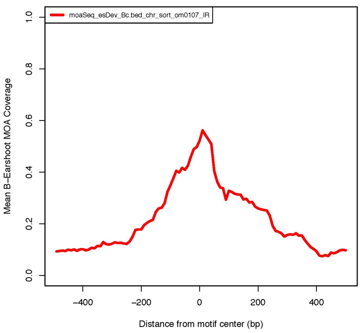

Motif Name	Total Number	In Repeats
om108	805	44%
Consensus	wwTAAGATAaw	
LOGO	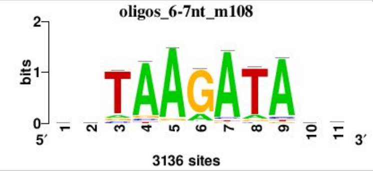	
LOGO RC	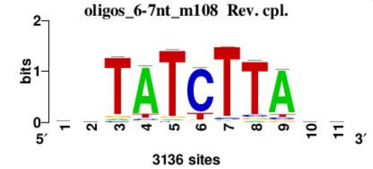	

	Frequency Distr. at TSS (FGS, B73v3)	Average Local Base Frequency (FGS, B73v3)
Median Position Relative to TSS =	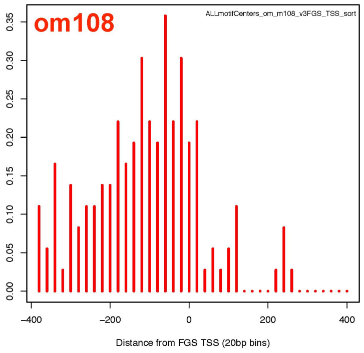	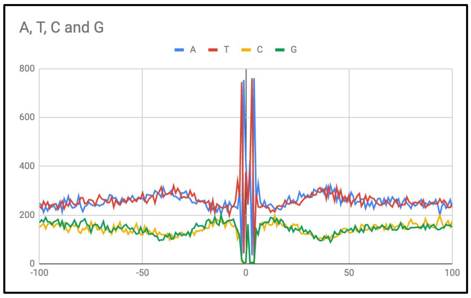
-103		

Average MOA Coverage Around Motif (RPM)		
All	Not in Repeats (NR)	In Repeats (IR)
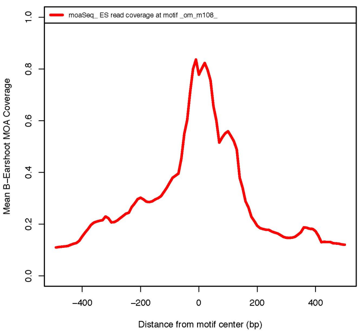	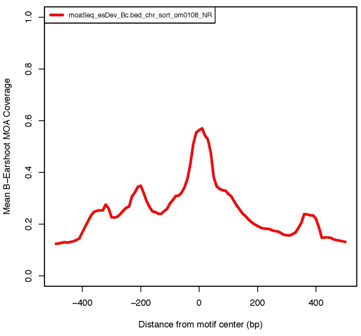	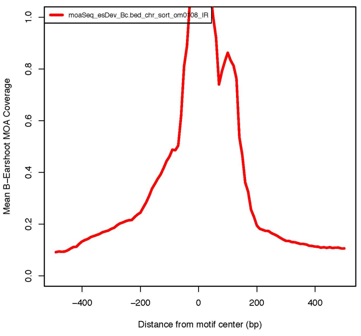

Motif Name	Total Number	In Repeats
om109	1,377	53%
Consensus	wkATCTAaym	
LOGO	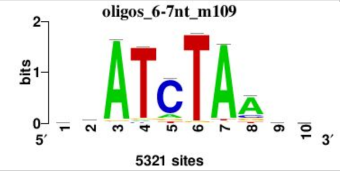	
LOGO RC	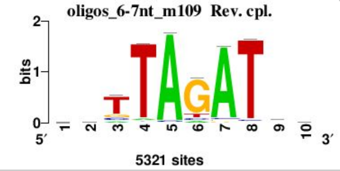	

	Frequency Distr. at TSS (FGS, B73v3)	Average Local Base Frequency (FGS, B73v3)
Median Position Relative to TSS =	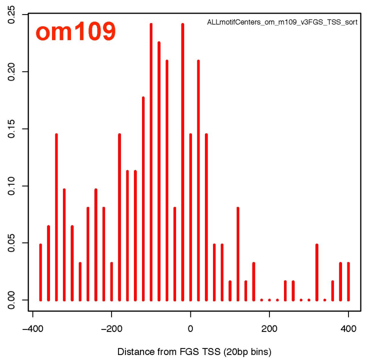	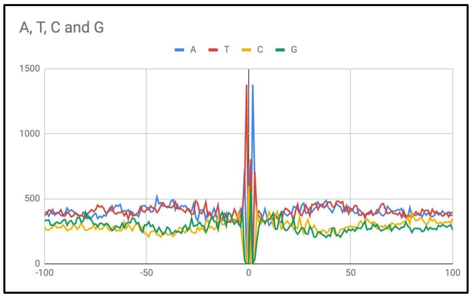
-70		

Average MOA Coverage Around Motif (RPM)		
All	Not in Repeats (NR)	In Repeats (IR)
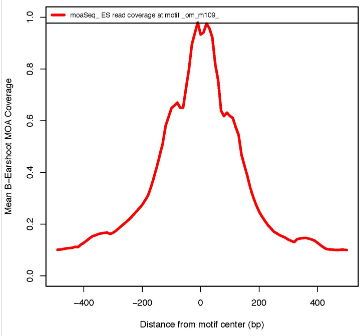	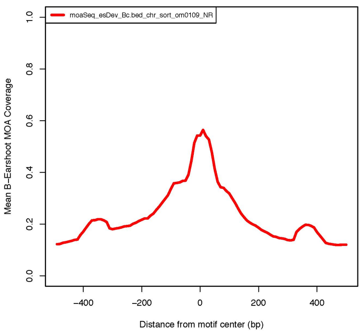	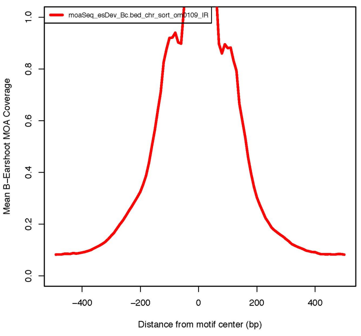

Motif Name	Total Number	In Repeats
om110	1,989	10%

Consensus	ybCCTGTCcb
LOGO	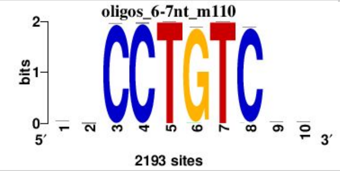
LOGO RC	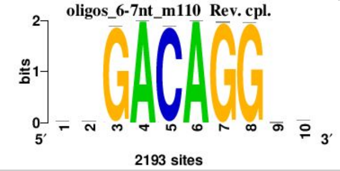

	Frequency Distr. at TSS (FGS, B73v3)	Average Local Base Frequency (FGS, B73v3)
Median Position Relative to TSS =	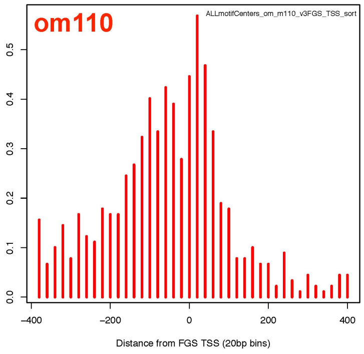	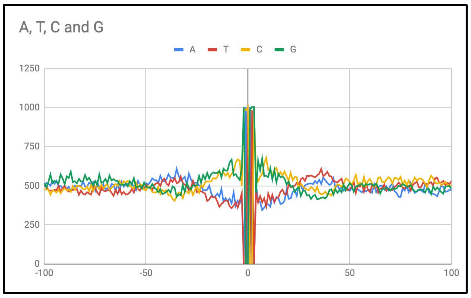
-66		

Average MOA Coverage Around Motif (RPM)

All	Not in Repeats (NR)	In Repeats (IR)
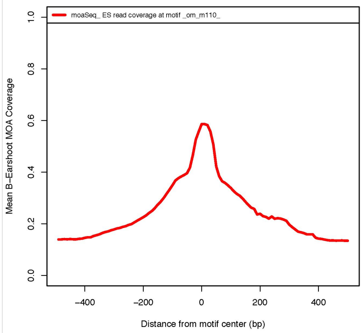	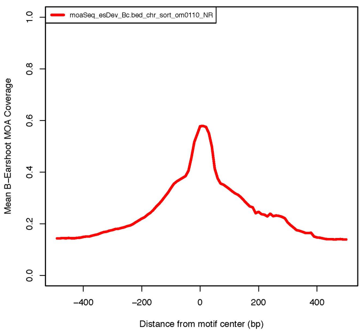	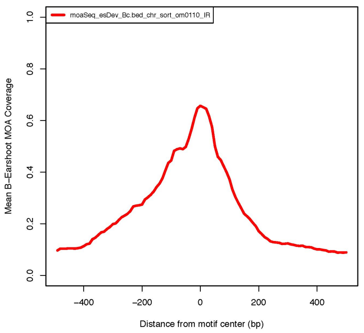

Motif Name	Total Number	In Repeats
om111	1,477	8%
Consensus	gsCAAGGCms	
LOGO	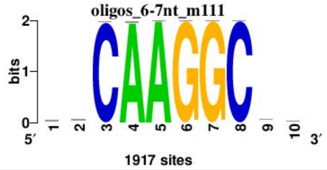	
LOGO RC	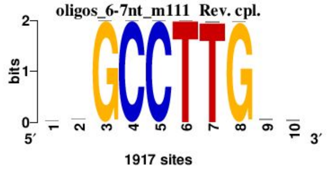	

	Frequency Distr. at TSS (FGS, B73v3)	Average Local Base Frequency (FGS, B73v3)
Median Position Relative to TSS =	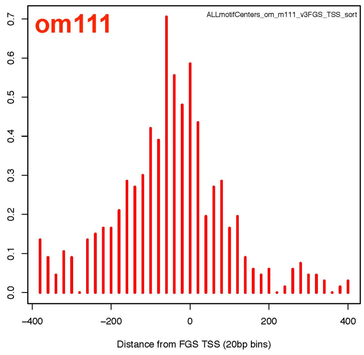	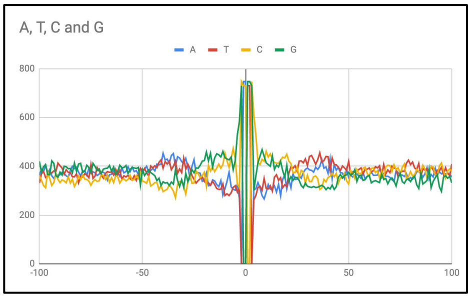
-60		

Average MOA Coverage Around Motif (RPM)

All	Not in Repeats (NR)	In Repeats (IR)
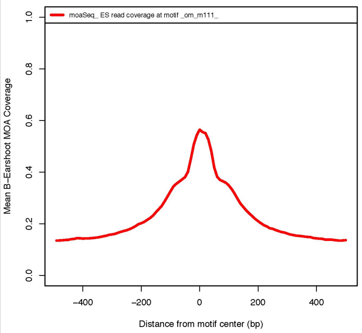	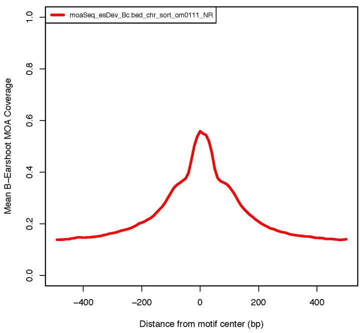	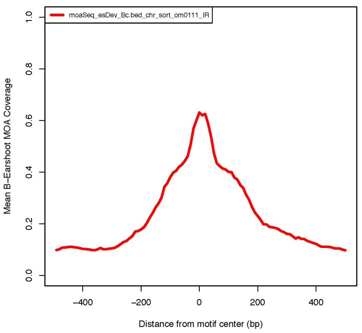

Motif Name	Total Number	In Repeats
om112	1,477	8%
Consensus	gsCAAGGCms	
LOGO	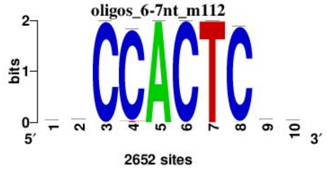	
LOGO RC	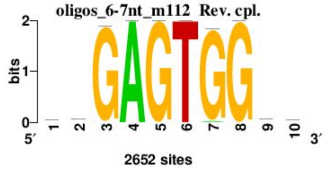	

	Frequency Distr. at TSS (FGS, B73v3)	Average Local Base Frequency (FGS, B73v3)
Median Position Relative to TSS =	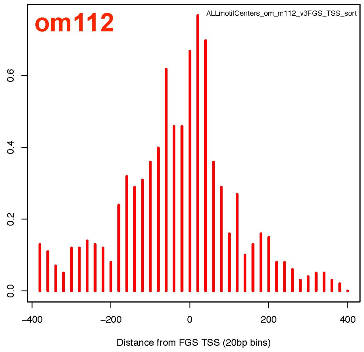	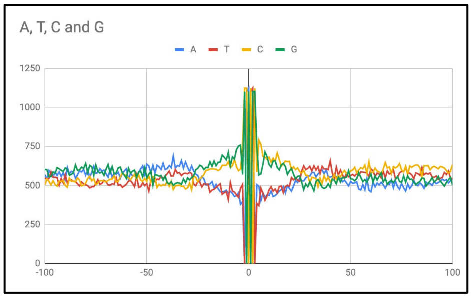
-60		

Average MOA Coverage Around Motif (RPM)

All	Not in Repeats (NR)	In Repeats (IR)
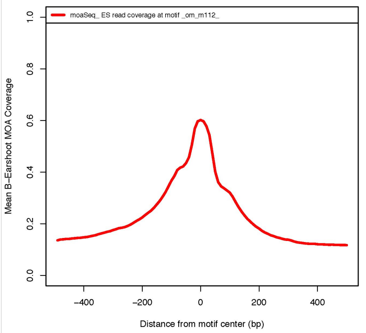	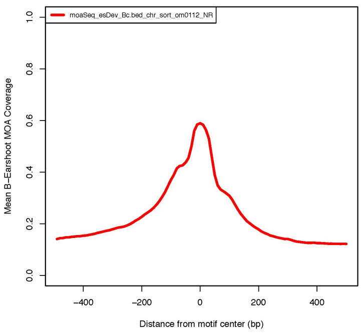	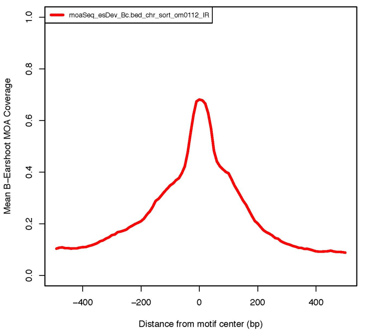

Motif Name	Total Number	In Repeats
om113	1,239	42%
Consensus	wwAAcATAya	
LOGO	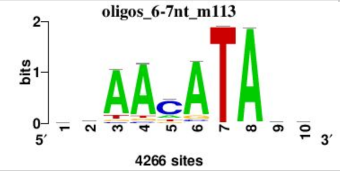	
LOGO RC	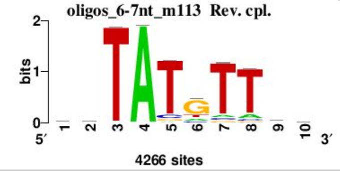	

	Frequency Distr. at TSS (FGS, B73v3)	Average Local Base Frequency (FGS, B73v3)
Median Position Relative to TSS =	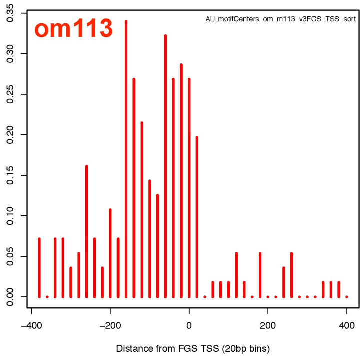	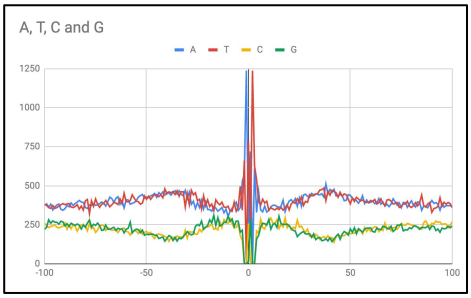
-112		

Average MOA Coverage Around Motif (RPM)

All	Not in Repeats (NR)	In Repeats (IR)
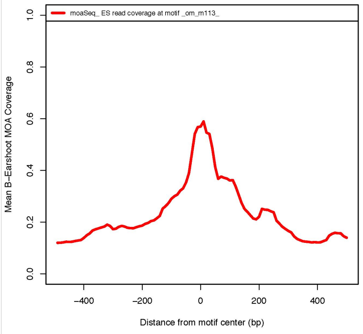	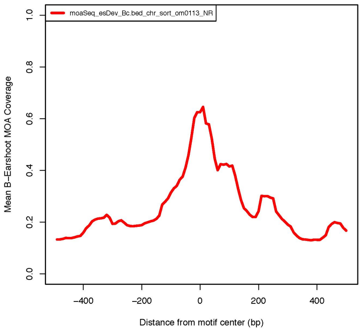	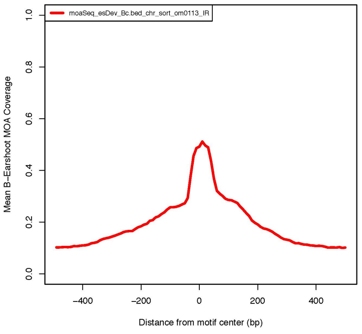

Motif Name	Total Number	In Repeats
om114	1,398	47%
Consensus	ctAGATCArm	
LOGO	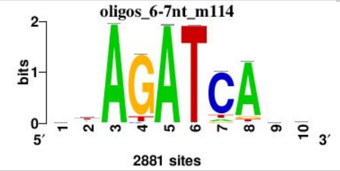	
LOGO RC	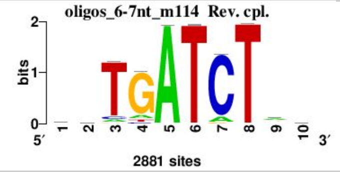	

	Frequency Distr. at TSS (FGS, B73v3)	Average Local Base Frequency (FGS, B73v3)
Median Position Relative to TSS =	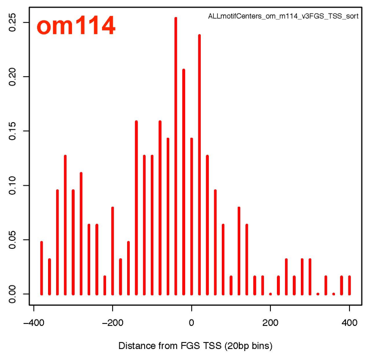	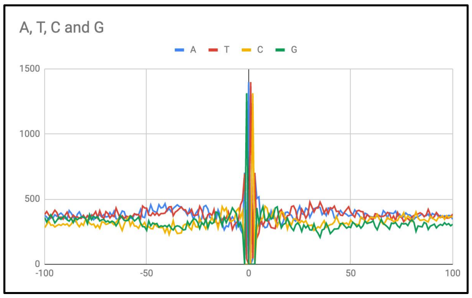
-54		

Average MOA Coverage Around Motif (RPM)

All	Not in Repeats (NR)	In Repeats (IR)
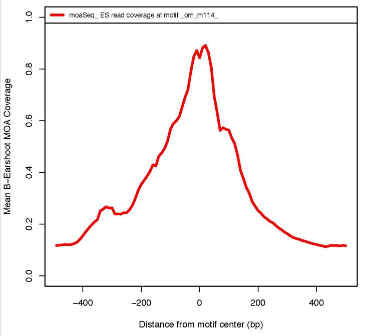	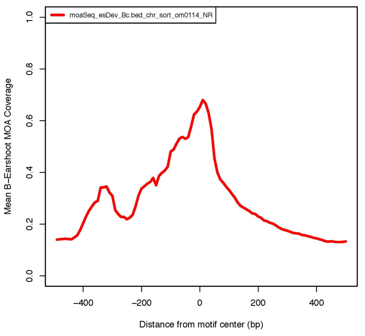	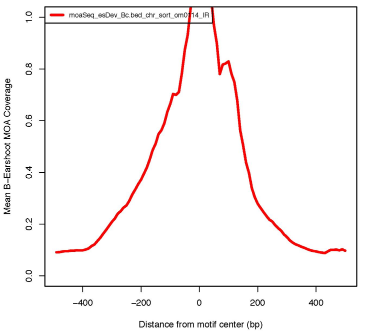

Motif Name	Total Number	In Repeats
om115	1,060	45%
Consensus	wtATTCTAba	
LOGO	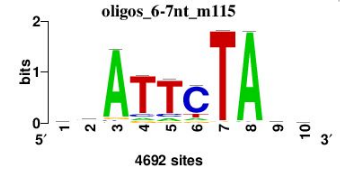	
LOGO RC	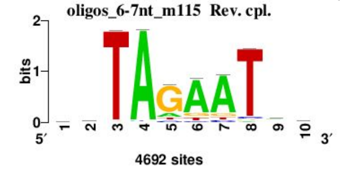	

	Frequency Distr. at TSS (FGS, B73v3)	Average Local Base Frequency (FGS, B73v3)
Median Position Relative to TSS =	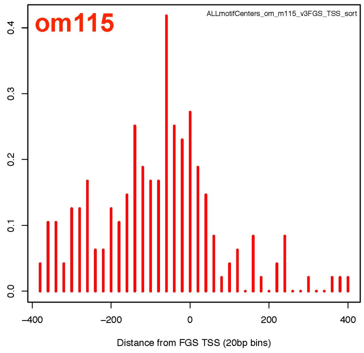	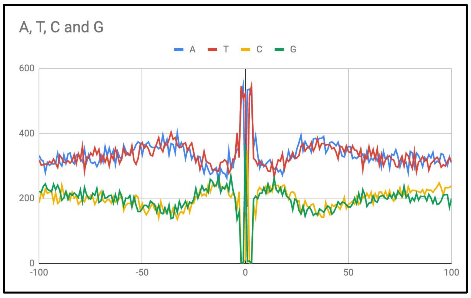
-69		

Average MOA Coverage Around Motif (RPM)

All	Not in Repeats (NR)	In Repeats (IR)
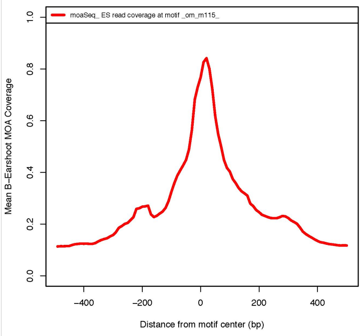	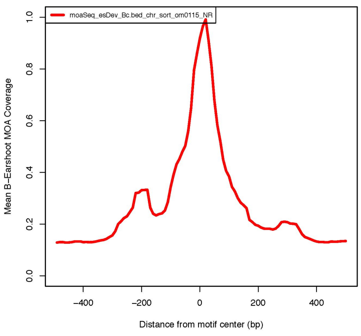	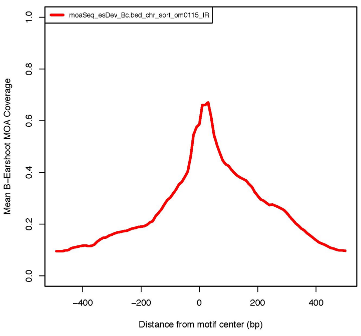

Motif Name	Total Number	In Repeats
om116	3,315	15%
Consensus	kgaTGGGCTgs	
LOGO	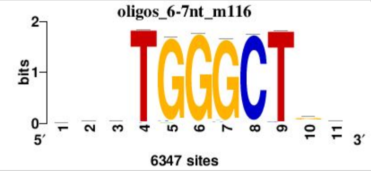	
LOGO RC	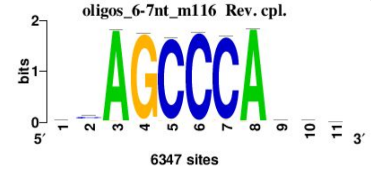	

	Frequency Distr. at TSS (FGS, B73v3)	Average Local Base Frequency (FGS, B73v3)
Median Position Relative to TSS = -87	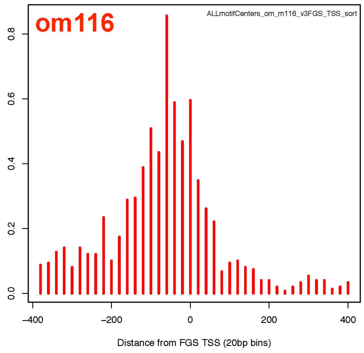	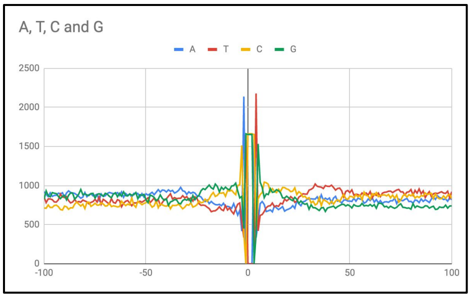

Average MOA Coverage Around Motif (RPM)

All	Not in Repeats (NR)	In Repeats (IR)
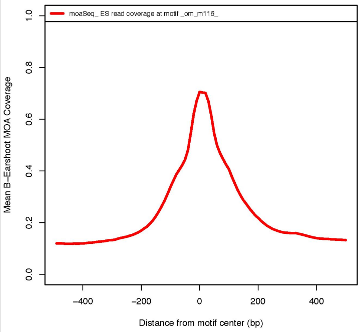	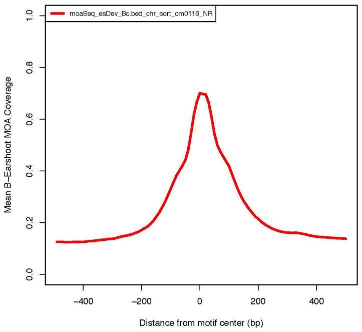	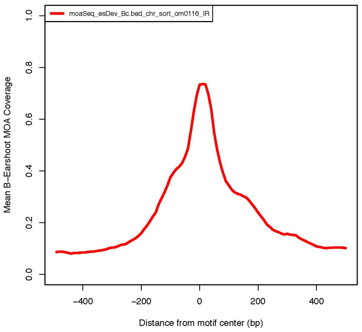

Motif Name	Total Number	In Repeats
om117	339	9%
Consensus	csGCTTCGCsb	
LOGO	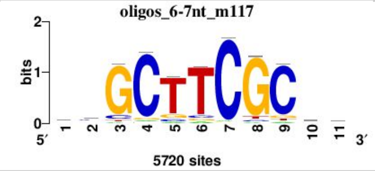	
LOGO RC	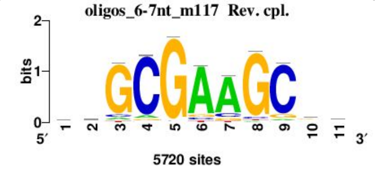	

	Frequency Distr. at TSS (FGS, B73v3)	Average Local Base Frequency (FGS, B73v3)
Median Position Relative to TSS =	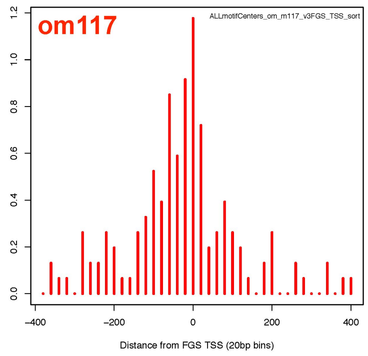	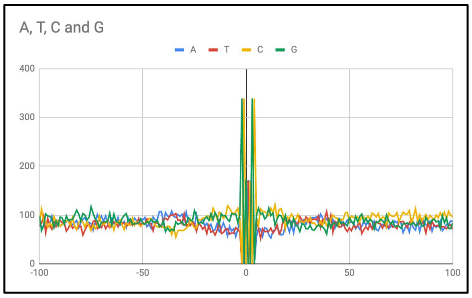
-39		

Average MOA Coverage Around Motif (RPM)

All	Not in Repeats (NR)	In Repeats (IR)
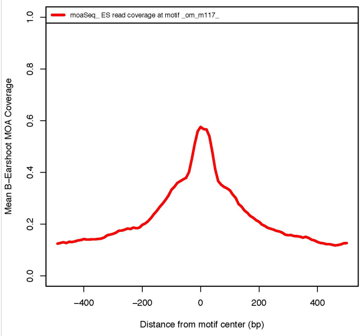	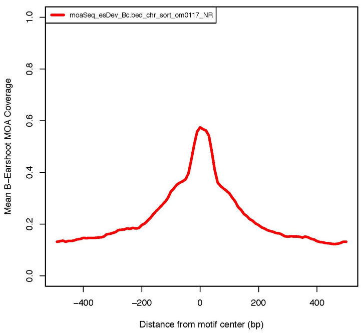	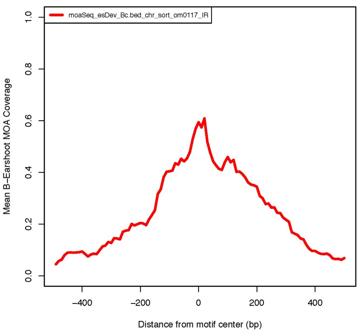

Motif Name	Total Number	In Repeats
om118	1,361	38%
Consensus	taTCTAGAta	

LOGO

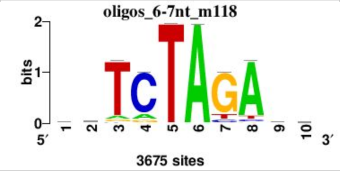

LOGO RC

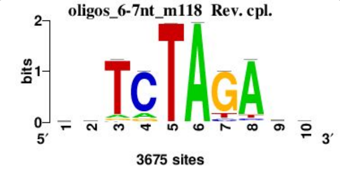

	Frequency Distr. at TSS (FGS, B73v3)	Average Local Base Frequency (FGS, B73v3)
Median Position Relative to TSS =		
-80		

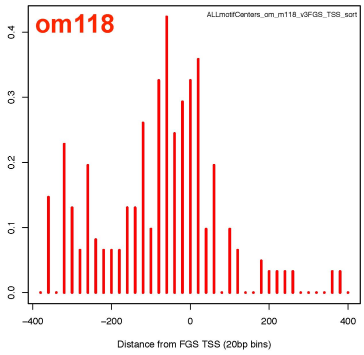

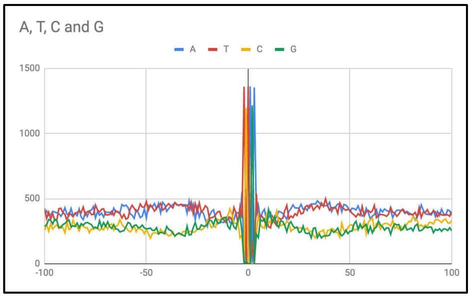

Average MOA Coverage Around Motif (RPM)

All	Not in Repeats (NR)	In Repeats (IR)

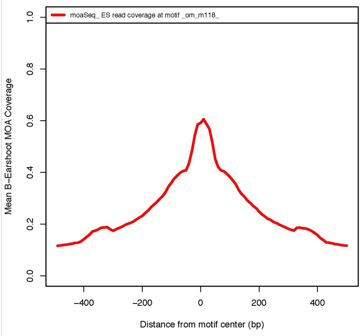

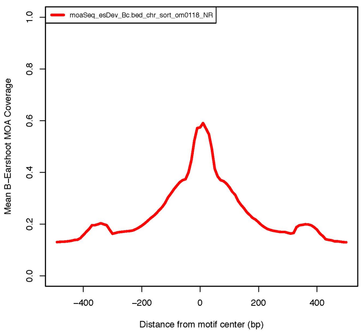

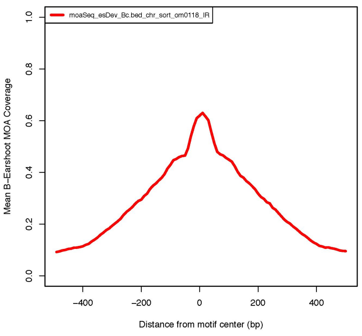

Motif Name	Total Number	In Repeats
om119	1,255	18%
Consensus	ycCAACTCcm	
LOGO	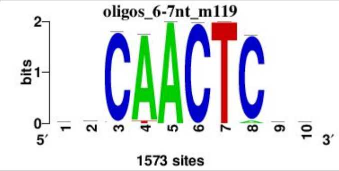	
LOGO RC	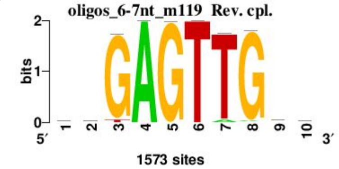	

	Frequency Distr. at TSS (FGS, B73v3)	Average Local Base Frequency (FGS, B73v3)
Median Position Relative to TSS = -81	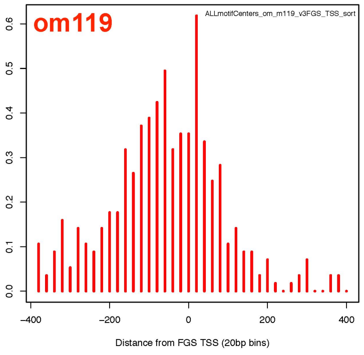	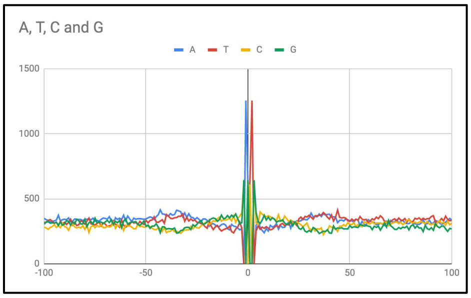

Average MOA Coverage Around Motif (RPM)

All	Not in Repeats (NR)	In Repeats (IR)
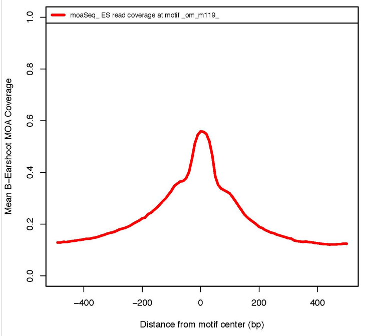	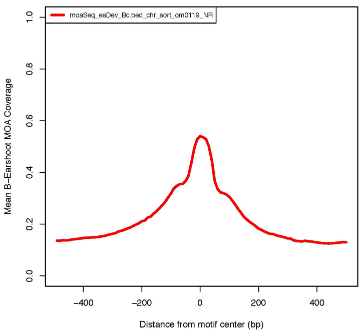	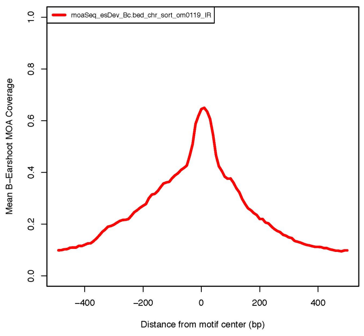

Motif Name	Total Number	In Repeats
om120	1,856	9%
Consensus	ssCCCCGCss	
LOGO	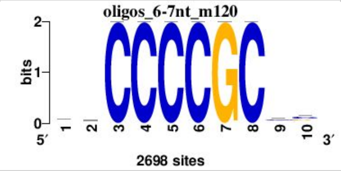	
LOGO RC	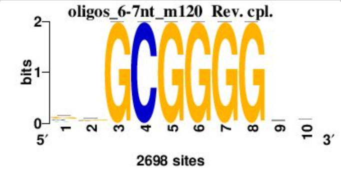	

	Frequency Distr. at TSS (FGS, B73v3)	Average Local Base Frequency (FGS, B73v3)
Median Position Relative to TSS = -67	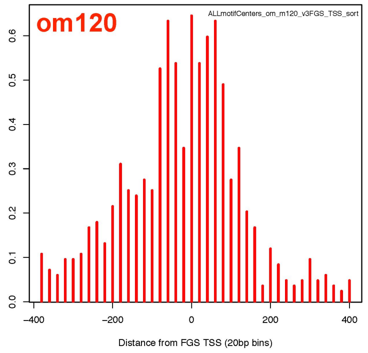	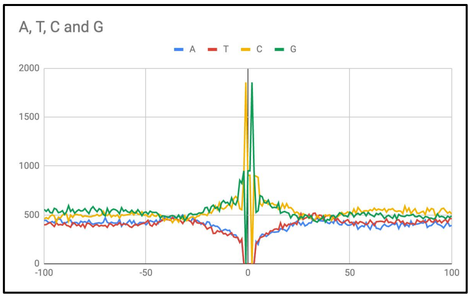

Average MOA Coverage Around Motif (RPM)

All	Not in Repeats (NR)	In Repeats (IR)
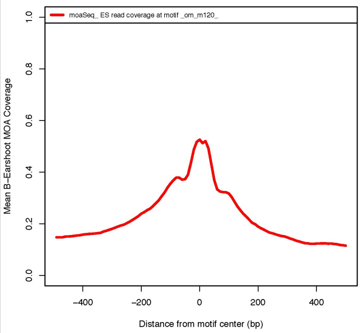	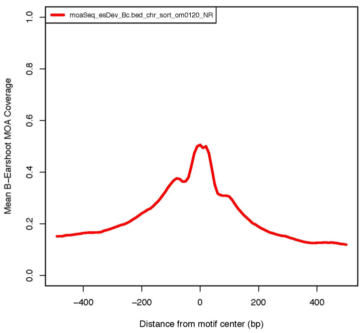	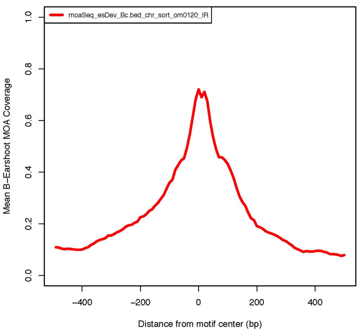

Motif Name	Total Number	In Repeats
om121	1,251	21%
Consensus	mtACAACAmm	
LOGO	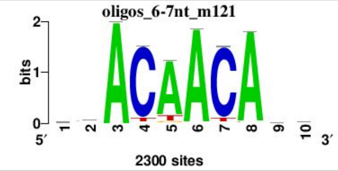	
LOGO RC	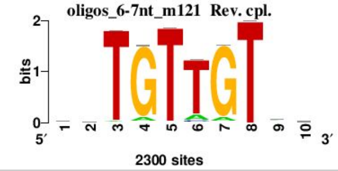	

	Frequency Distr. at TSS (FGS, B73v3)	Average Local Base Frequency (FGS, B73v3)
Median Position Relative to TSS =	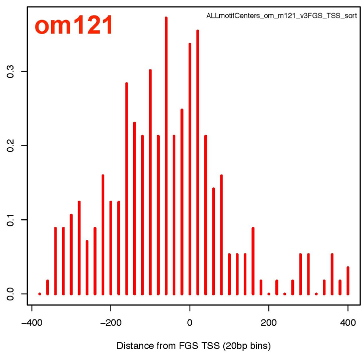	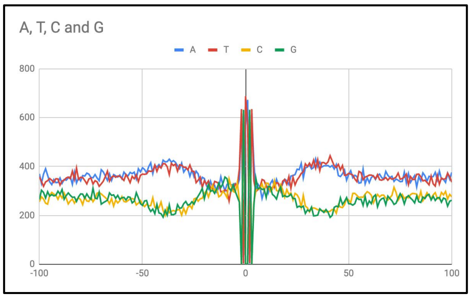
-77		

Average MOA Coverage Around Motif (RPM)

All	Not in Repeats (NR)	In Repeats (IR)
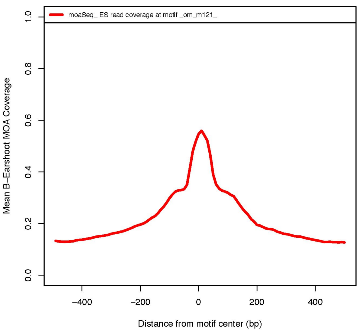	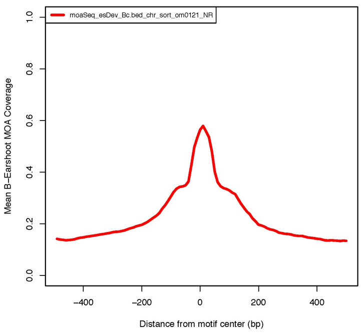	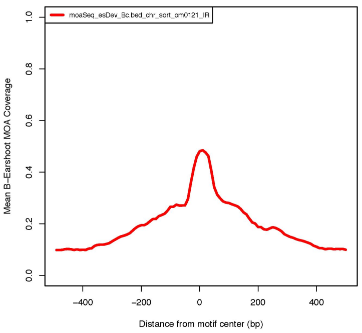

Motif Name	Total Number	In Repeats
om122	1,071	12%
Consensus	ccgCTCCACss	
LOGO	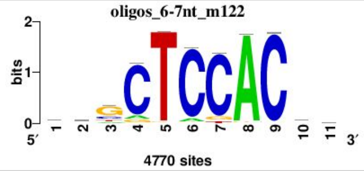	
LOGO RC	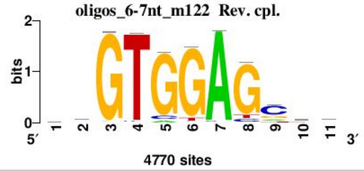	

	Frequency Distr. at TSS (FGS, B73v3)	Average Local Base Frequency (FGS, B73v3)
Median Position Relative to TSS =	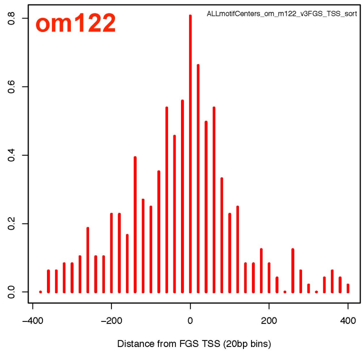	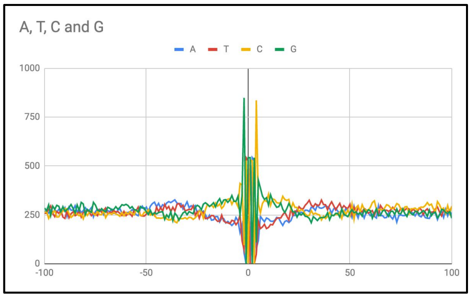
-71		

Average MOA Coverage Around Motif (RPM)

All	Not in Repeats (NR)	In Repeats (IR)
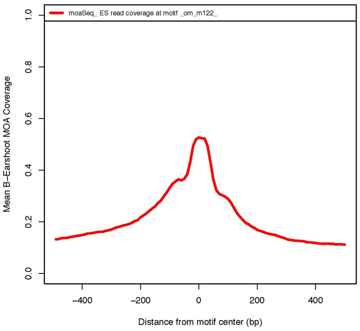	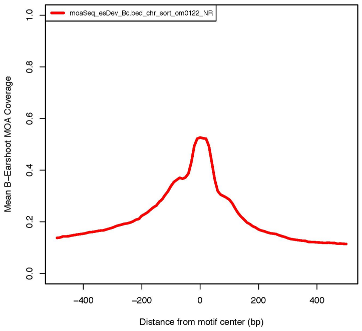	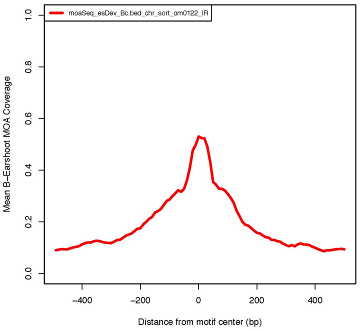

Motif Name	Total Number	In Repeats
om123	1,243	14%
Consensus	smCGAGTCsr	
LOGO	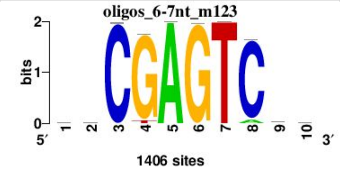	
LOGO RC	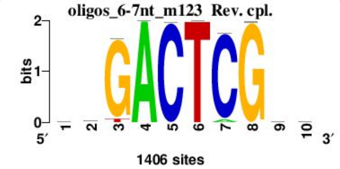	

	Frequency Distr. at TSS (FGS, B73v3)	Average Local Base Frequency (FGS, B73v3)
Median Position Relative to TSS =	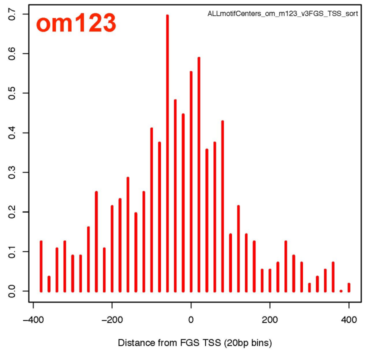	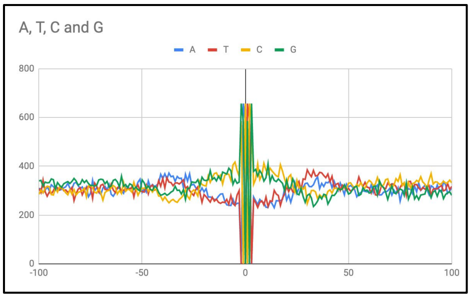
-57		

Average MOA Coverage Around Motif (RPM)

All	Not in Repeats (NR)	In Repeats (IR)
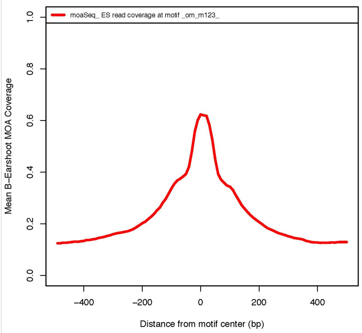	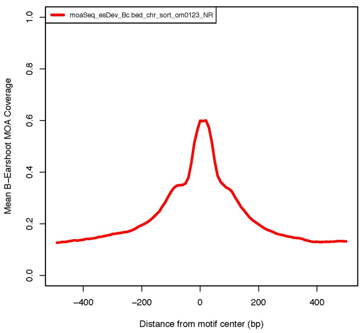	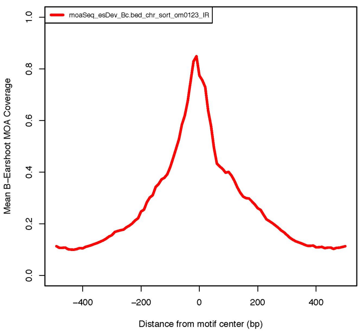

Motif Name	Total Number	In Repeats
**om124**	**429**	**17%**
**Consensus**	ccCATCTCGyc	
**LOGO**	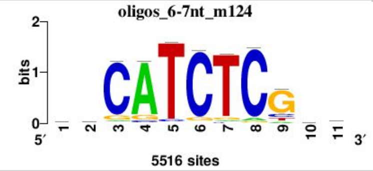	
**LOGO RC**	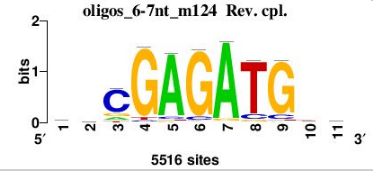	

	Frequency Distr. at TSS (FGS, B73v3)	Average Local Base Frequency (FGS, B73v3)
Median Position Relative to TSS = **-46**	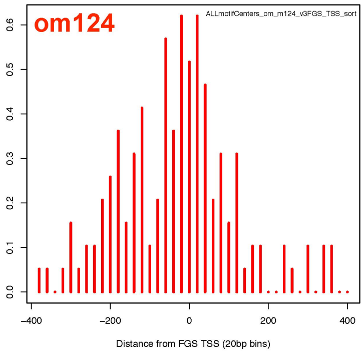	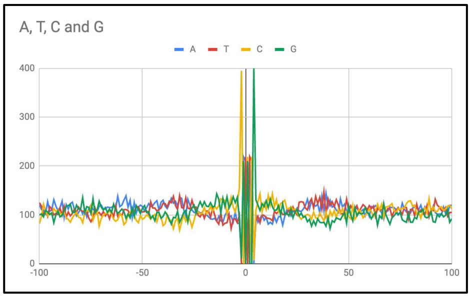

Average MOA Coverage Around Motif (RPM)

All	Not in Repeats (NR)	In Repeats (IR)
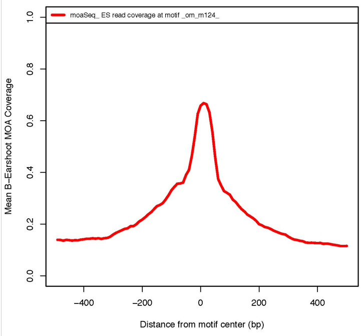	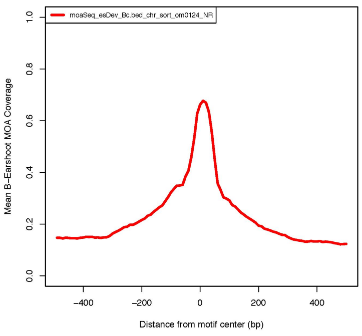	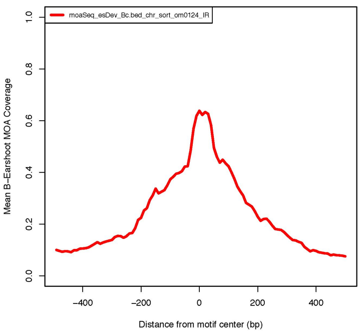

Motif Name	Total Number	In Repeats
om125	1,909	17%
Consensus	ssCCGACCsv	
LOGO	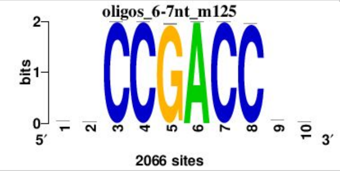	
LOGO RC	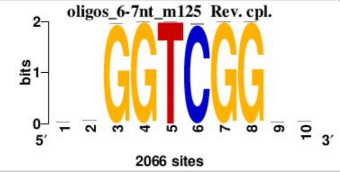	

	Frequency Distr. at TSS (FGS, B73v3)	Average Local Base Frequency (FGS, B73v3)
Median Position Relative to TSS =	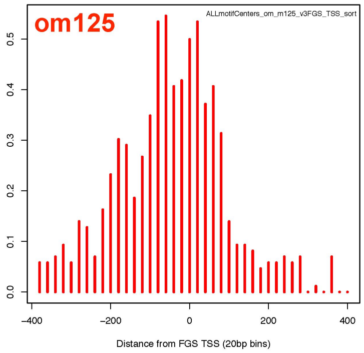	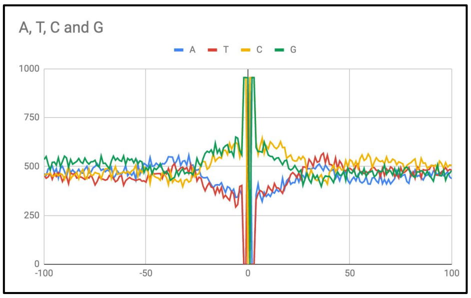
-88		

Average MOA Coverage Around Motif (RPM)

All	Not in Repeats (NR)	In Repeats (IR)
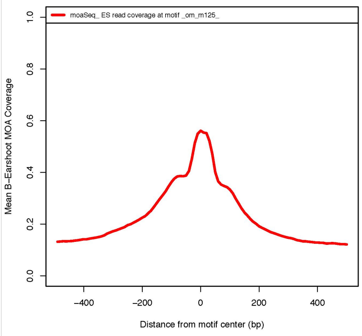	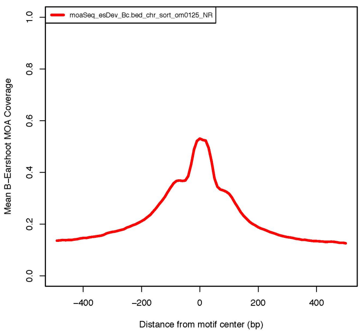	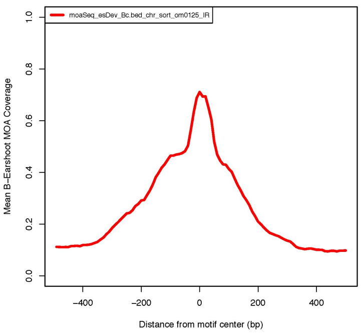

Motif Name	Total Number	In Repeats
om126	1,248	33%
Consensus	wwTATATAww	
LOGO	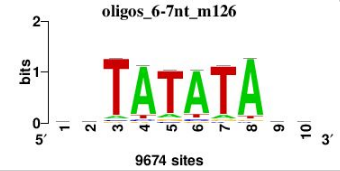	
LOGO RC	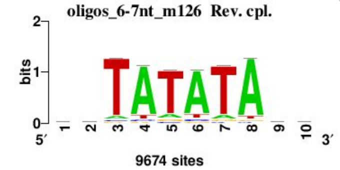	

	Frequency Distr. at TSS (FGS, B73v3)	Average Local Base Frequency (FGS, B73v3)
Median Position Relative to TSS =	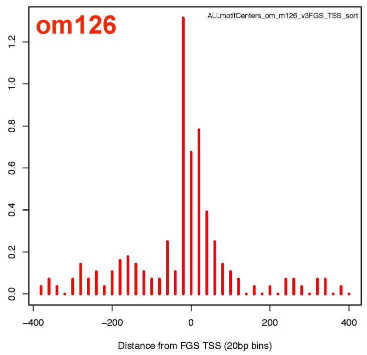	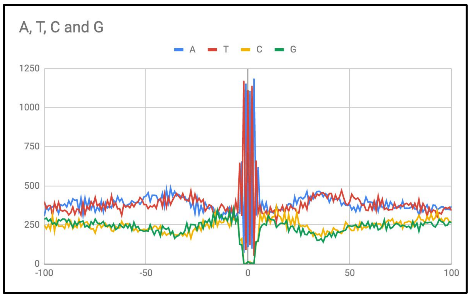
-20		

Average MOA Coverage Around Motif (RPM)		
All	Not in Repeats (NR)	In Repeats (IR)
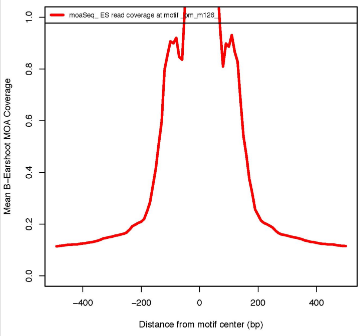	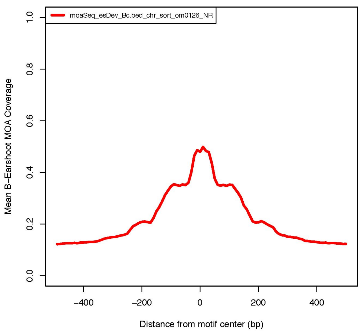	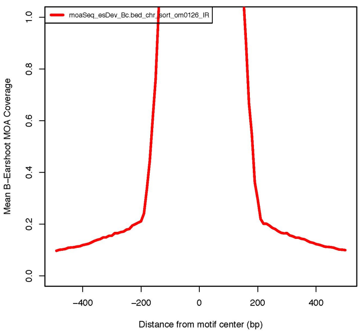

Motif Name	Total Number	In Repeats
om127	1,135	10%

Consensus: rrAAGCGAgv

LOGO

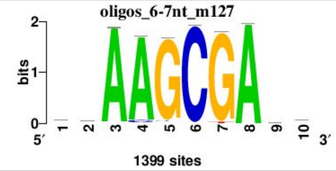

LOGO RC

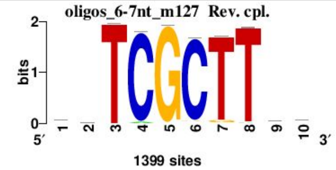

Frequency Distr. at TSS (FGS, B73v3)

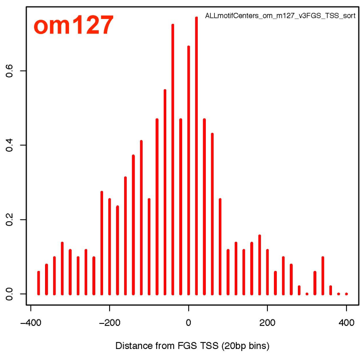

Average Local Base Frequency (FGS, B73v3)

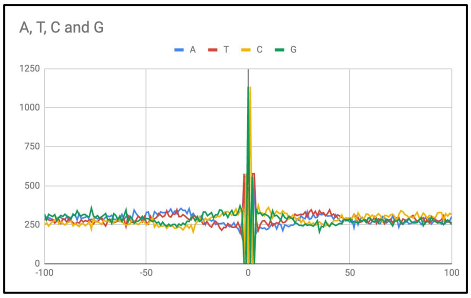

Median Position Relative to TSS = -57

Average MOA Coverage Around Motif (RPM)

All

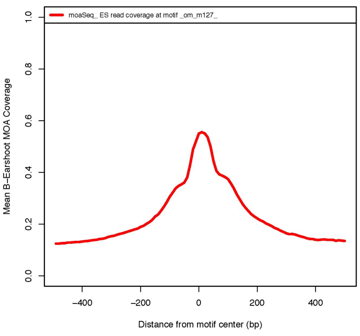

Not in Repeats (NR)

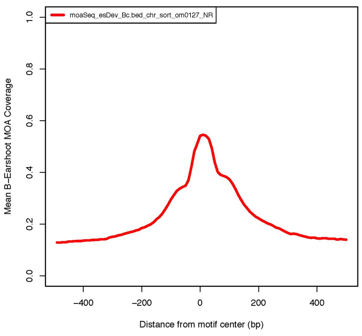

In Repeats (IR)

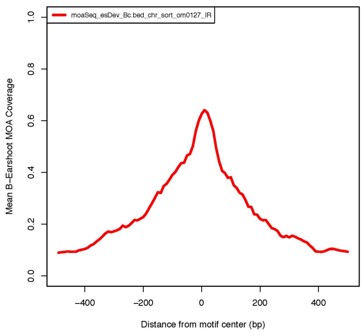

Motif Name	Total Number	In Repeats
om128	4,303	27%
Consensus	rscTGGGCCrss	
LOGO	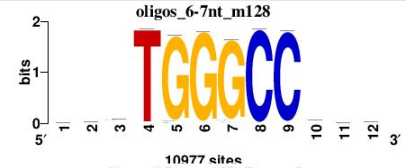	
LOGO RC	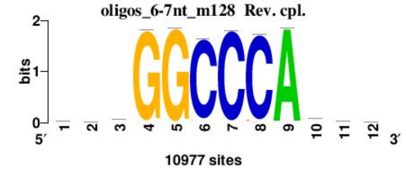	

	Frequency Distr. at TSS (FGS, B73v3)	Average Local Base Frequency (FGS, B73v3)
Median Position Relative to TSS =	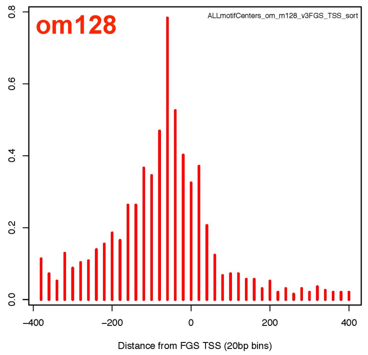	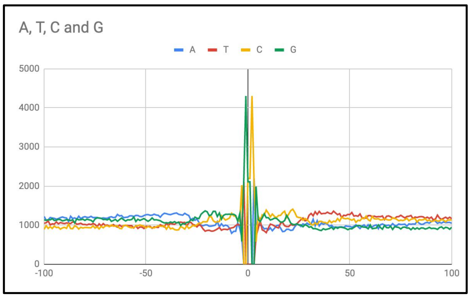
-82		

Average MOA Coverage Around Motif (RPM)

All	Not in Repeats (NR)	In Repeats (IR)
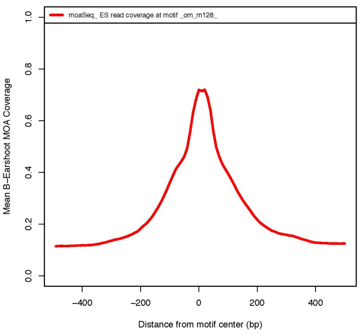	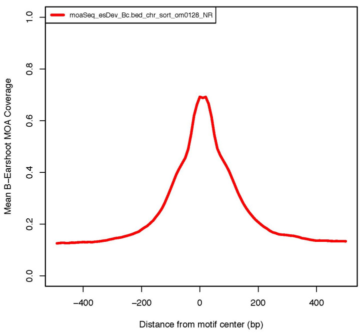	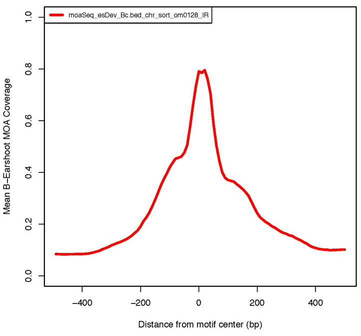

Motif Name	Total Number	In Repeats
om129	2,592	6%
Consensus	ssCGCTGCys	
LOGO	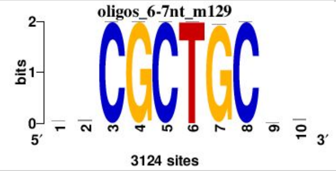	
LOGO RC	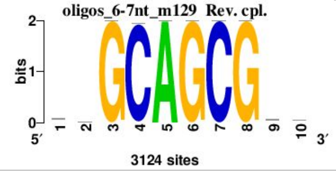	

	Frequency Distr. at TSS (FGS, B73v3)	Average Local Base Frequency (FGS, B73v3)
Median Position Relative to TSS =	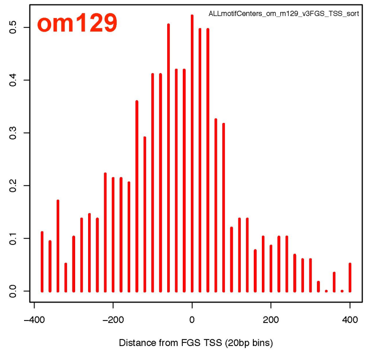	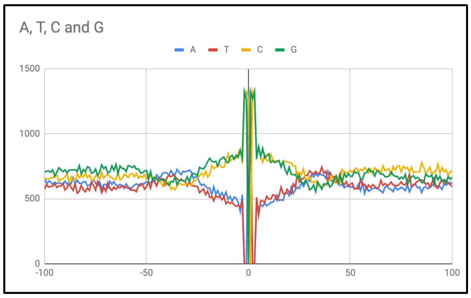
-76		

Average MOA Coverage Around Motif (RPM)

All	Not in Repeats (NR)	In Repeats (IR)
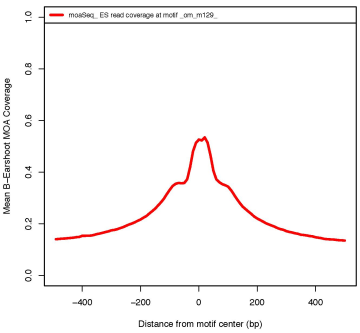	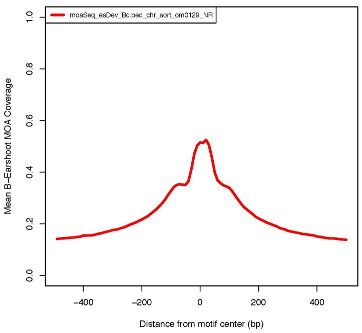	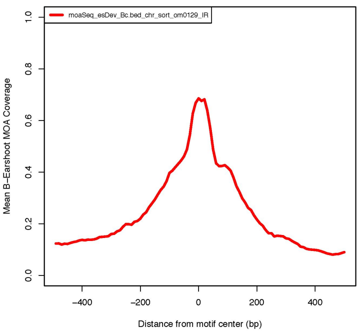

Motif Name	Total Number	In Repeats
om130	130	19%
Consensus	bsTATCCTGbs	
LOGO	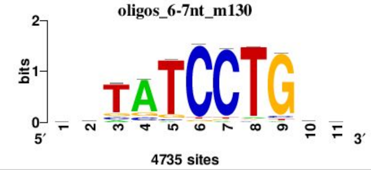	
LOGO RC	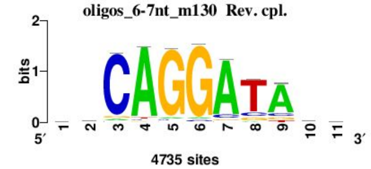	

	Frequency Distr. at TSS (FGS, B73v3)	Average Local Base Frequency (FGS, B73v3)
Median Position Relative to TSS =	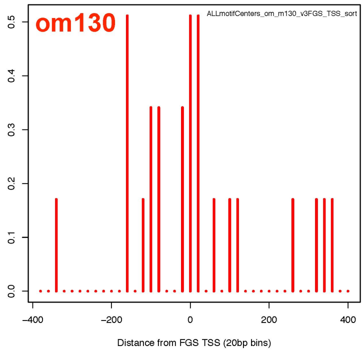	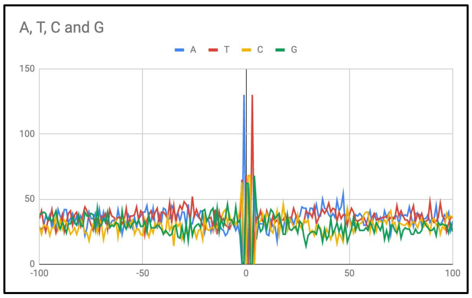
+9		

Average MOA Coverage Around Motif (RPM)

All	Not in Repeats (NR)	In Repeats (IR)
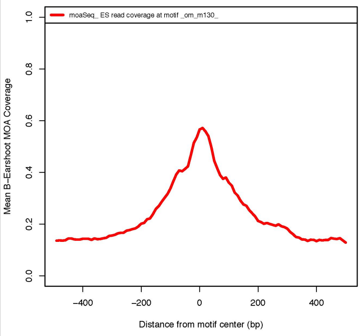	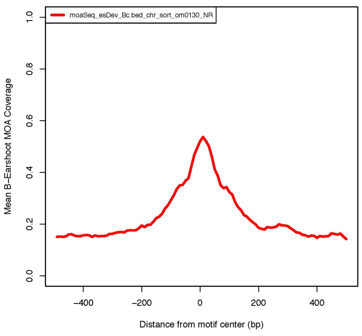	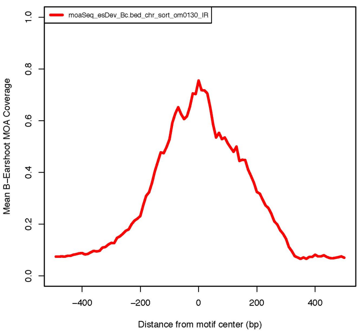

Motif Name	Total Number	In Repeats
om131	2,386	7%
Consensus	yaGCTAGCtr	
LOGO	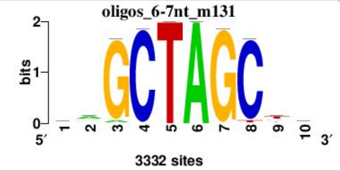	
LOGO RC	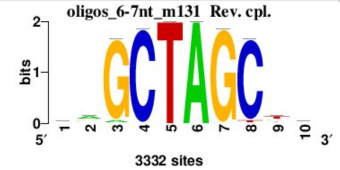	

	Frequency Distr. at TSS (FGS, B73v3)	Average Local Base Frequency (FGS, B73v3)
Median Position Relative to TSS =	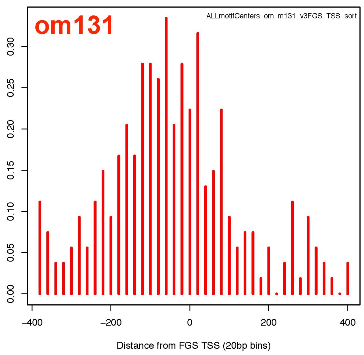	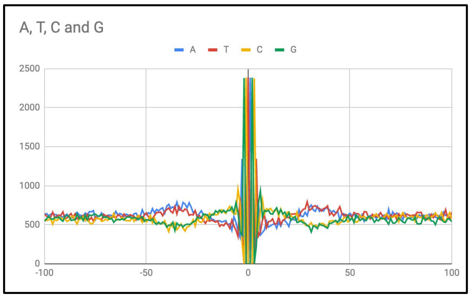
-175		

Average MOA Coverage Around Motif (RPM)

All	Not in Repeats (NR)	In Repeats (IR)
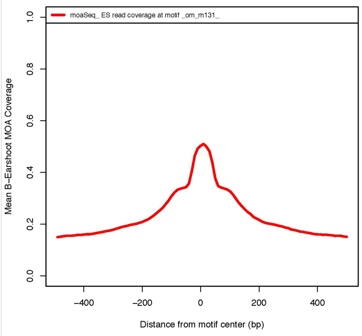	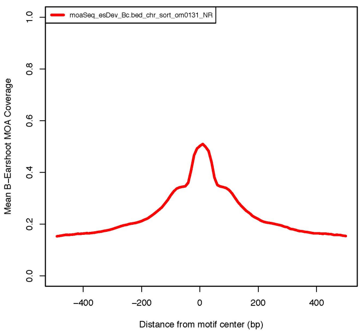	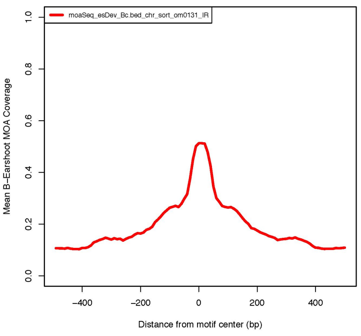

Motif Name	Total Number	In Repeats
om132	1,252	48%
Consensus	wtATTCAAwa	

LOGO

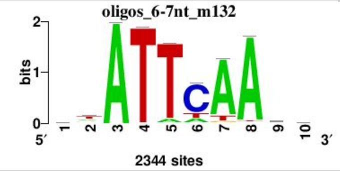

LOGO RC

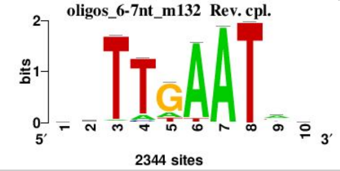

	Frequency Distr. at TSS (FGS, B73v3)	Average Local Base Frequency (FGS, B73v3)
Median Position Relative to TSS = -71	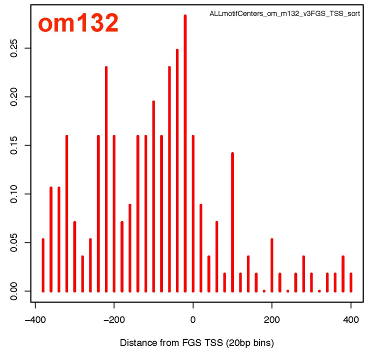	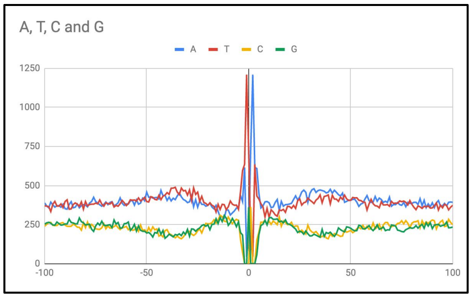

Average MOA Coverage Around Motif (RPM)

All	Not in Repeats (NR)	In Repeats (IR)
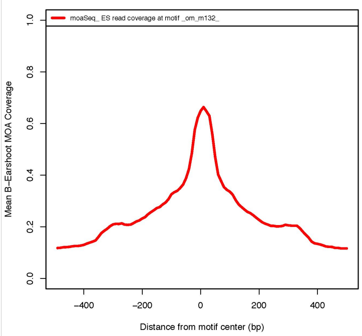	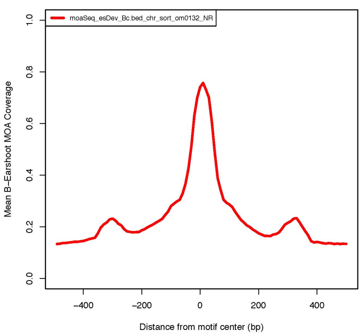	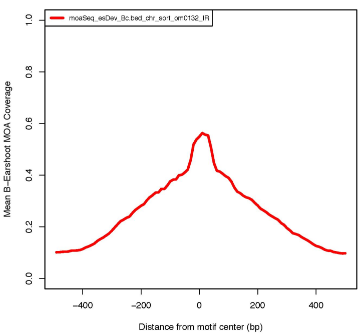

Motif Name	Total Number	In Repeats
om133	1,163	5%
Consensus	ccTCGCCTCaCsy	
LOGO	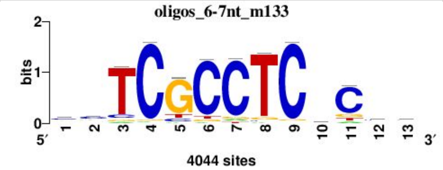	
LOGO RC	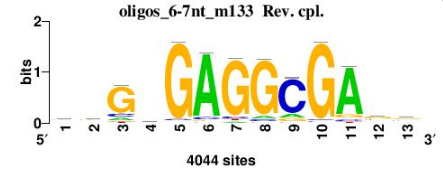	

	Frequency Distr. at TSS (FGS, B73v3)	Average Local Base Frequency (FGS, B73v3)
Median Position Relative to TSS =	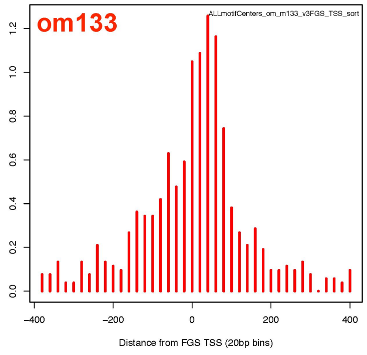	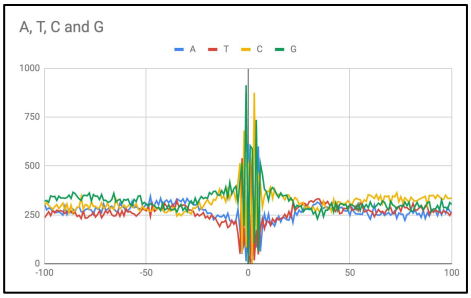
0		

Average MOA Coverage Around Motif (RPM)

All	Not in Repeats (NR)	In Repeats (IR)
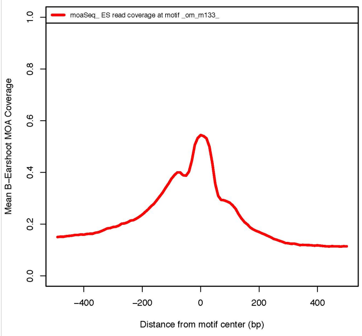	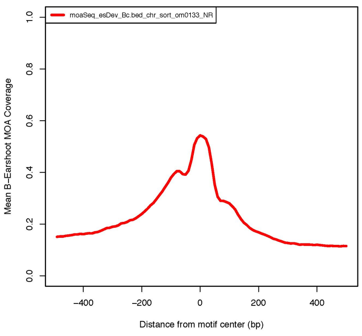	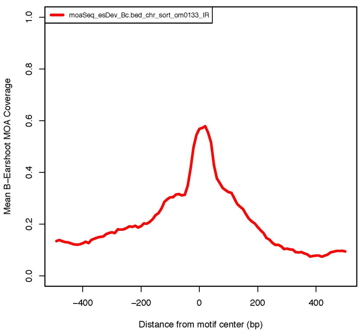

Motif Name	Total Number	In Repeats
om134	2,998	4%
Consensus	mtGCATGCak	
LOGO	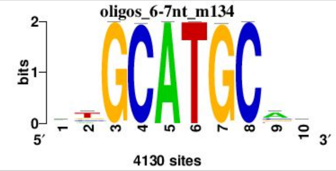	
LOGO RC	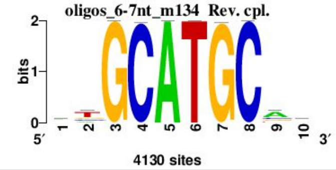	

	Frequency Distr. at TSS (FGS, B73v3)	Average Local Base Frequency (FGS, B73v3)
Median Position Relative to TSS =	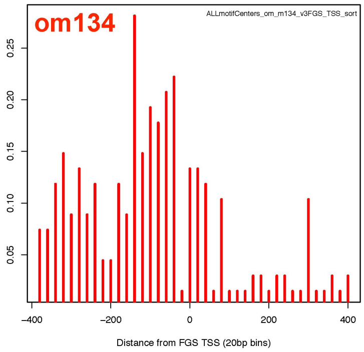	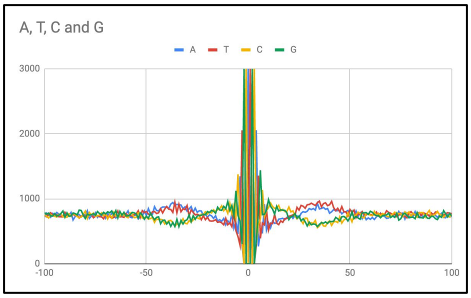
-149		

Average MOA Coverage Around Motif (RPM)

All	Not in Repeats (NR)	In Repeats (IR)
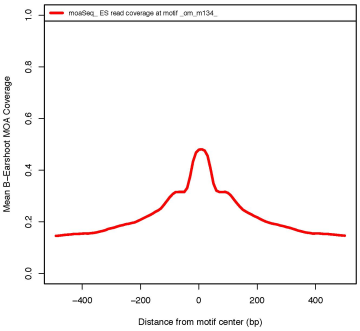	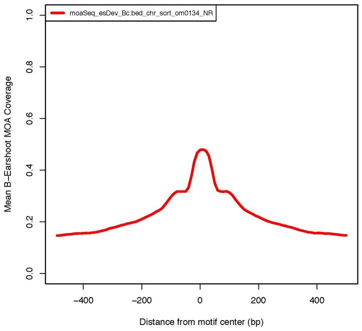	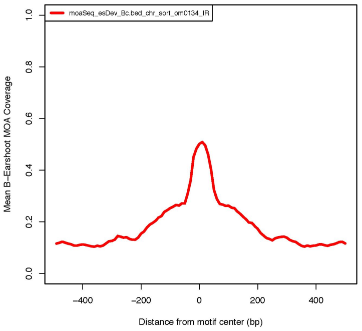

Motif Name	Total Number	In Repeats
om135	377	7%
Consensus	gsCAGGGCGGss	

LOGO

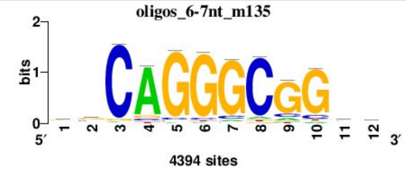

LOGO RC

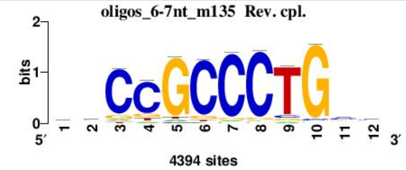

	Frequency Distr. at TSS (FGS, B73v3)	Average Local Base Frequency (FGS, B73v3)
Median Position Relative to TSS = -61		

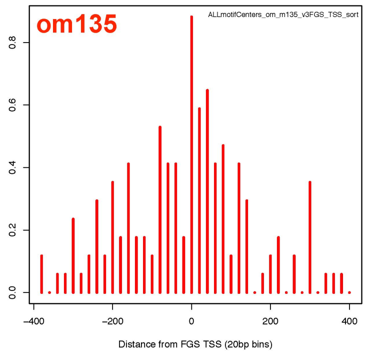

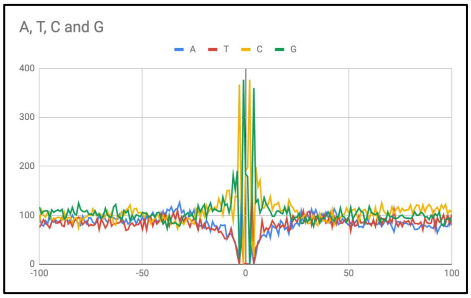

Average MOA Coverage Around Motif (RPM)

All	Not in Repeats (NR)	In Repeats (IR)

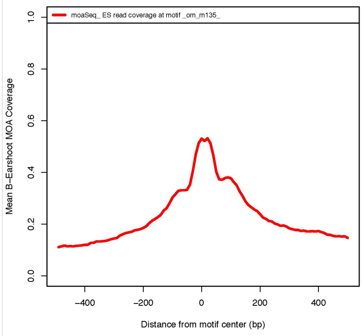

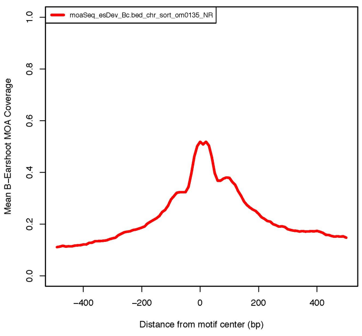

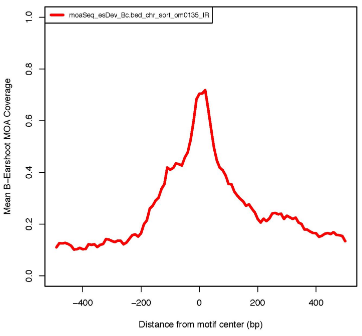

Motif Name	Total Number	In Repeats
om136	753	24%
Consensus	ssGGCtGAGCCGaass	
LOGO	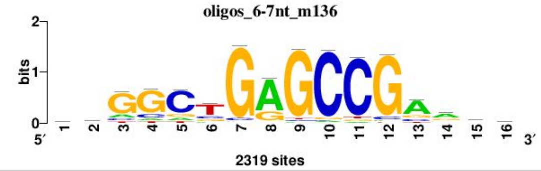	
LOGO RC	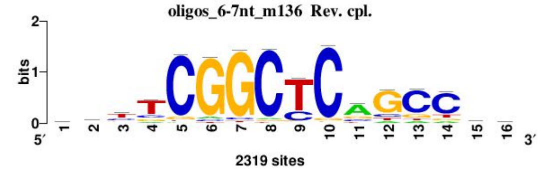	

	Frequency Distr. at TSS (FGS, B73v3)	Average Local Base Frequency (FGS, B73v3)
Median Position Relative to TSS =	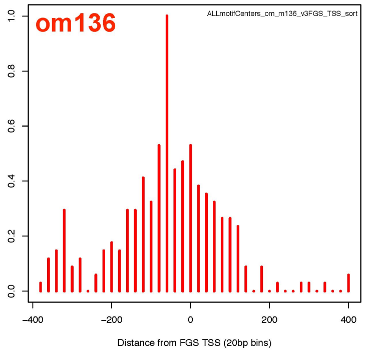	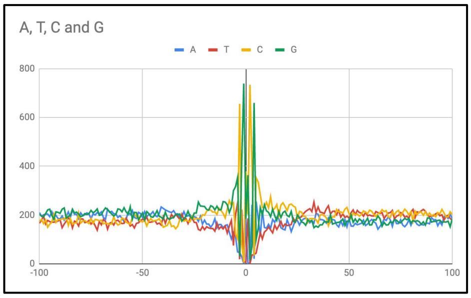
-77		

Average MOA Coverage Around Motif (RPM)

All	Not in Repeats (NR)	In Repeats (IR)
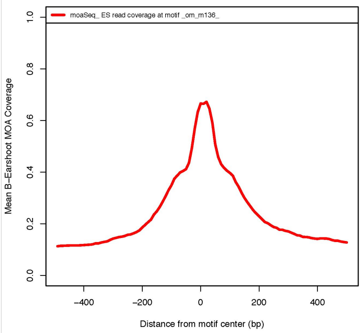	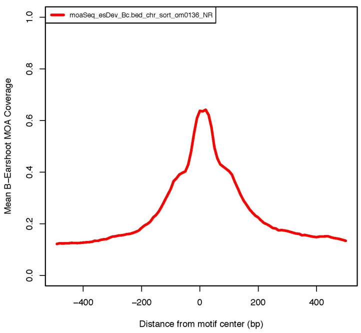	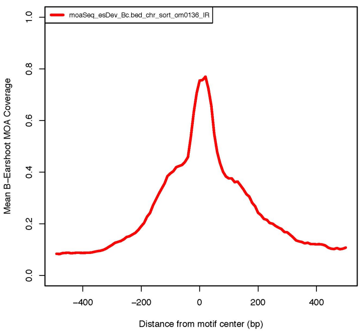

Motif Name	Total Number	In Repeats
om137	756	21%
Consensus	scGGTCCAGsb	
LOGO	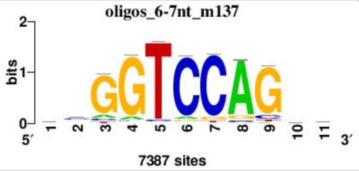	
LOGO RC	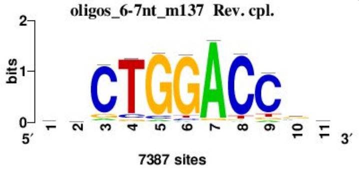	

	Frequency Distr. at TSS (FGS, B73v3)	Average Local Base Frequency (FGS, B73v3)
Median Position Relative to TSS =	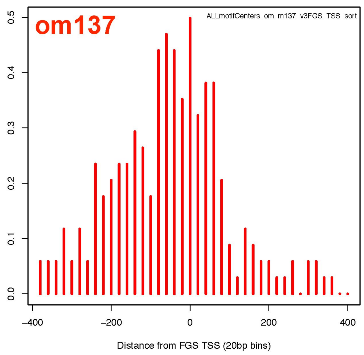	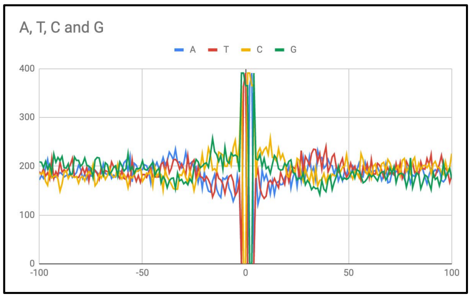
-91		

Average MOA Coverage Around Motif (RPM)		
All	Not in Repeats (NR)	In Repeats (IR)
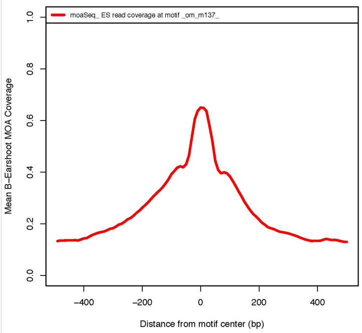	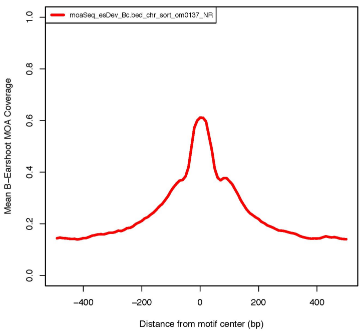	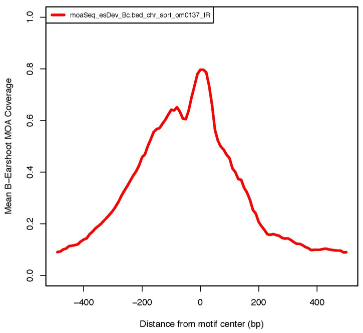

Motif Name	Total Number	In Repeats
om138	810	22%
Consensus	myAAGTCAyr	
LOGO	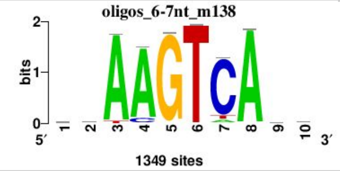	
LOGO RC	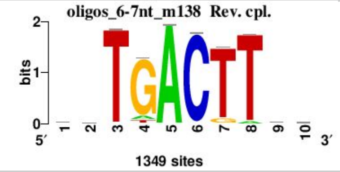	

	Frequency Distr. at TSS (FGS, B73v3)	Average Local Base Frequency (FGS, B73v3)
Median Position Relative to TSS =	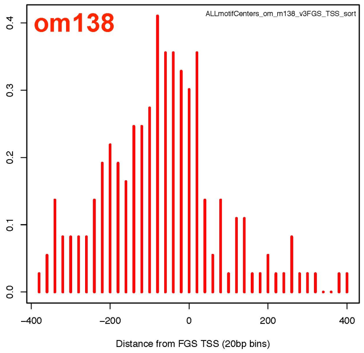	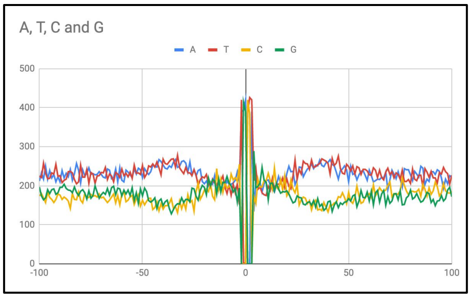
-89		

Average MOA Coverage Around Motif (RPM)

All	Not in Repeats (NR)	In Repeats (IR)

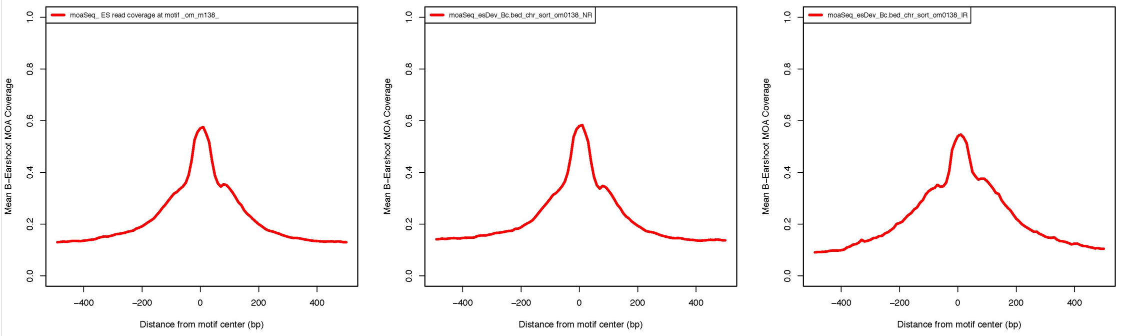

Motif Name	Total Number	In Repeats
om139	2,850	15%
Consensus	scCCACACvm	
LOGO	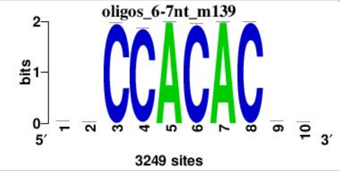	
LOGO RC	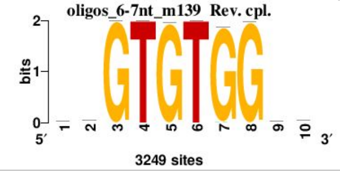	

	Frequency Distr. at TSS (FGS, B73v3)	Average Local Base Frequency (FGS, B73v3)
Median Position Relative to TSS = -64	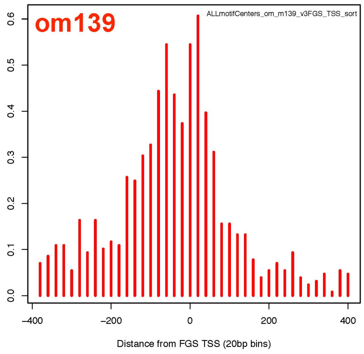	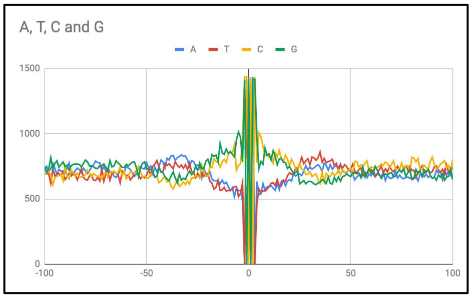

Average MOA Coverage Around Motif (RPM)

All	Not in Repeats (NR)	In Repeats (IR)
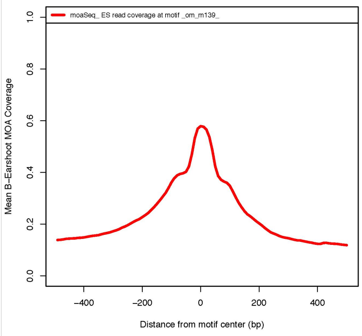	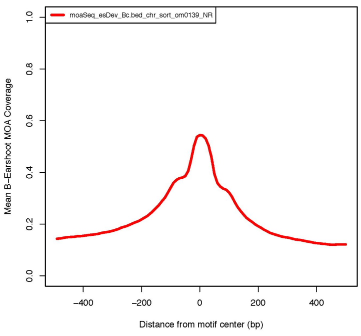	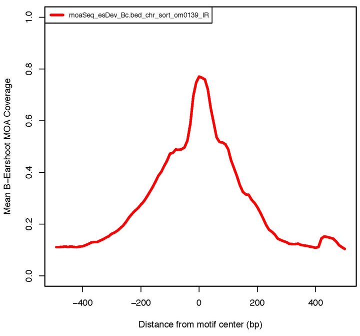

Motif Name	Total Number	In Repeats
om140	4,514	25%
Consensus	cymGGCCCAgsy	
LOGO	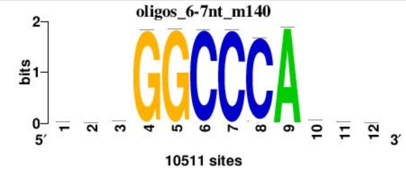	
LOGO RC	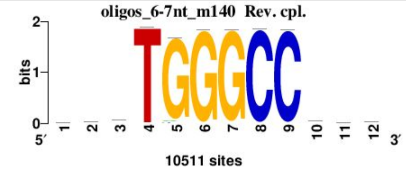	

	Frequency Distr. at TSS (FGS, B73v3)	Average Local Base Frequency (FGS, B73v3)
Median Position Relative to TSS =	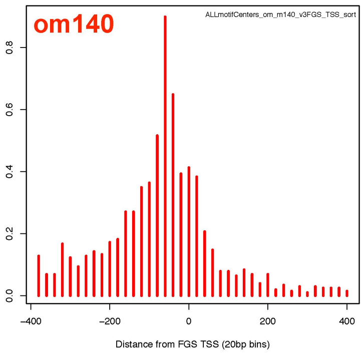	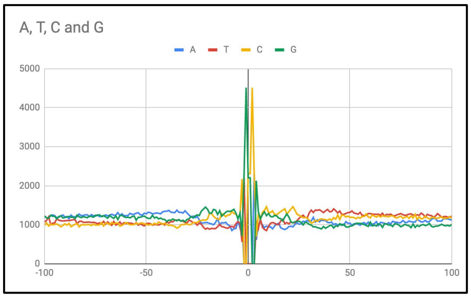
-81		

Average MOA Coverage Around Motif (RPM)		
All	Not in Repeats (NR)	In Repeats (IR)
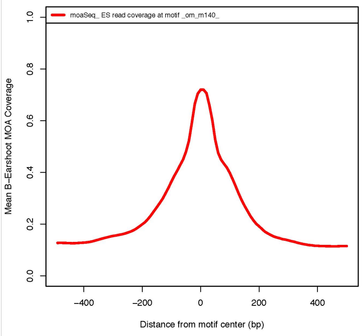	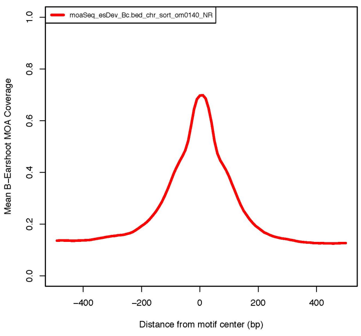	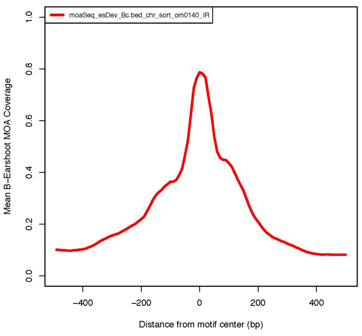
