# Supplementary material for: The native cistrome and sequence motif families of the maize ear
Source: PLoS Genet. 2021 Aug 12;17(8):e1009689. doi: 10.1371/journal.pgen.1009689 (PMC8360572; doi:10.1371/journal.pgen.1009689)
Supplement: S11 File — Read-normalized FRENTERs profiles from combined bio-replicate 1 libraries of earshoot MOA-seq aligned to B73v5 in 5 bp window bins. The bigwig file is published and available via FigShare, https://doi.org/10.6084/m9.figshare.14411849.v1. (DOC) [file pgen.1009689.s018.doc]

**MOA earshoot MFs rep1 bigwig file for B73v5.** Read-normalized FRENTERs profiles from combined bio-replicate 1 libraries of earshoot MOA-seq aligned to B73v5 in 5 bp window bins. The bigwig file is published and available via FigShare, <https://doi.org/10.6084/m9.figshare.14411849.v1>.

DataCite:

Bass, Hank (2021): S11 File. MOA earshoot MFs rep1 bigwig file for B73v5. figshare. Dataset. https://doi.org/10.6084/m9.figshare.14411849.v1.
