# Supplementary material for: The native cistrome and sequence motif families of the maize ear
Source: PLoS Genet. 2021 Aug 12;17(8):e1009689. doi: 10.1371/journal.pgen.1009689 (PMC8360572; doi:10.1371/journal.pgen.1009689)
Supplement: S14 File — Read-normalized coverage profile from MNase partial digest DNA control aligned to B73v5 in 20 bp window bins and used as input for peak segmentation. The bigwig file is published and available via FigShare, https://doi.org/10.6084/m9.figshare.14412551.v1. (DOC) [file pgen.1009689.s021.doc]

**B73 MNase Control COVERAGE bigwig file for B73v5.** Read-normalized coverage profile from MNase partial digest DNA control aligned to B73v5 in 20 bp window bins and used as input for peak segmentation. The bigwig file is published and available via FigShare, <https://doi.org/10.6084/m9.figshare.14412551.v1>.

DataCite:

Bass, Hank (2021): S14 File. B73 MNase Control COVERAGE bigwig file for B73v5.. figshare. Dataset. https://doi.org/10.6084/m9.figshare.14412551.v1.
