# Supplementary material for: The native cistrome and sequence motif families of the maize ear
Source: PLoS Genet. 2021 Aug 12;17(8):e1009689. doi: 10.1371/journal.pgen.1009689 (PMC8360572; doi:10.1371/journal.pgen.1009689)
Supplement: S15 File — Read-normalized FRENTER (fragment center) profile from MNase partial digest DNA control aligned to B73v5 in 5 bp window bins and used as input for peak segmentation. The bigwig file is published and available via FigShare, https://doi.org/10.6084/m9.figshare.14412851.v1. (DOC) [file pgen.1009689.s022.doc]

**B73 MNase Control fragment centers bigwig file for B73v5.** Read-normalized FRENTER (fragment center) profile from MNase partial digest DNA control aligned to B73v5 in 5 bp window bins and used as input for peak segmentation. The bigwig file is published and available via FigShare, <https://doi.org/10.6084/m9.figshare.14412851.v1>.

DataCite:

Bass, Hank (2021): S15 File. B73 MNase Control fragment centers bigwig file for B73v5.. figshare. Dataset. https://doi.org/10.6084/m9.figshare.14412851.v1.
