# Supplementary material for: The native cistrome and sequence motif families of the maize ear
Source: PLoS Genet. 2021 Aug 12;17(8):e1009689. doi: 10.1371/journal.pgen.1009689 (PMC8360572; doi:10.1371/journal.pgen.1009689)
Supplement: S3 Zip File — RSAT report for motif discovery of DYADs type search pattern, using input genomic sequences under MOA-seq maize earshoot MFs with settings: extension to 24bp if <24, n500, min6, max7, pat250, no database, max1000bp. The unzipped file produces folders & files summarizing the motif families. The Zip file is published and available via FigShare, https://doi.org/10.6084/m9.figshare.13012670.v1. (DOC) [file pgen.1009689.s028.doc]

**RSAT report for 75 DYAD motifs found at MFs (frenters) iSegBC7 peaks.** RSAT report for motif discovery of DYADs type search pattern, using input genomic sequences under MOA-seq maize earshoot MFs with settings: extension to 24bp if <24, n500, min6, max7, pat250, no database, max1000bp. The unzipped file produces folders & files summarizing the motif families. The Zip file is published and available via FigShare, <https://doi.org/10.6084/m9.figshare.13012670.v1>.

DataCite:

Bass, Hank (2021): S3 Zip File. RSAT report for 75 DYAD motifs found at MFs (Frenters) iSegBC7 peaks.. figshare. Online resource. https://doi.org/10.6084/m9.figshare.13012670.v1.
