# Supplementary material for: The native cistrome and sequence motif families of the maize ear
Source: PLoS Genet. 2021 Aug 12;17(8):e1009689. doi: 10.1371/journal.pgen.1009689 (PMC8360572; doi:10.1371/journal.pgen.1009689)
Supplement: S7 Zip File — Bed files of peak segments called by iSeg at a series of cutoffs (bc 1, 2, 3, 4, 5, 7, and 9) for MNase DNA-only control digests coverage (COV) or MF-like (FRENTERs) profiles as the input. Included are the 7-series output iSeg BED files for each input, and a readme (txt) file. (DOC) [file pgen.1009689.s032.doc]

**Zipped BED files of iSeg peaks, bigbed files for MNase CONTROL coverage (COV) and MFs (FRENTERs) on maize B73v5.** Bed files of peak segments called by iSeg at a series of cutoffs (bc 1, 2, 3, 4, 5, 7, and 9) for MNase DNA-only control digests coverage (COV) or MF-like (FRENTERs) profiles as the input. Included are the 7-series output iSeg BED files for each input, and a readme (txt) file. The Zip file is published and available via FigShare, <https://doi.org/10.6084/m9.figshare.14428298.v1>.

DataCite:

Bass, Hank (2021): S7 Zip File. Zipped BED files of iSeg peaks, bigbed files for MNase CONTROL coverage (COV) and MFs (FRENTERs) on maize B73v5. figshare. Dataset. https://doi.org/10.6084/m9.figshare.14428298.v1.
